# Role of non‐coding variants in cardiovascular disease

**DOI:** 10.1111/jcmm.17762

**Published:** 2023-05-15

**Authors:** Katayoun Heshmatzad, Niloofar Naderi, Majid Maleki, Shiva Abbasi, Serwa Ghasemi, Nooshin Ashrafi, Amir Farjam Fazelifar, Mohammad Mahdavi, Samira Kalayinia

**Affiliations:** ^1^ Rajaie Cardiovascular Medical and Research Center Iran University of Medical Sciences Tehran Iran; ^2^ Cardiogenetic Research Center, Rajaie Cardiovascular Medical and Research Center Iran University of Medical Sciences Tehran Iran

**Keywords:** arrhythmia, cardiomyopathy, cardiovascular diseases, congenital heart disease, hypercholesterolemia, hypertension, non‐coding variants

## Abstract

Cardiovascular diseases (CVDs) constitute one of the significant causes of death worldwide. Different pathological states are linked to CVDs, which despite interventions and treatments, still have poor prognoses. The genetic component, as a beneficial tool in the risk stratification of CVD development, plays a role in the pathogenesis of this group of diseases. The emergence of genome‐wide association studies (GWAS) have led to the identification of non‐coding parts associated with cardiovascular traits and disorders. Variants located in functional non‐coding regions, including promoters/enhancers, introns, miRNAs and 5′/3′ UTRs, account for 90% of all identified single‐nucleotide polymorphisms associated with CVDs. Here, for the first time, we conducted a comprehensive review on the reported non‐coding variants for different CVDs, including hypercholesterolemia, cardiomyopathies, congenital heart diseases, thoracic aortic aneurysms/dissections and coronary artery diseases. Additionally, we present the most commonly reported genes involved in each CVD. In total, 1469 non‐coding variants constitute most reports on familial hypercholesterolemia, hypertrophic cardiomyopathy and dilated cardiomyopathy. The application and identification of non‐coding variants are beneficial for the genetic diagnosis and better therapeutic management of CVDs.

## 1 INTRODUCTION

Cardiovascular diseases (CVDs) are the leading cause of death and account for 31% of mortality, worldwide.^1^ Some progressive pathologies linked to cardiovascular diseases are familial hypercholesterolemia, different types of cardiomyopathies, thoracic/aortic aneurysms, congenital heart diseases, coronary artery diseases, heart failure^2^ and strokes.^3^, ^4^ Despite the promising results of conventional pharmacological treatments, cardiovascular diseases still have poor prognoses.^5^ Many factors are associated with cardiovascular disease pathogenesis. Among them, the genetic component is a beneficial tool for the risk stratification of cardiovascular disease development. Improvements in sequencing technologies have conferred not only better clinical management and diagnosis of genetic disorders but also a better understanding of genetic disorders with unknown mechanisms.^6^ Before the completion of the Human Genome Project, genes associated with rare Mendelian forms of cardiovascular diseases had been identified. Recent years have found the identification of hundreds of loci by cardiovascular genome‐wide association studies (GWAS) and the formation of a general concept that common genetic associations located in the non‐coding parts of the genome have a considerable prevalence. A significant portion of loci associated with cardiovascular traits and disorders is not in linkage disequilibrium (LD) with causative coding regions and elements.^7^ The majority of non‐coding GWAS variants that play a significant functional role in gene regulation occur within the regions of open chromatin.^4^, ^7^, ^8^, ^9^ Research has indicated that even rare variations contribute to the development of both arrhythmias and cardiomyopathies.^10^, ^11^ More recent GWAS studies have unravelled the genetic architecture of more prevalent forms of cardiovascular diseases such as coronary artery diseases and atrial fib F2 cross lation and contributed to a better understanding of pathophysiological pathways involved in cardiovascular diseases.^12^ The era of cardiovascular genomics has ushered in two distinguished goals: understanding molecular pathways and implementing the knowledge to expand the field of personalized medicine.^13^

Limitations in mapping methods create problems in identifying variants located in non‐coding regions. Two main challenges of mapping are the amount of recombination and allelic diversity. Only allelic diversity within the recombinant inbred line^14^ population and F2 cross can be assayed through mapping.^15^ RIL is the result of sibmating progeny or continuous selfing of F2 population until we have complete homozygosity.^16^ The F2 cross is the offspring of two sister seedlings from the F1 hybrid, or the following generation.

What also continues to present a challenge is functional annotation.^17^ Accordingly, we conducted the present study to collect all non‐coding variants associated with different forms of cardiovascular diseases through a comprehensive review. We herein discuss the necessity of considering not only coding variants but also non‐coding variants in the risk of susceptibility to cardiovascular diseases.

### 1.1 Variant region: coding and non‐coding variants

Deoxyribonucleic acid (DNA) is composed of both genic and intergenic regions. Exonic regions encode amino acids and are generally well conserved.^18^ Whole‐exome sequencing and targeted sequencing of coding regions of the human genome have helped identify both causative frameshift mutations and nonsense and missense variants associated with human disorders.^19^ GWAS studies have also indicated that single‐nucleotide variants/polymorphisms (SNVs/SNPs) located in enhancer elements, DNase hypersensitivity regions and chromatin marks known as ‘functional non‐coding regions’ are associated with complex diseases.^20^, ^21^

## 2 SEARCH STRATEGY

**FIGURE 1:**
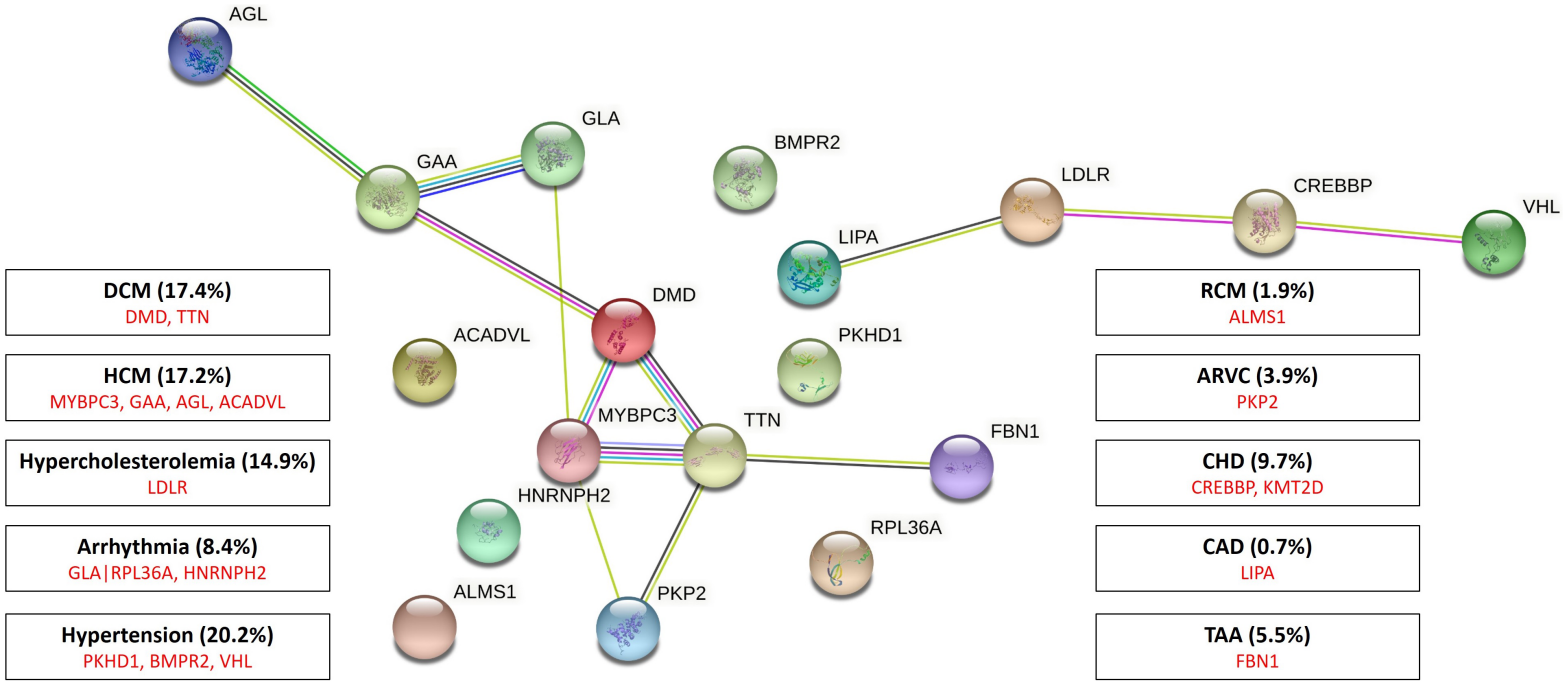
The image presents genes with the most frequently reported non‐coding variants, the total percentage of non‐coding variants in any type of cardiovascular diseases is shown in parentheses. ARVC, arrhythmogenic right ventricular cardiomyopathy; CAD, coronary artery disease; CHD, congenital heart disease; DCM, dilated cardiomyopathy; HCM, hypertrophic cardiomyopathy; RCM, restrictive cardiomyopathy; TAA, thoracic aortic aneurysm.

In the present study, a comprehensive and systematic search was conducted on literatures and Clinvar database in order to fulfil all reported non‐coding variants of different cardiovascular diseases such as dyslipidaemia, familial hypercholesterolemia, different types of cardiomyopathies, congenital heart diseases, thoracic aortic aneurysms and dissections, coronary artery diseases and strokes and sudden cardiac death. All genes and non‐coding variants involved in each disorder were checked separately in ClinVar, dbSNP, Iranome, 1000 Genomes Project, gnomAD and TOPMed databases. Nomenclature for variants was also confirmed according to the recommendations of the Human Genetic Variation Society (HGVS) (http://varnomen.hgvs.org/). In addition, we conducted a comprehensive search on published articles on non‐coding variants and some variants were extracted through this method. After collecting all the variants, total number and common variant of each separate gene were reported in our study (Figure 1).

## 3 NON‐CODING VARIANTS IN DYSLIPIDAEMIA AND FAMILIAL HYPERCHOLESTEROLEMIA

Familial hypercholesterolemia (OMIM ID‐143890) is an inherited disease with an autosomal dominant (AD) inheritance mode that is mainly the consequence of defects in three main genes (viz, the LDL receptor [*LDLR*], apolipoprotein B‐100 [*APOB*] and proprotein convertase subtilisin/kexin type 9 [*PCSK9*]). Defects in the *LDL‐receptor adapter protein* (*LDLRAP*) gene lead to the recessive inheritance (AR) form of familial hypercholesterolemia. Patients with familial hypercholesterolemia have higher levels of low‐density lipoprotein (LDL). If such patients do not receive pharmacological treatments, they develop different disorders, including coronary heart diseases, premature and progressive atherosclerotic cardiovascular diseases, xanthomas, myocardial infarction and premature coronary artery diseases.^22^, ^23^, ^24^, ^25^ Blood lipid levels depend on many factors, including the type of the variant, lifestyle and the impact of other associated variants. Further, homozygote carriers have higher lipid levels (13 mmol/L) than heterozygous carriers (9–12 mmol/L).^26^, ^27^ The first epidemiologic study, conducted by Goldstein et al., reported a frequency rate of 1:1500 for heterozygous familial hypercholesterolemia carriers; nevertheless, this rate has changed over time. A large‐scale study in 2012 reported a much higher prevalence for heterozygous familial hypercholesterolemia patients (1:1250), and this higher frequency has also been replicated and confirmed in other familial hypercholesterolemia patients of European ethnicity.^28^, ^29^ The homozygous form is very rare, and recent studies have estimated a prevalence rate of 1:160,000 to 1:300,000.^30^ As was mentioned, most familial hypercholesterolemia patients are carriers of candidate causal variants in *LDLR*, *APOB* and rarely *PCSK9*; however, variations in other genes such as *STAP1* and *APOE* have been reported.^31^ Novel rare variants associated with familial hypercholesterolemia in genes such as lysosomal lipase A (*LIPA*), patatin‐like phospholipase domain containing 5 (*PNPLA5*), hepatic lipase (*LIPC*), cytochrome P450 (*CYP27A1*), steroid 27‐hydroxylase, cerebrotendinous xanthomatosis (*subfamily XXVIIA*), polypeptide 1, endothelial lipase (*LIPG*) and cytochrome P450 family 7 subfamily A member 1 (*CYP7A1*) have been identified through pedigree analysis.^14^, ^32^, ^33^, ^34^, ^35^ In addition to the single‐gene form of familial hypercholesterolemia, patients with pseudo‐familial hypercholesterolemia, also termed ‘polygenic familial hypercholesterolemia’, have been described. This term was first used for patients who met the familial hypercholesterolemia criteria clinically and biochemically, while there were no variations in *LDLR*, *APOB* and *PCSK9*. Talmud et al. proposed a model called ‘12 SNP’, which comprised 12 common SNVs in 11 genes: *NYNRIN*, *APOB*, *ST3GAL4*, *SORT1*, *ABCG8 HFE*, *SLC‐22*, *SLC‐22*, *LDLR*, *APOE*, *MYLIP* and *PCSK9*. These variations were discovered through GWAS conducted by the Lipids Genetics Consortium (GLGC; http://lipidgenetics.org).^36^ Two effective strategies for the detection of familial hypercholesterolemia cases are genetic testing and family cascade screening, which also assist in distinguishing monogenic forms from polygenic or sporadic hypercholesterolemia. Since 2008, the implementation of next‐generation sequencing (NGS), as a high‐throughput technology, has also yielded promising results for familial hypercholesterolemia patients.^36^, ^37^ Sequencing in patients and high‐risk families affected with rare monogenic lipid diseases has revealed not only a remarkable number of rare coding mutations but also the necessary pathways involved in lipid metabolism.^38^, ^39^, ^40^ GWAS studies have also shown that blood lipid traits such as high‐density lipoprotein cholesterol (HDL‐C), total cholesterol levels of low‐density lipoprotein cholesterol (LDL‐C) and triglycerides have heritability rates of between 40% and 60% among different populations. Although GWAS studies have identified more than 100 common lipid‐associated variants, these variants constitute only a small portion (10%–14%) of variations in the lipid phenotype.^41^, ^42^ More recent studies on the rare variants of complex traits due to the facility of WES and its data interpretation have focused mainly on the coding regions of the genome,^43^ which explains why the effects of the non‐coding part of the genome have remained unknown.

The implementation of WES in recent years has proffered better clinical management and diagnosis of less common genetic disorders. Despite such promising improvements, however, WES is capable of investigating and examining only 30% of the genome, which underscores the role of the other parts of the genome such as regulatory regions.^6^, ^44^, ^45^, ^46^ There have also been reports of distal enhancers and alterations in the 3D genome structure.^17^, ^47^ Therefore, the next milestones in the interpretation of the human genome sequence will focus on the remaining 98% of genome regions.^48^ A study by Igartua et al. in 2017 provided strong support for the association between rare non‐coding variants and lipid traits. That GWAS study recruited 98 Hutterites (European descent), and imputation indicated that 660,238 SNPs that were either rare (frequency <1%) or absent in European ethnicity were more common in Hutterites (frequency >1%). The results also revealed 2 novel non‐coding rare variants. The first identified variant (viz, rs17242388 in *LDLR*) was associated with LDL‐C, and the second variant, located between *GOT2* and *APOOP5* (viz, rs189679427), had a robust association with HDL‐C. The third variation was rs138326449, which was previously replicated as a splice variant in *APOC3* and was associated with triglycerides and HDL‐C.^49^ Rare non‐coding variants are sometimes a reasonable explanation for AD familial hypercholesterolemia traits. In another whole‐genome sequencing (WGS) study on a large family clinically diagnosed with familial hypercholesterolemia, in whom no mutations were detected in the coding regions of *LDLR*, *APOB* and *PCSK9*, a novel *LDLR* deep intronic variant (viz, c.2140+103G>T) co‐segregated with LDL‐C and the familial hypercholesterolemia phenotype.^50^ The impact of regulatory elements on lipid traits has also been reported. Weissglas‐Volkov et al.^51^ in 2009 identified an rs1424032 SNP in a highly conserved non‐coding region of *APOB* that functioned as a regulatory element and contributed to serum apolipoprotein B levels. In the past 5 years, the main focus of genome analysis has been on three regions: cis‐ and trans‐regulatory elements, enhancers or promoters, and regulatory transcribed non‐coding regions.^52^, ^53^, ^54^ Regulatory elements, which are mainly located in non‐coding regions, play a role in the gene expression of various cell types and act through interactions with various transcription factors (TFs).^53^

In our search, we found 408 variants associated with familial hypercholesterolemia. The majority of the reported variants were for *LDLR* (328/408, 80.39%). Moreover, *LDLR* had an overlap with another gene, *AS1*, and 34 variants that reside in this region (34/408, 8.33). Three variants were reported for *LDLRAP1*, which is important in the rare forms of familial hypercholesterolemia (Table S1).

## 4 NON‐CODING VARIANTS IN CARDIOMYOPATHIES

Primary and secondary cardiomyopathies represent a clinically heterogeneous group of cardiac disorders, classified based on two important factors: ventricular function and morphology. These disorders are also defined by abnormal myocardial structure and/or function when conditions such as ischemic heart diseases or abnormal loading conditions such as coronary artery diseases, hypertension and valvular heart diseases do not exist.^55^, ^56^ Primary and secondary cardiomyopathies are categorized into four major subgroups: dilated cardiomyopathy (DCM), hypertrophic cardiomyopathy (HCM), left ventricular noncompaction, restrictive cardiomyopathy (RCM) and arrhythmogenic right ventricular cardiomyopathy (ARVC).^56^, ^57^ NGS and GWAS studies on patients with cardiomyopathies have indicated a significant number of defects in sarcomeres, mechanotransduction, calcium regulation and excitation‐contraction coupling genes, which are mainly expressed in cardiomyocytes.^11^, ^58^, ^59^ Although cardiomyopathy manifestation depends on the type of gene defect, mutations even in the same gene can lead to different clinical phenotypes.^58^ The combination of different factors such as incomplete penetrance, variable expressions, various clinical manifestations and mutational heterogeneity makes the diagnosis, prognostication and treatment of this disorder a complicated challenge.^60^ GWAS cardiomyopathy studies have detected some associated non‐coding variants.^61^ Defects in the 5′ and 3′ untranslated regions (UTRs) are also thought to be involved in cardiomyopathy pathogenesis.^62^

### 4.1 Non‐coding variants in hypertrophic cardiomyopathy

Hypertrophic cardiomyopathy, characterized by asymmetric hypertrophy in the ventricular wall, is the most prevalent Mendelian cardiomyopathy (≈1:500).^63^, ^64^ Despite variable clinical phenotypes such as breath shortness, palpitations, syncope, chest pain, heart failure and arrhythmias, a significant number of patients with HCM are asymptomatic.^60^, ^65^ HCM is also associated with both sudden cardiac death in young adults, including athletes, and progressive heart failure.^2^, ^66^ Although HCM is inherited in the AD mode, a few cases with AR and de novo mutations have also been reported.^67^ This disorder is denoted as the disease of sarcomeres, and mutations in two main genes (viz, *MYBPC3* and *MYH7*) are responsible for almost 70% of the identified HCM mutations. Other genes involved in HCM pathogenesis, with a frequency of 1%–5%, are *TNNI3*, *TNNT2*, *MYL2*, *TPM1*, *ACTC1* and *MYL3*.^68^ Rarely, defects in muscle LIM protein (*CSRP3*) and α‐actinin 2 (*ACTN2*), which encode proteins vital for sarcomere function and structure, lead to HCM.^69^ Genetic analysis is helpful for 60%–70% of patients with familial HCM and 30% for the sporadic form.^70^, ^71^ In recent years, even with the advent of NGS, no causative mutations in 50% of patients diagnosed with HCM have been identified.^72^, ^73^, ^74^ One explanation for this phenomenon is the possibility of deep intronic mutations, which are not detected by these genetic approaches. One of the pioneering studies on intronic mutations of HCM patients was a cohort by Daniel Jacoby in 2017. The DNA sequences of 16 unrelated patients were analysed by WES. Twenty‐six genes were targeted, and likely candidate causal variants were identified in *VCL*, *PRKAG2* and *TTN*. These variants, with a threefold frequency, disrupted TFs and splicing sites. Analysis with different bioinformatics tools such as Genome‐Wide Annotation of Variants (GWAVA), Combined Annotation‐Dependent Depletion (CADD) and Genomiser revealed that two intronic variants (viz, c.499+367T>C in *VCL* [frequency = 19%] and c.1234‐317T>G in *PRKAG2* [frequency = 3%]) had high frequency rates in the recruited probands by comparison with a healthy population. Eight intronic variants were identified in *TTN*. Among them, c.31484‐286G>T (rs142156368) and c.32077+31C>G (rs72650063) were highly enriched in the cohort. The findings suggested that these intronic variants might contribute to HCM.^75^ Luis R Lopes conducted a large‐scale study on 1644 multiethnic HCM patients for intronic screening by NGS and WES and found alterations in coding regions in 33.2% of the study population. Four intronic variants (viz, c.1224‐52G>A, c.1224‐80G>A, c.1224‐21A>G and c.906‐36G>A) in *MYBPC3*, with an overall frequency of 24/1644 (2.2%), led to a frameshift and a stop codon. Additionally, c.1224‐52G>A was surprisingly frequent in that study (1.1%) in comparison with p.Arg502Trp, which was the most common variant of HCM with a prevalence rate of between 1.4% and 2%. The reason for this high frequency is not clear; nonetheless, this common variant could be the result of proband Asian ethnicity. Also in that study, a novel variant, c.1898‐23A>C, was identified in two mutation‐negative families segregated by phenotype. The reverse transcription‐polymerase chain reaction (RT‐PCR) amplification of the RNA indicated the presence of additional 473 base pairs (bp) containing the intron 19 sequence. This mid‐splice variant caused premature stop codons and mediated mRNA decay, resulting in haploinsufficiency.^76^ The loss of canonical splicing site and the emergence of a new acceptor splice site may be a mechanism through which two *MYBPC3* intronic variants (viz, c.506‐2A>C and c.2308+3G>C) act in HCM pathogenesis. Moreover, c.393‐5C>A, located in *SCN5A*, leads to exon skipping and one small in‐frame deletion in the S1‐D1 transmembrane of the α subunit of the cardiac sodium channel. Interactions between S1–S3 and S4 segments are a vital factor in cardiac function, and this deletion leads to the loss of function of sodium channels.^77^ Sodium channels are composed of several subunits; the functional one, however, is the α subunit. This channel consists of four internal homologous domains containing six transmembrane segments individually.^78^ The sequencing of intronic variants and splice sites can improve our understanding of families with HCM.

In our search, we gathered 470 non‐coding variants associated with HCM. *MYBPC3* constituted 132/470 (28.08%) of the reported variants. Three other genes (viz, *GAA*: 51/470 [10.85%], *AGL*: 48/470 [10.21%] and *ACADVL*: 46/470 [9.79%]) had more variants than the other involved genes (Table S2).

### 4.2 Non‐coding variants in dilated cardiomyopathy

Dilated cardiomyopathy is the most common type of cardiomyopathy and is characterized by a left ventricular ejection fraction of less than 45%, systolic dysfunction and myocardium hypokinesia.^79^, ^80^ This disorder is an indication for heart transplantation, and it is also associated with increased risks of arrhythmia‐related mortality.^81^ The first investigations, between 1974 and 1985, reported a prevalence of 1:2500 for DCM patients, but recent epidemiological studies have estimated a prevalence of 1:250.^82^, ^83^ The clinical manifestations of DCM include sudden cardiac death, heart failure and thromboembolism.^84^ Many factors such as alcohol and cocaine abuse, myocarditis, *Coxsackieviruses*, beriberi, haemochromatosis, Chagas disease, drugs and pregnancy play a significant role in DCM aetiology.^80^, ^84^, ^85^, ^86^, ^87^ In addition, between 15% and 35% of DCM cases are idiopathic, and several genes that affect cytoskeletal proteins, Z‐disks, sarcomeres, desmosomes and extracellular matrices are involved in DCM pathology.^88^, ^89^, ^90^ More than 50 genes associated with DCM have been reported; nevertheless, previous studies have indicated that variants in 17 genes (viz, *MYH7*, *DMD*, *DSP*, *VCL*, *DES*, *LDB3SCN5A*, *ACTC1*, *NEXN*, *TNNT2*, *RBM20*, *PLN*, *LMNA*, *TPM1*, *TNNC1*, *BAG3* and *TTN*) constitute the majority of candidate causal and likely candidate causal alterations in DCM.^88^, ^91^, ^92^, ^93^, ^94^, ^95^ Among all the identified genes, *TTN* accounts for 12%–15% of sporadic DCM cases and 25% of familial DCM cases.^95^, ^96^ The current DCM genetic paradigm has nearly 50% sensitivity in mutation detection. This is due to the heterogeneity and low frequency (3%–5%) of the identified mutations compared with patients suffering from large DCM with no characterized variants.^94^ This fact highlights the role of other parts of the genome, especially the non‐coding region, in DCM pathogenesis. Liang‐Liang Fan et al. utilized WES to study a family affected by DCM, cardiac conduction disease, and transient syncope simultaneously and detected one splice‐site novel variant, c.333+2T>C, in TNNI3 interacting kinase (*TNNI3K*). Segregation analysis was conducted for the other family members, and the result indicated that this variant co‐segregated with the phenotype. Functional analysis and in silico tools categorized this variant as a deleterious one. The *TNNI3K* gene directly regulates cardiac contraction through the phosphorylation of 2 troponin I serine residues.^97^ A recent analysis proved that *TNNI3K* had direct interactions with three DCM‐linked sarcomeric proteins (viz, myosin‐binding protein C, troponin I and cardiac α‐actin); therefore, the loss of function of *TNNI3K* resulted in myocardial structural disorders and arrhythmias.^98^ Non‐coding variants function via various mechanisms such as alterations in chromatin organization and disruptions in both proximal and distal regulatory elements, leading to the manifestation of loss‐of‐function phenotypes.^99^ Nonsense‐mediated mRNA decay is a mechanism whereby gene expression errors are reduced by the elimination of mRNA transcripts. Although this mechanism is effective, it may lead to haploinsufficiency and finally a disorder. Previous investigations have demonstrated that *TNNI3K* has a key role in both sarcomere organization and heart development; hence, decreased *TNNI3K* mRNA levels might lead to DCM and cardiac conduction disease.^98^ Truncating variants in gene encoding titin (TTNtvs) are found in between 13% and 25% of DCM patients. TTNtvs are also found in normal populations, rendering their candidate causality evaluation somewhat challenging. Hoorntje et al. identified c.59926+1G>A, a splice‐site variant, in multiple DCM probands. The finding accounted for the first founder effect in *TTN*, and it was associated with a cardiomyopathy logarithm of the odds (LOD score) of 3.6.^100^ In a study by Ortiz‐Genga, 2877 patients were investigated via NGS. The results revealed 23 different *FLNC* truncating mutations in 20 DCM probands. Among these mutations, six mutations (viz, c.3791‐1G>C, c.7251+1G>A, c.4127+1delG, c.5539+1G>C, c.3791‐1G>A, and c.3965‐2A>T) were located in the non‐coding part of the genome. Previous investigations indicated that *FLNC* splice site variants may lead to haploinsufficiency and mediate candidate causality via this mechanism.^101^ *FLNC* encodes filamin‐C, which is responsible for sarcomere attachment to the plasmatic membrane.^102^ Defects in this gene are associated with myofibrillar myopathies and cardiac involvement. Cardiac myocytes express filamin‐C, which participates in various molecular mechanisms, signal transductions, and interactions between sarcomeres and plasmatic membranes. This protein has direct interactions with two other protein complexes, dystrophin‐associated glycoprotein and integrin complexes, which connect the subsarcolemmal actin cytoskeleton to the extracellular matrix, and any defects in this process may lead to DCM.^103^, ^104^, ^105^ Non‐coding parts are not restricted to splice sites and intronic regions. Mutations in 5′/3′ UTRs through impairment in gene expression may cause a pathological state. Although mutations in the 3′ UTR region of dystrophia myotonica 1 protein kinase (DMPK) and reticulon‐4 are found to be associated with DCM, more studies are required for further elucidation.^62^ Our comprehensive search yielded 476 non‐coding variants, with *DMD* (221/476 [46.43%]), and *TTN* (162/476 [34.03%]) comprising the majority of the reported variants, respectively (Table S3).

Heart failure is a public health problem affecting 1%–2% of the adult population in developed countries.^106^ This condition is a clinical syndrome manifesting itself in all types of cardiomyopathies. The estimation of the incidence of heart failure in DCM patients is challenging owing to a variety of factors that should be considered in patient selection. Indeed, only a few clinical trials and studies have been hitherto conducted on the aetiology of heart failure. In a study by Kubanek et al.,^107^ 32% of the enrolled DCM patients presented with heart failure, and 66% had experienced hospitalization for heart failure once before recruitment. In another large cohort study on 881 DCM patients, the most prevalent clinical manifestation, with a higher incidence rate among women (64% vs. 54%), was heart failure.^108^ The majority of HCM patients present with heart failure with a preserved ejection fraction rather than heart failure with a reduced ejection fraction (<40%). In a cohort consisting of 1000 HCM patients aged between 30 and 59 years, approximately 50% of the study population had heart failure and experienced mild‐to‐severe symptoms.^109^ A large investigation on a European cardiomyopathy registry revealed that the prevalence of heart failure among RCM patients was high (83%). This high rate was inconsistent with another cohort by Ammash et al.,^110^ who reported that 81% of their patients with RCM had overt heart failure. In recent years, GWAS studies have revealed an association between non‐coding variants and advanced heart failure. In a 2‐stage case–control study by Cappola et al., 1590 Caucasian patients affected with heart failure were investigated. The results revealed that 2 novel intronic susceptibility loci in *HSPB7* and *FRMD4B* (viz, rs1739843 and rs6787362, respectively) had a robust association with advanced heart failure. In contrast to rs1739843, which was associated with both ischemic and nonischemic heart failure, rs6787362 was associated only with ischemic heart failure. The results of one prospective meta‐analysis pooling the data of four previously published cohorts of the Heart and Aging Research in Genomic Epidemiology (CHARGE) Consortium indicated that 2 of 14 high‐signal SNPs were located in intronic regions: rs11118620 in *LOC100129376* and rs11880198 in *GNA15*.^111^

### 4.3 Non‐coding variants in other cardiomyopathies

Another type of cardiomyopathy is arrhythmogenic right ventricular dysplasia/cardiomyopathy (ARVD/ARVC), characterized by sudden death, syncope, heart failure, ventricular arrhythmias and palpitations. ARVC affects between 1/1000 and 1/5000 in the general population, and it usually affects young people, especially athletes. The first mutation reported for ARVC was a defect in plakoglobin. Recent advances in genetic testing have revealed that mutations in desmosomal genes constitute more than 50% of the affected patients.^112^, ^113^, ^114^ Most ARVC candidate causal variants are located in five genes: plakoglobin (*JUP*), desmocollin‐2 (*DSC2*), desmoplakin (*DSP*), plakophilin‐2 (*PKP2*) and desmoglein‐2 (*DSG2*).^115^ In addition to desmosomal genes, other genes such as *LMNA A/C*, *CTNNA*, *MEM43*, *TTN*, *PLN* and *DES* have been associated with ARVC.^116^, ^117^, ^118^, ^119^, ^120^, ^121^ Defects in these genes change the normal function and structure of specific desmosomal proteins in the right ventricle. The left ventricle may also be affected over time.^122^ Genetic testing can identify 30%–50% of causative genes in patients with ARVC. This rate is higher (26%–58%) in patients affected by familial forms.^113^, ^123^, ^124^, ^125^ Nearly 10% of candidate causal mutilations are intronic mutations.^126^ Accordingly, the remaining ARVC cases without any identified mutations may be carriers of non‐coding variants. Lorenzon et al. screened 91 ARVC index cases for *DES* mutations and identified 4 non‐coding variants: c.579‐38C>T, c.735+20C>T, c.736‐35C>A, and c.736‐11A>G. Mechanisms affecting subcellular organelles, desmin cytoskeleton and myofibril degradation can explain why these mutations result in desminopathies or myopathies.^127^ In a large retrospective cohort, four likely candidate causal non‐coding variants (viz, c.523+1G>A, c.523+2T>C in *DSG2*, c.2146‐1G>C and c.337‐2A>T) and 1 candidate causal variant (viz, c.2489+1G>A) were reported in *PKP2*. That study was the largest ARVC investigation by NGS to seek causative variants linked to ARVC.^128^ In addition to these intronic sites, a few studies have been conducted on the role of regulatory regions and ARVC. In an original study by Christensen et al., 65 adult patients diagnosed with ARVC were recruited. Genetic analysis indicated 1 rare novel heterozygous variant (‐317G>A) located in the upstream region of *DSG2* in a genetically unexplained patient. Further investigations revealed that DSG2 protein levels in the carriers of ‐317G>A were reduced compared with those in the healthy controls. Luciferase reporter gene assays also indicated that transcriptional activity was diminished in the carriers of the *DSG2*_A minor allele because of the impact on TGF‐β1 and an activator of the protein kinase C pathway. The presence of the *DSG2*_A cAMP response element‐binding protein results in the activation of TF (CREB/ATF) binding and changes in the interactions of c‐JUN.^129^ In our analysis, we identified variants of 10 genes for ARVC, among which 33/107 (30.84%) were for the *PKP2* gene (Table S4).

Restrictive cardiomyopathy is a rare cardiac disorder characterized by increased myocardial stiffness leading to impairment in the ventricular filling.^110^, ^130^ RCM is a poor prognosis disorder, especially when affected patients present symptoms in childhood and require cardiac transplantation.^131^, ^132^ Secondary causes such as infiltrative disorders (e.g. Gaucher disease and Hurler syndrome amyloidosis), storage diseases (e.g. haemochromatosis, Fabry disease and glycogen storage disease) and irradiation are involved in RCM aetiology.^130^, ^133^ The low incidence rate of RCM accounts for our limited knowledge of its genetics compared with HCM and DCM. RCM has a familial inheritance mode, and several sarcomeric and cytoskeletal genes such as *TNNT2*, *FLNC* and *MYPN* are associated with RCM.^133^, ^134^, ^135^ A study by Mogensen et al.^135^ indicated a relationship between abnormalities in cardiac troponin I and RCM. Cardiac troponin is sensitive to intracellular calcium ion (Ca2^+^) levels, and it participates in the regulation of muscle contraction. Cardiac troponin contains three subunits (viz, troponin T, troponin C and troponin I [TNNI3]), which prevent the interaction between actin and myosin in the absence of Ca2^+^. Defects in the troponin complex affect Ca2^+^ affinity, and the interactions between these proteins lead to cardiomyopathy development.^136^ Despite the development of high‐throughput sequencing methods, the rate of successful genotyping in RCM patients is 30%.^133^, ^134^, ^135^ The remaining patients may be carriers of putative variants in other parts of the genome. In our search, we identified 54 non‐coding variants reported for RCM, and the most frequently reported variants among them were in *ALMS1* (26/54 [48.15%]) (Table S5).

## 5 NON‐CODING VARIANTS IN CONGENITAL HEART DISEASES

Congenital heart diseases affect the outflow tract, the septum and the valves, and they are the most common birth defect with a prevalence of 0.8 to 1 child per 100 live births.^137^, ^138^ This group of diseases is classified based on haemodynamic and anatomic lesions into five major subtypes: outflow tract defects,^139^ abnormal left–right relationships, conotruncal defects and impairments affecting the inflow. Thirty percent of patients are affected by severe and lethal forms of congenital heart diseases, and surgical intervention in the first year of life is vital for them.^137^ Progress in surgery has conferred a survival rate of 95%; still, in developing countries, these diseases remain the major cause of child mortality.^137^, ^140^ Congenital heart diseases are genetically heterogeneous, with many patients being affected by the isolated form. The isolated form is a condition in which there is only one heart defect and no additional abnormalities or syndromes are present.^141^ For all the conducted studies, the aetiology and molecular mechanisms of these diseases have yet to be elucidated.^142^ Up to now, more than 50 genes and point mutations associated with congenital heart diseases have been reported. Among them, genes related to cardiac development such as TFs GATA4 and NKX2‐5 constitute a considerable portion. Chromosomal copy number variants (CNVs), signalling pathways related to cardiac morphogenesis (the Notch and Jagged pathways) and chromosomal abnormalities (chromosome 21 trisomy) account for nearly 25% of cases.^143^ In the majority of patients with congenital heart diseases, especially familial forms, no causative variants and single candidate genes have been identified, which highlights the role of de novo mutations and the polygenic inheritance mode in such patients. Research has indicated that 10% of patients with congenital heart diseases are carriers of de novo mutations.^144^ Although recent GWAS studies have underscored the role of the polygenic inheritance of congenital heart diseases, only a few significant associations have been reported. One of the strong associations was reported by Cordell et al. in 2013. Their investigation was a large‐scale GWAS study on 835 cases and 5159 controls. The results indicated that the top SNPs were located in intron 7 of the *GPC5* gene. Glypicans are a group of proteoglycans with six members binding to the outer surface of the plasma membrane through a glycosyl‐phosphatidylinositol anchor. Glypicans are involved in many intracellular pathways, including the Wnt pathway, Hedgehog developmental pathways and morphogenetic pathways; they are, therefore, candidate genes for major processes in heart development.^145^, ^146^ In patients with congenital heart diseases, similar to other unexplained genetic disorders, non‐coding variants may contribute to pathogenesis. In a study by Reamon‐Buettner et al., the 3′‐UTR of *TBX5*, which is a TF expressed in the heart, was sequenced in patients with congenital heart diseases and 10 variants were identified. Among these variants, the prevalence of 1 variant, c.*1101C>T (rs6489956), was considerably different between the case group and the healthy controls. *TBX5* mRNA expression was evaluated using quantitative RT‐PCR conducted on cardiac tissue samples. The results demonstrated that *TBX5* rs6489956 genotypes were correlated with transcription and translation levels.^147^ The 3′‐UTR of mRNA consists of regulatory elements that are vital for accurate gene expression. The direct sequencing of 12 patients revealed nine variants in the patients: c.+77C>T, c.+10T>C, c.+479A>G, c.+462T>C, c.+44T>A, c.+218C>T, c.+259A>G, c.+280T>C, and c.+442A>G. The 3′‐UTR of *GATA4* has conserved motifs that may play a role in post‐transcriptional regulation. The localization of mRNAs and proper configuration both rely on 3′‐UTR, and any defects in this region may lead to a pathological state.^148^ In recent years, NGS technology has been applied to investigate the genetics of both familial and sporadic forms of congenital heart diseases. Haploinsufficiency is another mechanism through which non‐coding variants act. Defects in elastin^149^ are associated with the nonsyndromic forms of supravalvular aortic stenosis. Blue et al. used targeted NGS and identified 1 splice‐site variant: c.950‐3C>G in *ELN*. Supravalvular aortic stenosis is found in 60%–84% of individuals due to *ELN* haploinsufficiency. This variant affects the 3′ acceptor splice‐site region in intron 17 and leads to premature splicing.^150^ In our analysis, we identified 275 variants associated with congenital heart diseases. Our results indicated that two genes (*CREBBP* 51/264 [19.32%] and *KMT2D* 38/264 [14.4%]) were more frequent. For 33 genes, only one non‐coding variant was reported (Table S6).

## 6 NON‐CODING VARIANTS IN THORACIC AORTIC ANEURYSMS AND DISSECTIONS

Thoracic aortic aneurysms constitute a silent and asymptomatic pathological state. They are characterized by an enlarged thoracic aorta, and they affect 1 per 100,000 people in the general population.^151^, ^152^ The detection of thoracic aortic aneurysms is difficult before the occurrence of complications such as dissections and ruptures.^153^ Thoracic aortic aneurysms comprise a multifactorial disorder, and they are associated with many risk factors such as genetic factors (e.g. congenital defects and hypertension) and environmental factors (e.g. smoking and aging).^154^ Conventionally, thoracic aortic aneurysms are categorized into two main forms: syndromic and nonsyndromic. Many syndromes, including Ehlers–Danlos syndrome,^155^ Marfan syndrome and Loeys–Dietz syndrome, are associated with thoracic aortic aneurysms.^156^ Despite surgical intervention, syndromic thoracic aortic aneurysms have a poor prognosis by comparison with the nonsyndromic form.^157^ Nonsyndromic thoracic aortic aneurysms are more prevalent; still, patient detection remains a challenge on account of the fact that some genes are involved in the pathogenesis of the 2 forms.^158^ Thoracic aortic disease is the consequence of a single mutated gene inherited in the AD mode in patients with a positive family history, and it constitutes 20% of cases with thoracic aortic aneurysms. Defects in genes, including *TGFB2*, *TGFBR2*, *TGFBR1* and *SMAD3*, are responsible for 10% of familial nonsyndromic thoracic aortic aneurysms/dissections. Additionally, mutated *ACTA2* accounts for 12%–21% of familial thoracic aortic aneurysms/dissections, and the remaining identified genes represent only 1%–2% of individuals with nonsyndromic thoracic aortic aneurysms/dissections.^159^, ^160^ In recent years, the remaining unexplained cases have been investigated by GWAS studies and genome sequencing technology, leading to the identification of candidate genes and SNPs that were located even in non‐coding regions in association with thoracic aortic aneurysms. Poninska et al. performed WES on 51 unrelated patients with thoracic aortic aneurysms/dissections and found 22 rare variants (six novel variants). One variant, c.6740‐2A>G in *FBN1*, which disrupts a splice‐site acceptor, was found in a 21‐year‐old woman suspected of Marfan syndrome. *FBN1* encodes fibrillin‐1, a glycoprotein that plays a role in maintaining fibre integrity.^161^ Exon skipping is another mechanism whereby some variants affect the normal process. In a previous study, targeted NGS revealed 1 novel splice‐site variant, c.871+1G>A in *SMAD3*, in two patients with nonsyndromic familial thoracic aortic aneurysms/dissections. In that study, aortic tissue was subjected to mRNA extraction, followed by RT‐PCR. Additionally, cDNA amplification on exons 5 to 8 revealed the skipping of exon 6, leading to a 213‐nucleotide deletion. Sequence analysis was then conducted as the confirmation test; the result showed that the shorter fragment did not have the entire exon 6. Afterward, in silico analysis indicated that SMAD3 conformation was essential for the function of this protein and its interaction with other proteins. Smad family proteins are TFs binding to DNA sequences, and any changes and alterations may affect transcription.^162^ Previous investigations have shown that the major portion of the candidate causal variants of *SMAD3* is in the MH2 domain within exon 6^163^ and that acceptor splice‐site variants usually result in proteins with impaired function.^164^ In addition, Aubart et al.^163^ and Regalado et al.^165^ categorized loss‐of‐function variants located in *SMAD3* as candidate causal ones. Moreover, *SMAD3* encodes a protein that plays a role in the cellular TGF‐β signalling pathway, and defects in this gene cause disorganization in the fragmentation of the elastic fibre, the media layer and collagen accumulation, all of which are involved in aortic aneurysm development.^166^, ^167^, ^168^ A functional study by Ying Wang revealed that a variant of *SMAD4* increased the risk of thoracic aortic aneurysms/dissections. Additionally, 202 thoracic aortic aneurysm/dissection cases were genotyped by five tagging SNPs of *SMAD4*, rs12455792, located in the 5′‐UTR of *SMAD4*, which is a binding site for TFs. A significant finding in a prior study indicated that rs12455792 might regulate the pathophysiological mechanisms related to smooth muscle cells such as proteoglycan degradation, apoptosis and accumulated fibre levels.^169^ In our search, 150 variants were associated with thoracic aortic aneurysms. Among them, 107/150 (71.33%) were identified in *FBN1* (Table S7).

## 7 NON‐CODING VARIANTS IN CORONARY ARTERY DISEASES AND STROKES

Coronary artery diseases are inflammatory, atherosclerotic cardiovascular diseases with various clinical manifestations such as sudden cardiac death, myocardial infarction, and both stable and unstable angina. Atherosclerotic coronary artery diseases are accountable for more than 80% of sudden cardiac death cases.^170^ Both genetic and environmental factors are responsible for coronary artery disease aetiology, and the heritability rate of this disorder is estimated to range between 40% and 60%.^171^ Different medications such as statins, aspirin and β‐blockers have been prescribed, conferring a better prognosis in some patients.^172^ GWAS studies have indicated that 9p21.3, containing the *CDKN2A* and *CDKN2B* regulating cell cycle, is associated with coronary artery diseases.^173^, ^174^, ^175^ Mutated genes such as *ABCA1*, *LDLR*, *APOB100*, *ARH*, *PCSK9* and *CYP7A1* in Tangier disease and familial hypercholesterolemia are responsible for premature coronary artery diseases.^176^, ^177^, ^178^, ^179^, ^180^ In addition, 396 SNPs within nine chromosomal regions have been reported to be associated with coronary artery diseases.^181^ This group of diseases has a complex genetic architecture. Indeed, although genes involved in many biological pathways such as vascular tone and remodelling, lipid metabolism and inflammation have been identified, the precise molecular mechanism is still unknown.^182^, ^183^ Coronary artery diseases and myocardial infarction were the first diseases targeted in GWAS studies.^184^ A study by Huang revealed the association between 3′‐UTR mutations of *MEF2A* and coronary artery diseases. Totally, 238 individuals affected with coronary artery diseases were recruited in that study, the results of which showed that the TA haplotype carrier of rs325380 had a meaningful association with coronary artery disease development. Given that UTRs are involved in gene expression and all post‐transcriptional processes, any defects in these areas may affect the normal process and lead to a pathological state.^185^ In our study, we identified 19 variants, of which eight were located in *LIPA* (Table S8).

## 8 NON‐CODING VARIANTS IN OTHER CARDIOVASCULAR DISEASES

Strokes, defined as focal neurological defects, rank second after ischemic heart diseases in terms of mortality among cardiovascular diseases.^186^ Strokes are categorized into two main types: ischemic and haemorrhagic. Ischemic strokes were reported to have an occurrence rate of 85% in a previous investigation.^187^ Many genetic and nongenetic risk factors such as sex, ethnicity, age, smoking, obesity and diabetes play roles in stroke development.^188^ Furthermore, strokes are the consequence of a considerable number of rare single‐gene disorders.^189^ Cerebral AD arteriopathy with subcortical infarcts and leukoencephalopathy (CADASIL) is categorized as the most frequent single‐gene disorder that causes ischemic strokes.^190^ Despite the identification of 160 defects in the *NOTCH3* gene associated with severe cerebral small vessel diseases, the mechanism of strokes remains unclear and challenging. Indeed, for all the investigations thus far performed on the issue, no strong replicable associations have been reported.^189^, ^191^ Several studies have implemented the exome approach to identify rare variants responsible for the development of complex diseases. A GWAS analysis by Söderholm et al. revealed an intronic variant, rs1842681 in *LOC105372028*, leading to the expression of protein phosphatase 1, which is involved in brain plasticity.^192^

As was mentioned above, in recent years, GWAS studies have enhanced our understanding of cardiovascular disease genetics. Another use of GWAS is to identify the genetic architecture of common complex and observational traits related to cardiovascular diseases such as hypertension.^193^ Hypertension is considered the leading cause of morbidity and mortality the world over. It leads to various pathological states such as heart failure, atrial fibrillation and coronary artery diseases.^194^ The renin–angiotensin system is responsible for hypertension development; nonetheless, enzymes and proteins involved in this system have yet to be characterized fully.^195^ A recent GWAS study was conducted on 140,000 patients with European ancestry, and it recruited patients and data from different projects such as the International Consortia, the 1000 Genomes Project and the UK10K imputation. The results demonstrated 24 loci for systolic blood pressure, 41 loci for diastolic blood pressure and 42 loci for pulse pressure.^196^ In a study by Lozano‐Gonzalez et al., the frequencies of four intronic variants (viz, rs2285666, rs2048683, rs2106809 and rs4240157) in *ACE2* were investigated. Regression analysis revealed that among these four SNPs, rs2048683 and rs4240157 had a significant association with systolic and diastolic blood pressures in both sexes.^197^ In another GWAS study on 1621 hypertensive cases, 1 variant, rs13333226, in the 5′ region of Uromodulin (UMOD) was detected.^198^ In our analysis, we gathered 552 variants and found that *PKHD1* (86/552 [15.58%]), *BMPR2* (63/552 [11.41%]) and *VHL*, *LOC107303340* (53/552 [9.6%]) constituted a considerable portion of the reported variants (Table S9).

The definition of sudden cardiac death by the World Health Organization is death within 1 h after symptom manifestation or 24 h after categorization as an asymptomatic patient.^199^, ^200^ Given that out‐of‐hospital sudden cardiac death has a 60% occurrence rate, the precise mechanism involved in sudden cardiac death pathogenesis usually remains challenging.^201^ Myocardial infarction in patients aged between 45 and 50 years or older is the most causative factor in sudden cardiac death.^202^ Inherited disorders such as cardiomyopathies and channelopathies constitute 5%–10% of sudden cardiac death cases.^203^ Sudden cardiac death due to inherited disorders is the consequence of defects in both sarcomere/desmosome proteins, regulatory proteins and ion channels.^204^ Jaouadi et al. conducted WES and detected 1 intronic variant, c.331+1G>A, in *TECRL* with an activating feature. This variant activated an intronic cryptic donor site, leading to a splicing alteration.^205^ In a study by Son et al., on 15 Korean survivors of sudden cardiac arrest, the coding exons of *SCN5A*, *KCNQ1* and *KCNH2* were investigated. The results revealed that 1 intronic variant in *KCNQ1* (viz, rs2283222) had a significant association with sudden cardiac death (odds ratio = 4.05), and four patients had intronic variants in the *SCN5A* gene.

Cardiac arrhythmias are defined as any variations in the rate or rhythm of the normal heart. Abnormal impulse formations and disturbances in conduction are two major reasons responsible for arrhythmias. Long QT syndrome, Brugada syndrome and short QT syndrome are all known as this disease entity.^206^ The identification of genetic components underlying arrhythmias highlights the role of ion channels. Ion channels are protein complexes that are located in the cardiomyocyte sarcolemma, and they play a role in ion flow conduction.^207^ In addition to the abovementioned disorders, atrial fibrillation is the most prevalent type of arrhythmia in that it affects 33 million people worldwide.^208^, ^209^ Environmental and genetic factors are both involved in the pathogenesis of atrial fibrillation.^210^ Defects in genes such as *MYL4*, *NPPA* and *KCNQ1* are responsible for this cardiac condition.^211^ GWAS studies on atrial fibrillation have revealed the role of non‐coding loci. The first GWAS study on atrial fibrillation in 2007 indicated that individuals carrying the non‐coding 4q25 locus near the *PITX2* gene were 60% more susceptible to this abnormal heart rhythm.^212^ In our analysis, we identified 233 variants reported for arrhythmias. Among them, 51/233 (21.88%) were located in *GLA|RPL36A‐HNRNPH2* (Table S10).

## 9 OVERLAP IN LOCI ASSOCIATED WITH DIFFERENT CVDS

Previous studies revealed that there is a considerable association between CVDs, major depressive disorder (MDD),^213^, ^214^, ^215^ severe mental disorders (SMDs), COPD and loneliness. In one study by Fuquan Zhang et al. in 2021, polygenic overlap and genetic correlation between eight different CVD and MDD were investigated. The results indicated that MDD has a considerable genetic correlation with CAD, atrial fibrillation, pulse pressure and heart failure.^213^ Mechanisms underlying vulnerability to CVD in SMDs have not yet identified. People with SMDs struggle with loneliness.^216^ Several genetic variants associated with this disorder have been identified in one recent GWASs. This study highlights the role of shared genetic architecture and polygenic overlap between SMDs and CVD.^215^

## 10 DISCUSSION

The current literature features a few non‐coding candidate likely candidate causal variants associated with Mendelian disorders.^217^, ^218^ The recent emergence of NGS (e.g. WGS and WES) has ushered in considerable advances in clinical genetics; however, 50% of patients remain without a definite diagnosis.^219^ Further, despite the use of NGS in the identification of changes in different regions of the genome such as insertions or deletions (indels), SNV inversions and translocations, CNVs, and structural variants, variants within the non‐coding parts of the genome and their effects have remained poorly understood.^220^, ^221^ Previous publications highlight the role of non‐coding variants contributing to the disease risk. However, they only discussed on one specific disease. This is the first comprehensive review collected evidence from published studies on non‐coding genetic variants associated with various types of cardiovascular diseases.

Functional non‐coding regions such as promoters/enhancers, introns, miRNAs, 5′/3′ UTRs and lncRNAs constitute a significant proportion of the genome harbouring candidate causal variations.^222^, ^223^, ^224^ These regions constitute approximately 85% of the human genome. They are also involved in different mechanisms such as transcription process regulation by promoters that are located 0.5 kb from the transcription start sites and recruit RNA polymerase II, TFs and enhancer elements.^2^, ^225^, ^226^, ^227^, ^228^ Previous GWAS studies have revealed that non‐coding variants associated with disease impose risk by changing and affecting functional DNA elements related to gene expression regulation. In addition, these types of variants have a considerable heritability rate, and they are categorized as an effective determinant of being susceptible to disease.^226^ Approximately 90% of all identified SNPs associated with a specific phenotype by GWAS are located within a non‐coding region.^229^, ^230^, ^231^ Cardiovascular diseases, as the leading cause of global mortality, are complex disorders, in whose pathogenesis genetic components and environment have a role. Most cardiovascular diseases are categorized as polygenic disorders; consequently, non‐coding variants, even those with small effects, play a considerable role in the final risk of disease susceptibility.^232^, ^233^, ^234^ Only a few review articles such as a study conducted by Zhang et al. in 2015 have collected different CNVs, large genomic deletions and non‐coding variants.^17^, ^235^ Genetic investigations of cardiovascular diseases, as complex disorders, have revealed that an affected individual is expected to be a carrier of more than 100 identified risk alleles, including non‐coding variants, which should be considered in disease aetiology.^232^ Previous studies have also indicated that the role of these non‐coding variants is not limited to cardiovascular diseases inasmuch as they have also been reported for other complex disorders. A GWAS study suggested that non‐coding variants were associated with obesity and type II diabetes.^233^, ^234^ The identification of non‐coding variants and underlying molecular mechanisms is challenging, and it is investigated via quantitative trait loci (QTL)‐mapping approaches.^236^ In addition to QTL approaches, technologies based on the genome‐wide detection of CNVs have assisted in identifying large causative genomic CNVs associated with disorders.^237^ For instance, a common deletion, 1q21, associated with thrombocytopenia was detected by genome‐wide CNV technology.^238^ The identification of non‐coding variants is not restricted to genetic diagnosis; these variants can also be therapeutic targets. For instance, variants in *PCSK9*, which has a role in the inhibition of LDL‐C circulation, can be a treatment target.^239^

In conclusion, novel genetic approaches and technologies, data sets, and the results of GWAS studies can be drawn upon to unravel the complex genetic architecture of cardiovascular diseases. The ultimate goal in the identification of non‐coding variants is to provide both a better understanding of the pathophysiological mechanisms involved in cardiovascular diseases and effective treatments.

## AUTHOR CONTRIBUTIONS

**Katayoun Heshmatzad:** Investigation (equal); methodology (equal); software (equal); writing – original draft (equal). **Niloofar Naderi:** Investigation (equal); methodology (equal); software (equal); writing – original draft (equal). **Majid Maleki:** Conceptualization (equal); data curation (equal); resources (equal); validation (equal); visualization (equal). **Shiva Abbasi:** Investigation (equal); methodology (equal); resources (equal); software (equal). **Serwa Ghasemi:** Investigation (equal); methodology (equal); software (equal); visualization (equal). **Nooshin Ashrafi:** Methodology (equal); resources (equal); software (equal). **Amir Farjam Fazelifar:** Investigation (equal); resources (equal); validation (equal); visualization (equal). **Mohammad Mahdavi:** Investigation (equal); methodology (equal); resources (equal); validation (equal); writing – review and editing (equal). **Samira Kalayinia:** Conceptualization (equal); data curation (equal); formal analysis (equal); investigation (equal); methodology (equal); project administration (equal); resources (equal); software (equal); supervision (equal); validation (equal); visualization (equal); writing – original draft (equal); writing – review and editing (equal).

## CONFLICT OF INTEREST STATEMENT

The authors declare that they have no competing interests.

## Supporting information


Tables S1–S10

## Data Availability

All data generated or analysed during this study are included in this published article (and its supplementary information files).
