# Supplementary material for: Role of non‐coding variants in cardiovascular disease
Source: J Cell Mol Med. 2023 May 15;27(12):1621–36. doi: 10.1111/jcmm.17762 (PMC10273088; doi:10.1111/jcmm.17762)
Supplement: Supplementary file 1 — Tables S1–S10 [file JCMM-27-1621-s001.docx]

Table 1. The candidate causal/likely candidate causal non-coding variants in Hypercholesterolemia.

| No. | Gene | Variant | Genomic location (hg19) | Transcript | Classification | dbSNP | Frequency of variants (%) | | | | Ref. |
| --- | --- | --- | --- | --- | --- | --- | --- | --- | --- | --- | --- |
|  |  |  |  |  |  |  | **Iranome** | **1000 Genome** | **genomAD** | **TOPMED** |  |
| 1 | LDLRAP1 | c.89-1G>C | Chr1: 25880412 | NM_015627.2 | Candidate causal | rs755104973 | - | - | 0 | - | 241 |
| 2 | LDLRAP1 | c.459+2T>G | Chr1: 25883760 | NM_015627.2 | Candidate causal | rs1461905374 | - | - | 0 | - | 242 |
| 3 | NPHS2 | c.535-1G>T | Chr1: 179526366 | NM_014625.3 | Candidate causal | rs1291398331 | - | 0 | 0 | 0 | 243 |
| 4 | LDLRAP1 | c.533-1G>A | Chr1: 25889560 | NM_014625.3 | Candidate causal | rs762148512 |  | 0 | - | 0 | 244 |
| 5 | APOB | c.11789-2A>C | Chr2: 21227549 | NM_000384.3 | Candidate causal | rs1135402766 | - | - | - | - | 245 |
| 6 | ALB | c.79+1G>A | Chr4: 74270124 | NM_000477.7 | Candidate causal | rs77408163 | - | 0 | 0 | - | 246 |
| 7 | SLC20A2 | c.935-2A>G | Chr8: 42295097 | NM_001257180.2 | Candidate causal | rs1586025869 | - | - | - | - | - |
| 8 | WT1 | c.1447+5G>A | Chr11: 32413513 | NM_024426.6 | Candidate causal | rs587776576 | - | 0 | - | - | 247 |
| 9 | WT1 | c.1447+4C>T | Chr11: 32413514 | NM_024426.6 | Likely Candidate causal | rs587776577 | - | 0 | - | - | 248 |
| 10 | MYBPC3 | c.3190+1G>A | Chr11: 47355107 | NM_000256.3 | Candidate causal | rs111683277 | - | 0 | 0 | 0 | 249 |
| 11 | SLC7A7 | c.1095+2del | Chr14: 23244651 | NM_001126105.2 | Candidate causal | rs1555320639 | - | - | - | - | - |
| 12 | SLC7A7 | c.895-2_895delinsCCATT | Chr14: 23245145 | NM_001126106.2 | Candidate causal | rs1594944871 | - | - | - | - | 250 |
| 13 | MPO | c.2031-2A>C | Chr17: 56348226 | NM_000250.2 | Candidate causal | rs35897051 | 0 | 0 | 0 | 0 | 251 |
| 14 | LDLR, LDLR-AS1 | c.-229_-90del | Chr19: 11199996 | NM_000527.4 | Uncertain Significance/Candidate causal | rs1555800611 | - | - | - | - | 245 |
| 15 | LDLR, LDLR-AS1 | c.-193_-187delinsTG | Chr19: 11200032 | NM_000527.4 | Uncertain Significance/Candidate causal | rs1555800620 | - | - | - | - | 245 |
| 16 | LDLR, LDLR-AS1 | c.-172G>A | Chr19: 11200053 | NM_000527.4 | Uncertain Significance/Candidate causal | rs1555800629 | - | - | - | - | 245 |
| 17 | LDLR, LDLR-AS1 | c.-151C>G | Chr19: 11200074 | NM_000527.4 | Uncertain Significance/Candidate causal | rs1555800631 | - | - | - | - | 245 |
| 18 | LDLR, LDLR-AS1 | c.-150A>G | Chr19: 11200075 | NM_000527.4 | Uncertain Significance/Candidate causal | rs1555800632 | - | - | - | - | 245 |
| 19 | LDLR, LDLR-AS1 | c.-142C>G | Chr19: 11200083 | NM_000527.4 | Uncertain Significance | rs879254370 | - | 0 | - | 0 | 245 |
| 20 | LDLR, LDLR-AS1 | c.-98C>T | Chr19: 11200127 | NM_000527.4 | Uncertain Significance/Candidate causal | rs1555800648 | - | - | - | - | 245 |
| 21 | LDLR, LDLR-AS1 | c.67+1G>T | Chr19: 11200292 | NM_000527.4 | Candidate causal | rs762417023 | - | - | - | - | 245 |
| 22 | LDLR, LDLR-AS1 | c.67+1G>A | Chr19: 11200292 | NM_000527.4 | Candidate causal | rs762417023 | - | - | - | - | 245 |
| 23 | LDLR, LDLR-AS1 | c.68-1G>C | Chr19: 11210898 | NM_000527.4 | Candidate causal | rs879254397 | 0 | 0 | 0 | - | 245 |
| 24 | LDLR, MIR6886 | c.190+984_1846-1160del | Chr19: 11212005 | NM_000527.4 | Candidate causal | - | - | - | - | - | - |
| 25 | LDLR | c.191-1G>T | Chr19: 11213339 | NM_000527.4 | Candidate causal | rs879254433 | - | - | 0 | - | 245 |
| 26 | LDLR | c.190+2323_190+2324insA | Chr19: 11213345 | NM_001195799.2 | Candidate causal | rs1555802710 | - | - | - | - | 245 |
| 27 | LDLR | c.190+2324_190+2325del | Chr19: 11213344 | NM_001195799.2 | Candidate causal | rs875989889 | - | - | - | - | 245 |
| 28 | LDLR | c.190+2332C>A | Chr19: 11213353 | NM_001195799.2 | Candidate causal | rs879254437 | - | - | - | - | 245 |
| 29 | LDLR | c.190+2342del | Chr19: 11213360 | NM_001195799.2 | Candidate causal | rs879254438 | - | 0 | - | - | 245 |
| 30 | LDLR | c.190+2372del | Chr19: 11213393 | NM_001195799.2 | Candidate causal | rs879254446 | - | - | - | - | 245 |
| 31 | LDLR | c.190+2388G>A | Chr19: 11213409 | NM_001195799.2 | Candidate causal | rs879254452 | - | - | - | - | 245 |
| 32 | LDLR | c.190+2389_190+2390delinsAG | Chr19: 11213410 | NM_001195799.2 | Candidate causal | rs879254453 | - | - | - | - | 245 |
| 33 | LDLR | c.190+2413C>A | Chr19: 11213434 | NM_001195799.2 | Candidate causal | rs139400379 | - | 0 | 0 | 0 | 245 |
| 34 | LDLR | c.190+2424_190+2425del | Chr19: 11213445 | NM_001195799.2 | Candidate causal | rs879254458 | - | - | - | - | 245 |
| 35 | LDLR | c.190+2429del | Chr19: 11213450 | NM_001195799.2 | Candidate causal | rs879254461 | - | - | - | - | 245 |
| 36 | LDLR | c.190+2431del | Chr19: 11213452 | NM_001195799.2 | Candidate causal | rs764797225 | - | - | 0 | - | 245 |
| 37 | LDLR | c.191-2437_191-2434del | Chr19: 11213458 | NM_001195799.2 | Candidate causal | rs879254463 | - | - | - | - | 252 |
| 38 | LDLR | c.313_313+1del | Chr19: 11213462 | NM_000527.4 | Candidate causal | rs875989896 | - | - | - | - | 245 |
| 39 | LDLR | c.313+4_313+16del | Chr19: 11213466 | NM_000527.4 | Uncertain Significance/Candidate causal | rs1555802822 | - | - | - | - | 245 |
| 40 | LDLR | c.314-2A>T | Chr19: 11215894 | NM_000527.4 | Candidate causal | rs879254470 | - | 0 | 0 | 0 | 245 |
| 41 | LDLR | c.314-1G>T | Chr19: 11215895 | NM_000527.4 | Candidate causal | rs879254471 | - | - | - | - | 245 |
| 42 | LDLR | c.314-2168del | Chr19: 11215898 | NM_001195800.2 | Candidate causal | rs875989898 | - | - | - | - | 245 |
| 43 | LDLR | c.314-2161_314-2149del | Chr19: 11215903 | NM_001195800.2 | Candidate causal | rs879254477 | - | - | - | - | 253 |
| 44 | LDLR | c.314-2151_314-2142del | Chr19: 11215916 | NM_001195800.2 | Candidate causal | rs879254479 | - | - | - | - | 245 |
| 45 | LDLR | c.314-2148_314-2133del | Chr19: 11215920 | NM_001195800.2 | Candidate causal | rs879254480 | - | - | - | - | 245 |
| 46 | LDLR | c.314-2133del | Chr19: 11215935 | NM_001195800.2 | Candidate causal | rs879254488 | - | - | - | - | 245 |
| 47 | LDLR | c.314-2132_314-2130del | Chr19: 11215936 | NM_001195800.2 | Candidate causal | rs879254489 | - | - | - | - | 245 |
| 48 | LDLR | c.314-2130_314-2124del | Chr19: 11215936 | NM_001195800.2 | Candidate causal | rs879254490 | - | - | - | - | 245 |
| 49 | LDLR | c.314-2129del | Chr19: 11215937 | NM_001195800.2 | Candidate causal | rs879254491 | - | - | - | - | 245 |
| 50 | LDLR | c.314-2117_314-2093del | Chr19: 11215950 | NM_001195800.2 | Candidate causal | rs879254497 | - | - | - | - | 245 |
| 51 | LDLR | c.314-2121TC[2] | Chr19: 11215947 | NM_001195800.2 | Candidate causal | rs879254496 | - | - | - | - | 245 |
| 52 | LDLR | c.314-2115_314-2114insC | Chr19: 11215954 | NM_001195800.2 | Candidate causal | rs879254499 | - | - | - | - | 245 |
| 53 | LDLR | c.314-2107del | Chr19: 11215961 | NM_001195800.2 | Candidate causal | rs878854028 | - | - | - | - | 245 |
| 54 | LDLR | c.314-2104_314-2101del | Chr19: 11215964 | NM_001195800.2 | Candidate causal | rs879254504 | - | - | - | - | 245 |
| 55 | LDLR | c.314-2097C>G | Chr19: 11215971 | NM_001195800.2 | Candidate causal | rs879254509 | - | - | - | - | 245 |
| 56 | LDLR | c.314-2062_314-2056del | Chr19: 11216003 | NM_001195800.2 | Candidate causal | rs879254521 | - | - | - | - | 245 |
| 57 | LDLR | c.314-2044_314-1982delinsA | Chr19: 11216024 | NM_001195800.2 | Candidate causal | rs1555803259 | - | - | - | - | 245 |
| 58 | LDLR | c.314-2014del | Chr19: 11216054 | NM_001195800.2 | Candidate causal | rs879254538 | - | - | - | - | 245 |
| 59 | LDLR | c.314-1999C>T | Chr19: 11216069 | NM_001195800.2 | Candidate causal | rs1135402768 | - | - | - | - | 245 |
| 60 | LDLR | c.314-1978_314-1977insC | Chr19: 11216091 | NM_001195800.2 | Candidate causal | rs879254551 | - | - | - | - | 245 |
| 61 | LDLR | c.314-1973del | Chr19: 11216092 | NM_001195800.2 | Candidate causal | rs879254552 | - | - | - | - | 245 |
| 62 | LDLR | c.314-1968G>C | Chr19: 11216100 | NM_001195800.2 | Candidate causal | rs879254560 | - | - | - | - | 254 |
| 63 | LDLR | c.314-1954_314-1953insT | Chr19: 11216115 | NM_001195800.2 | Candidate causal | rs879254564 | - | - | - | - | 245 |
| 64 | LDLR | c.314-1942C>T | Chr19: 11216126 | NM_001195800.2 | Candidate causal | rs879254570 | - | 0 | 0 | - | 245 |
| 65 | LDLR | c.314-1929del | Chr19: 11216136 | NM_001195800.2 | Candidate causal | rs879254573 | - | - | - | - | 245 |
| 66 | LDLR | c.314-1928_314-1927insG | Chr19: 11216141 | NM_001195800.2 | Candidate causal | rs879254574 | - | - | - | - | 245 |
| 67 | LDLR | c.314-1925_314-1924del | Chr19: 11216142 | NM_001195800.2 | Candidate causal | rs879254575 | - | - | - | - | 245 |
| 68 | LDLR | c.314-1924del | Chr19: 11216142 | NM_001195800.2 | Candidate causal | rs879254575 | - | - | - | - | 245 |
| 69 | LDLR | c.314-1922C>A | Chr19: 11216146 | NM_001195800.2 | Candidate causal | rs121908034 | - | 0 | 0 | 0 | 245 |
| 70 | LDLR | c.314-1921_314-1918delinsTG | Chr19: 11216147 | NM_001195800.2 | Candidate causal | rs1131692195 | - | - | - | - | 245 |
| 71 | LDLR | c.314-1918_314-1896del | Chr19: 11216148 | NM_001195800.2 | Candidate causal | rs879254577 | - | - | - | - | 245 |
| 72 | LDLR | c.314-1908del | Chr19: 11216160 | NM_001195800.2 | Candidate causal | rs879254580 | - | - | - | - | 245 |
| 73 | LDLR | c.314-1908_314-1902del | Chr19: 11216159 | NM_001195800.2 | Candidate causal | rs879254579 | - | - | - | - | 245 |
| 74 | LDLR | c.314-1905_314-1904insA | Chr19: 11216164 | NM_001195800.2 | Candidate causal | rs879254581 | - | - | - | - | 245 |
| 75 | LDLR | c.314-1903_314-1897del | Chr19: 11216164 | NM_001195800.2 | Candidate causal | rs879254582 | - | - | - | - | 245 |
| 76 | LDLR | c.314-1895C>A | Chr19: 11216173 | NM_001195800.2 | Candidate causal | rs879254584 | - | - | - | - | 245 |
| 77 | LDLR | c.314-1893C>A | Chr19: 11216175 | NM_001195800.2 | Candidate causal | rs748672083 | - | 0 | 0 | - | 245 |
| 78 | LDLR | c.314-1886del | Chr19: 11216182 | NM_001195800.2 | Candidate causal | rs879254588 | - | - | - | - | 245 |
| 79 | LDLR | c.314-1863_314-1842del | Chr19: 11216200 | NM_001195800.2 | Candidate causal | rs879254598 | - | - | - | - | - |
| 80 | LDLR | c.314-1844G>A | Chr19: 11216224 | NM_001195800.2 | Candidate causal | rs879254606 | - | - | - | - | 245 |
| 81 | LDLR | c.314-1838_314-1837del | Chr19: 11216228 | NM_001195800.2 | Candidate causal | rs879254612 | - | - | - | - | 245 |
| 82 | LDLR | c.314-1837_314-1836insT | Chr19: 11216232 | NM_001195800.2 | Candidate causal | rs879254614 | - | - | - | - | 245 |
| 83 | LDLR | c.314-1835_314-1799del | Chr19: 11216232 | NM_001195800.2 | Candidate causal | rs879254615 | - | - | - | - | 245 |
| 84 | LDLR | c.314-1826del | Chr19: 11216239 | NM_001195800.2 | Candidate causal | rs875989905 | - | - | - | - | 245 |
| 85 | LDLR | c.314-1825_314-1809del | Chr19: 11216242 | NM_001195800.2 | Candidate causal | rs879254619 | - | - | - | - | 245 |
| 86 | LDLR | c.314-1822_314-1805delinsCCGACTG | Chr19: 11216246 | NM_001195800.2 | Candidate causal | rs879254622 | - | - | - | - | 245 |
| 87 | LDLR | c.314-1817_314-1803delinsAACTGCGGTAAACTGCGGTAAACT | Chr19: 11216251 | NM_001195800.2 | Candidate causal | rs1131692198 | - | - | - | - | 245 |
| 88 | LDLR | c.314-1815_314-1811del | Chr19: 11216253 | NM_001195800.2 | Candidate causal | rs1555803455 | - | - | - | - | 245 |
| 89 | LDLR | c.314-1813_314-1804delinsTGCAA | Chr19: 11216255 | NM_001195800.2 | Candidate causal | rs879254631 | - | - | - | - | 245 |
| 90 | LDLR | c.314-1811del | Chr19: 11216255 | NM_001195800.2 | Candidate causal | rs879254633 | - | - | - | - | 255 |
| 91 | LDLR | c.314-1806_314-1804delinsCA | Chr19: 11216262 | NM_001195800.2 | Candidate causal | rs879254637 | - | - | - | - | 245 |
| 92 | LDLR | c.314-1803del | Chr19: 11216265 | NM_001195800.2 | Candidate causal | rs1555803481 | - | - | - | - | 245 |
| 93 | LDLR | c.314-1803_314-1792del | Chr19: 11216265 | NM_001195800.2 | Candidate causal | rs1064792905 | - | - | - | - | 245 |
| 94 | LDLR | c.314-1802G>T | Chr19: 11216266 | NM_001195800.2 | Candidate causal | - | - | - | - | - | 254 |
| 95 | LDLR | c.693_694+20del | Chr19: 11216273 | NM_001195800.2 | Candidate causal | rs879254645 | - | - | - | - | 245 |
| 96 | LDLR | c.314-1792_314-827del | Chr19: 11216276 | NM_001195800.2 | Uncertain Significance/Candidate causal | - | - | - | - | - | 245 |
| 97 | LDLR | c.694+1G>C | Chr19: 11216277 | NM_000527.4 | Candidate causal | rs879254646 | - | - | - | - | 245 |
| 98 | LDLR | c.695-356_940+1059del | Chr19: 11216885 | NM_000527.4 | Candidate causal | - | - | - | - | - | - |
| 99 | LDLR, MIR6886 | c.695-67_1586+371del | Chr19: 11217174 | NM_000527.4 | Candidate causal | - | - | - | - | - | - |
| 100 | LDLR | c.314-785del | Chr19: 11217282 | NM_001195800.2 | Candidate causal | rs879254660 | - | - | - | - | 245 |
| 101 | LDLR | c.314-778C>A | Chr19: 11217290 | NM_001195800.2 | Candidate causal | rs879254664 | - | 0 | - | - | 245 |
| 102 | LDLR | c.314-746_314-745del | Chr19: 11217320 | NM_001195800.2 | Candidate causal | rs879254673 | - | - | - | - | 245 |
| 103 | LDLR | c.314-732_314-729del | Chr19: 11217336 | NM_001195800.2 | Candidate causal | rs879254675 | - | - | - | - | 245 |
| 104 | LDLR | c.314-726_314-723del | Chr19: 11217342 | NM_001195800.2 | Candidate causal | rs879254677 | - | - | - | - | 245 |
| 105 | LDLR | c.314-708_314del | Chr19: 11217360 | NM_001195800.2 | Candidate causal | rs1555803722 | - | - | - | - | 245 |
| 106 | LDLR | c.314-708_314-705del | Chr19: 11217360 | NM_001195800.2 | Candidate causal | rs879254684 | - | - | - | - | 245 |
| 107 | LDLR | c.817+1G>T | Chr19: 11217364 | NM_000527.4 | Candidate causal | rs879254685 | - | - | 0 | - | 245 |
| 108 | LDLR | c.817+2T>G | Chr19: 11217365 | NM_000527.4 | Candidate causal | rs879254686 | - | - | - | - | 245 |
| 109 | LDLR | c.818-2A>G | Chr19: 11218066 | NM_000527.4 | Candidate causal | rs879254687 | - | - | - | - | 256 |
| 110 | LDLR | c.939_940+3del | Chr19: 11218186 | NM_000527.4 | Candidate causal | rs879254727 | - | - | - | - | 245 |
| 111 | LDLR | c.940_940+14del | Chr19: 11218186 | NM_000527.4 | Candidate causal | rs1057519665 | - | - | - | - | 245 |
| 112 | LDLR | c.940+1G>T | Chr19: 11218191 | NM_000527.4 | Candidate causal | rs879254729 | - | - | - | - | 245 |
| 113 | LDLR | c.940+2T>G | Chr19: 11218192 | NM_000527.4 | Candidate causal | rs875989912 | - | - | - | - | 245 |
| 114 | LDLR | c.1060+1G>A | Chr19: 11221448 | NM_000527.4 | Candidate causal | rs879254771 | - | - | - | - | 245 |
| 115 | LDLR | c.1186+1del | Chr19: 11222314 | NM_000527.4 | Candidate causal | rs879254819 | - | - | - | - | 245 |
| 116 | LDLR, MIR6886 | c.1186+700_2141-545del | Chr19: 11223015 | NM_000527.4 | Candidate causal | - | - | - | - | - | - |
| 117 | LDLR, MIR6886 | c.1187-169_2312-790del | Chr19: 11223785 | NM_000527.4 | Candidate causal | - | - | - | - | - | - |
| 118 | LDLR | c.1187del | Chr19: 11223954 | NM_000527.4 | Candidate causal | rs1057519667 | - | - | - | - | - |
| 119 | LDLR | c.1358+5G>T | Chr19: 11224130 | NM_000527.4 | Uncertain Significance/Candidate causal | rs1060499924 | - | - | - | - | 245 |
| 120 | LDLR, MIR6886 | c.1359-25A>G | Chr19: 11224186 | NM_000527.4 | Uncertain Significance/Candidate causal | rs1131692206 | - | - | - | - | 245 |
| 121 | LDLR | c.1573_1586+5del | Chr19: 11224425 | NM_000527.4 | Candidate causal | rs1135402776 | - | - | - | - | 245 |
| 122 | LDLR | c.1587-5_1618del | Chr19: 11226765 | NM_000527.4 | Candidate causal | rs1555805930 | - | - | - | - | 245 |
| 123 | LDLR | c.1705+1del | Chr19: 11226888 | NM_000527.4 | Candidate causal | rs879254992 | - | - | - | - | 245 |
| 124 | LDLR | c.1705+2T>C | Chr19: 11226890 | NM_000527.4 | Candidate causal | rs1555806110 | - | - | - | - | 245 |
| 125 | LDLR | c.1706-2A>T | Chr19: 11227533 | NM_000527.4 | Candidate causal | rs878854027 | - | - | - | - | 245 |
| 126 | LDLR | c.1706-1G>C | Chr19: 11227534 | NM_000527.4 | Candidate causal | rs879254996 | - | - | 0 | - | - |
| 127 | LDLR | c.1842_1845+2del | Chr19: 11227671 | NM_000527.4 | Candidate causal | rs1555806582 | - | - | - | - | 245 |
| 128 | LDLR | c.1845+1G>C | Chr19: 11227675 | NM_000527.4 | Candidate causal | rs879255049 | - | - | - | - | 245 |
| 129 | LDLR | c.1845+2T>A | Chr19: 11227676 | NM_000527.4 | Candidate causal | rs778408161 | - | 0 | 0 | - | 245 |
| 130 | LDLR | c.1846-2179_2140+2154del | Chr19: 11228589 | NM_000527.4 | Candidate causal | - | - | - | - | - | - |
| 131 | LDLR | c.1975_1987+16del | Chr19: 11230896 | NM_000527.4 | Candidate causal | rs879255093 | - | - | - | - | 257 |
| 132 | LDLR | c.1987+1del | Chr19: 11230909 | NM_000527.4 | Candidate causal | rs879255097 | - | - | - | - | 245 |
| 133 | LDLR | c.1987+2T>A | Chr19: 11230911 | NM_000527.4 | Candidate causal | rs1600742966 | - | - | - | - | 258 |
| 134 | LDLR | c.1988-52_2006del | Chr19: 11230994 | NM_000527.4 | Candidate causal | rs1555807306 | - | - | - | - | 245 |
| 135 | LDLR | c.1988-2A>G | Chr19: 11231044 | NM_000527.4 | Candidate causal | rs879255101 | - | - | - | - | 245 |
| 136 | LDLR | c.1988-1G>C, A | Chr19: 11231045 | NM_000527.4 | Candidate causal | rs1555807335 | - | - | - | - | 245 |
| 137 | LDLR | c.1606+147del | Chr19: 11231055 | NM_001195803.2 | Candidate causal | rs879255106 | - | - | - | - | 245 |
| 138 | LDLR | c.1606+150_1606+151del | Chr19: 11231056 | NM_001195803.2 | Candidate causal | rs1600743301 | - | - | - | - | - |
| 139 | LDLR | c.1606+149del | Chr19: 11231058 | NM_001195803.2 | Candidate causal | rs879255107 | - | - | - | - | 259 |
| 140 | LDLR | c.1606+150del | Chr19: 11231059 | NM_001195803.2 | Candidate causal | rs1060500988 | - | - | - | - | 245 |
| 141 | LDLR | c.1606+150T>A | Chr19: 11231059 | NM_001195803.2 | Candidate causal | rs879255109 | - | - | - | - | 245 |
| 142 | LDLR | c.1606+150_1606+156del | Chr19: 11231058 | NM_001195803.2 | Candidate causal | rs879255108 | - | - | - | - | 245 |
| 143 | LDLR | c.1606+160del | Chr19: 11231069 | NM_001195803.2 | Candidate causal | rs1555807356 | - | - | - | - | 245 |
| 144 | LDLR | c.1606+162_1606+163del | Chr19: 11231070 | NM_001195803.2 | Candidate causal | rs879255110 | - | - | - | - | 245 |
| 145 | LDLR | c.1606+163del | Chr19: 11231073 | NM_001195803.2 | Candidate causal | rs879255110 | - | - | - | - | 245 |
| 146 | LDLR | c.1606+164del | Chr19: 11231073 | NM_001195803.2 | Candidate causal | rs879255112 | - | - | - | - | 245 |
| 147 | LDLR | c.1606+176del | Chr19: 11231084 | NM_001195803.2 | Candidate causal | rs875989937 | - | - | - | - | 245 |
| 148 | LDLR | c.1606+195_1606+198del | Chr19: 11231101 | NM_001195803.2 | Candidate causal | rs879255120 | - | - | - | - | 245 |
| 149 | LDLR | c.1606+199_1606+212delinsC | Chr19: 11231108 | NM_001195803.2 | Candidate causal | rs1555807390 | - | - | - | - | 245 |
| 150 | LDLR | c.1606+203del | Chr19: 11231109 | NM_001195803.2 | Candidate causal | rs879255122 | - | - | - | - | 245 |
| 151 | LDLR | c.1606+204del | Chr19: 11231113 | NM_001195803.2 | Candidate causal | rs879255124 | - | - | - | - | 245 |
| 152 | LDLR | c.1606+204_1606+216del | Chr19: 11231109 | NM_001195803.2 | Candidate causal | rs879255123 | - | - | - | - | 245 |
| 153 | LDLR | c.1606+205_1606+217del | Chr19: 11231114 | NM_001195803.2 | Candidate causal | rs879255125 | - | - | - | - | 245 |
| 154 | LDLR | c.1606+237C>A | Chr19: 11231146 | NM_001195803.2 | Candidate causal | rs112954220 | - | 0 | 0 | 0 | 245 |
| 155 | LDLR | c.1606+241del | Chr19: 11231150 | NM_001195803.2 | Candidate causal | rs879255135 | - | - | - | - | 245 |
| 156 | LDLR | c.1606+245del | Chr19: 11231152 | NM_001195803.2 | Candidate causal | rs879255138 | - | - | - | - | 257 |
| 157 | LDLR | c.1606+282C>A | Chr19: 11231191 | NM_001195803.2 | Candidate causal | rs879255146 | - | - | - | - | 245 |
| 158 | LDLR | c.1606+287del | Chr19: 11231196 | NM_001195803.2 | Candidate causal | rs1257344396 | - | 0 | 0 | 0 | 254 |
| 159 | LDLR | c.2140+1_2140+12del | Chr19: 11231199 | NM_000527.4 | Candidate causal | rs1555807475 | - | - | - | - | 245 |
| 160 | LDLR | c.2141-2A>G | Chr19: 11233848 | NM_000527.4 | Candidate causal | rs1135402780 | - | - | - | - | 245 |
| 161 | LDLR | c.2311+1G>A | Chr19: 11234021 | NM_000527.4 | Candidate causal | rs879255175 | - | - | - | - | 245 |
| 162 | LDLR | c.2312-3C>T | Chr19: 11238681 | NM_000527.4 | Uncertain Significance | rs875989942 | - | 0 | 0 | 0 | 245 |
| 163 | LDLR | c.2390-2A>G | Chr19: 11240187 | NM_000527.4 | Candidate causal | rs767790696 | - | 0 | 0 | - | 245 |
| 164 | LDLR | c.2390-1G>A | Chr19: 11240188 | NM_000527.4 | Candidate causal | rs879255193 | - | - | - | - | 245 |
| 165 | LDLR | c.2547+1G>A | Chr19: 11240347 | NM_000527.4 | Candidate causal | rs879255224 | - | - | - | - | 245 |
| 166 | LDLR | c.2547+5G>A | Chr19: 11240351 | NM_000527.4 | Uncertain Significance | rs879255226 | - | - | - | - | 245 |
| 167 | LDLR | c.2548-2del | Chr19: 11241961 | NM_001195803.2 | Candidate causal | rs879255229 | - | - | - | - | 245 |
| 168 | APOE | c.237-2A>G | Chr19: 45411788 | NM_000041.4 | Candidate causal | rs397514253 | - | 0 | - | - | 260 |
| 169 | PHKA2 | c.717+1G>T | ChrX: 18961827 | NM_000292.3 | Candidate causal | rs587776731 | - | 0 | - | - | 261 |
| 170 | WDR45 | c.827+1G>A | ChrX: 48933022 | NM_001029896.2 | Candidate causal | rs1557083958 | - | - | - | - | 262 |
| 171 | ABCG8 | c.965-1G>A | Chr2: 44099114 | NM_022437.3 | Candidate causal | rs957176669 | - | - | 0 | - |  |
| 172 | APOB | c.2604+1G>A | Chr2: 21246396 | NM_000384.3 | Candidate causal | rs775345377 | - | 0 | 0 | 0 | 263 |
| 173 | APOB | c.11789-1G>C | Chr2: 21227548 | NM_000384.3 | Candidate causal | rs1558560212 | - | - | - | - | 264 |
| 174 | BBS1 | c.-3_37del | Chr11: 66278117-66278156 | NM_024649.5 | Candidate causal | rs113994178 | - | - | - | - | 265 |
| 175 | BBS1 | c.480-2A>C | Chr11: 66283162 | NM_024649.5 | Candidate causal | - | - | - | - | - | 244 |
| 176 | BBS1 | c.723+1G>C | Chr11: 66287220 | NM_024649.5 | Candidate causal | [rs1295318869](https://varsome.com/variant/hg19/rs1295318869?&annotation-mode=germline) | - | - | 0 | - | 244 |
| 177 | BBS1 | c.432+1G>A | Chr11: 66282150 | NM_024649.5 | Candidate causal | rs587777829 | - | 0 | - | 0 | 266 |
| 178 | BBS1 | c.1340-2A>G | Chr11: 66297288 | NM_024649.5 | Candidate causal | rs113994180 | - | 0 | - | - | 267 |
| 179 | BBS1 | c.1111-2A>G | Chr11: 66293592 | NM_024649.5 | Candidate causal | rs761969357 | - | 0 | 0 | 0 | 244 |
| 180 | BBS1 | c.831-2A>G | Chr11: 66290925 | NM_024649.5 | Candidate causal | rs1057517332 | - | 0 | 0 | - | 244 |
| 181 | BBS1 | c.1110+3G>C | Chr11: 66291356 | NM_024649.5 | Uncertain Significance/Candidate causal | rs762276925 | - | 0 | - | 0 | 268 |
| 182 | BBS1 | c.1473+4A>G | Chr11: 66297427 | NM_024649.5 | Uncertain Significance/Candidate causal | rs1486200900 | - | 0 | 0 | - | 269 |
| 183 | BBS1 | c.951+1G>A | Chr11: 66291048 | NM_024649.5 | Candidate causal | rs746875134 | - | 0 | 0 | - | 270 |
| 184 | BBS2 | c.805-1G>C | Chr16: 56536369 | NM_031885.4 | Candidate causal | - | - | - | - | - | 244 |
| 185 | BBS2 | c.1398-2A>G | Chr16: 56533821 | NM_031885.4 | Candidate causal | rs767609303 | - | - | 0 | - | 244 |
| 186 | BBS2 | c.804+1_804+14del | Chr16: 56539848-56539861 | NM_031885.4 | Candidate causal | rs1202682571 | - | 0 | 0 | 0 | 244 |
| 187 | BBS2 | c.535-2A>C | Chr16: 56539950 | NM_031885.4 | Candidate causal | - | - | - | - | - | 244 |
| 188 | BBS2 | c.941-2A>C | Chr16: 56536370 | NM_031885.4 | Candidate causal | rs878962682 | - | - | 0 | - | 244 |
| 189 | BBS2 | c.534+1G>T | Chr16: 56544770 | NM_031885.4 | Candidate causal | rs773862084 | - | 0 | 0 | 0 | 244 |
| 190 | BBS4 | c.76+1G>T | Chr15: 72987570 | NM_033028.5 | Candidate causal | rs1465437164 | - | 0 | 0 | - | 271 |
| 191 | BBS4 | c.406-2A>C | Chr15: 73015133 | NM_033028.5 | Candidate causal | rs113994191 | - | 0 | - | - | 266 |
| 192 | BBS4 | c.333-1G>C | Chr15: 73009118 | NM_033028.5 | Candidate causal | - | - | - | - | - | 244 |
| 193 | BBS4 | c.1248+1G>T | Chr15:73028308 | NM_033028.5 | Candidate causal | - | - | - | - | - | 244 |
| 194 | BBS4 | c.157-3C>G | Chr15: 73004582 | NM_033028.5 | Uncertain Significance/Candidate causal | rs1567412639 | - | 0 | - | - | 271 |
| 195 | BBS4 | c.220+1G>C | Chr15: 73004649 | NM_033028.5 | Candidate causal | rs113994190 | - | 0 | 0 | 0 | 266 |
| 196 | BBS4 | c.77-216del | Chr15: 73001821 | NM_033028.5 | Uncertain Significance/Candidate causal | rs113994189 | - | - | - | - | 266 |
| 197 | BBS4 | c.1106+2T>A | Chr15: 73027525 | NM_033028.5 | Candidate causal | rs886041464 | - | - | - | - | 272 |
| 198 | BBS4 | c.157-2A>G | Chr15: 73004583 | NM_033028.5 | Candidate causal | rs113994192 | - | 0 | 0 | - | 273 |
| 199 | BBS4 | c.712-1G>A | Chr15: 73023645 | NM_033028.5 | Candidate causal | rs377031435 | - | 0 | 0 | 0 | 274 |
| 200 | BBS5 | c.143-1G>A | Chr2: 170343578 | NM_152384.3 | Candidate causal | - | - | - | - | - | 244 |
| 201 | BBS5 | c.817-1G>A | Chr2: 170359604 | NM_152384.3 | Candidate causal | rs1466289570 | - | 0 | 0 | 0 | 244 |
| 202 | BBS5 | c.143-1G>C | Chr2: 170343578 | NM_152384.3 | Candidate causal | rs1054138918 | - | 0 | 0 | 0 | 244 |
| 203 | BBS5 | c.619-1G>C | Chr2: 170354136 | NM_152384.3 | Candidate causal | rs753234582 | - | 0 | 0 | 0 | 275 |
| 204 | BBS7 | c.529-1dup | Chr4: 122776714-122776715 | NM_176824.3 | Candidate causal | - | - | - | - | - | - |
| 205 | BBS7 | c.1677-490_1804del | Chr4: 122749643-122750373 | NM_176824.3 | Candidate causal | rs1578522416 | - | - | - | - | 276 |
| 206 | BBS7 | c.1371+1G>A | Chr4: 122760785 | NM_176824.3 | Candidate causal | rs1578537379 | - | - | - | - | 244 |
| 207 | BBS7 | c.1786+1G>T | Chr4: 122749773 | NM_176824.3 | Candidate causal | rs1560638613 | - | - | - | - | 244 |
| 208 | BBS9 | c.263+4A>G | Chr7: 33192467 | NM_198428.3 | Uncertain Significance | rs370916293 | - | 0 | 0 | - | 271 |
| 209 | BBS9 | c.113-2A>G | Chr7: 33192311 | NM_198428.3 | Candidate causal | rs1562686929 | - | - | - | - | 271 |
| 210 | BBS9 | c.2115+1G>A | Chr7: 33427757 | NM_198428.3 | Candidate causal | rs886039801 | - | - | - | - | - |
| 211 | BBS9 | c.1552+2T>A | Chr7: 33392487 | NM_198428.3 | Candidate causal | rs1563049863 | - | 0 | - | - | 244 |
| 212 | BBS9 | c.1789+1G>T | Chr7: 33407475 | NM_198428.3 | Candidate causal | rs201938124 | - | 0 | 0 | - | 244 |
| 213 | BBS9 | c.263+1G>A | Chr7: 33192464 | NM_198428.3 | Candidate causal | rs137962929 | - | 0 | 0 | 0 | 277 |
| 214 | EHMT1 | c.2193-1G>C | Chr9: 140674086 | NM_024757.5 | Candidate causal | rs137852720 | - | - | - | - | 278 |
| 215 | IFT27|LOC105373021 | c.352+1G>T | Chr22: 37159962 | NM_001177701.3 | Uncertain Significance/Candidate causal | rs780659194 | - | 0 | 0 | - | 279 |
| 216 | LDLR | c.1186+2T>G | Chr19: 11222317 | NM_000527.4 | Candidate causal | rs779921498 | - | 0 | 0 | 0 | 280 |
| 217 | LDLR | c.1891_2311+1065del | Chr19: 11230813-11235085 | NM_000527.5 | Candidate causal | - | - | - | - | - | 244 |
| 218 | LDLR | c.2370_2389+20del | Chr19: 11238742 - 11238781 | NM_000527.4 | Candidate causal | rs1600762098 | - | - | - | - | 244 |
| 219 | LDLR | c.1987+2T>G | Chr19: 11230911 | NM_000527.4 | Candidate causal | rs1600742966 | - | - | - | - | 244 |
| 220 | LDLR | c.940+3_940+6del | Chr19: 11218191-11218194 | NM_000527.5 | Uncertain Significance/Candidate causal | - | - | - | - | - | 281 |
| 221 | LDLR | c.1056_1060+3del | Chr19: 11221443-11221450 | NM_000527.5 | Candidate causal | rs879254770 | - | 0 | 0 | 0 | 282 |
| 222 | LDLR | c.1587-1G>A | Chr19: 11226769 | NM_000527.5 | Candidate causal | rs879254948 | - | - | - | - | 283 |
| 223 | LDLR | c.190+2358del | Chr19: 11213375 | NM_001195799.2 | Candidate causal | rs879254440 | - | - | - | - | 283 |
| 224 | LDLR | c.313+1G>C | Chr19: 11213463 | NM_000527.5 | Candidate causal | rs112029328 | - | - | - | 0 | 284 |
| 225 | LDLR | c.1706-1G>A | Chr19: 11227534 | NM_000527.5 | Candidate causal | rs879254996 | - | - | - | - | 285 |
| 226 | LDLR | c.941-1G>A | Chr19: 11221327 | NM_000527.5 | Candidate causal | rs879254735 | - | - | - | - | 286 |
| 227 | LDLR | c.313+5G>T | Chr19: 11213467 | NM_000527.5 | Uncertain Significance | rs879254467 | - | - | - | - | 287 |
| 228 | LDLR | c.191-732_740del | Chr19: 11212605-11217283 | NM_000527.5 | Candidate causal | - | - | - | - | - | 244 |
| 229 | LDLR | c.817+9T>C | Chr19: 11213467 | NM_000527.4 | Uncertain Significance | rs879254467 | - | - | - | - | 244 |
| 230 | LDLR | c.1706-1G>T | Chr19: 11227534 | NM_000527.5 | Candidate causal | rs879254996 | - | - | 0 | - | 288 |
| 231 | LDLR | c.314-2146_314-2142del | Chr19: 11215921-11215925 | NM_001195800.2 | Candidate causal | rs1057516132 | - | - | - | - |  |
| 232 | LDLR | c.191-1G>A | Chr19: 11213339 | NM_000527.5 | Candidate causal | rs879254433 | - | - | 0 | - | 289 |
| 233 | LDLR | c.1705+1G>A | Chr19: 11226889 | NM_000527.5 | Candidate causal | rs875989926 | - | - | - | - | 290 |
| 234 | LDLR | c.68-1G>A | Chr19: 11210898 | NM_000527.4 | Candidate causal | rs879254397 | 0 | 0 | 0 | - | 291 |
| 235 | LDLR | c.695-1G>T | Chr19: 11217240 | NM_000527.5 | Candidate causal | rs879254652 | - | - | - | - | 292 |
| 236 | LDLR | c.694+1G>A | Chr19: 11216277 | NM_000527.5 | Candidate causal | rs879254646 | - | - | - | - | 293 |
| 237 | LDLR | c.695-1G>A | Chr19: 11217240 | NM_000527.4 | Candidate causal | rs879254652 | - | - | - | - | 294 |
| 238 | LDLR | c.1586+1G>A | Chr19: 11224439 | NM_000527.5 | Candidate causal | rs755389753 | - | - | 0 | - | 295 |
| 239 | LDLR | c.818-1G>A | Chr19: 11218067 | NM_000527.5 | Candidate causal | rs879254688 | - | - | - | - | 282 |
| 240 | LDLR | c.694+2T>C | Chr19: 11216278 | NM_000527.4 | Candidate causal | rs200238879 | - | 0 | - | - | 296 |
| 241 | LDLR | c.2140+1G>T | Chr19: 11231199 | NM_000527.5 | Candidate causal | rs145787161 | - | 0 | - | - | 295 |
| 242 | LDLR | c.941-2A>G | Chr19: 11221326 | NM_000527.5 | Candidate causal | rs112366278 | - | - | - | - | 297 |
| 243 | LDLR | c.314-2A>C | Chr19: 11215894 | NM_000527.5 | Candidate causal | rs879254470 | - | 0 | 0 | 0 | 294 |
| 244 | LDLR | c.1358+2T>A, C | Chr19: 11224127 | NM_000527.5 | Candidate causal | rs193922567 | - | 0 | - | 0 | 298 |
| 245 | LDLR | c.1586+5G>C | Chr19: 11224443 | NM_000527.4 | Uncertain Significance | rs781362878 | - | 0 | - | 0 | 299 |
| 246 | LDLR | c.1061-1G>C | Chr19: 11222189 | NM_000527.5 | Candidate causal | rs879254774 | - | 0 | - | 0 | 300 |
| 247 | LDLR | c.1186+5G>C | Chr19: 11222320 | NM_000527.4 | Uncertain Significance | rs879254821 | - | 0 | 0 | 0 | - |
| 248 | LDLR | c.1845+2T>C | Chr19: 11227676 | NM_000527.5 | Candidate causal | rs778408161 | - | 0 | 0 | - | 301 |
| 249 | LDLR | c.313+2dup | Chr19: 11213464 | NM_000527.5 | Likely Candidate causal | rs875989897 | - | - | 0 | - | 302 |
| 250 | LDLR | c.1060+10G>A | Chr19: 11221457 | NM_000527.5 | Uncertain Significance | rs12710260 | 0.4 | 0.28 | 0.34 | 0.32 | 297 |
| 251 | LDLR | c.817+1G>A | Chr19: 11217364 | NM_000527.5 | Candidate causal | rs879254685 | - | - | 0 | - | 303 |
| 252 | LDLR | c.1186+5G>A | Chr19: 11222320 | NM_000527.5 | Uncertain Significance | rs879254821 | - | 0 | 0 | 0 | 297 |
| 253 | LDLR | c.191-2A>G | Chr19: 11213338 | NM_000527.5 | Candidate causal | rs544203837 | - | - | - | - | 304 |
| 254 | LDLR | c.313+2T>C | Chr19: 11213464 | NM_000527.5 | Candidate causal | rs793888517 | - | 0 | 0 | 0 | 305 |
| 255 | LDLR | c.2312-3C>A | Chr19: 11238681 | NM_000527.5 | Uncertain Significance | rs875989942 | - | 0 | 0 | 0 | 283 |
| 256 | LDLR | c.1571_1586+3dup | Chr19: 11224421-11224422 | NM_000527.4 | Uncertain Significance/Candidate causal | rs1555805531 | - | - | - | - | - |
| 257 | LDLR | c.70_192del | Chr19: 11210899-11211021 | NM_000527.5 | Candidate causal | rs1555802242 | - | - |  |  | - |
| 258 | LDLR | c.314-62_1186+60dup | Chr19: 11221454 | NM_000527.5 | Uncertain Significance/Candidate causal | - | - | - | - | - | - |
| 259 | LDLR | c.1606+203C>A | Chr19: 11231112 | NM_001195803.2 | Candidate causal | rs28942084 | - | 0 | 0 | 0 | - |
| 260 | LDLR | c.314-1804delinsTCAAGGAAACCCGACTGCA | Chr19: 11216264 | NM_001195800.2 | Candidate causal | rs879254641 | - | - | - | - |  |
| 261 | LDLR | c.314-1805_314-1804insTCAGGGAAACCCGACTGC | Chr19: 11216262-11216263 | NM_001195800.2 | Candidate causal | rs1555803471 | - | - | - | - |  |
| 262 | LDLR | c.314-1814_314-1800del | Chr19: 11216250-11216264 | NM_001195800.2 | Candidate causal | rs1555803439 | - | - | - | - | - |
| 263 | LDLR | c.314-2007G>T | Chr19: 11216061 | NM_001195800.2 | Candidate causal | rs879254541 | - | - | - | - | - |
| 264 | LDLR | c.314-2016G>A | Chr19: 11216052 | NM_001195800.2 | Likely Candidate causal | rs1555803280 | - | - | - | - | - |
| 265 | LDLR | c.314-2046_314-2036dup | Chr19: 11216021-11216022 | NM_001195800.2 | Candidate causal | rs1555803254 | - | - | - | - | - |
| 266 | LDLR | c.314-2124_314-2110del | Chr19: 11215941-11215955 | NM_001195800.2 | Candidate causal | rs1555803186 | - | - | - | - | - |
| 267 | LDLR | c.314-2007_314-2006delinsTT | Chr19: 11216061-11216062 | NM_001195800.2 | Candidate causal | rs1135402767 | - | - | - | - | - |
| 268 | LDLR | c.940+1dup | Chr19: 11218189-11218190 | NM_000527.4 | Candidate causal | rs1057519666 | - | - | - | - | - |
| 269 | LDLR | c.314-2116dup | Chr19: 11215951-11215952 | NM_001195800.2 | Candidate causal | rs1057516133 | - | - | - | - | 294 |
| 270 | LDLR | c.190+2361del | Chr19: 11213382 | NM_001195799.2 | Candidate causal | rs1057516129 | - | - | - | - | 294 |
| 271 | LDLR | c.1060+2T>G | Chr19: 11221449 | NM_000527.4 | Candidate causal | rs774069731 | - | - | 0 | - | 294 |
| 272 | LDLR | c.2548-2A>G | Chr19: 11241955 | NM_000527.5 | Candidate causal | rs879255227 | - | - | - | - | 306 |
| 273 | LDLR | c.2389+2_2389+5delinsGGCCCCAT | Chr19: 11238763-11238766 | NM_000527.5 | Candidate causal | rs879255187 | - | - | - | - |  |
| 274 | LDLR | c.2311+1G>C | Chr19: 11234021 | NM_000527.5 | Candidate causal | rs879255175 | - | - | - | - | 282 |
| 275 | LDLR | c.2140+2T>A | Chr19: 11231200 | NM_000527.5 | Candidate causal | rs879255147 | - | 0 | 0 | - | 307 |
| 276 | LDLR | c.2140+1G>C | Chr19: 11231199 | NM_000527.5 | Candidate causal | rs145787161 | - | 0 | - | - | 308 |
| 277 | LDLR | c.1987+2_1987+34del | Chr19: 11230910-11230942 | NM_000527.4 | Candidate causal | rs879255098 | - | - | - | - | 309 |
| 278 | LDLR | c.1987+1del | Chr19: 11230909 | NM_000527.4 | Candidate causal | rs879255097 | - | - | - | - | 310 |
| 279 | LDLR | c.1846-1G>C | Chr19: 11230767 | NM_000527.5 | Candidate causal | rs879255051 | - | 0 | 0 | 0 | 311 |
| 280 | LDLR | c.1587-2A>G | Chr19: 11226768 | NM_000527.5 | Candidate causal | rs879254947 | - | - | - | - | 312 |
| 281 | LDLR | c.1358+3_1358+8del | Chr19: 11224128-11224133 | NM_000527.5 | Uncertain Significance/Candidate causal | rs879254875 | - | - | - | - | 258 |
| 282 | LDLR | c.1358+1G>T | Chr19: 11224126 | NM_000527.5 | Candidate causal | rs775924858 | - | 0 | - | - | 313 |
| 283 | LDLR | c.1135_1186+14del | Chr19: 11222262-11222327 | NM_000527.4 | Candidate causal | rs1555804793 | - | - | - | - | 295 |
| 284 | LDLR | c.1060+1G>T | Chr19: 11221448 | NM_000527.5 | Candidate causal | rs879254771 | - | - | - | - | 293 |
| 285 | LDLR | c.940+845_1186+531del | Chr19: 11219035-11222846 | NM_000527.5 | Candidate causal | - | - | - | - | - | 314 |
| 286 | LDLR | c.817+2T>C | Chr19: 11217365 | NM_000527.5 | Candidate causal | rs879254686 | - | - | - | - | 313 |
| 287 | LDLR | c.695-6_698del | Chr19: 11217235-11217244 | NM_000527.5 | Candidate causal | rs879254651 | - | - | - | - | 313 |
| 288 | LDLR | c.694+4dup | Chr19: 11216279-11216280 | NM_000527.4 | Uncertain Significance/Candidate causal | rs879254647 | - | - | - | - | 295 |
| 289 | LDLR | c.191-512_940+631del | Chr19: 11212828-11218821 | NM_000527.4 | Candidate causal | - | - | 0 | - | - | 314 |
| 290 | LDLR | c.190+2T>G | Chr19: 11211023 | NM_000527.5 | Candidate causal | rs879254430 | - | - | - | - | 315 |
| 291 | LDLR | c.190+1G>T | Chr19: 11211022 | NM_000527.5 | Candidate causal | rs879254428 | - | - | - | - | 311 |
| 292 | LDLR | c.67+4761_818-332del | Chr19: 11205052-11217736 | NM_000527.5 | Candidate causal | - | - | - | - | - | 316 |
| 293 | LDLR | c.1846-1G>A | Chr19: 11230767 | NM_000527.5 | Candidate causal | rs879255051 | - | 0 | 0 | 0 | 317 |
| 294 | LDLR | c.2140+1G>A | Chr19: 11231199 | NM_000527.5 | Candidate causal | rs145787161 | - | 0 | - | - | 302 |
| 295 | LDLR | c.190+5G>A | Chr19: 11211026 | NM_000527.5 | Uncertain Significance/Candidate causal | rs1131692190 | - | 0 | - | 0 | - |
| 296 | LDLR | c.1987+10G>T | Chr19: 11230919 | NM_000527.5 | Uncertain Significance | rs375846192 | - | 0 | 0 | 0 | - |
| 297 | LDLR | c.1586+1G>T | Chr19: 11224439 | NM_000527.5 | Candidate causal | rs755389753 | - | - | 0 | - | 318 |
| 298 | LDLR | c.190+1G>A | Chr19: 11211022 | NM_000527.5 | Candidate causal | rs879254428 | - | - | - | - | 319 |
| 299 | LDLR | c.68-5_68-2del | Chr19: 11210894-11210897 | NM_000527.4 | Candidate causal | rs879254395 | - | - | - | - | 304 |
| 300 | LDLR | c.1586+5G>A | Chr19: 11224443 | NM_000527.5 | Uncertain Significance | rs781362878 | - | 0 | - | 0 | 320 |
| 301 | LDLR | c.1187-10_1272dup | Chr19: 11223938-11223939 | NM_000527.4 | Likely Candidate causal | rs1555805127 | - | - | - | - | 321 |
| 302 | LDLR | c.2140+2T>C | Chr19: 11231200 | NM_000527.5 | Candidate causal | rs879255147 | - | 0 | 0 | - | 321 |
| 303 | LDLR | c.313+2T>A | Chr19: 11213464 | NM_000527.5 | Candidate causal | rs793888517 | - | 0 | 0 | 0 | 297 |
| 304 | LDLR | c.68-2A>T | Chr19: 11210897 | NM_000527.5 | Candidate causal | rs879254396 | - | - | - | - | 321 |
| 305 | LDLR | c.818-3C>G | Chr19: 11218065 | NM_000527.5 | Likely Candidate causal | rs886039831 | - | 0 | 0 | 0 | 322 |
| 306 | LDLR | c.2547+5G>C | Chr19: 11240351 | NM_000527.5 | Uncertain Significance | rs879255226 | - | - | - | - | 258 |
| 307 | LDLR | c.1705+2_1705+3insC | Chr19: 11226890-11226891 | NM_000527.5 | Uncertain Significance/Candidate causal | rs879254994 | - | - | - | - | 258 |
| 308 | LDLR | c.1705+1G>C | Chr19: 11226889 | NM_000527.5 | Candidate causal | rs875989926 | - | - | - | - | 323 |
| 309 | LDLR | c.1587-2A>T | Chr19: 11226768 | NM_000527.5 | Candidate causal | rs879254947 | - | - | - | - | 258 |
| 310 | LDLR | c.1586+2T>A | Chr19: 11224440 | NM_000527.5 | Candidate causal | rs879254945 | - | - | - | - | 252 |
| 311 | LDLR | c.940+1G>A | Chr19: 11218191 | NM_000527.5 | Candidate causal | rs879254729 | - | - | - | - | 258 |
| 312 | LDLR | c.694+1G>T | Chr19: 11216277 | NM_000527.5 | Candidate causal | rs879254646 | - | - | - | - | 258 |
| 313 | LDLR | c.313+6T>C | Chr19: 11213468 | NM_000527.5 | Uncertain Significance | rs879254468 | - | - | - | - | 324 |
| 314 | LDLR | c.190+2_190+3dup | Chr19: 11240188-11240347 | NM_000527.5 | Uncertain Significance/Candidate causal | - | - | - | - | - | 325 |
| 315 | LDLR | c.2389+1G>A | Chr19: 11238762 | NM_000527.5 | Candidate causal | rs879255186 | - | - | - | - | 326 |
| 316 | LDLR | c.2311+1941_*1216dup | Chr19: 11235956-11235957 | NM_000527.5 | Uncertain Significance/Candidate causal | - | - | - | - | - | 321 |
| 317 | LDLR | c.1187-1G>A | Chr19: 11223953 | NM_000527.5 | Candidate causal | rs879254824 | - | - | - | - | 258 |
| 318 | LDLR | c.1061-1G>A | Chr19: 11222189 | NM_000527.5 | Candidate causal | rs879254774 | - | 0 | 0 | 0 | 327 |
| 319 | LDLR | c.940+2T>A | Chr19: 11218192 | NM_000527.5 | Candidate causal | rs875989912 | - | - | - | - | 258 |
| 320 | LDLR | c.314-446_1187-386dup | Chr19: 11221454 | NM_000527.5 | Uncertain Significance/Candidate causal | - | - | - | - | - | 321 |
| 321 | LDLR | c.313+1dup | Chr19: 11213462-11213463 | NM_001195798.2 | Candidate causal | rs879254466 | - | - | - | - | 328 |
| 322 | LDLR | c.2140+86C>G | Chr19: 11231284 | NM_000527.5 | Likely Candidate causal | rs587776886 | - | 0 | 0 | 0 | 329 |
| 323 | LDLR | c.2312-2A>G | Chr19: 11238682 | NM_000527.5 | Candidate causal | - | - | - | - | - | - |
| 324 | LDLR | c.1988-2A>T | Chr19: 11231044 | NM_000527.5 | Candidate causal | rs879255101 | - | - | - | - | 295 |
| 325 | LDLR | c.1845+1del | Chr19: 11227674 | NM_000527.4 | Candidate causal | rs879255048 | - | - | - | - | 324 |
| 326 | LDLR | c.1706-2A>C | Chr19: 11227533 | NM_000527.5 | Candidate causal | rs878854027 | - | - | - | - | 330 |
| 327 | LDLR | c.1186+1G>A | Chr19: 11222316 | NM_000527.5 | Candidate causal | rs730880131 | - | - | - | - | 331 |
| 328 | LDLR | c.1061-2A>G | Chr19: 11222188 | NM_000527.5 | Candidate causal | rs879254773 | - | - | - | - | 332 |
| 329 | LDLR | c.68-2A>G | Chr19: 11210897 | NM_000527.5 | Candidate causal | rs879254396 | - | - | - | - | 293 |
| 330 | LDLR | c.1891_2311+1062del | Chr19: 11230813-11235082 | NM_000527.5 | Candidate causal | - | - | - | - | - | 333 |
| 331 | LDLR | c.2311+2T>G | Chr19: 11234022 | NM_000527.5 | Candidate causal | rs879255176 | - | - | - | - | 297 |
| 332 | LDLR | c.2389+5G>A | Chr19: 11238766 | NM_000527.5 | Uncertain Significance | rs879255191 | - | - | - | - | 319 |
| 333 | LDLR | c.1845+1G>T | Chr19: 11227675 | NM_000527.5 | Candidate causal | rs879255049 | - | - | - | - | 321 |
| 334 | LDLR | c.313+1G>T | Chr19: 11213463 | NM_000527.5 | Candidate causal | rs112029328 | - | 0 | 0 | 0 | 285 |
| 335 | LDLR | c.940+2T>C | Chr19: 11218192 | NM_000527.5 | Candidate causal | rs875989912 | - | - | - | - | 293 |
| 336 | LDLR | c.1187-10G>A | Chr19: 11223944 | NM_000527.5 | Likely Candidate causal | rs765696008 | - | 0 | 0 | 0 | 334 |
| 337 | LDLR | c.2311+1G>T | Chr19: 11234021 | NM_000527.5 | Candidate causal | rs879255175 | - | - | - | - | 258 |
| 338 | LDLR | c.1586+2T>C | Chr19: 11224440 | NM_000527.5 | Candidate causal | rs879254945 | - | - | - | - | 335 |
| 339 | LDLR | c.940+1G>C | Chr19: 11218191 | NM_000527.5 | Candidate causal | rs879254729 | - | - | - | - | 258 |
| 340 | LDLR | c.314-2019del | Chr19: 11216048 | NM_001195800.2 | Candidate causal | rs1057516135 | - | - | - | - | - |
| 341 | LDLR | c.2389+2T>G | Chr19: 11238763 | NM_000527.5 | Candidate causal | rs879255188 | - | - | - | - | 327 |
| 342 | LDLR | c.2389+1G>T | Chr19: 11238762 | NM_000527.5 | Candidate causal | rs879255186 | - | - | - | - | 304 |
| 343 | LDLR | c.1187-2A>G | Chr19: 11223952 | NM_000527.5 | Candidate causal | rs879254823 | - | - | - | - | 313 |
| 344 | LDLR | c.313+5G>A | Chr19: 11213463 | NM_000527.4 | Uncertain Significance | rs879254467 | - | 0 | - | - | 313 |
| 345 | LDLR | c.313+1G>A | Chr19: 11213463 | NM_000527.4 | Candidate causal | rs112029328 | - | 0 | 0 | 0 | 336 |
| 346 | LDLR | c.2390-1G>C | Chr19: 11240188 | NM_000527.5 | Candidate causal | rs879255193 | - | - | - | - | 297 |
| 347 | LDLR | c.941-12G>A | Chr19: 11221316 | NM_000527.5 | Uncertain Significance | rs879254734 | - | - | - | - | 258 |
| 348 | LDLR | c.1845+1G>C | Chr19: 11227675 | NM_000527.5 | Candidate causal | rs879255049 | - | - | - | - | 337 |
| 349 | LDLR | c.1845+1G>A | Chr19: 11227675 | NM_000527.5 | Candidate causal | rs879255049 | - | - | - | - | 303 |
| 350 | LDLR | c.314-1G>A | Chr19: 11215895 | NM_000527.5 | Candidate causal | rs879254471 | - | - | - | - | 338 |
| 351 | LDLR | c.1706-10G>A | Chr19: 11227525 | NM_000527.5 | Uncertain Significance | rs17248882 | 0.01 | 0 | 0 | 0 | 338 |
| 352 | LDLR | c.190+4A>T | Chr19: 11211025 | NM_000527.5 | Uncertain Significance | rs769446356 | - | 0 | 0 | 0 | 339 |
| 353 | LDLR | c.1705+1G>T | Chr19: 11226889 | NM_000527.5 | Candidate causal | rs875989926 | - | - | - | - | 323 |
| 354 | LDLR | c.314-1832_314-1830del | Chr19: 11216233-11216235 | NM_001195800.2 | Candidate causal | rs121908027 | - | 0 | 0 | 0 | 321 |
| 355 | LDLR | c.1358+1G>A | Chr19: 11224126 | NM_000527.5 | Candidate causal | rs775924858 | - | 0 | - | - | 302 |
| 356 | LDLR | c.1845+11C>G | Chr19: 11227685 | NM_000527.5 | Likely Candidate causal | rs370245937 | - | 0 | 0 | 0 | 285 |
| 357 | LDLR | c.68-4038G>A | Chr19:11206861 | NM_000527.5 | Uncertain Significance | rs17242388 | - | 0 | 0 | 0 | 340 |
| 358 | LDLR | c.2140+103G>T | Chr19: 11231301 | NM_000527.5 | Uncertain Significance | - | - | - | - | - | 341 |
| 359 | LDLR, LDLR-AS1 | c.-142C>G | Chr19: 11200083 | NM_000527.4 | Uncertain Significance | rs879254370 | - | 0 | - | 0 | - |
| 360 | LDLR, LDLR-AS1 | c.-153C>T | Chr19: 11200072 | NM_000527.4 | Uncertain Significance | rs879254366 | - | 0 | 0 | 0 | 321 |
| 361 | LDLR, LDLR-AS1 | c.-156C>T | Chr19: 11200069 | NM_000527.4 | Uncertain Significance | rs879254364 | - | - | - | - | 342 |
| 362 | LDLR, LDLR-AS1 | c.-120C>T | Chr19: 11200105 | NM_000527.4 | Uncertain Significance | rs875989886 | - | 0 | 0 | 0 | 330 |
| 363 | LDLR, LDLR-AS1 | c.-152C>T | Chr19: 11200073 | NM_000527.4 | Uncertain Significance | rs879254367 | - | 0 | - | 0 | 343 |
| 364 | LDLR, LDLR-AS1 | c.-121T>C | Chr19: 11200104 | NM_000527.4 | Uncertain Significance | rs777716188 | - | 0 | 0 | 0 | 344 |
| 365 | LDLR, LDLR-AS1 | c.-135C>G | Chr19: 11200090 | NM_000527.4 | Uncertain Significance | rs879254375 | - | - | 0 | - | 302 |
| 366 | LDLR, LDLR-AS1 | c.-99A>G | Chr19: 11200126 | NM_000527.4 | Uncertain Significance/Candidate causal | rs1057519650 | - | - | - | - | - |
| 367 | LDLR, LDLR-AS1 | c.-124dup | Chr19: 11200098-11200099 | NM_000527.4 | Uncertain Significance/Candidate causal | rs1057519649 | - | - | - | - | - |
| 368 | LDLR, LDLR-AS1 | c.-155_-150del | Chr19: 11200070-11200075 | NM_000527.4 | Uncertain Significance/Candidate causal | rs1057519648 | - | - | - | - | - |
| 369 | LDLR, LDLR-AS1 | c.-168A>G | Chr19: 11200057 | NM_000527.4 | Uncertain Significance/Candidate causal | rs1057519647 | - | - | - | - | - |
| 370 | LDLR, LDLR-AS1 | c.-185_-183del | Chr19: 11200039-11200041 | NM_000527.4 | Uncertain Significance/Candidate causal | rs879254363 | - | - | - | - | 345 |
| 371 | LDLR, LDLR-AS1 | c.-140C>A | Chr19: 11200085 | NM_000527.4 | Uncertain Significance/Candidate causal | rs875989887 | - | - | - | - | - |
| 372 | LDLR, LDLR-AS1 | c.-136C>G | Chr19: 11200089 | NM_000527.4 | Uncertain Significance | rs879254374 | - | - | - | - | 346 |
| 373 | LDLR, LDLR-AS1 | c.-139C>G | Chr19: 11200086 | NM_000527.4 | Uncertain Significance | rs879254371 | - | 0 | 0 | - | 347 |
| 374 | LDLR, LDLR-AS1 | c.67+2T>A | Chr19: 11200293 | NM_000527.5 | Candidate causal | rs879254394 | - | - | - | - | 297 |
| 375 | LDLR, LDLR-AS1 | c.-138delT | Chr19: 11200087 | NM_000527.4 | Likely Candidate causal | rs387906307 | - | - | - | - | 348 |
| 376 | LDLR, LDLR-AS1 | c.-140C>T | Chr19: 11200085 | NM_000527.4 | Uncertain Significance/Candidate causal | rs875989887 | - | - | - | - | 346 |
| 377 | LDLR, LDLR-AS1 | c.-136C>T | Chr19: 11200089 | NM_000527.4 | Uncertain Significance | rs879254374 | - | - | - | - | 293 |
| 378 | LDLR|MIR6886 | c.1357_1359-37del | Chr19: 11224122-11224172 | NM_000527.4 | Candidate causal | rs1600726930 | - | - | - | - | - |
| 379 | LDLR|MIR6886 | c.1359-5C>G | Chr19: 11224206 | NM_000527.5 | Uncertain Significance | rs531005522 | - | 0 | 0 | 0 | 324 |
| 380 | LDLR|MIR6886 | c.1359-1G>A | Chr19: 11224210 | NM_000527.5 | Candidate causal | rs139617694 | - | 0 | 0 | 0 | 302 |
| 381 | LDLR|MIR6886 | c.1359-31_1359-23delinsCGGCT | Chr19: 11224180-11224188 | NM_000527.5 | Uncertain Significance | rs879254876 | - | - | - | - | 349 |
| 382 | LDLR, LDLR-AS1 | c.-97G>A | Chr19: 11200128 | NM_000527.4 | Uncertain Significance | rs944580031 | - | 0 | 0 | - | 350 |
| 383 | LDLR, LDLR-AS1 | c.-101T>C | Chr19: 11200124 | NM_000527.4 | Uncertain Significance | rs747068848 | - | 0 | - | - | 313 |
| 384 | LDLR, LDLR-AS1 | c.-149C>A | Chr19: 11200076 | NM_000527.4 | Uncertain Significance | rs879254368 | - | - | - | - | 321 |
| 385 | LDLR, LDLR-AS1 | c.-138T>C | Chr19: 11200087 | NM_000527.4 | Uncertain Significance | rs879254372 | - | - | - | - | 351 |
| 386 | LDLR, LDLR-AS1 | c.-140C>G | Chr19: 11200085 | NM_000527.4 | Uncertain Significance/Candidate causal | rs875989887 | - | - | - | - | 330 |
| 387 | TRIM32|ASTN2 | c.2806+26456_2806+26462del | Chr9: 119461588-119461594 | NM_001365068.1 | Candidate causal | rs886044106 | - | 0 | 0 | 0 | - |
| 388 | TRIM32|ASTN2 | c.2806+27641G>A | Chr9: 119460409 | NM_001365068.1 | Likely Candidate causal | rs111033571 | - | 0 | 0 | 0 | 352 |
| 389 | TRIM32|ASTN2 | c.2806+26922del | Chr9: 119461128 | NM_001365068.1 | Candidate causal | rs759376012 | - | 0 | 0 | 0 | 244 |
| 390 | TRIM32|ASTN2 | c.2806+26807G>A | Chr9: 119461243 | NM_001365068.1 | Uncertain Significance/Candidate causal | rs3747835 | - | 0 | 0 | 0 | 353 |
| 391 | TRIM32|ASTN2 | c.2806+26570C>T | Chr9: 119461480 | NM_001365068.1 | Candidate causal | rs111033570 | - | 0 | 0 | 0 | 244 |
| 392 | TTC8 | c.1049+2_1049+4del | Chr14: 89336544-89336546 | NM_144596.3 | Candidate causal | rs587777807 | - | - | 0 | - | 354 |
| 393 | WASHC5 | c.1151-2A>G | Chr8: 126079963 | NM_014846.4 | Candidate causal | rs1563627853 | - | - | - | - | 244 |
| 394 | WASHC5 | c.3335+2T>A | Chr8: 126044481 | NM_014846.4 | Candidate causal | rs398123007 | - | 0 | - | - | 355 |
| 395 | WDPCP | c.633+2T>C | Chr2: 63664553 | NM_015910.7 | Candidate causal | rs765356177 | - | 0 | 0 | 0 | 244 |
| 396 | WDPCP | c.1916-2A>G | Chr2: 63401969 | NM_015910.7 | Candidate causal | rs763299856 | - | 0 | 0 | 0 | 244 |
| 397 | WDPCP | c.208+1G>A | Chr2: 63714580 | NM_015910.7 | Candidate causal | rs187135801 | - | 0 | 0 | - | 356 |
| 398 | SLC25A13 | c.1311+1G>A | Chr7: 95799356 | NM_014251.3 | Candidate causal | rs80338723 | - | 0 | 0 | 0 | 357 |
| 399 | SLC25A13 | c.1177+1G>A | Chr7: 95813588 | NM_014251.3 | Candidate causal | rs80338722 | - | 0 | 0 | 0 | 357 |
| 400 | SLC25A13 | c.615+1G>C | Chr7: 95822348 | NM_014251.3 | Candidate causal | rs80338718 | - | - | - | - | 358 |
| 401 | PLIN1 | c.1210-1G>T | Chr15: 90209174 | NM_014251.3 | Uncertain Significance/Candidate causal | rs1567075176 | - | - | - | - | 359 |
| 402 | SLC25A13 | c.848+3A>C | Chr7: 95818890 | NM_014251.3 | Uncertain Significance/Candidate causal | rs369402461 | - | 0 | 0 | 0 |  |
| 403 | SLC25A13 | c.468+1G>C | Chr7: 95838149 | NM_014251.3 | Candidate causal | rs1060499612 | - | 0 | 0 | - |  |
| 404 | SLC25A13 | c.1453-1G>A | Chr7: 95761194 | NM_014251.3 | Candidate causal | - | - | - | - | - |  |
| 405 | SLC25A13 | c.328+1G>T | Chr7: 95864113 | NM_014251.3 | Candidate causal | - | - | - | - | - |  |
| 406 | SLC25A13 | c.70-1G>A | Chr7: 95906651 | NM_014251.3 | Candidate causal | rs962082210 | - | 0 | 0 | 0 |  |
| 407 | SLC25A13 | c.848+1G>T | Chr7: 95818892 | NM_014251.3 | Candidate causal | rs761370420 | - | 0 | 0 | - |  |
| 408 | SLC25A13 | c.615+5G>A | Chr7: 95822344 | NM_014251.3 | Likely Candidate causal | rs80338717 | - | 0 | 0 | 0 |  |

Table 2. The candidate causal/likely candidate causal non-coding variants in Hypertrophic cardiomyopathy (HCM).

| No. | Gene | Variant | Genomic location (hg19) | Transcript | Classification | dbSNP | Frequency of variants (%) | | | | Ref. |
| --- | --- | --- | --- | --- | --- | --- | --- | --- | --- | --- | --- |
|  |  |  |  |  |  |  | **Iranome** | **1000 Genome** | **genomAD** | **TOPMED** |  |
| 1 | AGL | c.958+1G>A | Chr1: 100336426 | NM_000642.3 | Candidate causal | rs1553184657 | - | 0 | - | - | 360 |
| 2 | AGL | c.2681+1G>T | Chr1: 100350260 | NM_000642.3 | Candidate causal | rs201201443 | 0 | 0 | - | - | 361 |
| 3 | AGL | c.2681+1G>A | Chr1: 100350260 | NM_000642.3 | Candidate causal | rs201201443 | 0 | 0 | - | - | 361 |
| 4 | AGL | c.3481_3589-407del | Chr1: 100366310 | NM_000642.3 | Candidate causal | - | - | - | - | - | - |
| 5 | AGL | c.3836+1G>A | Chr1: 100376404 | NM_000642.3 | Candidate causal | rs780883601 | - | 0 | 0 | 0 | 361 |
| 6 | AGL | c.4347+1G>A | Chr1: 100382054 | NM_000642.3 | Candidate causal | rs1553193463 | - | 0 | - | 0 | 362 |
| 7 | AGL | c.4481+1G>C | Chr1: 100382288 | NM_000642.3 | Candidate causal | rs1553193529 | - | - | - | - | - |
| 8 | AGL | c.3362+1G>A | Chr1: 100361945 | NM_000642.3 | Candidate causal | rs1553189468 | - | - | - | - | - |
| 9 | AGL | c.2950-2A>C | Chr1: 100357160 | NM_000642.3 | Candidate causal | rs770438130 | - | - | 0 | - | - |
| 10 | AGL | c.3941_3949+12del | Chr1: 100378064-100378084 | NM_000642.3 | Candidate causal | rs1553192718 | - | - | - | - | - |
| 11 | AGL | c.2682-2A>G | Chr1: 100353532 | NM_000642.3 | Candidate causal | rs1185321132 | - | 0 | 0 | - | - |
| 12 | AGL | c.4481+2T>G | Chr1: 100382289 | NM_000642.3 | Candidate causal | rs1553193530 | - | 0 | - | 0 | - |
| 13 | AGL | c.1082+1G>C | Chr1: 100340367 | NM_000642.3 | Candidate causal | rs751112302 | - | 0 | 0 | 0 | - |
| 14 | AGL | c.2949+2T>A | Chr1: 100356914 | NM_000642.3 | Candidate causal | rs750492389 | - | - | - | - | - |
| 15 | AGL | c.2949+2T>C | Chr1: 100356914 | NM_000642.3 | Candidate causal | rs750492389 | - | - | - | - | - |
| 16 | AGL | c.1612-1G>C | Chr1: 100345478 | NM_000642.3 | Candidate causal | rs1327892944 | - | - | 0 | - | - |
| 17 | AGL | c.3589-1G>A | Chr1: 100368238 | NM_000642.3 | Candidate causal | rs1289339080 | - | - | 0 | - | - |
| 18 | AGL | c.460+1G>A | Chr1: 100327980 | NM_000642.3 | Candidate causal | rs930434905 | - | - | - | - | - |
| 19 | AGL | c.294-1G>C | Chr1: 100327812 | NM_000642.3 | Candidate causal | rs7579067016 | - | - | - | - | - |
| 20 | AGL | c.3083+2T>C | Chr1: 100357297 | NM_000642.3 | Candidate causal | rs1057516952 | - | 0 | - | 0 | - |
| 21 | AGL | c.1185+1G>A | Chr1: 100340813 | NM_000642.3 | Candidate causal | rs1057516948 | - | 0 | - | - | - |
| 22 | AGL | c.294-2A>T | Chr1: 100327811 | NM_000642.3 | Candidate causal | rs1057516868 | - | 0 | - | - |  |
| 23 | AGL | c.2950-1G>A | Chr1: 100357161 | NM_000642.3 | Candidate causal | rs1057516793 | - | - | - | - | - |
| 24 | AGL | c.2001+2T>C | Chr1: 100346735 | NM_000642.3 | Candidate causal | rs1057516708 | - | - | - | - | - |
| 25 | AGL | c.2949+5G>A | Chr1: 100356917 | NM_000642.3 | Uncertain Significance/Candidate causal | - | - | - | - | - | - |
| 26 | AGL | c.1612-1G>A | Chr1: 100345478 | NM_000642.3 | Candidate causal | - | - | - | - | - | - |
| 27 | AGL | c.664+1G>C | Chr1: 100330146 | NM_000642.3 | Candidate causal | - | - | - | - | - | - |
| 28 | AGL | c.82+1G>A | Chr1: 100316681 | NM_000642.3 | Candidate causal | rs1354714214 | - | 0 | - | - | 363 |
| 29 | AGL | c.3363-1G>A | Chr1: 100366191 | NM_000642.3 | Candidate causal | rs1057516513 | - | - | - | - | 364 |
| 30 | AGL | c.1284-2A>G | Chr1: 100342012 | NM_000642.3 | Candidate causal | - | - | - | - | - | 365 |
| 31 | AGL | c.1900-2A>G | Chr1: 100346630 | NM_000642.3 | Candidate causal | rs1254391875 | - | - | 0 | - | 365 |
| 32 | AGL | c.664+2T>C | Chr1: 100330147 | NM_000642.3 | Candidate causal | - | - | - | - | - | 365 |
| 33 | AGL | c.664+1G>A | Chr1: 100330146 | NM_000642.3 | Candidate causal | rs61811105 | - | - | - | - | 363 |
| 34 | AGL | c.2001+5G>A | Chr1: 100346738 | NM_000642.3 | Uncertain Significance/Candidate causal | rs1553186489 | - | - | - | - | 362 |
| 35 | AGL | c.4347+1G>C | Chr1: 100382054 | NM_000642.3 | Candidate causal | - | - | - | - | - | 362 |
| 36 | AGL | c.3589-3C>G | Chr1: 100368236 | NM_000642.3 | Uncertain Significance/Candidate causal | - | - | - | - | - | 366 |
| 37 | AGL | c.2158-2A>G | Chr1: 100347095 | NM_000642.3 | Candidate causal | rs878959417 | - | 0 | - | 0 | 365 |
| 38 | AGL | c.4347+1G>A | Chr1: 100382054 | NM_000642.3 | Candidate causal | rs1553193463 | - | 0 | - | 0 | 362 |
| 39 | AGL | c.958+1G>T | Chr1: 100336426 | NM_000642.3 | Candidate causal | - | - | - | - | - | 367 |
| 40 | AGL | c.2309-1G>A | Chr1: 100349675 | NM_000642.3 | Candidate causal | rs786204481 | - | 0 | 0 | - | 368 |
| 41 | AGL | c.4259+5G>A | Chr1: 100381047 | NM_000642.3 | Uncertain Significance/Candidate causal | rs780504025 | - | - | 0 | - | 369 |
| 42 | AGL | c.1735+1_1735+15del | Chr1: 100345600-100345614 | NM_000642.3 | Candidate causal | - | - | - | - | - | 370 |
| 43 | AGL | c.1423+1G>C | Chr1: 100342154 | NM_000642.3 | Candidate causal | rs751952198 | - | 0 | 0 | - | 362 |
| 44 | AGL | c.958+1G>A | Chr1: 100336426 | NM_000642.3 | Candidate causal | rs1553184657 | - | 0 | - | - | 362 |
| 45 | AGL | c.293+1del | Chr1: 100327268 | NM_000642.3 | Candidate causal | rs777857395 | - | 0 | - | - | 368 |
| 46 | AGL | c.664+3A>G | Chr1: 100330148 | NM_000642.3 | Likely Candidate causal | rs370792293 | - | 0 | 0 | 0 | 371 |
| 47 | AGL | c.1735+1G>T | Chr1: 100345603 | NM_000642.3 | Candidate causal | rs199922945 | - | 0 | 0 | 0 | 372 |
| 48 | AGL | c.4260-12A>G | Chr1: 100381954 | NM_000642.3 | Likely Candidate causal | rs369973784 | - | 0 | 0 | 0 | 373 |
| 49 | LMNA | c.1968+5G>A | Chr1: 156108553 | NM_170707.4 | Uncertain Significance/Candidate causal | rs797044488 | - | - | - | - | 374 |
| 50 | TNNT2 | c.842+1G>A | Chr1: 201328750 | NM_001276345.2 | Candidate causal | rs111377893 | - | - | - | - | 375 |
| 51 | TNNT2 | c.851+1G>T | Chr1: 201328750 | NM_001276345.2 | Candidate causal | rs111377893 | - | - | - | - | - |
| 52 | TNNT2 | c.851+1G>C | Chr1: 201328750 | NM_001276345.2 | Candidate causal | rs111377893 | - | - | - | - | - |
| 53 | TNNT2 | c.851+1G>A | Chr1:201328750 | NM_001276345.2 | Candidate causal | rs111377893 | - | - | - | - | 376 |
| 54 | TNNT2 | c.852-1G>T | Chr1: 201328384 | NM_001276345.2 | Candidate causal | rs730881114 | - | - | - | - | 366 |
| 55 | TNNT2 | c.851+5G>A | Chr1: 201328746 | NM_001276345.2 | Uncertain Significance | rs193922620 | - | - | - | - | - |
| 56 | TNNT2 | c.490-1G>C | Chr1: 201332535 | NM_001276345.2 | Candidate causal | rs111344408 | - | 0 | 0 | 0 | - |
| 57 | TNNT2 | c.609+1del | Chr1: 201331513 | NM_001276345.2 | Candidate causal | rs1571608729 | - | - | - | - | - |
| 58 | TPM1 | c.240+1G>A | Chr15: 63336352 | NM_001018005.2 | Candidate causal | rs730881146 | - | - | - | - | - |
| 59 | TTN | c.2371-1G>A | Chr2: 179650470 | NM_001267550.2 | Uncertain Significance/Candidate causal | rs755365744 | 0 | 0 | 0 | 0 | 377 |
| 60 | TTN | c.10361-1G>A | Chr2: 179603088 | NM_133378.4 | Candidate causal | rs869312099 | - | 0 | 0 | 0 | 378 |
| 61 | TTN | c.44281+1G>A | Chr2: 179494967 | NM_001267550.2 | Candidate causal | rs771562210 | - | 0 | 0 | - | 379 |
| 62 | TTN|TTN-AS1 | c.48160+1G>A | Chr2: 179481455 | NM_001267550.2 | Candidate causal | - | - | - | - | - | - |
| 63 | VCL | c.2746-2193_2746-2192del | Chr10: 75871749-75871750 | NM_003373.4 | Uncertain Significance | rs781036800 | - | 0 | 0 | 0 | 380 |
| 64 | COQ8A | c.656-1G>T | Chr1: 227165149 | NM_020247.5 | Candidate causal | rs903436781 | - | - | - | - | - |
| 65 | COQ8A | c.1398+2T>C | Chr1: 227171938 | NM_020247.5 | Candidate causal | rs606231138 | - | - | - | - | 381 |
| 66 | COQ8A | c.1506+1G>A | Chr1: 227172357 | NM_020247.5 | Candidate causal | rs974677376 | - | 0 | 0 | 0 | - |
| 67 | COQ8A | c.1081-1_1082dup | Chr1: 227171250-227171251 | NM_020247.5 | Candidate causal | rs1057519344 | - | 0 | - | 0 | 382 |
| 68 | SUCLG1 | c.201+1G>T | Chr2: 84676772 | NM_003849.4 | Candidate causal | rs1573374828 | - | 0 | - | - | - |
| 69 | SUCLG1 | c.97+3G>C | Chr2: 84686294 | NM_003849.4 | Uncertain Significance/Candidate causal | rs786205871 | - | 0 | 0 | 0 | - |
| 70 | SUCLG1 | c.825+1G>A | Chr2: 84658631 | NM_003849.4 | Candidate causal | rs750388794 |  | 0 | 0 | 0 | - |
| 71 | SLC25A20 | c.200_326+1del | Chr3: 48921430 | NM_000387.6 | Candidate causal | rs1553686314 | - | - | - | - | - |
| 72 | ACAD9 | c.1249C>T | Chr3: 128625063 | NM_014049.4 | Likely Candidate causal | rs368949613 | - | 0 | 0 | - | - |
| 73 | SLC25A4 | c.111+1G>A | Chr4: 186064638 | NM_001151.4 | Candidate causal | rs398122942 | - | - | - | - | - |
| 74 | NDUFA2 | c.208+5G>A | Chr5: 140026836 | NM_002488.4 | Uncertain Significance/Candidate causal | rs1168752295 | - | 0 | 0 | 0 | - |
| 75 | FBXL4 | c.1389+3_1389+6del | Chr6: 99328423 | NM_001278716.2 | Uncertain Significance/Candidate causal | rs1554216735 | - | - | - | - | - |
| 76 | FBXL4 | c.859-1G>T | Chr6: 99353547 | NM_001278716.2 | Candidate causal | rs368965675 | - | 0 | 0 | 0 | - |
| 77 | FBXL4 | c.858+1G>T | Chr6: 99365249 | NM_001278716.2 | Candidate causal | rs1394080480 | - | - | 0 | - | - |
| 78 | FBXL4 | c.513-1G>A | Chr6: 99365596 | NM_001278716.2 | Candidate causal | rs1554221258 | - | - | - | - | - |
| 79 | FBXL4 | c.858+5G>C | Chr6: 99365245 | NM_001278716.2 | Uncertain Significance/Candidate causal | rs1257765682 | - | 0 | 0 | 0 | 383 |
| 80 | PDSS1 | c.130-2A>G | Chr10: 26991089 | NM_014317.5 | Likely Candidate causal | rs752912769 | - | 0 | - | 0 | - |
| 81 | NCF1, LOC106029312 | c.*179G>A | Chr7: 74203683 | NM_000265.6 | Uncertain Significance/Candidate causal | rs1057519503 | - | - | - | - | 245 |
| 82 | FLNC | c.2390-10_2406del | Chr7: 128482838 | NM_001458.4 | Candidate causal | rs1554398674 | - | - | - | - | - |
| 83 | FLNC | c.4127+1G>T | Chr7: 128486518 | NM_001458.4 | Candidate causal | rs1346981294 | - | 0 | 0 | - | - |
| 84 | FLNC | c.1548_1549+2del | Chr7: 128480213-128480216 | NM_001458.4 | Candidate causal | rs763330423 | - | - | 0 | - | 244 |
| 85 | FLNC | c.1047+1G>T | Chr7: 128478119 | NM_001458.4 | Candidate causal | rs762493013 | - | - | 0 | - | 384 |
| 86 | FLNC | c.5199+1G>C | Chr7: 128489633 | NM_001458.4 | Candidate causal | - | - | - | - | - | 384 |
| 87 | FLNC | c.4128-2_4128-1del | Chr7: 128486797-128486798 | NM_001458.4 | Candidate causal | - | - | - | - | - | 384 |
| 88 | FLNC | c.3964+1G>A | Chr7: 128486218 | NM_001458.4 | Candidate causal | - | - | - | - | - | 384 |
| 89 | FLNC | c.4127+1G>T | Chr7: 128486518 | NM_001458.4 | Candidate causal | rs1346981294 | - | 0 | 0 | - | 384 |
| 90 | FLNC | c.2265+1G>A | Chr7: 128482429 | NM_001458.4 | Candidate causal | rs1585157354 | - | - | - | - | 384 |
| 91 | FLNC | c.2550+2T>C | Chr7: 128483010 | NM_001458.4 | Candidate causal | rs113972676 | - | - | - | - | 384 |
| 92 | FLNC | c.3193-2A>G | Chr7: 128484710 | NM_001458.4 | Candidate causal | rs749889670 | - | - | 0 | - | 384 |
| 93 | FLNC | c.4952-2A>T | Chr7: 128489383 | NM_001458.4 | Candidate causal | rs774945928 | - | - | 0 | - | 384 |
| 94 | FLNC | c.2389+2T>C | Chr7: 128482754 | NM_001458.4 | Candidate causal | rs112903432 | - | - | - | - | 384 |
| 95 | FLNC | c.699+1G>A | Chr7: 128477312 | NM_001458.4 | Candidate causal | rs1562991776 | - | - | - | - | 384 |
| 96 | FLNC | c.3791-1G>C | Chr7: 128486043 | NM_001458.4 | Candidate causal | rs781135153 | - | 0 | 0 | 0 | 384 |
| 97 | FLNC|FLNC-AS1 | c.6362-1G>T | Chr7: 128493768 | NM_001458.5 | Candidate causal | - | - | - | - | - | 384 |
| 98 | FLNC|FLNC-AS1 | c.7780+2T>C | Chr7: 128497392 | NM_001458.5 | Candidate causal | - | - | - | - | - | 384 |
| 99 | FLNC|FLNC-AS1 | c.5843-2A>G | Chr7: 128492643 | NM_001458.4 | Candidate causal | - | - | - | - | - | 384 |
| 100 | FLNC|FLNC-AS1 | c.7780+1G>A | Chr7: 128497391 | NM_001458.4 | Candidate causal | rs1563005607 | - | - | - | - | 384 |
| 101 | FLNC|FLNC-AS1 | c.7251+1G>T | Chr7: 128495369 | NM_001458.4 | Candidate causal | - | - | - | - | - | 384 |
| 102 | FLNC|FLNC-AS1 | c.7251+1G>A | Chr7: 128495369 | NM_001458.5 | Candidate causal | rs1554401581 | - | - | - | - | 384 |
| 103 | FLNC|FLNC-AS1 | c.5398+1G>C | Chr7: 128490538 | NM_001458.5 | Candidate causal | - | - | - | - | - | 384 |
| 104 | FLNC|FLNC-AS1 | c.6997+1G>T | Chr7: 128494737 | NM_001458.5 | Candidate causal | rs1585169973 | - | - | - | - | 384 |
| 105 | FLNC, FLNC-AS1 | c.5672delG | Chr7: 128491512 | NM_001458.4 | Candidate causal | rs1563001548 | - | - | - | - | - |
| 106 | FLNC, FLNC-AS1 | c.7251+1G>A | Chr7: 128495369 | NM_001458.4 | Candidate causal | rs1554401581 | - | - | - | - | - |
| 107 | BRAF | c.2128-4_2129del | Chr7: 140434570 | NM_004333.4 | Candidate causal | rs1131692058 | - | - | - | - | - |
| 108 | AGK | c.141+2T>C | Chr7: 141292987 | NM_018238.4 | Candidate causal | rs1554399572 | - | - | - | - | - |
| 109 | AGK | c.424-3C>G | Chr7: 141315268 | NM_018238.4 | Likely Candidate causal | rs766413410 | - | 0 | - | - | 385 |
| 110 | TMEM70 | c.317-2A>G | Chr8: 74893388 | NM_017866.6 | Candidate causal | rs183973249 | - | 0 | 0 | 0 | 381 |
| 111 | COQ4 | c.402+1G>C | Chr9: 131088161 | NM_016035.5 | Candidate causal | rs747779231 | - | 0 | - | - | - |
| 112 | COQ4 | c.402+1G>A | Chr9: 131088161 | NM_016035.5 | Candidate causal | rs747779231 | - | 0 | - | - | 386 |
| 113 | COQ4 | c.70+2C>G | Chr9: 131085209 | NM_016035.5 | Candidate causal | rs767839639 | - | 0 | 0 | 0 | 386 |
| 114 | COQ4 | c.300-2A>G | Chr9: 131088056 | NM_016035.5 | Candidate causal | rs141303335 | - | 0 | 0 | 0 | 386 |
| 115 | SURF1 | c.515+2T>G | Chr9: 136220602 | NM_003172.4 | Candidate causal | rs781787822 | - | - | 0 | - | - |
| 116 | SURF1 | c.323+2T>C | Chr9: 136221512 | NM_003172.4 | Candidate causal | rs1588691694 | - | - | - | - | - |
| 117 | AGPAT2 | c.661+2T>G | Chr9: 139569185 | NM_006412.4 | Candidate causal | rs1057517654 | - | - | - | - | - |
| 118 | AGPAT2 | c.589-2A>G | Chr9: 139569261 | NM_006412.4 | Candidate causal | rs116807569 | - | 0 | 0 | 0 | - |
| 119 | AGPAT2 | c.366_588+534del | Chr9: 139570503 | NM_006412.4 | Candidate causal | - | - | - | - | - | - |
| 120 | AGPAT2 | c.493-1G>C | Chr9: 139571133 | NM_006412.4 | Candidate causal | rs606231168 | - | 0 | 0 | 0 | 387 |
| 121 | AGPAT2 | c.492+1G>A | Chr9: 139571412 | NM_006412.4 | Candidate causal | rs933422777 | - | 0 | 0 | 0 | - |
| 122 | AGPAT2 | c.183-2A>G | Chr9: 139572010 | NM_006412.4 | Candidate causal | rs1057517649 | - | - | - | - | - |
| 123 | AGPAT2 | c.182+1G>A | Chr9: 139581627 | NM_006412.4 | Candidate causal | rs1057517650 | - | - | - | - | - |
| 124 | MYBPC3 | c.3627+2T>G | Chr11: 47354115 | NM_000256.3 | Candidate causal | rs1299079662 | - | - | 0 | - | 388 |
| 125 | MYBPC3 | c.3627+1G>A | Chr11: 47354116 | NM_000256.3 | Candidate causal | rs397516031 | - | - | - | - | 68 |
| 126 | MYBPC3 | c.3490+1G>A | Chr11: 47354364 | NM_000256.3 | Candidate causal | rs397516020 | - | - | 0 | - | 389 |
| 127 | MYBPC3 | c.3331-1G>A | Chr11: 47354525 | NM_000256.3 | Candidate causal | rs727504305 | - | - | - | - | - |
| 128 | MYBPC3 | c.3330+5G>C | Chr11: 47354740 | NM_000256.3 | Likely Candidate causal | rs373746463 | - | 0 | - | - | 390 |
| 129 | MYBPC3 | c.3330+5G>A | Chr11: 47354740 | NM_000256.3 | Likely Candidate causal | rs373746463 | - | 0 | - | - | 390 |
| 130 | MYBPC3 | c.3330+2T>C | Chr11: 47354743 | NM_000256.3 | Candidate causal | rs387906397 | - | 0 | 0 | 0 | - |
| 131 | MYBPC3 | c.3190+5G>A | Chr11: 47355103 | NM_000256.3 | Likely Candidate causal | rs587782958 | - | 0 | 0 | 0 | 391 |
| 132 | MYBPC3 | c.3190+2T>G | Chr11: 47355106 | NM_000256.3 | Candidate causal | rs113358486 | - | 0 | 0 | - | 392 |
| 133 | MYBPC3 | c.3190+1G>A | Chr11: 47355107 | NM_000256.3 | Candidate causal | rs111683277 | - | 0 | 0 | 0 | 393 |
| 134 | MYBPC3 | c.3079G>AA | Chr11: 47355219 | NM_000256.3 | Candidate causal | rs730880666 | - | - | - | - | 394 |
| 135 | MYBPC3 | c.2994+2T>C | Chr11: 47355471 | NM_000256.3 | Candidate causal | rs727503176 | - | - | - | - | - |
| 136 | MYBPC3 | c.2906-2A>G | Chr11: 47355563 | NM_000256.3 | Candidate causal | rs727504333 | - | - | - | - | - |
| 137 | MYBPC3 | c.2905+445_2905+448del | Chr11: 47356145 | NM_000256.3 | Uncertain Significance/Candidate causal | rs1595842238 | - | - | - | - | 395 |
| 138 | MYBPC3 | c.2905+1G>C | Chr11: 47356592 | NM_000256.3 | Candidate causal | rs397515991 | - | - | - | - | - |
| 139 | MYBPC3 | c.2737+2T>A | Chr11: 47357426 | NM_000256.3 | Candidate causal | rs727503184 | - | - | - | - | - |
| 140 | MYBPC3 | c.2737+1G>A | Chr11: 47357427 | NM_000256.3 | Candidate causal | rs727504314 | - | - | - | - | - |
| 141 | MYBPC3 | c.2737+1G>C | Chr11: 47357427 | NM_000256.3 | Candidate causal | rs727504314 | - | - | - | - | - |
| 142 | MYBPC3 | c.2413+1G>A | Chr11: 47359240 | NM_000256.3 | Candidate causal | rs1595843828 | - | - | - | - | 396 |
| 143 | MYBPC3 | c.2309-2A>G | Chr11: 47359347 | NM_000256.3 | Candidate causal | rs111729952 | - | - | - | - | 397 |
| 144 | MYBPC3 | c.2309-26A>G | Chr11: 47359371 | NM_000256.3 | Uncertain Significance/Candidate causal | rs886041030 | - | - | - | - | 245 |
| 145 | MYBPC3 | c.2308+1G>T | Chr11: 47360070 | NM_000256.3 | Candidate causal | rs112738974 | - | 0 | - | 0 | - |
| 146 | MYBPC3 | c.2308+1G>A | Chr11: 47360070 | NM_000256.3 | Candidate causal | rs112738974 | - | 0 | - | 0 | 245 |
| 147 | MYBPC3 | c.2149-1G>T | Chr11: 47360231 | NM_000256.3 | Candidate causal | rs727504334 | - | 0 | - | 0 | 245 |
| 148 | MYBPC3 | c.1928-2A>G | Chr11: 47361343 | NM_000256.3 | Candidate causal | rs397515937 | - | 0 | - | - | 245 |
| 149 | MYBPC3 | c.1927+600C>T | Chr11: 47361954 | NM_000256.3 | Likely Candidate causal | rs1595845204 | - | - | - | - | 395 |
| 150 | MYBPC3 | c.1898-1G>A | Chr11: 47362584 | NM_000256.3 | Candidate causal | rs730880558 | - | 0 | - | - | 398 |
| 151 | MYBPC3 | c.1897+1G>A | Chr11: 47362688 | NM_000256.3 | Candidate causal | rs397515935 | - | 0 | - | 0 | 399 |
| 152 | MYBPC3 | c.1791-2A>C | Chr11: 47362797 | NM_000256.3 | Candidate causal | rs112179534 | - | - | - | - | - |
| 153 | MYBPC3 | c.1458-1G>C | Chr11: 47364296 | NM_000256.3 | Candidate causal | rs397515903 | - | 0 | 0 | - | 245 |
| 154 | MYBPC3 | c.1458-1G>A | Chr11: 47364296 | NM_000256.3 | Candidate causal | rs397515903 | - | 0 | 0 | - | 397 |
| 155 | MYBPC3 | c.1227-1G>C | Chr11: 47364697 | NM_000256.3 | Candidate causal | rs113276889 | - | - | - | - | - |
| 156 | MYBPC3 | c.1227-2A>G | Chr11: 47364698 | NM_000256.3 | Candidate causal | rs730880531 | - | 0 | 0 | - | - |
| 157 | MYBPC3 | c.1224-2A>G | chr11: 47364815 | NM_000256.3 | Candidate causal | rs397515891 | - | - | - | - |  |
| 158 | MYBPC3 | c.1224-80G>A | Chr11: 47364779 | NM_000256.3 | Uncertain Significance/Candidate causal | rs1025692267 | - | 0 | 0 | 0 | - |
| 159 | MYBPC3 | c.1223+2T>G | Chr11: 47365041 | NM_000256.3 | Candidate causal | rs730880641 | - | - | - | - | - |
| 160 | MYBPC3 | c.1090+2T>G | Chr11: 47367756 | NM_000256.3 | Candidate causal | rs730880634 | - | - | - | - | - |
| 161 | MYBPC3 | c.1090+1G>T | Chr11: 47367757 | NM_000256.3 | Candidate causal | rs727504269 | - | - | 0 | - | 400 |
| 162 | MYBPC3 | c.1090+1G>A | Chr11: 47367757 | NM_000256.3 | Candidate causal | rs727504269 | - | - | 0 | - | 400 |
| 163 | MYBPC3 | c.927-2A>G | Chr11: 47367923 | NM_000256.3 | Candidate causal | rs397516082 | - | 0 | 0 | 0 | 401 |
| 164 | MYBPC3 | c.927-9G>A | Chr11: 47367930 | NM_000256.3 | Likely Candidate causal | rs397516083 | - | 0 | 0 | 0 | 402 |
| 165 | MYBPC3 | c.926+1_926+5del | Chr11: 47368173 | NM_000256.3 | Candidate causal | rs1555122811 | - | - | - | - | - |
| 166 | MYBPC3 | c.926+1G>A | Chr11: 47368177 | NM_000256.3 | Candidate causal | rs767239679 | - | - | 0 | - | - |
| 167 | MYBPC3 | c.906-1G>A,C | Chr11:47368581 | NM_000256.3 | Candidate causal | rs587776700 | - | - | 0 | - | - |
| 168 | MYBPC3 | c.906-36G>A | Chr11: 47368616 | NM_000256.3 | Likely Candidate causal | rs864622197 | - | - | - | - | - |
| 169 | MYBPC3 | c.821+2T>A | Chr11: 47369406 | NM_000256.3 | Candidate causal | rs397516076 | - | - | - | - | 403 |
| 170 | MYBPC3 | c.821+2T>C | Chr11: 47369406 | NM_000256.3 | Candidate causal | rs397516076 | - | - | - | - | - |
| 171 | MYBPC3 | c.821+1G>C | Chr11: 47369407 | NM_000256.3 | Candidate causal | rs397516073 | - | 0 | 0 | 0 | 404 |
| 172 | MYBPC3 | c.821+1G>A | Chr11: 47369407 | NM_000256.3 | Candidate causal | rs397516073 | - | 0 | 0 | 0 | 405 |
| 173 | MYBPC3 | c.655-1G>A | Chr11: 47370093 | NM_000256.3 | Candidate causal | rs397516067 | - | 0 | - | - | 399 |
| 174 | MYBPC3 | c.655-2A>C | Chr11: 47370094 | NM_000256.3 | Candidate causal | rs1219818351 | - | 0 | 0 | - | - |
| 175 | MYBPC3 | c.654+1G>A | Chr11: 47371324 | NM_000256.3 | Candidate causal | rs730880621 | - | - | 0 | - | 245 |
| 176 | MYBPC3 | c.506-1G>A | Chr11: 47371474 | NM_000256.3 | Candidate causal | rs397516056 | - | - | - | - | 406 |
| 177 | MYBPC3 | c.506-1G>T | Chr11: 47371474 | NM_000256.3 | Candidate causal | rs397516056 | - | - | - | - | - |
| 178 | MYBPC3 | c.506-2A>C | Chr11: 47371475 | NM_000256.3 | Candidate causal | rs397516057 | - | - | - | - | 407 |
| 179 | MYBPC3 | c.26-2A>G | Chr11: 47373058 | NM_000256.3 | Candidate causal | rs376395543 | - | 0 | 0 | 0 | 408 |
| 180 | MYBPC3 | c.25+1G>A | Chr11: 47374173 | NM_000256.3 | Candidate causal | rs113709679 | - | - | - | - | 409 |
| 181 | MYBPC3 | c.2994+1G>A | Chr11: 47355472 | NM_000256.3 | Candidate causal | - | - | - | - | - | - |
| 182 | MYBPC3 | c.773-2A>T | Chr11: 47369458 | NM_000256.3 | Candidate causal | rs1595848628 | - | - | - | - | - |
| 183 | MYBPC3 | c.2731_2737+19del | Chr11: 47357409-47357434 | NM_000256.3 | Candidate causal | - | - | - | - | - | - |
| 184 | MYBPC3 | c.1352-2_1355del | Chr11: 47364483 - 47364488 | NM_000256.3 | Candidate causal | - | - | - | - | - | - |
| 185 | MYBPC3 | c.927-1G>C | Chr11: 47367922 | NM_000256.3 | Candidate causal | - | - | - | - | - | - |
| 186 | MYBPC3 | c.1090+453C>T | Chr11: 47367305 | NM_000256.3 | Uncertain Significance/ Candidate causal | - | - | - | - | - | 410 |
| 187 | MYBPC3 | c.3191-11_3193del | Chr11: 47354882-47354895 | NM_000256.3 | Candidate causal | rs1565623216 | - | - | - | - | - |
| 188 | MYBPC3 | c.1227-1G>T | Chr11: 47364697 | NM_000256.3 | Candidate causal | rs113276889 | - | - | - | - | - |
| 189 | MYBPC3 | c.2905+2T>A | Chr11: 47356591 | NM_000256.3 | Candidate causal | - | - | - | - | - | 403 |
| 190 | MYBPC3 | c.2994+1del | Chr11: 47355472 | NM_000256.3 | Candidate causal | - | - | - | - | - | 403 |
| 191 | MYBPC3 | c.3191-1G>A | Chr11: 47354885 | NM_000256.3 | Candidate causal | - | - | - | - | - | 403 |
| 192 | MYBPC3 | c.3627+2T>C | Chr11: 47354115 | NM_000256.3 | Candidate causal | rs1299079662 | - | - | 0 | - | 403 |
| 193 | MYBPC3 | c.506-3_506dup | Chr11: 47371472-47371473 | NM_000256.3 | Candidate causal | rs1555123438 | - | - | - | - | 403 |
| 194 | MYBPC3 | c.905+1G>T | Chr11: 47368976 | NM_000256.3 | Candidate causal | rs767698543 | - | - | - | - | 403 |
| 195 | MYBPC3 | c.1458-1G>T | Chr11: 47364296 | NM_000256.3 | Candidate causal | - | - | - | - | - | 403 |
| 196 | MYBPC3 | c.1223+1G>T | Chr11: 47365042 | NM_000256.3 | Candidate causal | - | - | - | - | - | 411 |
| 197 | MYBPC3 | c.1223+2T>C | Chr11: 47365041 | NM_000256.3 | Candidate causal | rs730880641 | - | - | - | - | 412 |
| 198 | MYBPC3 | c.1790+1G>A | Chr11: 47363541 | NM_000256.3 | Candidate causal | rs1555122053 | - | - | - | - | 413 |
| 199 | MYBPC3 | c.3627+2del | Chr11: 47354115 | NM_000256.3 | Candidate causal | rs1555120258 | - | - | - | - | 388 |
| 200 | MYBPC3 | c.2149-2del | Chr11: 47360232 | NM_000256.3 | Candidate causal | rs1555121488 | - | - | - | - | 414 |
| 201 | MYBPC3 | c.2738-2A>T | Chr11: 47356762 | NM_000256.3 | Candidate causal | rs1595842632 | - | - | - | - | 396 |
| 202 | MYBPC3 | c.505+1G>A | Chr11: 47371564 | NM_000256.3 | Candidate causal | rs730880620 | - | - | - | - | - |
| 203 | MYBPC3 | c.821+1G>T | Chr11: 47369407 | NM_000256.3 | Candidate causal | - | - | - | - | - | 415 |
| 204 | MYBPC3 | c.2148+1G>T | Chr11: 47360874 | NM_000256.3 | Candidate causal | rs1060499604 | - | 0 | 0 | 0 | 416 |
| 205 | MYBPC3 | c.3814+1G>A | Chr11: 47353622 | NM_000256.3 | Candidate causal | rs1057521823 | - | - | - | - | 417 |
| 206 | MYBPC3 | c.3491-3C>G | Chr11: 47354256 | NM_000256.3 | Uncertain Significance | rs730880592 | - | - | 0 | - | 418 |
| 207 | MYBPC3 | c.3814+2T>C | Chr11: 47353621 | NM_000256.3 | Candidate causal | rs869025470 | - | - | - | - | - |
| 208 | MYBPC3 | c.1927+2T>C | Chr11: 47362552 | NM_000256.3 | Candidate causal | rs869025467 | - | - | - | - | - |
| 209 | MYBPC3 | c.1625-1G>A | Chr11: 47363708 | NM_000256.3 | Candidate causal | rs869025466 | - | - | - | - | - |
| 210 | MYBPC3 | c.1091-1G>A | Chr11: 47365176 | NM_000256.3 | Candidate causal | rs730880143 | - | - | - | - | - |
| 211 | MYBPC3 | c.1351+1G>A | Chr11: 47364571 | NM_000256.3 | Candidate causal | rs727503204 | - | - | - | - | 419 |
| 212 | MYBPC3 | c.852-2A>G | Chr11: 47369032 | NM_000256.3 | Candidate causal | rs1565629792 | - | - | - | - | 403 |
| 213 | MYBPC3 | c.3182_3190+4del | Chr11: 47355104-47355116 | NM_000256.3 | Candidate causal | rs730880718 | - | - | - | - | 420 |
| 214 | MYBPC3 | c.1224-19G>A | Chr11: 47364832 | NM_000256.3 | Likely Candidate causal | rs587776699 | - | 0 | 0 | - | 411 |
| 215 | MYBPC3 | c.1458-6G>A | Chr11: 47364301 | NM_000256.3 | Uncertain Significance | rs375347534 | - | 0 | 0 | 0 | 421 |
| 216 | MYBPC3 | c.3331-1G>C | Chr11: 47354525 | NM_000256.3 | Candidate causal | rs727504305 | - | - | - | - | 422 |
| 217 | MYBPC3 | c.1351+2T>C | Chr11: 47364570 | NM_000256.3 | Candidate causal | rs397515897 | - | - | - | - | 412 |
| 218 | MYBPC3 | c.3191-3C>G | Chr11: 47354887 | NM_000256.3 | Uncertain Significance | rs1064793891 | - | - | 0 | - | 377 |
| 219 | MYBPC3 | c.1624+3G>C | Chr11: 47364126 | NM_000256.3 | Uncertain Significance | rs730880690 | - | - | - | - | 377 |
| 220 | MYBPC3 | c.927-10C>A | Chr11: 47367931 | NM_000256.3 | Uncertain Significance/Candidate causal | rs201078659 | 0 | 0 | 0 | - | 399 |
| 221 | MYBPC3 | c.3331-2A>C | Chr11: 47354526 | NM_000256.3 | Candidate causal | rs869025469 | - | 0 | 0 | 0 | - |
| 222 | MYBPC3 | c.1224-52G>A | Chr11: 47364865 | NM_000256.3 | Likely Candidate causal | rs786204336 | - | 0 | 0 | - | 410 |
| 223 | MYBPC3 | c.1223+1G>A | Chr11: 47365042 | NM_000256.3 | Candidate causal | rs730880639 | - | - | 0 | - | 423 |
| 224 | MYBPC3 | c.2414-1G>A | Chr11: 47359131 | NM_000256.3 | Candidate causal | rs863224899 | - | - | - | - | 424 |
| 225 | MYBPC3 | c.2737+2_2737+3del | Chr11: 47357425-47357426 | NM_000256.3 | Candidate causal | rs1265248322 | - | - | - | - | 425 |
| 226 | MYBPC3 | c.2149-1G>A | Chr11: 47360231 | NM_000256.3 | Candidate causal | rs727504334 | - | 0 | - | 0 | 415 |
| 227 | MYBPC3 | c.505+5G>C | Chr11: 47371560 | NM_000256.3 | Likely Candidate causal | rs727503219 | - | - | - | - | 426 |
| 228 | MYBPC3 | c.3815-1G>A | Chr11: 47353433 | NM_000256.3 | Candidate causal | rs397516044 | - | 0 | 0 | 0 | 399 |
| 229 | MYBPC3 | c.821+5G>A | Chr11: 47369403 | NM_000256.3 | Uncertain Significance | rs397516077 | - | - | - | - | 427 |
| 230 | MYBPC3 | c.2067+1G>A | Chr11: 47361201 | NM_000256.3 | Candidate causal | rs1444727212 | - | - | - | - | 428 |
| 231 | MYBPC3 | c.2737+5G>A | Chr11: 47357423 | NM_000256.3 | Uncertain Significance | rs398123280 | - | 0 | 0 | 0 | 429 |
| 232 | MYBPC3 | c.1624+4A>T | Chr11: 47364125 | NM_000256.3 | Candidate causal | rs397515916 | - | 0 | 0 | 0 | 430 |
| 233 | MYBPC3 | c.1624+2T>C | Chr11: 47364127 | NM_000256.3 | Candidate causal | rs111437311 | - | - | - | - | 425 |
| 234 | MYBPC3 | c.3491-2A>T | Chr11: 47354255 | NM_000256.3 | Candidate causal | rs397516022 | - | 0 | 0 | - | 399 |
| 235 | MYBPC3 | c.3330+5G>T | Chr11: 47354740 | NM_000256.3 | Likely Candidate causal | rs373746463 | - | 0 | - | - | 399 |
| 236 | MYBPC3 | c.3490+1G>T | Chr11: 47354364 | NM_000256.3 | Candidate causal | rs397516020 | - | - | 0 | - | 431 |
| 237 | MYBPC3 | c.772+1G>A | Chr11: 47369974 | NM_000256.3 | Candidate causal | rs397516072 | - | - | - | - | 432 |
| 238 | MYBPC3 | c.1227-13G>A | Chr11: 47364709 | NM_000256.3 | Likely Candidate causal | rs397515893 | - | 0 | 0 | 0 | 433 |
| 239 | MYBPC3 | c.821+3G>T | Chr11: 47369405 | NM_000256.3 | Uncertain Significance | rs727503213 | - | 0 | 0 | 0 | 388 |
| 240 | MYBPC3 | c.3330+2T>G | Chr11: 47354743 | NM_000256.3 | Candidate causal | rs387906397 | - | 0 | 0 | 0 | 434 |
| 241 | MYBPC3 | c.2603-2A>G | Chr11: 47357564 | NM_000256.3 | Candidate causal | rs1419155559 | - | 0 | - | 0 | 420 |
| 242 | MYBPC3 | c.3491-3C>A | Chr11: 47354256 | NM_000256.3 | Uncertain Significance/Candidate causal | rs730880592 | - | - | 0 | - | - |
| 243 | MYH7 | c.732+1G>C | Chr14: 23900793 | NM_000257.4 | Candidate causal | rs730880557 | - | - | - | - | 435 |
| 244 | MYH7 | c.732+2T>G | Chr14: 23900792 | NM_000256.3 | Candidate causal | rs1555338658 | - | - | - | - | 435 |
| 245 | MYH7 | c.345+1G>A | Chr14: 23902292 | NM_000256.3 | Candidate causal | rs11290731 | - | - | - | - | 436 |
| 246 | MYH7 | c.732+1del | Chr14: 23900793 | NM_000256.3 | Candidate causal | rs397516266 | - | - | - | - | 435 |
| 247 | MYH7 | c.732+1G>A | Chr11: 23900793 | NM_000256.3 | Candidate causal | rs730880850 | - | 0 | 0 | 0 | 435 |
| 248 | HNRNPUL2-BSCL2, BSCL2 | c.672-2A>G | Chr11: 62458895 | NM_032667.6 | Candidate causal | rs766061024 | - | 0 | 0 | 0 | - |
| 249 | HNRNPUL2-BSCL2, BSCL2 | c.672-2A>C | Chr11: 62458895 | NM_032667.6 | Candidate causal | rs766061024 | - | 0 | 0 | 0 | - |
| 250 | HNRNPUL2-BSCL2, BSCL2 | c.672-3C>G | Chr11: 62458896 | NM_032667.6 | Uncertain Significance/Candidate causal | rs786205073 | - | - | - | - | - |
| 251 | HNRNPUL2-BSCL2, BSCL2 | c.671+5G>A | Chr11: 62459843 | NM_032667.6 | Uncertain Significance/Candidate causal | rs786205072 | - | - | - | - | - |
| 252 | HNRNPUL2-BSCL2, BSCL2 | c.574-2A>G | Chr11: 62459947 | NM_032667.6 | Candidate causal | rs1013079991 | - | 0 | - | 0 | - |
| 253 | HNRNPUL2-BSCL2, BSCL2 | c.212+1G>T | Chr11: 62472772 | NM_032667.6 | Candidate causal | rs1565152616 | - | - | - | - | 437 |
| 254 | COX8A | c.115-1G>C | Chr11: 63743696 | NM_004074.3 | Candidate causal | rs869025575 | - | - | - | - | - |
| 255 | CBL | c.1096-1G>T | Chr11: 119148875 | NM_005188.3 | Candidate causal | rs397517076 | - | 0 | 0 | 0 | 438 |
| 256 | CBL | c.1096-1G>C | Chr11: 119148875 | NM_005188.3 | Candidate causal | rs397517076 | - | 0 | 0 | 0 | 439 |
| 257 | CBL | c.1096-4_1096-1del | Chr11: 119148872-119148875 | NM_005188.3 | Candidate causal | rs397517077 | - |  | - | - | - |
| 258 | CBL | c.1228-2A>G | Chr11: 119149218 | NM_005188.3 | Candidate causal | rs727504426 | - |  | - | - | 440 |
| 259 | CACNA1C | c.1114-304G>C | Chr12: 2613704 | NM_000719.7 | Candidate causal | rs786205745 | - | - | - | - | - |
| 260 | CACNA1C | c.1114-304G>A | Chr12: 2613704 | NM_000719.7 | Candidate causal | rs786205745 | - | - | - | - | - |
| 261 | KRAS | c.*22C>G | Chr12: 25362828 | NM_033360.4 | Candidate causal | rs104894362 | - | - | - | - | 441 |
| 262 | KRAS | c.*20T>A | Chr12: 25362830 | NM_033360.4 | Likely Candidate causal | rs397517042 | - | - | - | - | 442 |
| 263 | KRAS | c.*12A>T | Chr12: 25362838 | NM_033360.4 | Candidate causal | rs104894360 | - | - | - | - | 443 |
| 264 | KRAS | c.*9T>G | Chr12: 25362841 | NM_033360.4 | Likely Candidate causal | rs104894367 | - | 0 | 0 | - | - |
| 265 | KRAS | c.451-5642A>C | Chr12: 25368487 | NM_004985.5 | Likely Candidate causal | - | - | - | - | - | - |
| 266 | PKP2 | c.2014-1G>C | Chr12: 32955491 | NM_001005242.3 | Candidate causal | rs193922674 | - | 0 | 0 | 0 | ^444^, ^445^ |
| 267 | TSFM | c.997C>T | Chr12: 58190322 | NM_001172696.1 | Candidate causal | rs121909485 | - | 0 | 0 | 0 | 445 |
| 268 | SLC25A3 | c.158-237G>A | Chr12: 98989268 | NM_002635.4 | Likely Candidate causal | rs104894375 | - | 0 | - | - | - |
| 269 | SLC25A3 | c.158-303A>G | Chr12: 98989202 | NM_002635.4 | Likely Candidate causal | rs745305932 | - | 0 | 0 | 0 | 446 |
| 270 | MYL2, LOC114827850 | c.3+2T>C | Chr12: 111358329 | NM_000432.4 | Candidate causal | rs111373423 | - | - | - | - | 245 |
| 271 | COA8 | c.124-1G>A | Chr14: 104037959 | NM_001302652.2 | Candidate causal | rs587777785 | - | - | - | - | - |
| 272 | ALPK3 | c.4736-1G>A | Chr15: 85405865 | NM_020778.4 | Candidate causal | rs762110595 | - | 0 | 0 | 0 | - |
| 273 | COQ9 | c.521+1del | Chr16: 57490559 | NM_020312.4 | Candidate causal | rs786205897 | - | - | 0 | - | - |
| 274 | COQ9 | c.74-13G>A | Chr16: 57484939 | NM_020312.4 | Uncertain Significance | rs181356497 | 0 | 0 | 0 | 0 | - |
| 275 | COQ9 | c.73+9del | Chr16: 57481495 | NM_020312.4 | Uncertain Significance/Candidate causal | rs749532852 | - | 0 | - | 0 | - |
| 276 | MLYCD | c.640_641+2del | Chr16: 83940700 | NM_012213.3 | Candidate causal | rs1294168838 | - | 0 | 0 | 0 | - |
| 277 | MTO1 | c.417+2T>C | Chr6: 74176133 | NM_012123.4 | Candidate causal | rs1030161382 | - | 0 | 0 | - | 447 |
| 278 | MTO1 | c.1261-5T>G | Chr6: 74191758 | NM_012123.4 | Uncertain Significance/Candidate causal | rs1275100093 | - | 0 | 0 | - | 448 |
| 279 | ACADVL | c.342+1G>C | Chr17: 7124150 | NM_000018.4 | Candidate causal | rs780020193 | - | 0 | 0 | 0 | - |
| 280 | ACADVL | c.623-1G>A | Chr17: 7125270 | NM_000018.4 | Candidate causal | rs1597526782 | - | - | - | - | - |
| 281 | ACADVL | c.753-2A>C | Chr17: 7125494 | NM_000018.4 | Candidate causal | rs398123092 | - | 0 | 0 | 0 | 449 |
| 282 | ACADVL | c.1269+1G>A | Chr17: 7127050 | NM_000018.4 | Candidate causal | rs773401248 | - | 0 | 0 | - | 450 |
| 283 | ACADVL | c.1605+2T>C | Chr17: 7127714 | NM_000018.4 | Candidate causal | rs1597537351 | - | - | - | - | 451 |
| 284 | ACADVL | c.623-2_623-1del | Chr17: 7125269-7125270 | NM_000018.4 | Candidate causal | rs1555528265 | - | - | - | - | - |
| 285 | ACADVL | c.1606-2A>C | Chr17: 7127797 | NM_000018.4 | Candidate causal | rs113467582 | - | - | - | - | - |
| 286 | ACADVL | c.1434+2T>G | Chr17: 7127390 | NM_000018.4 | Candidate causal | rs1555528804 | - | - | - | - | - |
| 287 | ACADVL | c.1182+2T>C | Chr17: 7126558 | NM_000018.4 | Candidate causal | rs1555528635 | - | - | - | - | - |
| 288 | ACADVL | c.277+1del | Chr17: 7123995 | NM_000018.4 | Candidate causal | rs1555527741 | - | - | - | - | - |
| 289 | ACADVL | c.1606-1G>A | Chr17: 7127798 | NM_000018.4 | Candidate causal | rs1057517386 | - | - | - | - | - |
| 290 | ACADVL | c.1333-2A>T | Chr17: 7127285 | NM_000018.4 | Candidate causal | rs1057517280 | - | - | - | - | - |
| 291 | ACADVL | c.478-1G>C | Chr17: 7124856 | NM_000018.4 | Candidate causal | rs1057517130 | - | - | - | - | - |
| 292 | ACADVL | c.277+1G>T | Chr17: 7123996 | NM_000018.4 | Candidate causal | rs1057517012 | - | - | - | - | - |
| 293 | ACADVL | c.1183-1G>A | Chr17: 7126962 | NM_000018.4 | Candidate causal | rs1057516818 | - | - | - | - | - |
| 294 | ACADVL | c.1269+1del | Chr17: 7127049 | NM_000018.4 | Candidate causal | - | - | - | - | - | - |
| 295 | ACADVL | c.1533-2A>C | Chr17: 7127638 | NM_000018.4 | Candidate causal | rs996348255 | - | 0 | 0 | 0 | - |
| 296 | ACADVL | c.753-2A>G | Chr17: 7125494 | NM_000018.4 | Candidate causal | - | - | - | - | - | - |
| 297 | ACADVL | c.1752-3_1755del | Chr17: 7128124-7128130 | NM_000018.4 | Candidate causal | - | - | - | - | - | - |
| 298 | ACADVL | c.1605+2T>A | Chr17: 7127714 | NM_000018.4 | Candidate causal | - | - | - | - | - | - |
| 299 | ACADVL | c.1751+1G>A | Chr17: 7128034 | NM_000018.4 | Candidate causal | rs1567569262 | - | - | - | - | - |
| 300 | ACADVL | c.343-1G>A | Chr17: 7124242 | NM_000018.4 | Candidate causal | rs796051918 | - | - | - | - | - |
| 301 | ACADVL | c.879-8T>A | Chr17: 7125978 | NM_000018.4 | Uncertain Significance/Candidate causal | - | - | - | - | - | 452 |
| 302 | ACADVL | c.343-14T>C | Chr17: 7124229 | NM_000018.4 | Uncertain Significance | rs200368309 | - | 0 | 0 | 0 | - |
| 303 | ACADVL | c.62+6T>C | Chr17: 7123371 | NM_000018.4 | Uncertain Significance/Candidate causal | rs1555527495 | - | - | - | - | - |
| 304 | ACADVL | c.879-1G>A | Chr17: 7125985 | NM_000018.4 | Candidate causal | - | - | - | - | - | 453 |
| 305 | ACADVL | c.139-1G>T | Chr17: 7123782 | NM_000018.4 | Candidate causal | rs1597518019 | - | - | - | - | 453 |
| 306 | ACADVL | c.205-2A>G | Chr17: 7123921 | NM_000018.4 | Candidate causal | rs1597518954 | - | - | - | - | 453 |
| 307 | ACADVL | c.1751+2T>C | Chr17: 7128035 | NM_000018.4 | Candidate causal | rs1597539537 | - | - | - | - | 453 |
| 308 | ACADVL | c.138+1G>A | Chr17: 7123517 | NM_000018.4 | Candidate causal | rs747351687 | - | 0 | 0 | - | 454 |
| 309 | ACADVL | c.1532+2T>C | Chr17: 7127564 | NM_000018.4 | Candidate causal | rs111851815 | - | - | - | - | 454 |
| 310 | ACADVL | c.1752-2del | Chr17: 7128126 | NM_000018.4 | Candidate causal | rs1555529044 | - | - | - | - | 453 |
| 311 | ACADVL | c.138+2T>C | Chr17: 7123518 | NM_000018.4 | Candidate causal | rs1057516817 | - | - | 0 | - | 453 |
| 312 | ACADVL | c.1679-6G>A | Chr17: 7127955 | NM_000018.4 | Likely Candidate causal | rs113994171 | - | 0 | 0 | 0 | 455 |
| 313 | ACADVL | c.878+1G>C | Chr17: 7125622 | NM_000018.4 | Candidate causal | rs757946752 | - | 0 | 0 | 0 | 456 |
| 314 | ACADVL | c.1605+2T>C | Chr17: 7127714 | NM_000018.4 | Candidate causal | rs1597537351 | - | - | - | - | 453 |
| 315 | ACADVL | c.277+2T>G | Chr17: 7123997 | NM_000018.4 | Candidate causal | rs1555527745 | - | - | - | - | 457 |
| 316 | ACADVL | c.63-2A>C | Chr17: 7123439 | NM_000018.4 | Candidate causal | rs1555527513 | - | 0 | 0 | - | 458 |
| 317 | ACADVL | c.342+1G>C | Chr17: 7124150 | NM_000018.4 | Candidate causal | rs780020193 | - | 0 | 0 | 0 | 459 |
| 318 | ACADVL | c.138+2dup | Chr17: 7123517-7123518 | NM_000018.4 | Uncertain Significance/Candidate causal | rs1555527548 | - | - | - | - | - |
| 319 | ACADVL | c.1183-15A>G | Chr17: 7126948 | NM_000018.4 | Likely Candidate causal | rs765390290 | - | 0 | 0 | - | 453 |
| 320 | ACADVL | c.278-1G>A | Chr17: 7124084 | NM_000018.4 | Candidate causal | rs1298004609 | - | 0 | 0 | - | 454 |
| 321 | ACADVL | c.1077+1G>T | Chr17: 7126185 | NM_000018.4 | Candidate causal | rs140989450 | - | 0 | 0 | 0 | 460 |
| 322 | ACADVL | c.1077_1077+1delinsCAC | Chr17: 7126184-7126185 | NM_000018.4 | Candidate causal | rs1057516686 | - | - | - | - | 453 |
| 323 | ACADVL | c.1077+1G>A | Chr17: 7126185 | NM_000018.4 | Candidate causal | rs140989450 | - | 0 | 0 | 0 | 461 |
| 324 | ACADVL | c.1077+2T>C | Chr17: 7126186 | NM_000018.4 | Candidate causal | rs1057516370 | - | - | - | - | 453 |
| 325 | ACADVL | c.1182+1G>A | Chr17: 7126557 | NM_000018.4 | Candidate causal | rs113690956 | - | 0 | 0 | 0 | 458 |
| 326 | ACADVL|DLG4 | c.62+1G>A | Chr17: 7123366 | NM_000018.4 | Candidate causal | - | - | - | - | - | 453 |
| 327 | GAA | c.-32-2A>G | Chr17: 78078352 | NM_000152.5 | Candidate causal | rs1445232530 | - | - | 0 | - | 462 |
| 328 | GAA | c.1888+1G>A | Chr17: 78086511 | NM_000152.5 | Candidate causal | rs776325453 | - | - | 0 | - | 463 |
| 329 | GAA | c.1195-2A>G | Chr17: 78082494 | NM_000152.5 | Candidate causal | rs76536065 | - | 0 | 0 | 0 | 464 |
| 330 | GAA | c.1326+2T>C | Chr17: 78082629 | NM_000152.5 | Candidate causal | rs1434761678 | - | - | 0 | - | - |
| 331 | GAA | c.692+2T>C | Chr17: 78079695 | NM_000152.5 | Candidate causal | rs1555599171 | - | - | - | - | - |
| 332 | GAA | c.2647-1_2648del | Chr17: 78092450-78092452 | NM_000152.5 | Candidate causal | rs1555603216 | - | - | - | - | - |
| 333 | GAA | c.2040+1del | Chr17: 78086826 | NM_000152.5 | Candidate causal | rs1555601662 | - | - | - | - | - |
| 334 | GAA | c.1552-2A>G | Chr17: 78084738 | NM_000152.5 | Candidate causal | rs1555600846 | - | - |  |  | - |
| 335 | GAA | c.546+2_546+5del | Chr17: 78078931-78078934 | NM_000152.5 | Candidate causal | rs118135486 | - | 0 | 0 | 0 | - |
| 336 | GAA | c.692+1G>A | Chr17: 78079694 | NM_000152.5 | Candidate causal | rs773281453 | - | - | - | - | 465 |
| 337 | GAA | c.955+1G>A | Chr17: 78081696 | NM_000152.5 | Candidate causal | rs1403691329 | - | - | - | - | 466 |
| 338 | GAA | c.1194+5G>A | Chr17: 78082411 | NM_000152.5 | Uncertain Significance/Candidate causal | - | - | - | - | - | 467 |
| 339 | GAA | c.2189+1G>T | Chr17: 78087166 | NM_000152.5 | Candidate causal | rs1209887739 | - | 0 | 0 | 0 | 468 |
| 340 | GAA | c.-32-1G>C | Chr17: 78078353 | NM_000152.5 | Candidate causal | rs1555598460 | - | 0 | - | 0 | 469 |
| 341 | GAA | c.1076-1G>A | Chr17: 78082287 | NM_000152.5 | Candidate causal | rs1555600050 | - | 0 | 0 | 0 | 470 |
| 342 | GAA | c.1195-1G>A | Chr17: 78082495 | NM_000152.5 | Candidate causal | rs1555600166 | - | - | - | - | 471 |
| 343 | GAA | c.858+2T>A | Chr17: 78081523 | NM_000152.5 | Candidate causal | rs1555599713 | - | - | - | - | 464 |
| 344 | GAA | c.1076-2A>G | Chr17: 78082286 | NM_000152.5 | Candidate causal | rs1057516290 | - | - | - | - | 472 |
| 345 | GAA | c.1194+2T>C | Chr17: 78082408 | NM_000152.5 | Candidate causal | rs1057516215 | - | - | - | - | 473 |
| 346 | GAA | c.1326+1G>A | Chr17: 78082628 | NM_000152.5 | Candidate causal | rs1205507761 | - | 0 | - | 0 | 474 |
| 347 | GAA | c.2332-1G>C | Chr17: 78091398 | NM_000152.5 | Candidate causal | rs1555602832 | - | - | - | - | - |
| 348 | GAA | c.-32-3C>A | Chr17: 78078351 | NM_000152.5 | Uncertain Significance/Candidate causal | rs1055945806 | - | 0 | 0 | 0 | 475 |
| 349 | GAA | c.692+1G>C | Chr17: 78079694 | NM_000152.5 | Candidate causal | rs773281453 | - | - | - | - | 476 |
| 350 | GAA | c.1754+1_1754+7del | Chr17: 78085899-78085905 | NM_000152.5 | Candidate causal | - | - | - | - | - | 464 |
| 351 | GAA | c.1551+1G>C | Chr17: 78084640 | NM_000152.5 | Candidate causal | rs770780848 | - | - | 0 | - | 477 |
| 352 | GAA | c.2041-1G>A | Chr17: 78087016 | NM_000152.5 | Candidate causal | rs760731229 | - | - | 0 | - | 477 |
| 353 | GAA | c.1327-2A>G | Chr17: 78083742 | NM_000152.5 | Candidate causal | rs1410829147 | - | 0 | 0 | - | 478 |
| 354 | GAA | c.1755-1G>A | Chr17: 78086376 | NM_000152.5 | Candidate causal | - | - | - | - | - | 477 |
| 355 | GAA | c.1438-1G>T | Chr17: 78084525 | NM_000152.5 | Candidate causal | rs147804176 | - | 0 | 0 | - | 463 |
| 356 | GAA | c.2647-7G>A | Chr17: 78092445 | NM_000152.5 | Likely Candidate causal | rs192679574 | - | 0 | 0 | - | 479 |
| 357 | GAA | c.2331+2T>A | Chr17: 78090910 | NM_000152.5 | Candidate causal | rs1057517148 | - | 0 | 0 | 0 | 480 |
| 358 | GAA | c.1437+2T>C | Chr17: 78083856 | NM_000152.5 | Candidate causal | - | - | - | - | - | 481 |
| 359 | GAA | c.1195-2A>C | Chr17: 78082494 | NM_000152.5 | Candidate causal | rs765360653 | - | - | 0 | - | 482 |
| 360 | GAA | c.546+5G>T | Chr17: 78078936 | NM_000152.5 | Uncertain Significance | rs756024023 | - | 0 | - | - | 483 |
| 361 | GAA | c.2646+2T>A | Chr17: 78092158 | NM_000152.5 | Candidate causal | rs786204561 | - | 0 | - | 0 | 484 |
| 362 | GAA | c.1438-2A>G | Chr17: 78084524 | NM_000152.5 | Candidate causal | rs886043399 | - | 0 | 0 | 0 | 485 |
| 363 | GAA | c.1754+1G>A | Chr17: 78085900 | NM_000152.5 | Candidate causal | rs886043399 | - | 0 | 0 | 0 | 486 |
| 364 | GAA | c.692+5G>T | Chr17: 78079698 | NM_000152.5 | Uncertain Significance/Candidate causal | rs763027848 | - | 0 | 0 | 0 | 487 |
| 365 | GAA | c.2040+1G>T | Chr17: 78086827 | NM_000152.5 | Candidate causal | rs1057516928 | - | - | 0 | - | 488 |
| 366 | GAA | c.1076-22T>G | Chr17: 78082266 | NM_000152.5 | Likely Candidate causal | rs762260678 | - | 0 | 0 | 0 | 489 |
| 367 | GAA | c.1552-3C>G | Chr17: 78084737 | NM_000152.5 | Likely Candidate causal | rs375470378 | - | 0 | 0 | 0 | 490 |
| 368 | GAA | c.-32-13T>G | Chr17: 78078341 | NM_000152.5 | Candidate causal | rs386834236 | 0 | 0 | 0 | 0 | 491 |
| 369 | GAA | c.148_859-11del | Chr17: 78078528 | NM_000152.5 | Candidate causal | - | - | - | - | - | - |
| 370 | GAA | c.1195-19_2190-17del | Chr17: 78082477 | NM_000152.3 | Candidate causal | - | - | - | - | - | - |
| 371 | GAA | c.1437+1G>A | Chr17: 78083855 | NM_000152.5 | Candidate causal | rs1555600575 | - | - | 0 | - | 442 |
| 372 | GAA | c.1551+1G>T | Chr17: 78084640 | NM_000152.5 | Candidate causal | rs770780848 | - | - | 0 | - | 492 |
| 373 | GAA | c.1551+1G>C | Chr17: 78084640 | NM_000152.5 | Candidate causal | rs770780848 | - | - | 0 | - | 493 |
| 374 | GAA | c.1636+460_2672del | Chr17: 78085284 | NM_000152.3 | Candidate causal | - | - | - | - | - | - |
| 375 | GAA | c.2331+2T>A | Chr17: 78090910 | NM_000152.5 | Candidate causal | rs1057517148 | - | 0 | 0 | 0 | 442 |
| 376 | GAA | c.2481+110_2646+39del | Chr17: 78091650 | NM_000152.5 | Candidate causal | rs1598592604 | - | - | - | - | 494 |
| 377 | GAA | c.2483_2646+1del | Chr17: 78091992 | NM_000152.5 | Candidate causal | rs1555603048 | - | - | - | - | 442 |
| 378 | NDUFV2 | c.120+5_120+8del | Chr18: 9117902 | NM_021074.5 | Uncertain Significance/Candidate causal | rs752670374 | - | 0 | 0 | 0 | 495 |
| 379 | NDUFV2 | c.97+5G>A | Chr19:5903618 | NM_001193375.2 | Uncertain Significance/Candidate causal | rs1348957889 | - | 0 | - | 0 | - |
| 380 | MYH7B | c.750+1G>A | Chr20: 33570359 | NM_020884.5 | Candidate causal | rs1050997719 | - | 0 | 0 | - | 245 |
| 381 | TANGO2 | c.57-1743_*2502del | Chr22: 20029135 | NM_152906.7 | Likely Candidate causal | - | - | - | - | - | - |
| 382 | LZTR1 | c.-38T>A | Chr22: 21336623 | NM_006767.4 | Uncertain Significance/Candidate causal | rs1459786357 | - | 0 | 0 | 0 | 496 |
| 383 | LZTR1 | c.2220-17C>A | Chr22: 21350968 | NM_006767.4 | Uncertain Significance/Candidate causal | rs1249726034 | - | - | 0 | - | 496 |
| 384 | LZTR1 | c.1785+1G>C | Chr22: 21349017 | NM_006767.4 | Candidate causal | - | - | - | - | - | - |
| 385 | LZTR1 | c.993+1G>A | Chr22: 21346119 | NM_006767.4 | Candidate causal | rs770368435 | - | 0 | 0 | 0 | - |
| 386 | LZTR1 | c.510-2A>G | Chr22: 21343076 | NM_006767.4 | Candidate causal | rs1458682620 | - | 0 | 0 | 0 | - |
| 387 | LZTR1 | c.2220-17C>A | Chr22: 21350968 | NM_006767.4 | Uncertain Significance/Candidate causal | rs1249726034 | - | - | 0 | - | 497 |
| 388 | LZTR1 | c.2407-2A>G | Chr22: 21351519 | NM_006767.4 | Candidate causal | rs1158550690 | - | 0 | 0 | 0 | 497 |
| 389 | LZTR1 | c.1943-256C>T | Chr22: 21349779 | NM_006767.4 | Uncertain Significance | rs761685529 | 0 | 0 | 0 | 0 | 497 |
| 390 | SCO2, NCAPH2 | c.*619C>T | Chr22: 50962423 | NM_152299.4 | Candidate causal | rs74315511 | - | 0 | 0 | 0 | 498 |
| 391 | NCAPH2 | c.*880G>A | Chr22: 50962684 | NM_152299.4 | Candidate causal | rs74315510 | - | 0 | 0 | - | - |
| 392 | COX7B | c.41-2A>G | ChrX: 77158138 | NM_001866.3 | Candidate causal | rs397514584 | - | 0 | - | - | - |
| 393 | GLA | c.640-801G>A | ChrX: 100654735 | NM_000169.3 | Uncertain Significance/Candidate causal | rs199473684 | - | 0 | - | 0 | 499 |
| 394 | GLA | c.640-801G>A | ChrX:100654735 | NM_000169.3 | Uncertain Significance/Candidate causal | rs199473684 | - | 0 | - | 0 | 500 |
| 395 | HRAS | c.110_111+1dup | Chr11:534210-534211 | NM_005343.4 | Candidate causal | rs398122808 | - | - | - | - | 501 |
| 396 | IDUA | c.300-3C>G | Chr4:994397 | NM_000203.5 | Uncertain Significance | rs1226056948 | - | - | 0 | - | 502 |
| 397 | LAMP2 | c.864+3_864+6del | ChrX: 119580154 | NM_170707.4 | Likely Candidate causal | rs397516751 | - | - | - | - | 503 |
| 398 | LAMP2 | c.183+1G>A | ChrX: 119590505 | NM_170707.4 | Candidate causal | rs727503120 | - | - | - | - | 442 |
| 399 | LAMP2 | c.65-1G>C | ChrX: 119590625 | NM_170707.4 | Candidate causal | rs730880496 | - | - | - | - | - |
| 400 | LAMP2 | c.865-1G>C | ChrX: 119576518 | NM_170707.4 | Candidate causal | rs397516752 | - | - | - | - | 504 |
| 401 | LAMP2 | c.183+1G>A | ChrX:119590505 | NM_170707.4 | Candidate causal | rs727503120 | - | - | - | - | 412 |
| 402 | LAMP2 | c.929-1G>A | ChrX: 119575750 | NM_170707.4 | Candidate causal | rs727504262 | - | - | - | - | 505 |
| 403 | LAMP2 | c.864+5G>C | ChrX: 119580155 | NM_170707.4 | Uncertain Significance/ Candidate causal | rs1352584474 | - | - | - | - | 506 |
| 404 | LAMP2 | c.864+1G>T | ChrX: 119580159 | NM_170707.4 | Candidate causal | rs727503119 | - | - | - | - | - |
| 405 | LAMP2 | c.741+1G>A | ChrX: 119581695 | NM_170707.4 | Candidate causal | rs1251075016 | - | - | - | - | 506 |
| 406 | LAMP2 | c.65-2A>G | ChrX: 119590626 | NM_170707.4 | Candidate causal | rs397516743 | - | 0 | - | - | 507 |
| 407 | LAMP2 | c.64+1G>C | ChrX: 119602960 | NM_170707.4 | Candidate causal | - | - | - | - | - | 507 |
| 408 | LAMP2 | c.1093+1G>A | ChrX: 119575584 | NM_170707.4 | Candidate causal | rs727504742 | - | - | - | - | - |
| 409 | LAMP2 | c.1609-3C>G | ChrX: 156107442 | NM_170707.4 | Likely Candidate causal | rs267607581 | - | - | - | - |  |
| 410 | DNASE1L1, TAZ | c.110-2A>G | ChrX: 153640421 | NM_000116.5 | Candidate causal | rs1603376833 | - | - | - | - | - |
| 411 | TAZ | c.239-1G>C | ChrX: 153641543 | NM_000116.5 | Candidate causal | rs1603377590 | - | - | - | - | - |
| 412 | TAZ | c.239-1G>A | ChrX: 153641543 | NM_000116.5 | Candidate causal | rs1603377590 | - | - | - | - | - |
| 413 | TAZ | c.284+110G>A | ChrX: 153641699 | NM_000116.5 | Uncertain Significance/Candidate causal | rs1603377747 | - | - | - | - | - |
| 414 | TAZ | c.647-1G>C | ChrX: 153648550 | NM_000116.5 | Candidate causal | rs587776741 | - | - | - | - | - |
| 415 | TAZ | c.700-1G>A | ChrX: 153648996 | NM_000116.5 | Candidate causal | rs397515747 | - | - | - | - | 508 |
| 416 | BSCL2 | c.438+1G>A | Chr11: 62462039 | NM_032667.6 | Candidate causal | - | - | - | - | - | - |
| 417 | BSCL2|HNRNPUL2-BSCL2 | c.212+1G>T | Chr11: 62472772 | NM_032667.6 | Candidate causal | rs1565152616 | - | - | - | - | - |
| 418 | BSCL2|HNRNPUL2-BSCL2 | c.672-2A>G | Chr11:62458895 | NM_032667.6 | Candidate causal | rs766061024 | - | 0 | 0 | 0 | 509 |
| 419 | BSCL2|HNRNPUL2-BSCL2 | c.672-2A>C | Chr11: 62458895 | NM_032667.6 | Candidate causal | rs766061024 | - | 0 | 0 | 0 | 509 |
| 420 | BSCL2|HNRNPUL2-BSCL2 | c.574-2A>G | Chr11: 62459947 | NM_032667.6 | Candidate causal | rs1013079991 | - | 0 | - | 0 | 509 |
| 421 | BSCL2|HNRNPUL2-BSCL2 | c.1043-2A>C | Chr11: 62457995 | NM_032667.6 | Candidate causal | rs1565142553 | - | - | - | - | 510 |
| 422 | BSCL2|HNRNPUL2-BSCL2 | c.438+1G>A | Chr11: 62462039 | NM_032667.6 | Candidate causal | - | - | - | - | - | 511 |
| 423 | BSCL2|HNRNPUL2-BSCL2 | c.672-3C>G | Chr11: 62458896 | NM_032667.6 | Uncertain Significance/Candidate causal | rs786205073 | - | - | - | - | 511 |
| 424 | BSCL2|HNRNPUL2-BSCL2 | c.671+5G>A | Chr11: 62459843 | NM_032667.6 | Uncertain Significance/Candidate causal | rs786205072 | - | - | - | - | 511 |
| 425 | BSCL2|HNRNPUL2-BSCL2 | c.814-2A>G | Chr11: 62458615 | NM_032667.6 | Candidate causal | rs879254029 | - | - | - | - | - |
| 426 | ACTN2 | c.-490G>T | Chr1: 236882284 | NM_001278344.1 | Likely Candidate causal | rs786204949 | - | - | - | - | 512 |
| 427 | BRAF | c.2248-4_2249del | Chr7:140434570-140434575 | NM_001374258.1 | Candidate causal | rs1131692058 | - | - | - | - | - |
| 428 | CLCN1 | c.2403+5G>A | Chr7: 143044047 | NM_000083.3 | Uncertain Significance | rs1474520642 | - | - | 0 | - | - |
| 429 | COX15 | c.396-3C>G | Chr10:101486914 | NM_078470.6 | Uncertain Significance | rs200910834 | - | 0 | 0 | 0 | 513 |
| 430 | CSRP3 | c.415-2A>C | Chr11: 19206594 | NM_003476.5 | Candidate causal | - | - | - | - | - | - |
| 431 | ETFA | c.664+1G>A | Chr15: 76577977 | NM_000126.4 | Candidate causal | - | - | - | - | - | 514 |
| 432 | ETFB | c.58-57C>T | Chr19: 51857619 | NM_001985.3 | Uncertain Significance/Candidate causal | rs766066977 | - | 0 | 0 | 0 | - |
| 433 | ETFDH | c.684+2T>G | Chr4:159611579 | NM_004453.4 | Candidate causal | rs1580406119 | - | - | - | - | - |
| 434 | ETFDH | c.485_487+13del | Chr4:59605823-159605838 | NM_004453.4 | Candidate causal | - | - | - | - | - | - |
| 435 | ETFDH | c.405+3A>G | Chr4: 159603579 | NM_004453.4 | Uncertain Significance/Candidate causal | rs796051965 | - | 0 | 0 | - | 515 |
| 436 | ETFDH | c.176-2A>T | Chr4: 159603345 | NM_004453.4 | Candidate causal | rs1485038306 | - | 0 | 0 | 0 | 514 |
| 437 | ETFDH | c.487+1G>A | Chr4: 159605826 | NM_004453.4 | Candidate causal | rs1444488601 | - | 0 | - | 0 | 514 |
| 438 | ETFDH | c.1117-1G>A | Chr4: 159624574 | NM_004453.4 | Candidate causal | - | - | - | - | - | 514 |
| 439 | ETFDH | c.973-1G>A | Chr4: 159620138 | NM_004453.4 | Candidate causal | rs1580415323 | - | - | - | - | 514 |
| 440 | ETFDH | c.1285+1G>A | Chr4: 159624744 | NM_004453.4 | Candidate causal | rs767046886 | - | 0 | 0 | 0 | 514 |
| 441 | ETFDH | c.405+3A>T | Chr4:159603579 | NM_004453.4 | Uncertain Significance/Candidate causal | rs796051965 | - | 0 | - | 0 | 515 |
| 442 | MYL2 | c.403-1G>T | Chr12: 111348980 | NM_000432.4 | Candidate causal | rs199474813 | - | 0 | 0 | - | 516 |
| 443 | MYL2 | c.403-1G>C | Chr12: 111348980 | NM_000432.4 | Candidate causal | rs199474813 | - | 0 | 0 | - | 516 |
| 444 | MYL2|LOC114827850 | c.3+1G>T | Chr12: 111358330 | NM_000432.3 | Candidate causal | rs730880948 | - | 0 | 0 | 0 | - |
| 445 | POLR1C|AARS2 | c.2599-1G>A | Chr6: 44269202 | NM_020745.4 | Candidate causal | - | - | - | - | - | - |
| 446 | POLR1C|AARS2 | c.2146-2A>G | Chr6: 44270914 | NM_020745.4 | Candidate causal | rs368934219 | - | 0 | 0 | 0 | - |
| 447 | PYGL | c.528+2T>C | Chr14: 51398389 | NM_002863.5 | Candidate causal | rs1243388778 | - | - | 0 | - | 517 |
| 448 | PYGL | c.1620+1G>C | Chr14: 51379746 | NM_002863.5 | Candidate causal | rs113993981 | - | 0 | 0 | 0 | 517 |
| 449 | PYGL | c.2177+2T>C | Chr14: 51376611 | NM_002863.5 | Candidate causal | rs1368182301 | - | - | 0 | - | - |
| 450 | PYGL | c.529-1G>C | Chr14: 51390819 | NM_002863.5 | Candidate causal | rs113993974 | - | - | - |  | 518 |
| 451 | PYGL | c.1620+1G>T | Chr14: 51379746 | NM_002863.5 | Candidate causal | rs113993981 | - | 0 | 0 | 0 | 519 |
| 452 | PYGL | c.772+2_772+3del | Chr14: 51387671-51387672 | NM_002863.5 | Candidate causal | rs765425704 | - | 0 | 0 | 0 | 519 |
| 453 | PYGL | c.772+1G>A | Chr14: 51387673 | NM_002863.5 | Candidate causal | rs776545903 | - | 0 | 0 | 0 | 519 |
| 454 | PYGL | c.1620+1G>A | Chr14: 51379746 | NM_002863.5 | Candidate causal | rs113993981 | - | 0 | 0 | 0 | 519 |
| 455 | PYGL | c.1768+1G>A | Chr14: 51378873 | NM_002863.5 | Candidate causal | rs113993982 | 0 | 0 | 0 | 0 | 518 |
| 456 | TAFAZZIN | c.542-3C>G | ChrX: 153648041 | NM_000116.5 | Uncertain Significance/Candidate causal | rs781795144 | - | - | 0 | - | - |
| 457 | TAFAZZIN | c.528_541+7del | ChrX: 153647946-153647966 | NM_000116.5 | Candidate causal | - | - | - | - | - | - |
| 458 | TAFAZZIN | c.647-1G>C | ChrX: 153648550 | NM_000116.5 | Candidate causal | rs587776741 | - | - | - | - | 520 |
| 459 | TAFAZZIN | c.284+110G>A | ChrX: 153641699 | NM_000116.5 | Uncertain Significance/Candidate causal | rs1603377747 | - | - | - | - | 521 |
| 460 | TAFAZZIN | c.239-1G>C, A | ChrX: 153641543 | NM_000116.5 | Candidate causal | rs1603377590 | - | - | - | - | 522 |
| 461 | TAFAZZIN | c.700-1G>A | ChrX: 153648996 | NM_000116.5 | Candidate causal | rs397515747 | - | - | - | - | 508 |
| 462 | TAFAZZIN | c.542-2A>G | ChrX: 153648042 | NM_000116.5 | Candidate causal | - | - | - | - | - | 523 |
| 463 | TAFAZZIN | c.236_238+5del | ChrX: 153640549-153640556 | NM_000116.5 | Candidate causal | - | - | - | - | - | 523 |
| 464 | TAFAZZIN | c.238+2_238+9del | ChrX: 153640550-153640557 | NM_000116.5 | Candidate causal | rs1603376938 | - | - | - | - | 523 |
| 465 | TNNI3 | c.12-1G>T | Chr19: 55668677 | NM_000363.5 | Candidate causal | rs397516340 | - | 0 | 0 | 0 | - |
| 466 | TNNI3 | c.24+2T>A | Chr19: 55668662 | NM_000363.5 | Candidate causal | rs777702465 | - | 0 | 0 | - | 524 |
| 467 | MYBPC3 | c.1224-21A>G | Chr11: 47364834 | NM_000256.3 | Uncertain Significance | - | - | - | - | - | 525 |
| 468 | MYBPC3 | c.1898-23A>C | Chr11: 47362606 | NM_000256.3 | Uncertain Significance | - | - | - | - | - | 525 |
| 469 | MYBPC3 | c.2308+3G>C | Chr11: 47360068 | NM_000256.3 | Uncertain Significance | - | - | - | - | - | 402 |
| 470 | SCN5A | c.393-5C>A | Chr3: 38663985 | NM_001160161.2 | Uncertain Significance | rs368678204 | - | 0 | - | - | 402 |

Table 3. The candidate causal/likely candidate causal non-coding variants in Dilated cardiomyopathy (DCM).

| No. | Gene | Variant | Genomic location (hg19) | Transcript | Classification | dbSNP | Frequency of variants (%) | | | | Ref. |
| --- | --- | --- | --- | --- | --- | --- | --- | --- | --- | --- | --- |
|  |  |  |  |  |  |  | **Iranome** | **1000 Genome** | **genomAD** | **TOPMED** |  |
| 1 | TNNI3K, FPGT-TNNI3K | c.333+2T>C | Chr1: 74716455 | NM_015978.3 | Uncertain Significance/Candidate causal | rs762721434 | - | 0 | 0 | 0 | 526 |
| 2 | LMNA | c.356+1G>A | Chr1: 156085066 | NM_170707.4 | Candidate causal | rs794728589 | - | 0 | 0 | 0 | 527 |
| 3 | LMNA | c.513+45T>G | Chr1: 156100609 | NM_170707.4 | Uncertain Significance/Candidate causal | rs878853220 | - | - | - | - | 528 |
| 4 | LMNA | c.810+1G>C | Chr1: 156104767 | NM_170707.4 | Candidate causal | rs267607632 | - | - | - | - | 529 |
| 5 | LMNA | c.513+1G>C | Chr1: 156100565 | NM_170707.4 | Candidate causal | rs397517904 | - | - | - | - | 530 |
| 6 | LMNA | c.356+1G>C | Chr1: 156085066 | NM_170707.4 | Candidate causal | rs794728589 | - | 0 | 0 | 0 | 531 |
| 7 | LMNA | c.1609-3C>G | Chr1: 156107442 | NM_170707.4 | Likely Candidate causal | rs267607581 | - | - | - | - | 532 |
| 8 | LMNA | c.1609-1G>A | Chr1: 156107444 | NM_170707.4 | Candidate causal | rs111569862 | - | - | - | - | 532 |
| 9 | LMNA | c.640-10A>G | Chr1: 156104586 | NM_170707.4 | Uncertain Significance | rs80356807 | - | 0 | 0 | - | 533 |
| 10 | LMNA | c.1698+13C>A | Chr1: 156107547 | NM_170707.4 | Candidate causal | rs80338938 | - | 0 | - | - | 534 |
| 11 | LMNA | c.1608+1G>A | Chr1: 156107024 | NM_170707.4 | Candidate causal | rs267607592 | - | - | - | - | 535 |
| 12 | LMNA | c.936+2T>C | Chr1: 156105105 | NM_170707.4 | Candidate causal | rs797045011 | - | - | - | - | 536 |
| 13 | LMNA | c.1608+5G>C | Chr1: 156107028 | NM_170707.4 | Uncertain Significance/Candidate causal | rs267607539 | - | 0 | 0 | - | 537 |
| 14 | LMNA | c.640-2A>G | Chr1: 156104594 | NM_170707.4 | Candidate causal | rs1572359505 | - | - | - | - | 412 |
| 15 | TTN, TTN-AS1 | c.66769+2T>A | Chr2: 179446224 | NM_001267550.2 | Candidate causal | rs1060500483 | - | 0 | - | 0 | 379 |
| 16 | TTN, TTN-AS1 | c.59926+1G>A | Chr2: 179456704 | NM_001267550.2 | Candidate causal | rs553526525 | - | 0 | 0 | - | 538 |
| 17 | TTN, TTN-AS1 | c.49648+2del | Chr2: 179477886 | NM_001267550.2 | Candidate causal | rs727504851 | - | 0 | 0 | 0 | 379 |
| 18 | TTN | c.669+1G>A | Chr2: 179664551 | NM_001267550.2 | Candidate causal | rs1574982376 | - | - | - | - | - |
| 19 | TTN | c.10115-1G>C | Chr2: 179623900 | NM_001267550.2 | Candidate causal | - | - | - | - | - | - |
| 20 | TTN | c.33826+1G>A | Chr2: 179543473 | NM_001267550.2 | Candidate causal | rs1389908421 | - | - | 0 | - | - |
| 21 | TTN | c.44281+1G>A | Chr2: 179494967 | NM_001267550.2 | Candidate causal | rs771562210 | - | 0 | 0 | - | 379 |
| 22 | TTN | c.30709+1G>A | Chr2: 179539765 | NM_133378.4 | Candidate causal | rs577363824 | - | 0 | 0 | 0 | 539 |
| 23 | TTN | c.32888-1del | Chr2: 179547631 | NM_001267550.2 | Candidate causal | rs869312041 | - | - | - | - | 379 |
| 24 | TTN | c.45895+1G>A | Chr2: 179485441 | NM_001267550.2 | Candidate causal | rs727504589 | - | 0 | - | - | - |
| 25 | TTN | c.10361-1G>A | Chr2: 179603088 | NM_001267550.2 | Candidate causal | rs869312099 | - | 0 | 0 | 0 | 379 |
| 26 | TTN | c.31763-1G>A | Chr2: 179554624 | NM_001267550.2 | Uncertain Significance/Candidate causal | rs202234172 | - | 0 | 0 | 0 | 379 |
| 27 | TTN | c.15496+1G>T | Chr2: 179599054 | NM_001267550.2 | Candidate causal | rs397517481 | - | 0 | 0 | - | - |
| 28 | TTN | c.42946+1G>T | Chr2: 179498139 | NM_001267550.2 | Candidate causal | rs1553741531 | - | - | - | - | 539 |
| 29 | TTN | c.30895+1G>A | Chr2: 179554539 | NM_001256850.1 | Candidate causal | rs794727043 | - | 0 | - | 0 | 539 |
| 30 | TTN | c.39893-1G>A | Chr2: 179487495 | NM_001256850.1 | Candidate causal | rs749705939 | - | 0 | 0 | 0 | 540 |
| 31 | TTN | c.15496+1G>A | Chr2: 179599054 | NM_001256850.2 | Candidate causal | rs397517481 | - | 0 | 0 | - | 540 |
| 32 | TTN | c.32095+1G>A | Chr2: 179553779 | NM_001256850.2 | Uncertain Significance/Candidate causal | rs727503636 | - | 0 | 0 | 0 | 541 |
| 33 | TTN | c.34855+1G>A | Chr2: 179537361 | NM_001256850.2 | Uncertain Significance/Candidate causal | rs377319699 | - | 0 | 0 | 0 | 539 |
| 34 | TTN | c.2371-1G>A | Chr2: 179650470 | NM_001256850.2 | Uncertain Significance/Candidate causal | rs755365744 | 0 | 0 | 0 | 0 | 540 |
| 35 | TTN | c.10303+2T>C | Chr2: 179623709 | NM_001256850.2 | Candidate causal | rs371596417 | - | 0 | 0 | - | 542 |
| 36 | TTN|TTN-AS1 | c.49948+2_49948+10del | Chr2: 179477490-179477498 | NM_001267550.2 | Candidate causal | - | - | - | - | - | 542 |
| 37 | TTN|TTN-AS1 | c.51739+1G>C | Chr2: 179474410 | NM_001256850.2 | Candidate causal | rs727504799 | - | - | - | - | - |
| 38 | TTN|TTN-AS1 | c.54811+1G>A | Chr2: 179468602 | NM_001256850.2 | Candidate causal | - | - | - | - | - | 379 |
| 39 | TTN|TTN-AS1 | c.99290-6G>T | Chr2: 179402650 | NM_001256850.2 | Uncertain Significance | rs727504987 | - | 0 | 0 | - | - |
| 40 | TTN|TTN-AS1 | c.107377+1G>A | Chr2: 179393000 | NM_001256850.2 | Candidate causal | rs112188483 | - | 0 | 0 | 0 | 379 |
| 41 | TTN|TTN-AS1 | c.53881+1G>T | Chr2: 179470140 | NM_001256850.2 | Candidate causal | rs869312051 | - | - | - | - | 379 |
| 42 | TTN|TTN-AS1 | c.66161-1G>C | Chr2: 179446936 | NM_001256850.2 | Candidate causal | rs1553627403 | - | 0 | - | 0 | 379 |
| 43 | TTN|TTN-AS1 | c.98989+1G>A | Chr2: 179403672 | NM_001267550.2 | Candidate causal | rs112240298 | - | - | - | - | 379 |
| 44 | TTN|TTN-AS1 | c.52706-1G>C | Chr2: 179472809 | NM_001267550.2 | Candidate causal | - | - | - | - | - | 379 |
| 45 | TTN|TTN-AS1 | c.67637-2A>G | Chr2: 179444122 | NM_001267550.2 | Candidate causal | rs869025550 | - | - | - | - | - |
| 46 | TTN|TTN-AS1 | c.54812-1G>T | Chr2: 179467318 | NM_001267550.2 | Candidate causal | rs1416873295 | - | - | - | - | - |
| 47 | TTN|TTN-AS1 | c.80305+5G>A | Chr2: 179421975 | NM_001256850.4 | Uncertain Significance | rs148231754 | - | 0 | 0 | - | - |
| 48 | TTN|TTN-AS1 | c.55303-1G>A | Chr2: 179466515 | NM_001256850.2 | Uncertain Significance/Candidate causal | rs748369265 | - | 0 | 0 | 0 | 379 |
| 49 | TTN|TTN-AS1 | c.51436+1G>A | Chr2: 179474816 | NM_001256850.2 | Candidate causal | rs761807131 | - | 0 | 0 | 0 | 379 |
| 50 | TTN|TTN-AS1 | c.56647+1G>A | Chr2: 179463872 | NM_001256850.2 | Candidate causal | rs397517624 | - | - | - | - | 379 |
| 51 | TTN|TTN-AS1 | c.96904+1G>A | Chr2: 179407795 | NM_001256850.2 | Candidate causal | rs1553517092 | - | - | - | - | 379 |
| 52 | TTN|TTN-AS1 | c.44423-1G>A | Chr2: 179478665 | NM_001256850.1 | Candidate causal | rs869312070 | - | 0 | 0 | - | 379 |
| 53 | TTN|TTN-AS1 | c.91564+2T>C | Chr2: 179415692 | NM_001256850. 2 | Candidate causal | - | - | - | - | - | - |
| 54 | TTN|TTN-AS1 | c.86822-2A>G | Chr2: 179423366 | NM_001256850. 2 | Candidate causal | - | - | - | - | - | - |
| 55 | TTN|TTN-AS1 | c.48160+1G>A | Chr2: 179481455 | NM_001256850. 2 | Candidate causal | - | - | - | - | - | - |
| 56 | TTN|TTN-AS1 | c.89197+1G>C | Chr2: 179418640 | NM_001256850. 2 | Candidate causal | rs1131691873 | - | 0 | - | 0 | 379 |
| 57 | TTN|TTN-AS1 | c.97470_97492+248del | Chr2: 179406743-179407013 | NM_001256850. 2 | Candidate causal | rs1559108136 | - | - | - | - | 379 |
| 58 | TTN|TTN-AS1 | c.94220-1G>A | Chr2: 179412033 | NM_001256850. 2 | Candidate causal | rs1553525592 | - | - | - | - | 379 |
| 59 | TTN|TTN-AS1 | c.66769+2T>A | Chr2: 179446224 | NM_001256850. 2 | Candidate causal | rs1060500483 | - | 0 | - | 0 | 379 |
| 60 | TTN|TTN-AS1 | c.65576-2A>G | Chr2: 179447956 | NM_001256850. 2 | Candidate causal | rs869025549 | - | - | - | - | - |
| 61 | TTN|TTN-AS1 | c.89197_89197+2del | Chr2: 179418639-179418641 | NM_001256850. 2 | Candidate causal | rs397517741 | - | - | - | - | - |
| 62 | TTN|TTN-AS1 | c.97492+1G>C | Chr2: 179406990 | NM_001256850. 2 | Candidate causal | rs727505319 | - | 0 | 0 | - | 541 |
| 63 | TTN|TTN-AS1 | c.66160+1G>T | Chr2: 179447022 | NM_001256850. 2 | Candidate causal | - | - | - | - | - | 379 |
| 64 | TTN|TTN-AS1 | c.58432+2T>C | Chr2: 179458686 | NM_001256850. 2 | Candidate causal | - | - | - | - | - | 379 |
| 65 | TTN|TTN-AS1 | c.53582-1G>A | Chr2: 179470441 | NM_001256850. 2 | Candidate causal | - | - | - | - | - | 379 |
| 66 | TTN|TTN-AS1 | c.64672+1G>A | Chr2: 179449798 | NM_001256850. 2 | Candidate causal | - | - | - | - | - | 379 |
| 67 | TTN|TTN-AS1 | c.55432+1G>A | Chr2: 179466384 | NM_001256850. 2 | Candidate causal | - | - | - | - | - | 379 |
| 68 | TTN|TTN-AS1 | c.56348-1G>A | Chr2: 179464173 | NM_001256850. 2 | Candidate causal | - | - | - | - | - | 379 |
| 69 | TTN|TTN-AS1 | c.57544+1G>A | Chr2: 179462264 | NM_001256850. 2 | Candidate causal | - | - | - | - | - | 379 |
| 70 | TTN|TTN-AS1 | c.100766-2A>T | Chr2: 179400578 | NM_001256850. 2 | Candidate causal | - | - | - | - | - | 379 |
| 71 | TTN|TTN-AS1 | c.66464-2A>G | Chr2: 179446533 | NM_001256850. 2 | Candidate causal | rs1575921187 | - | - | - | - | 379 |
| 72 | TTN|TTN-AS1 | c.54314_54381+139delinsATAAGG | Chr2: 179469296-179469502 | NM_001256850. 2 | Candidate causal | rs1576327348 | - | - | - | - | 379 |
| 73 | TTN|TTN-AS1 | c.88895-1G>A | Chr2: 179418944 | NM_001256850. 2 | Candidate causal | rs1575540533 | - | - | - | - | 379 |
| 74 | TTN|TTN-AS1 | c.55121-1G>A | Chr2: 179466878 | NM_001256850. 2 | Candidate causal | rs1576297857 | - | - | - | - | 379 |
| 75 | TTN|TTN-AS1 | c.89503+1G>C | Chr2: 179418228 | NM_001256850. 2 | Candidate causal | rs1559218619 | - | - | - | - | 379 |
| 76 | TTN|TTN-AS1 | c.49345+2T>C | Chr2: 179478777 | NM_001256850. 2 | Candidate causal | rs1559805633 | - | - | - | - | 379 |
| 77 | TTN|TTN-AS1 | c.47269+2T>C | Chr2: 179482914 | NM_001256850. 2 | Candidate causal | rs1060500419 | - | - | - | - | - |
| 78 | TTN|TTN-AS1 | c.99866-1G>A | Chr2: 179401971 | NM_001256850. 2 | Candidate causal | rs876657672 | - | - | - | - | - |
| 79 | TTN|TTN-AS1 | c.64397-1G>C | Chr2: 179450075 | NM_001256850. 2 | Candidate causal | rs876657668 | - | - | - | - | - |
| 80 | TTN|TTN-AS1 | c.64094-2A>G | Chr2: 179451536 | NM_001256850. 2 | Candidate causal | rs876657667 | - | - | - | - | 543 |
| 81 | TTN|TTN-AS1 | c.69412+1G>A | Chr2: 179441649 | NM_001256850. 2 | Candidate causal | rs869312074 | - | - | - | - | 379 |
| 82 | TTN|TTN-AS1 | c.59627-1G>A | Chr2: 179457005 | NM_001256850. 2 | Candidate causal | rs869312073 | - | - | - | - | 379 |
| 83 | TTN|TTN-AS1 | c.47875+1G>A | Chr2: 179481846 | NM_001256850. 2 | Candidate causal | rs869312047 | - | - | - | - | 379 |
| 84 | TTN|TTN-AS1 | c.49346-2A>T | Chr2: 179478666 | NM_001256850. 2 | Candidate causal | rs794729263 | - | 0 | 0 | 0 | 379 |
| 85 | TTN|TTN-AS1 | c.106531+1G>A | Chr2: 179394686 | NM_001256850. 2 | Candidate causal | rs760915007 | - | 0 | 0 | - | - |
| 86 | TTN|TTN-AS1 | c.86821+2T>A | Chr2: 179424036 | NM_001256850. 2 | Candidate causal | rs397517735 | - | 0 | 0 | 0 | 541 |
| 87 | TTN|TTN-AS1 | c.63794-1G>A | Chr2: 179452145 | NM_001256850. 2 | Candidate causal | - | - | - | - | - | 544 |
| 88 | TTN|TTN-AS1 | c.48312+2T>C | Chr2: 179481204 | NM_001256850. 2 | Candidate causal | - | - | - | - | - | 379 |
| 89 | TTN|TTN-AS1 | c.89197+2T>G | Chr2: 179418639 | NM_001256850. 2 | Candidate causal | rs1575536935 | - | - | - | - | 545 |
| 90 | TTN|TTN-AS1 | c.88594+1G>T | Chr2: 179419591 | NM_001256850. 2 | Candidate causal | rs794727467 | - | 0 | - | 0 | 379 |
| 91 | TTN|TTN-AS1 | c.48958+5G>T | Chr2: 179470136 | NM_001256850. 1 | Uncertain Significance | rs753527304 | - | 0 | 0 | - | 379 |
| 92 | TTN|TTN-AS1 | c.59644+1G>A | Chr2: 179444665 | NM_133378.4 | Candidate causal | rs758279518 | - | 0 | 0 | - | 379 |
| 93 | TTN|TTN-AS1 | c.58732+2T>C | Chr2: 179458293 | NM_001267550.2 | Candidate causal | rs869312054 | - | 0 | 0 | 0 | 379 |
| 94 | TTN|TTN-AS1 | c.67349-2A>C | Chr2: 179444577 | NM_001267550.1 | Candidate causal | rs753948675 | - | 0 | 0 | 0 | 546 |
| 95 | TTN|TTN-AS1 | c.48761-1G>C | Chr2: 179479481 | NM_001267550.2 | Candidate causal | rs876657665 | - | - | - | - | 541 |
| 96 | TTN|TTN-AS1 | c.56648-1G>A | Chr2:179463790 | NM_001267550.2 | Candidate causal | rs769912484 | - | 0 | 0 | - | 379 |
| 97 | TTN|TTN-AS1 | c.68527+1G>C | Chr2: 179442714 | NM_001267550.2 | Candidate causal | rs766984722 | - | 0 | 0 | 0 | 379 |
| 98 | TTN|TTN-AS1 | c.51436+1G>T | Chr2: 179474816 | NM_001267550.2 | Candidate causal | rs761807131 | - | 0 | 0 | 0 | 379 |
| 99 | TTN|TTN-AS1 | c.48461-2A>C | Chr2: 179480213 | NM_001267550.2 | Candidate causal | rs1553705079 | - | - | - | - | 379 |
| 100 | TTN|TTN-AS1 | c.52406-2A>C | Chr2: 179473206 | NM_001267550.2 | Candidate causal | rs753798236 | - | 0 | 0 | 0 | 379 |
| 101 | TTN|TTN-AS1 | c.66770-2A>C | Chr2: 179445338 | NM_001267550.2 | Candidate causal | rs1553624468 | - | - | - | - | 379 |
| 102 | TTN|TTN-AS1 | c.54190+1G>A | Chr2: 179469713 | NM_001267550.2 | Candidate causal | rs756339648 | - | - | - | - | 547 |
| 103 | TTN|TTN-AS1 | c.107223+1G>A | Chr2: 179393254 | NM_001267550.2 | Candidate causal | rs876658102 | - | - | - | - | 379 |
| 104 | TTN|TTN-AS1 | c.107377+1G>C | Chr2: 179393000 | NM_001267550.2 | Candidate causal | - | - | - | - | - | 548 |
| 105 | DES | c.639+4_639+5del | Chr2: 220284880 | NM_001927.4 | Uncertain Significance/Candidate causal | rs730880289 | - | - | - | - | 549 |
| 106 | DES | c.735+3A>G | Chr2: 220285071 | NM_001927.4 | Likely Candidate causal | rs267607483 | - | - | - | - | 549 |
| 107 | DES | c.1289-2A>G | Chr2: 220290383 | NM_001927.4 | Candidate causal | rs398122940 | - | - | - | - | 550 |
| 108 | DES | c.735+1G>T | Chr2: 220285069 | NM_001927.4 | Candidate causal | rs397516698 | - | - | - | - | 551 |
| 109 | DES | c.1371+1G>A | Chr2: 220290468 | NM_001927.4 | Candidate causal | rs748323823 | - | 0 | 0 | 0 | 244 |
| 110 | DES | c.1288+1G>A | Chr2: 220288543 | NM_001927.4 | Candidate causal | rs112224037 | - | 0 | 0 | - | 552 |
| 111 | DES | c.735+1G>C | Chr2: 220285069 | NM_001927.4 | Candidate causal | rs397516698 | - | - | - | - | - |
| 112 | DES | c.735+1G>A | Chr2: 220285069 | NM_001927.4 | Candidate causal | rs397516698 | - | - | - | - | 156 |
| 113 | GBE1 | c.1480-2A>G | Chr3: 33055804 | NM_000404.4 | Candidate causal | rs587776526 | - | 0 | 0 | 0 | 553 |
| 114 | GBE1 | c.993-1G>T | Chr3: 81643175 | NM_000158.4 | Candidate causal | rs763016962 | - | 0 | 0 | 0 | - |
| 115 | GBE1 | c.691+5G>C | Chr3: 81698002 | NM_000158.4 | Uncertain Significance/Candidate causal | rs397515344 | - | 0 | - | 0 | - |
| 116 | GBE1 | c.691+2T>C | Chr3: 81698005 | NM_000158.4 | Candidate causal | rs192044702 | - | 0 | 0 | 0 | 554 |
| 117 | GBE1 | c.143+1G>A | Chr3: 81810525 | NM_000158.4 | Candidate causal | rs397515343 | - | 0 | - | - | - |
| 118 | GBE1 | c.1934+2T>A | Chr3: 81584344 | NM_000158.4 | Candidate causal | - | - | - | - | - | - |
| 119 | GBE1 | c.555+1G>T | Chr3: 81698946 | NM_000158.4 | Candidate causal | rs759707498 | - | 0 | 0 | 0 | - |
| 120 | GBE1 | c.1618+1G>A | Chr3: 81627075 | NM_000158.4 | Candidate causal | - | - | - |  |  | 555 |
| 121 | GBE1 | c.1803+2T>C | Chr3: 81586060 | NM_000158.4 | Candidate causal | rs539203557 | - | 0 | 0 | 0 | 555 |
| 122 | DNAJC19 | c.130-1G>C | Chr3: 180704811 | NM_145261.4 | Candidate causal | rs137854888 | - | - | 0 | - | 556 |
| 123 | DSP | c.5671_*836delinsAGAGAAGAACAGTCTT | Chr6: 7583166 | NM_004415.4 | Uncertain Significance/Candidate causal | - | - | - | - | - | - |
| 124 | DSP | c.1140+2T>G | Chr6: 7567684 | NM_004415.4 | Candidate causal | rs869025398 | - | - | - | - | - |
| 125 | DSP | c.2130+1G>A | Chr6: 7572302 | NM_004415.4 | Candidate causal | rs727505115 | - | - | - | - | - |
| 126 | DSP | c.2298-1G>C | Chr6: 7574889 | NM_004415.4 | Candidate causal | rs1417627909 | - | - | - | - | 557 |
| 127 | DSP | c.2794-1G>A | Chr6: 7577191 | NM_004415.4 | Candidate causal | - | - | - | - | - | 557 |
| 128 | DSP | c.2298-2A>C | Chr6: 7574888 | NM_004415.4 | Candidate causal | - | - | - | - | - | 557 |
| 129 | DSP | c.778-2A>G | Chr6: 7565590 | NM_004415.4 | Candidate causal | rs113726158 | - | 0 | - | 0 | 557 |
| 130 | DSP | c.1267-2A>G | Chr6: 7568668 | NM_004415.4 | Candidate causal | rs1554106830 | - | - | - | - | 557 |
| 131 | DSP | c.2793+1G>T | Chr6: 7576690 | NM_004415.4 | Candidate causal | rs1554107741 | - | - | - | - | 557 |
| 132 | DSP | c.939+1G>A | Chr6: 7565754 | NM_004415.4 | Candidate causal | rs727504443 | - | 0 | 0 | - | 557 |
| 133 | DSP | c.939+1del | Chr6: 7565754 | NM_004415.4 | Candidate causal | - | - | - | - | - | 557 |
| 134 | DSP | c.2437-1G>C | Chr6: 7575527 | NM_004415.4 | Candidate causal | rs1057517903 | - | 0 | - | - | 557 |
| 135 | DSP | c.2436+2T>C | Chr6: 7575030 | NM_004415.4 | Candidate causal | rs774514264 | - | 0 | 0 | 0 | 542 |
| 136 | EMD | c.265+2T>A | ChrX: 153608381 | NM_000117.3 | Candidate causal | rs1603365762 | - | - | - | - | - |
| 137 | FKTN | c.647+2084G>T | Chr9: 108368857 | NM_006731.2 | Likely Candidate causal | rs1554754182 | - | 0 | - | - | 558 |
| 138 | FLNC | c.1676+1G>A | Chr7: 128480729 | NM_001458.4 | Candidate causal | rs111452612 | - | - | - | - | - |
| 139 | FLNC | c.1549+2T>G | Chr7: 128480216 | NM_004415.4 | Candidate causal | rs111806457 | - | - | - | - | - |
| 140 | FLNC | c.1548_1549+2del | Chr7: 128480213-128480216 | NM_004415.4 | Candidate causal | rs763330423 | - | - | 0 | - | - |
| 141 | FLNC | c.1047+1G>T | Chr7: 128478119 | NM_001458.5 | Candidate causal | rs762493013 | - | - | 0 | - | - |
| 142 | FLNC | c.5199+1G>C | Chr7: 128489633 | NM_001458.4 | Candidate causal | - | - | - | - | - | - |
| 143 | FLNC | c.4128-2_4128-1del | Chr7: 128486797-128486798 | NM_004415.4 | Candidate causal | - | - | - | - | - | - |
| 144 | FLNC | c.3964+1G>A | Chr7: 128486218 | NM_004415.4 | Candidate causal | - | - | - | - | - | - |
| 145 | FLNC | c.2265+1G>A | Chr7: 128482429 | NM_004415.4 | Candidate causal | rs1585157354 | - | - | - | - | - |
| 146 | FLNC | c.2550+2T>C | Chr7: 128483010 | NM_004415.4 | Candidate causal | rs113972676 | - | - | - | - | - |
| 147 | FLNC | c.3193-2A>G | Chr7: 128484710 | NM_004415.4 | Candidate causal | rs749889670 | - | - | 0 | - | - |
| 148 | FLNC | c.4952-2A>T | Chr7: 128489383 | NM_004415.4 | Candidate causal | rs774945928 | - | - | 0 | - | - |
| 149 | FLNC | c.2389+2T>C | Chr7: 128482754 | NM_004415.4 | Candidate causal | rs112903432 | - | - | - | - | - |
| 150 | FLNC | c.699+1G>A | Chr7: 128477312 | NM_004415.4 | Candidate causal | rs1562991776 | - | - | - | - | - |
| 151 | FLNC | c.3791-1G>C | Chr7: 128486043 | NM_004415.4 | Candidate causal | rs781135153 | - | 0 | 0 | 0 | 559 |
| 152 | FLNC|FLNC-AS1 | c.7385-1G>A | Chr7: 128496798 | NM_004415.4 | Candidate causal | rs1585171818 | - | - | - | - | - |
| 153 | FLNC|FLNC-AS1 | c.7780+2T>C | Chr7: 128497392 | NM_004415.4 | Candidate causal | - | - | - | - | - | - |
| 154 | FLNC|FLNC-AS1 | c.5843-2A>G | Chr7: 128492643 | NM_004415.4 | Candidate causal | - | - | - | - | - | - |
| 155 | FLNC|FLNC-AS1 | c.7780+1G>A | Chr7: 128497391 | NM_004415.4 | Candidate causal | rs1563005607 | - | - | - | - | - |
| 156 | FLNC|FLNC-AS1 | c.7251+1G>T | Chr7: 128495369 | NM_004415.4 | Candidate causal | - | - | - | - | - | 560 |
| 157 | FLNC-AS1|FLNC | c.5398+1G>C | Chr7: 128490538 | NM_004415.4 | Candidate causal | - | - | - | - | - | 560 |
| 158 | FLNC-AS1|FLNC | c.6997+1G>T | Chr7: 128494737 | NM_004415.4 | Candidate causal | rs1585169973 | - | - | - | - | - |
| 159 | FLNC | c.2390-10_2406del | Chr7: 128482838 | NM_001458.4 | Candidate causal | rs1554398674 | - | - | - | - | - |
| 160 | FLNC | c.4127+1G>T | Chr7: 128486518 | NM_001458.4 | Candidate causal | rs1346981294 | - | 0 | 0 | - | - |
| 161 | FLNC, FLNC-AS1 | c.5672delG | Chr7: 128491512 | NM_001458.4 | Candidate causal | rs1563001548 | - | - | - | - | - |
| 162 | FLNC, FLNC-AS1 | c.7251+1G>A | Chr7: 128495369 | NM_001458.4 | Candidate causal | rs1554401581 | - | - | - | - | - |
| 163 | AGK | c.424-3C>G | Chr7: 141315268 | NM_018238.4 | Uncertain Significance | rs766413410 | - | 0 | - | - | 561 |
| 164 | ANO5 | c.1898+1G>A | Chr11: 22284590 | NM_213599.2 | Candidate causal | rs142027093 | - | 0 | 0 | 0 | 562 |
| 165 | MYH7 | c.5655+5G>C | Chr14: 23883211 | NM_000257.4 | Uncertain Significance/Candidate causal | rs1595070689 | - | - | - | - | 394 |
| 166 | MYH7 | c.2163-1G>A | Chr14: 23895028 | NM_000257.4 | Candidate causal | rs606231334 | - | - | - | - | - |
| 167 | UBR1 | c.2839+5G>A | Chr15: 43314895 | NM_174916.3 | Uncertain Significance/Candidate causal | rs1596106023 | - | - | - | - | 563 |
| 168 | CCDC78 | c.61-1G>A | Chr16: 776086 | NM_001031737.3 | Uncertain Significance/Candidate causal | rs1006891646 | - | 0 | 0 | 0 | 564 |
| 169 | ACADVL | c.342+1G>C | Chr17: 7124150 | NM_000018.4 | Candidate causal | rs780020193 | - | 0 | 0 | 0 | - |
| 170 | ACADVL | c.623-1G>A | Chr17: 7125270 | NM_000018.4 | Candidate causal | rs1597526782 | - | - | - | - | - |
| 171 | ACADVL | c.753-2A>C | Chr17: 7125494 | NM_000018.4 | Candidate causal | rs398123092 | - | 0 | 0 | 0 | 565 |
| 172 | ACADVL | c.1269+1G>A | Chr17: 7127050 | NM_000018.4 | Candidate causal | rs773401248 | - | 0 | 0 | - | 450 |
| 173 | ACADVL | c.623-2_623-1del | Chr17: 7125269-7125270 | NM_000018.4 | Candidate causal | rs1555528265 | - | - | - | - | - |
| 174 | ACADVL | c.1606-2A>C | Chr17: 7127797 | NM_000018.4 | Candidate causal | rs113467582 | - | - | - | - | - |
| 175 | ACADVL | c.1434+2T>G | Chr17: 7127390 | NM_000018.4 | Candidate causal | rs1555528804 | - | - | - | - | - |
| 176 | ACADVL | c.1182+2T>C | Chr17: 7126558 | NM_000018.4 | Candidate causal | rs1555528635 | - | - | - | - | - |
| 177 | ACADVL | c.277+1del | Chr17: 7123995 | NM_000018.4 | Candidate causal | rs1555527741 | - | - | - | - | - |
| 178 | ACADVL | c.864del | Chr17: 7125607 | NM_000018.4 | Candidate causal | rs1555528386 | - | - | - | - | - |
| 179 | ACADVL | c.277+1del | Chr17: 7123995 | NM_000018.4 | Candidate causal | rs1555527741 | - | - | - | - | - |
| 180 | ACADVL | c.1606-1G>A | Chr17: 7127798 | NM_000018.4 | Candidate causal | rs1057517386 | - | - | - | - | - |
| 181 | ACADVL | c.1333-2A>T | Chr17: 7127285 | NM_000018.4 | Candidate causal | rs1057517280 | - | - | - | - | - |
| 182 | ACADVL | c.478-1G>C | Chr17: 7124856 | NM_000018.4 | Candidate causal | rs1057517130 | - | - | - | - | - |
| 183 | ACADVL | c.277+1G>T | Chr17: 7123996 | NM_000018.4 | Candidate causal | rs1057517012 | - | - | - | - | - |
| 184 | ACADVL | c.1183-1G>A | Chr17: 7126962 | NM_000018.4 | Candidate causal | rs1057516818 | - | - | - | - | - |
| 185 | ACADVL | c.1269+1del | Chr17: 7127049 | NM_000018.4 | Candidate causal | - | - | - | - | - | - |
| 186 | ACADVL | c.1533-2A>C | Chr17: 7127638 | NM_000018.4 | Candidate causal | rs996348255 | - | 0 | 0 | 0 | - |
| 187 | ACADVL | c.753-2A>G | Chr17: 7125494 | NM_000018.4 | Candidate causal | - | - | - | - | - | - |
| 188 | ACADVL | c.1752-3_1755del | Chr17: 7128124-7128130 | NM_000018.4 | Candidate causal | - | - | - | - | - | - |
| 189 | ACADVL | c.1605+2T>A | Chr17: 7127714 | NM_000018.4 | Candidate causal | - | - | - | - | - | - |
| 190 | ACADVL | c.1751+1G>A | Chr17: 7128034 | NM_000018.4 | Candidate causal | rs1567569262 | - | - | - | - | - |
| 191 | ACADVL | c.343-1G>A | Chr17: 7124242 | NM_000018.4 | Candidate causal | rs1555527877 | - | - | 0 | - | 566 |
| 192 | ACADVL | c.1077+1G>T | Chr17: 7126185 | NM_000018.4 | Candidate causal | rs140989450 | - | 0 | 0 | 0 | 460 |
| 193 | ACADVL | c.879-8T>A | Chr17: 7125978 | NM_000018.4 | Uncertain Significance/Candidate causal | - | - | - | - | - | 452 |
| 194 | ACADVL | c.62+6T>C | Chr17: 7123371 | NM_000018.4 | Uncertain Significance/Candidate causal | rs1555527495 | - | - | - | - | - |
| 195 | ACADVL | c.879-1G>A | Chr17: 7125985 | NM_000018.4 | Candidate causal | - | - | - | - | - | 451 |
| 196 | ACADVL | c.139-1G>T | Chr17: 7123782 | NM_000018.4 | Candidate causal | rs1597518019 | - | - | - | - | 451 |
| 197 | ACADVL | c.205-2A>G | Chr17: 7123921 | NM_000018.4 | Candidate causal | rs1597518954 | - | - | - | - | 451 |
| 198 | ACADVL | c.1751+2T>C | Chr17: 7128035 | NM_000018.4 | Candidate causal | rs1597539537 | - | - | - | - | 451 |
| 199 | ACADVL | c.138+1G>A | Chr17: 7123517 | NM_000018.4 | Candidate causal | rs747351687 | - | 0 | 0 | - | 454 |
| 200 | ACADVL | c.1532+2T>C | Chr17: 7127564 | NM_000018.4 | Candidate causal | rs111851815 | - | - | - | - | 454 |
| 201 | ACADVL | c.1752-2del | Chr17: 7128126 | NM_000018.4 | Candidate causal | rs1555529044 | - | - | - | - | 451 |
| 202 | ACADVL | c.138+2T>C | Chr17: 7123518 | NM_000018.4 | Candidate causal | rs1057516817 | - | - | 0 | - | 451 |
| 203 | ACADVL | c.878+1G>C | Chr17: 7125622 | NM_000018.4 | Candidate causal | rs757946752 | - | 0 | 0 | 0 | 456 |
| 204 | ACADVL | c.277+2T>G | Chr17: 7123997 | NM_000018.4 | Candidate causal | rs1555527745 | - | - | - | - | 567 |
| 205 | ACADVL | c.63-2A>C | Chr17: 7123439 | NM_000018.4 | Candidate causal | rs1555527513 | - | 0 | 0 | - | 458 |
| 206 | ACADVL | c.138+2dup | Chr17: 7123517-7123518 | NM_000018.4 | Uncertain Significance/Candidate causal | rs1555527548 | - | - | - | - | - |
| 207 | ACADVL | c.1183-15A>G | Chr17: 7126948 | NM_000018.4 | Likely Candidate causal | rs765390290 | - | 0 | 0 | - | 568 |
| 208 | ACADVL | c.278-1G>A | Chr17: 7124084 | NM_000018.4 | Candidate causal | rs1298004609 | - | 0 | 0 | - | 454 |
| 209 | ACADVL | c.1077+1G>A | Chr17: 7126185 | NM_000018.4 | Candidate causal | rs140989450 | - | 0 | 0 | 0 | 461 |
| 210 | ACADVL | c.1077+2T>C | Chr17: 7126186 | NM_000018.4 | Candidate causal | rs1057516370 | - | - | - | - | 461 |
| 211 | ACADVL | c.1182+1G>A | Chr17: 7126557 | NM_000018.4 | Candidate causal | rs113690956 | - | 0 | 0 | 0 | 458 |
| 212 | ACADVL | c.1679-6G>A | Chr17: 7127955 | NM_000018.4 | Likely Candidate causal | rs113994171 | - | 0 | 0 | 0 | 568 |
| 213 | ACADVL | c.62+1G>A | Chr17: 7123366 | NM_000018.4 | Candidate causal | - | - | - | - | - | 568 |
| 214 | ACADVL | c.1605+2T>C | Chr17: 7127714 | NM_000018.4 | Candidate causal | rs1597537351 | - | - | - | - | 451 |
| 215 | ITGB4 | c.3793+1G>A | Chr17: 73747193 | NM_000213.5 | Candidate causal | rs147222357 | - | 0 | 0 | 0 | 569 |
| 216 | DSG2 | c.523+1G>C | Chr18: 29101207 | NM_001943.5 | Candidate causal | rs553299589 | - | 0 | - | - | 245 |
| 217 | TNNI3 | c.24+2T>A | Chr19: 55668662 | NM_001943.5 | Candidate causal | rs777702465 | - | 0 | 0 | - | - |
| 218 | DMD | c.2843+9127del | ChrX: 31187659 | NM_004020.3 | Candidate causal | rs398123839 | - | - | - | - | 570 |
| 219 | DMD | c.2843+9102C>T | ChrX: 31187684 | NM_004020.3 | Candidate causal | - | - | - | - | - | 570 |
| 220 | DMD | c.2843+6294del | ChrX: 31190494 | NM_004020.3 | Candidate causal | rs1556028034 | - | - | - | - | 570 |
| 221 | DMD | c.2843+5106C>G | ChrX: 31191680 | NM_004020.3 | Candidate causal | - | - | - | - | - | 570 |
| 222 | DMD | c.1019+713del | ChrX: 31196073 | NM_004017.3 | Candidate causal | rs1556035795 | - | - | - | - | 570 |
| 223 | DMD | c.10224-175_10230del | ChrX: 31196081 | NM_004006.2 | Candidate causal | rs1556035817 | - | - | - | - | 571 |
| 224 | DMD | c.10223+1G>C | ChrX: 31196785 | NM_004006.2 | Candidate causal | rs398123834 | - | - | - | - | 570 |
| 225 | DMD | c.10063-1G>A | ChrX: 31196923 | NM_000109.4 | Candidate causal | rs1602417503 | - | - | - | - | 570 |
| 226 | DMD | c.10062+1G>T | ChrX: 31198486 | NM_000109.4 | Candidate causal | rs398123828 | - | - | - | - | 570 |
| 227 | DMD | c.9975-1G>A | ChrX: 31198599 | NM_004006.2 | Candidate causal | rs1556040444 | - | - | - | - | 572 |
| 228 | DMD | c.9975-2A>C | ChrX: 31198600 | NM_004006.2 | Candidate causal | rs886044502 | - | - | - | - | 573 |
| 229 | DMD | c.9974+175T>A | ChrX: 31200680 | NM_004006.2 | Uncertain Significance/Candidate causal | rs1602451773 | - | - | - | - | 570 |
| 230 | DMD | c.9784-2A>T | ChrX: 31201023 | NM_000109.4 | Candidate causal | rs1602456486 | - | - | - | - | 570 |
| 231 | DMD | c.9625+5G>C | ChrX: 31224694 | NM_000109.4 | Uncertain Significance/Candidate causal | rs1602695527 | - | - | - | - | 570 |
| 232 | DMD | c.9649+1G>A | ChrX: 31224698 | NM_004006.2 | Candidate causal | rs1556256329 | - | - | - | - | 570 |
| 233 | DMD | c.9540-2A>G | ChrX: 31224786 | NM_000109.4 | Candidate causal | rs1602696519 | - | - | - | - | 570 |
| 234 | DMD | c.9563+2T>A | ChrX: 68500735 | NM_004006.2 | Likely Candidate causal | rs746753722 | - | 0 | 0 | 0 | 574 |
| 235 | DMD | c.9539+1G>T | ChrX: 31227614 | NM_000109.4 | Candidate causal | rs886043989 | - | - | - | - | 570 |
| 236 | DMD | c.9563+1G>A | ChrX: 31227614 | NM_004006.2 | Candidate causal | rs886043989 | - | - | - | - | 572 |
| 237 | DMD | c.9361+1G>A | ChrX: 31241163 | NM_004006.2 | Candidate causal | rs398124094 | - | - | - | - | 575 |
| 238 | DMD | c.9224+1G>A | ChrX: 31341714 | NM_004006.2 | Candidate causal | rs1569526122 | - | - | - | - | 571 |
| 239 | DMD | c.8913+1G>C | ChrX: 31496222 | NM_000109.4 | Candidate causal | rs1556656077 | - | - | - | - | 570 |
| 240 | DMD | c.8645-2A>T | ChrX: 31496493 | NM_000109.4 | Candidate causal | rs794727770 | - | - | - | - | 570 |
| 241 | DMD | c.8548-1G>C | ChrX: 31497221 | NM_004006.2 | Candidate causal | rs1569546198 | - | - | - | - | 576 |
| 242 | DMD | c.8367-1G>A | ChrX: 31515062 | NM_000109.4 | Candidate causal | rs1603264731 | - | - | - | - | 570 |
| 243 | DMD | c.8390+2T>C | ChrX: 31525396 | NM_004006.2 | Candidate causal | rs863225013 | - | - | - | - | 570 |
| 244 | DMD | c.8027+2T>A | ChrX: 31676105 | NM_004006.2 | Candidate causal | rs863225010 | - | - | - | - | 570 |
| 245 | DMD | c.8003+1G>T | ChrX: 31676106 | NM_000109.4 | Candidate causal | rs1556789913 | - | - | - | - | 570 |
| 246 | DMD | c.8027+1G>A | ChrX: 31676106 | NM_004006.2 | Candidate causal | rs1556789913 | - | - | - | - | 577 |
| 247 | DMD | c.7848+2T>A | ChrX: 31697490 | NM_000109.4 | Candidate causal | rs1603445277 | - | - | - | - | 570 |
| 248 | DMD | c.7848+1G>A | ChrX: 31697491 | NM_000109.4 | Candidate causal | rs1603445278 | - | - | - | - | 570 |
| 249 | DMD | c.7661-2A>G | ChrX: 31697705 | NM_004006.2 | Candidate causal | rs1556806356 | - | - | - | - | 578 |
| 250 | DMD | c.7310-1G>A | ChrX: 31792310 | NM_004006.2 | Candidate causal | rs1556880354 | - | - | - | - | 570 |
| 251 | DMD | c.7285+1G>T | ChrX: 31838091 | NM_000109.4 | Candidate causal | rs398124044 | - | - | - | - | 570 |
| 252 | DMD | c.-113del | ChrX: 31838133 | NM_004013.2 | Candidate causal | rs1556917057 | - | - | - | - | 570 |
| 253 | DMD | c.7074+1G>A | ChrX: 31893304 | NM_000109.4 | Candidate causal | rs1556962223 | - | - | - | - | 570 |
| 254 | DMD | c.7098+1G>T | ChrX: 31893304 | NM_004006.2 | Candidate causal | rs1556962223 | - | - | - | - | 570 |
| 255 | DMD | c.-313_-312del | ChrX: 31893334 | NM_004013.2 | Candidate causal | rs1060502659 | - | - | - | - | 570 |
| 256 | DMD | c.-313del | ChrX: 31893335 | NM_004013.2 | Candidate causal | rs1603513680 | - | - | - | - | 570 |
| 257 | DMD | c.-333_-324del | ChrX: 31893346 | NM_004013.2 | Candidate causal | rs1603513697 | - | - | - | - | 570 |
| 258 | DMD | c.-352G>A | ChrX: 31893374 | NM_004013.2 | Candidate causal | - | - | - | - | - | 570 |
| 259 | DMD | c.-353G>A | ChrX: 31893375 | NM_004013.2 | Candidate causal | - | - | - | - | - | 570 |
| 260 | DMD | c.-354del | ChrX: 31893376 | NM_004013.2 | Uncertain Significance/Candidate causal | - | - | - | - | - | 570 |
| 261 | DMD | c.-395del | ChrX: 31893417 | NM_004013.2 | Candidate causal | rs398124040 | - | - | - | - | 579 |
| 262 | DMD | c.-426C>T | ChrX: 31893448 | NM_004013.2 | Candidate causal | rs128625230 | - | 0 | - | - | 570 |
| 263 | DMD | c.6889-1G>T | ChrX: 31893491 | NM_000109.4 | Candidate causal | rs1603514271 | - | - | - | - | 570 |
| 264 | DMD | c.6889-2A>T | ChrX: 31893492 | NM_000109.4 | Candidate causal | rs1603514272 | - | - | - | - | 570 |
| 265 | DMD | c.-591C>T | ChrX: 31947835 | NM_004013.2 | Candidate causal | rs128626252 | - | 0 | - | - | 570 |
| 266 | DMD | c.-621A>T | ChrX: 31950199 | NM_004013.2 | Candidate causal | - | - | - | - | - | 570 |
| 267 | DMD | c.-627C>T | ChrX: 31950205 | NM_004013.2 | Candidate causal | - | - | - | - | - | 570 |
| 268 | DMD | c.-719del | ChrX: 31950297 | NM_004013.2 | Candidate causal | rs1060502620 | - | - | - | - | 570 |
| 269 | DMD | c.6615-2A>T | ChrX: 31950346 | NM_004006.2 | Candidate causal | rs1557011973 | - | - | - | - | 570 |
| 270 | DMD | c.6614+2T>C | ChrX: 31986454 | NM_004006.2 | Candidate causal | rs1060502641 | - | - | - | - | 570 |
| 271 | DMD | c.-771_-770del | ChrX: 31986459 | NM_004013.2 | Candidate causal | - | - | - | - | - | 570 |
| 272 | DMD | c.6251_6290+12981del | ChrX: 32292665 | NM_004006.2 | Candidate causal | - | - | - | - | - | - |
| 273 | DMD | c.6118-1G>A | ChrX: 32305819 | NM_004006.2 | Candidate causal | rs1060502656 | - | - | - | - | 570 |
| 274 | DMD | c.4518+5G>A | ChrX: 32407613 | NM_004006.2 | Uncertain Significance | rs398123960 | - | - | - | - | 570 |
| 275 | DMD | c.4071+1G>A | ChrX: 32456357 | NM_004006.2 | Candidate causal | rs1060502643 | - | - | - | - | 570 |
| 276 | DMD | c.3604-1G>C | ChrX: 32466756 | NM_004006.2 | Candidate causal | rs1569562952 | - | - | - | - | 580 |
| 277 | DMD | c.3603+3A>T | ChrX: 32472776 | NM_004006.2 | Uncertain Significance/Candidate causal | rs1060502615 | - | - | - | - | 581 |
| 278 | DMD | c.3579+2T>A | ChrX: 32472777 | NM_000109.4 | Candidate causal | rs146071084 | - | 0 | - | - | 570 |
| 279 | DMD | c.3603+2T>G | ChrX: 32472777 | NM_004006.2 | Candidate causal | rs146071084 | - | - | - | - | 570 |
| 280 | DMD | c.3433-5_3434del | ChrX: 32472948 | NM_004006.2 | Candidate causal | rs863224994 | - | - | - | - | 570 |
| 281 | DMD | c.3432+1G>A | ChrX: 32481555 | NM_004006.2 | Candidate causal | rs398123937 | - | - | - | - | 570 |
| 282 | DMD | c.2926-2A>G | ChrX: 32486829 | NM_000109.4 | Candidate causal | rs398123915 | - | - | - | - | 570 |
| 283 | DMD | c.2949+1G>A | ChrX: 32490280 | NM_004006.2 | Candidate causal | rs1557374482 | - | - | - | - | 571 |
| 284 | DMD | c.2804-1del | ChrX: 32490427 | NM_004006.2 | Candidate causal | rs1557374667 | - | - | - | - | 572 |
| 285 | DMD | c.2804-1G>T | ChrX: 32490427 | NM_004006.2 | Candidate causal | rs398123909 | - | - | - | - | 549 |
| 286 | DMD | c.2804-2A>C | ChrX: 32490428 | NM_004006.2 | Candidate causal | rs794727357 | - | - | - | - | 572 |
| 287 | DMD | c.2623-3C>G | ChrX: 32503219 | NM_004006.2 | Uncertain Significance | rs863224988 | - | - | 0 | - | 570 |
| 288 | DMD | c.2598+1G>T | ChrX: 32509393 | NM_000109.4 | Candidate causal | rs398123901 | - | - | - | - | 570 |
| 289 | DMD | c.2380+3A>C | ChrX: 32519869 | NM_004006.2 | Uncertain Significance | rs1569564916 | - | - | - | - | 549 |
| 290 | DMD | c.2356+1G>A | ChrX: 32519871 | NM_000109.4 | Candidate causal | rs398123884 | - | - | - | - | 570 |
| 291 | DMD | c.2145-1G>A | ChrX: 32536249 | NM_000109.4 | Candidate causal | - | - | - | - | - | 570 |
| 292 | DMD | c.2168+1G>A | ChrX: 32563275 | NM_004006.2 | Candidate causal | rs1057518207 | - | - | - | - | 570 |
| 293 | DMD | c.1992+1G>T | ChrX: 32583818 | NM_004006.2 | Candidate causal | rs1556802319 | - | - | - | - | 571 |
| 294 | DMD | c.1812+1G>A | ChrX: 32591646 | NM_004006.2 | Candidate causal | rs373286166 | - | 0.2 | 0 | 0 | 582 |
| 295 | DMD | c.1705-1G>T | ChrX: 32591755 | NM_004006.2 | Candidate causal | rs878854619 | - | - | - | - | 570 |
| 296 | DMD | c.1705-2A>G | ChrX: 32591756 | NM_004006.2 | Candidate causal | rs1601809011 | - | - | - | - | 571 |
| 297 | DMD | c.1483-1G>C | ChrX: 32613994 | NM_004006.2 | Candidate causal | rs863224982 | - | - | - | - | 581 |
| 298 | DMD | c.1482+1G>T | ChrX: 32632419 | NM_004006.2 | Candidate causal | rs398123862 | - | 0 | - | - | 583 |
| 299 | DMD, MIR548F5 | c.1307_1332-7556del | ChrX: 32640126 | NM_004006.2 | Candidate causal | - | - | - | - | - | - |
| 300 | DMD | c.1331+1G>A | ChrX: 32662248 | NM_004006.2 | Candidate causal | rs863224980 | - | - | - | - | 570 |
| 301 | DMD | c.1126-2A>T | ChrX: 32662432 | NM_000109.4 | Candidate causal | - | - | - | - | - | 570 |
| 302 | DMD | c.1150-2del | ChrX: 32662432 | NM_004006.2 | Candidate causal | rs863224978 | - | - | - | - | 584 |
| 303 | DMD | c.937-1G>A,C | ChrX: 32663270 | NM_000109.4 | Candidate causal | - | - | - | - | - | 570 |
| 304 | DMD | c.831+1G>A | ChrX:32717228 | NM_000109.4 | Candidate causal | rs1239018406 | - | - | - | - | - |
| 305 | DMD | c.650-1828_770del | ChrX: 32717290 | NM_004006.2 | Candidate causal | - | - | - | - | - | - |
| 306 | DMD | c.531-1G>C | ChrX: 32827729 | NM_004006.2 | Candidate causal | rs1569526579 | - | - | - | - | 571 |
| 307 | DMD | c.358-2A>G | ChrX: 32834759 | NM_004006.2 | Candidate causal | rs863224996 | - | - | - | - | 571 |
| 308 | DMD | c.-14del | ChrX: 32841413 | NM_004010.3 | Candidate causal | rs1557058308 | - | - | - | - | 570 |
| 309 | DMD | c.-76_-64del | ChrX: 32841463 | NM_004010.3 | Candidate causal | rs1603441550 | - | - | - | - | 570 |
| 310 | DMD | c.265-2A>G | ChrX: 32841506 | NM_004006.2 | Candidate causal | rs863224989 | - | - | - | - | 570 |
| 311 | DMD | c.241-463A>G | ChrX: 32841967 | NM_000109.4 | Uncertain Significance/Candidate causal | - | - | - | - | - | 570 |
| 312 | DMD | c.240+5G>A | ChrX: 32862895 | NM_000109.4 | Uncertain Significance/Candidate causal | - | - | - | - | - | 570 |
| 313 | DMD | c.-117C>T | ChrX: 32862911 | NM_004010.3 | Candidate causal | rs128626234 | - | 0 | - | - | 570 |
| 314 | DMD | c.187-1G>T | ChrX: 32862978 | NM_004006.2 | Candidate causal | rs1603447081 | - | - | - | - | 571 |
| 315 | DMD | c.186+1G>A | ChrX: 32867844 | NM_004006.2 | Candidate causal | rs1557084067 | - | - | - | - | 571 |
| 316 | DMD | c.-209T>G | ChrX: 32867870 | NM_004010.3 | Uncertain Significance/Candidate causal | rs128626231 | - | - | - | - | 570 |
| 317 | DMD | c.69_69+14del | ChrX: 33038242 | NM_000109.4 | Candidate causal | - | - | - | - | - | 570 |
| 318 | DMD | c.69+1G>T | ChrX: 33038255 | NM_000109.4 | Candidate causal | - | - | - | - | - | 570 |
| 319 | DMD | c.93+1G>A | ChrX: 33038255 | NM_004006.2 | Candidate causal | rs886042604 | - | - | - | - | 570 |
| 320 | DMD | c.-312del | ChrX: 33038291 | NM_004010.3 | Candidate causal | rs1602766499 | - | - | - | - | 570 |
| 321 | DMD | c.-317del | ChrX: 33038296 | NM_004010.3 | Candidate causal | rs1602766531 | - | - | - | - | 570 |
| 322 | DMD | c.-331del | ChrX: 33038310 | NM_004010.3 | Candidate causal | rs1602766655 | - | - | - | - | 570 |
| 323 | DMD | c.-336_-332del | ChrX: 33038311 | NM_004010.3 | Candidate causal | rs1602766725 | - | - | - | - | 570 |
| 324 | DMD | c.7+127982G>A | ChrX: 33229394 | NM_000109.4 | Uncertain Significance/Candidate causal | rs1403865104 | - | - | - | - | 570 |
| 325 | DMD | c.7+127978del | ChrX: 33229398 | NM_000109.4 | Candidate causal | - | - | - | - | - | 570 |
| 326 | DMD | c.31+1G>T | ChrX: 33229398 | NM_004006.2 | Candidate causal | rs398123923 | - | - | - | - | 572 |
| 327 | DMD | c.7+127975del | ChrX: 33229401 | NM_000109.4 | Candidate causal | - | - | - | - | - | 570 |
| 328 | DMD | c.7+127965del | ChrX: 33229411 | NM_000109.4 | Candidate causal | rs1557300135 | - | - | - | - | 570 |
| 329 | DMD | c.4345-3C>G | ChrX: 32407794 | NM_000109.4 | Uncertain Significance/Candidate causal | - | - | - | - | - | - |
| 330 | DMD | c.93+1G>C | ChrX: 33038255 | NM_000109.4 | Candidate causal | rs886042604 | - | - | - | - | - |
| 331 | DMD | c.-193del | ChrX: 32867854 | NM_000109.4 | Candidate causal | rs1057518834 | - | - | - | - | - |
| 332 | DMD | c.10086+1G>A | ChrX: 31198486 | NM_000109.4 | Candidate causal | rs398123828 | - | - | - | - | 585 |
| 333 | DMD | c.5448+1G>T | ChrX: 32366522 | NM_000109.4 | Candidate causal | - | - | - | - | - | - |
| 334 | DMD | c.8524-1G>C | ChrX:31497221 | NM_000109.4 | Candidate causal | rs1569546198 | - | - | - | - | 586 |
| 335 | DMD | c.2356+3A>C | ChrX: 32519869 | NM_000109.4 | Uncertain Significance | rs1569564916 | - | - | - | - | 587 |
| 336 | DMD | c.31+1G>C | ChrX: 33229398 | NM_004006.2 | Candidate causal | rs398123923 | - | - | - | - | 588 |
| 337 | DMD | c.8548-2A>G | ChrX: 31497222 | NM_004006.3 | Candidate causal | - | - | - | - | - | - |
| 338 | DMD | c.31+1del | ChrX: 33229398 | NM_004006.3 | Candidate causal | rs1603419074 | - | - | - | - | - |
| 339 | DMD | c.31+5G>A | ChrX: 33229394 | NM_004006.3 | Uncertain Significance/Candidate causal | rs1403865104 | - | - | - | - | - |
| 340 | DMD | c.93+1G>T | ChrX: 33038255 | NM_004006.3 | Candidate causal | rs886042604 | - | - | - | - | - |
| 341 | DMD | c.93_93+14del | ChrX: 33038242-33038256 | NM_004006.3 | Candidate causal | rs1602765633 | - | - | - | - | - |
| 342 | DMD | c.264+5G>A | ChrX: 32862895 | NM_004006.3 | Uncertain Significance | rs398123902 | - | - | - | - | - |
| 343 | DMD | c.265-463A>G | ChrX: 32841967 | NM_004006.3 | Uncertain Significance/Candidate causal | rs1603441629 | - | - | - | - | 589 |
| 344 | DMD | c.961-1G>A | ChrX: 32663270 | NM_004006.3 | Candidate causal | rs1602469384 | - | - | - | - | 590 |
| 345 | DMD | c.1150-2A>T | ChrX: 32662432 | NM_004006.3 | Candidate causal | rs794727030 | - | - | - | - | - |
| 346 | DMD | c.2169-1G>A | ChrX: 32536249 | NM_004006.3 | Candidate causal | rs1603635331 | - | - | - | - | - |
| 347 | DMD | c.2287_2292+12del | ChrX: 32536113-32536130 | NM_004006.3 | Candidate causal | rs1603635326 | - | - | - | - | - |
| 348 | DMD | c.2380+1G>A | ChrX: 32519871 | NM_004006.3 | Candidate causal | rs398123884 | - | - | - | - | - |
| 349 | DMD | c.2622+1G>T | ChrX: 32509393 | NM_004006.3 | Candidate causal | rs398123901 | - | - | - | - | - |
| 350 | DMD | c.2803+5G>C | ChrX: 32503031 | NM_004006.3 | Uncertain Significance/Candidate causal | rs1603634603 | - | - | - | - | 591 |
| 351 | DMD | c.6913-2A>T | ChrX: 31893492 | NM_004006.3 | Candidate causal | rs1603514272 | - | - | - | - | - |
| 352 | DMD | c.6913-1G>T | ChrX: 31893491 | NM_004006.3 | Candidate causal | rs1603514271 | - | - | - | - | 592 |
| 353 | DMD | c.7098+1G>A | ChrX: 31893304 | NM_004006.3 | Candidate causal | rs1556962223 | - | - | - | - | 593 |
| 354 | DMD | c.7309+1G>T | ChrX: 31838091 | NM_004006.3 | Candidate causal | rs398124044 | - | - | - | - | - |
| 355 | DMD | c.7309+1G>C | ChrX: 31838091 | NM_004006.3 | Candidate causal | rs398124044 | - | - | - | - | - |
| 356 | DMD | c.7872+1G>A | ChrX: 31697491 | NM_004006.3 | Candidate causal | rs1603445278 | - | - | - | - | - |
| 357 | DMD | c.7872+2T>A | ChrX: 31697490 | NM_004006.3 | Candidate causal | rs1603445277 | - | - | - | - | - |
| 358 | DMD | c.8027+1G>T | ChrX: 31676106 | NM_004006.3 | Candidate causal | rs1556789913 | - | - | - | - | - |
| 359 | DMD | c.8391-1G>A | ChrX: 31515062 | NM_004006.3 | Candidate causal | rs1603264731 | - | - | - | - | - |
| 360 | DMD | c.8669-2A>T | ChrX: 31496493 | NM_004006.3 | Candidate causal | rs794727770 | - | - | - | - | 594 |
| 361 | DMD | c.8937+1G>C | ChrX: 31496222 | NM_004006.3 | Candidate causal | rs1556656077 | - | - | - | - | - |
| 362 | DMD | c.9564-2A>G | ChrX: 31224786 | NM_004006.3 | Candidate causal | rs1602696519 | - | - | - | - | - |
| 363 | DMD | c.9649+2dup | ChrX: 31224696-31224697 | NM_004006.3 | Uncertain Significance/Candidate causal | rs1602695597 | - | - | - | - | - |
| 364 | DMD | c.9649+5G>C | ChrX: 31224694 | NM_004006.3 | Uncertain Significance/Candidate causal | rs1602695527 | - | - | - | - | - |
| 365 | DMD | c.9808-2A>T | ChrX: 31201023 | NM_004006.3 | Candidate causal | rs1602456486 | - | - | - | - | - |
| 366 | DMD | c.10087-1G>A | ChrX: 31196923 | NM_004006.3 | Candidate causal | rs1602417503 | - | - | - | - | - |
| 367 | DMD | c.10224-1dup | ChrX: 31196087-31196088 | NM_004006.3 | Candidate causal | rs1602409174 | - | - | - | - | - |
| 368 | DMD | c.94_264dup | ChrX: 31496222 | NM_004006.3 | Uncertain Significance | - | - | - | - | - | - |
| 369 | DMD | c.4675-1G>A | ChrX: 32398798 | NM_004006.3 | Candidate causal | - | - | - | - | - | - |
| 370 | DMD | c.4519-2A>C | ChrX: 32404584 | NM_004006.3 | Candidate causal | - | - | - | - | - | - |
| 371 | DMD | c.6615-2A>G | ChrX: 31950346 | NM_004006.3 | Candidate causal | - | - | - | - | - | 595 |
| 372 | DMD | c.2381-2A>C | ChrX: 32509637 | NM_004006.3 | Candidate causal | rs1603634749 | - | - | - | - | - |
| 373 | DMD | c.5922+1G>T | ChrX: 32360216 | NM_004006.3 | Candidate causal | rs1603631219 | - | - | - | - | - |
| 374 | DMD | c.2843+6256_2843+6260dup | ChrX: 31190525-31190526 | NM_004006.3 | Candidate causal | rs1602360475 | - | - | - | - | - |
| 375 | DMD | c.9563+1G>T | ChrX: 31227614 | NM_004006.3 | Candidate causal | rs886043989 | - | - | - | - | - |
| 376 | DMD | c.10395-1G>A | ChrX: 31187719 | NM_004006.3 | Candidate causal | rs1602338283 | - | - | - | - | - |
| 377 | DMD | c.7309+12789_7490del | ChrX: 31792129-31825303 | NM_004006.2 | Candidate causal | - | - | - | - | - | - |
| 378 | DMD | c.2292+2T>G | ChrX: 32536123 | NM_004006.2 | Candidate causal | rs1557396600 | - | - | - | - | - |
| 379 | DMD | c.6889-11_6894del | ChrX: 31893485-31893501 | NM_000109.4 | Candidate causal | rs1556962571 | - | - | - | - | - |
| 380 | DMD | c.9287-27_9287-2del | ChrX: 31241240-31241265 | NM_004006.2 | Candidate causal | rs1064792968 | - | - | - | - | - |
| 381 | DMD | c.9626-4_9631del | ChrX: 31222230-31222239 | NM_000109.4 | Candidate causal | rs1060502653 | - | - | - | - | - |
| 382 | DMD | c.-132dup | ChrX: 32862925-32862926 | NM_004006.3 | Candidate causal | rs1557079469 | - | - | - | - | - |
| 383 | DMD | c.-217dup | ChrX: 32867877-32867878 | NM_004006.3 | Candidate causal | rs1557084128 | - | - | - | - | - |
| 384 | DMD | c.-140_-138delinsGTTT | ChrX: 31838158-31838160 | NM_004013.2 | Candidate causal | rs1064792967 | - | - | - | - | - |
| 385 | DMD | c.4072-1G>T | ChrX: 32430031 | NM_004006.2 | Candidate causal | rs1060502637 | - | - | - | - | - |
| 386 | DMD | c.8367-1_8367delinsAA | ChrX: 31515061-31515062 | NM_000109.4 | Candidate causal | rs1060502619 | - | - | - | - | - |
| 387 | DMD | c.832-15A>G | ChrX: 32716130 | NM_004006.2 | Uncertain Significance/Candidate causal | rs72470513 | - | - | - | - | 596 |
| 388 | DMD | c.2843+9182_2843+9183del | ChrX: 31187603-31187604 | NM_004006.3 | Candidate causal | rs878854366 | - | - | - | - | 597 |
| 389 | DMD | c.3579+2T>G | ChrX: 32472777 | NM_000109.4 | Candidate causal | rs146071084 | - | 0 | - | - | 598 |
| 390 | DMD | c.9563+5G>C | ChrX: 31227610 | NM_004006.3 | Uncertain Significance/Candidate causal | - | - | - | - | - | 599 |
| 391 | DMD | c.3921+1_3921+2dup | ChrX: 32459294-32459295 | NM_004006.3 | Uncertain Significance/Candidate causal | - | - | - | - | - | 600 |
| 392 | DMD | c.-229dup | ChrX: 32867889-32867890 | NM_004006.3 | Candidate causal | rs1569533965 | - | - | - | - | 601 |
| 393 | DMD | c.8547+1G>A | ChrX: 31514904 | NM_004006.2 | Candidate causal | rs1556665052 | - | - | - | - | 602 |
| 394 | DMD | c.1019+732dup | ChrX: 31196052-31196053 | NM_004006.3 | Candidate causal | rs398123835 | - | - | - | - | - |
| 395 | DMD | c.2949+2T>C | ChrX: 32490279 | NM_004006.3 | Candidate causal | - | - | - | - | - | 602 |
| 396 | DMD | c.9807+2T>G | ChrX: 31222076 | NM_004006.3 | Candidate causal | - | - | - | - | - | 602 |
| 397 | DMD | c.8666_8668+3del | ChrX: 31497097-31497102 | NM_004006.3 | Candidate causal | - | - | - | - | - | 600 |
| 398 | DMD | c.832-2A>C | ChrX: 32716117 | NM_004006.3 | Candidate causal | - | - | - | - | - | 602 |
| 399 | DMD | c.650-1G>A | ChrX: 32717411 | NM_004006.3 | Candidate causal | - | - | - | - | - | 602 |
| 400 | DMD | c.4846-1G>T | ChrX: 32383317 | NM_004006.2 | Candidate causal | rs1603631757 | - | - | - | - | 602 |
| 401 | DMD | c.8028-3C>T | ChrX: 31645982 | NM_004006.2 | Uncertain Significance | rs1603430208 | - | 0 | - | 0 | - |
| 402 | DMD | c.2293-8T>C | ChrX: 32519967 | NM_004006.2 | Uncertain Significance | rs377050013 | - | 0 | 0 | 0 | - |
| 403 | DMD | c.3432+10A>G | ChrX: 32430031 | NM_004006.3 | Uncertain Significance | rs746260475 | - | 0 | 0 | 0 | - |
| 404 | DMD | c.9807+5G>A | ChrX: 31222073 | NM_004006.2 | Uncertain Significance/Candidate causal | rs1569460607 | - | - | - | - | 603 |
| 405 | DMD | c.5154+2T>C | ChrX: 32382697 | NM_004006.2 | Candidate causal | rs1569559822 | - | - | - | - | 604 |
| 406 | DMD | c.4675-11A>G | ChrX: 32398808 | NM_004006.2 | Uncertain Significance | rs1557316295 | - | - | - | - | 589 |
| 407 | DMD | c.8367-2A>G | ChrX: 31515063 | NM_000109.4 | Candidate causal | rs1556665303 | - | - | - | - | - |
| 408 | DMD | c.6762+3A>T | ChrX: 31950194 | NM_004006.2 | Uncertain Significance | rs1085307811 | - | - | - | - | - |
| 409 | DMD | c.5715+1G>T | ChrX: 32361250 | NM_000109.4 | Candidate causal | rs886043041 | - | 0 | 0 | 0 | - |
| 410 | DMD | c.6614+3310G>T | ChrX: 31983146 | NM_004006.2 | Uncertain Significance/Candidate causal | rs797045526 | - | - | - | - | 605 |
| 411 | DMD | c.-395dup | ChrX: 31893416-31893417 | NM_004006.2 | Candidate causal | rs398124040 | - | - | - | - | 600 |
| 412 | DMD | c.3162+1G>A | ChrX: 32486614 | NM_004006.3 | Candidate causal | - | - | - | - | - | 601 |
| 413 | DMD | c.3277-2A>T | ChrX: 32481713 | NM_004006.3 | Candidate causal | - | - | - | - | - | 606 |
| 414 | DMD | c.10797+2T>A | ChrX: 31165390 | NM_004006.3 | Candidate causal | - | - | - | - | - | 607 |
| 415 | DMD | c.5026-2A>G | ChrX: 32382829 | NM_004006.2 | Candidate causal | rs1569559849 | - | - | - | - | 602 |
| 416 | DMD | c.10797+1G>A | ChrX: 31165391 | NM_004006.2 | Candidate causal | rs1060502626 | - | - | - | - | 607 |
| 417 | DMD | c.10797+5G>A | ChrX: 31165387 | NM_004006.2 | Likely Candidate causal | rs398123846 | - | - | - | - | 608 |
| 418 | DMD | c.3603+2T>C | ChrX: 32472777 | NM_004006.3 | Candidate causal | - | - | - | - | - | 600 |
| 419 | DMD | c.3579+2dup | ChrX: 32472776-32472777 | NM_000109.4 | Uncertain Significance/Candidate causal | rs796523999 | - | - | - | - | 609 |
| 420 | DMD | c.7661-1G>A | ChrX: 31697704 | NM_004006.3 | Candidate causal | - | - | - | - | - | 583 |
| 421 | DMD | c.187-2A>G | ChrX: 32862979 | NM_004006.3 | Candidate causal | - | - | - | - | - | - |
| 422 | DMD | c.6290+2T>A | ChrX: 32305644 | NM_004006.3 | Candidate causal | - | - | - | - | - | 610 |
| 423 | DMD | c.831+1G>T | ChrX: 32717228 | NM_004006.2 | Candidate causal | rs1239018406 | - | - | - | - | 611 |
| 424 | DMD | c.3603+1G>T | ChrX: 32472778 | NM_004006.2 | Candidate causal | rs1209389771 | - | 0 | - | - | 611 |
| 425 | DMD | c.1149+1G>A | ChrX: 32663080 | NM_004006.2 | Candidate causal | rs1060502634 | - | - | - | - | 607 |
| 426 | DMD | c.961-5831C>T | ChrX: 32669100 | NM_004006.2 | Uncertain Significance/Candidate causal | rs398124099 | - | - | - | - | 612 |
| 427 | DMD | c.531-10T>A | ChrX: 32827738 | NM_004006.2 | Uncertain Significance | rs747522183 | - | - | 0 | - | - |
| 428 | DMD | c.2780-1del | ChrX: 32490427 | NM_000109.4 | Candidate causal | rs1557374667 | - | - | - | - | 602 |
| 429 | DMD | c.2804-1G>C | ChrX: 32490427 | NM_004006.3 | Candidate causal | - | - | - | - | - | 609 |
| 430 | DMD | c.3432+2036A>G | ChrX: 32479520 | NM_004006.2 | Likely Candidate causal | rs182575709 | - | 0 | 0 | 0 | 613 |
| 431 | DMD | c.8390+1G>A | ChrX: 31525397 | NM_004006.2 | Candidate causal | rs1064794569 | - | - | - | - | 578 |
| 432 | DMD | c.358-1G>A | ChrX: 32834758 | NM_004006.2 | Candidate causal | rs886044582 | - | - | - | - | 600 |
| 433 | DMD | c.9225-647A>G | ChrX: 31279780 | NM_004006.2 | Likely Candidate causal | rs398124091 | - | - | - | - | 612 |
| 434 | DMD | c.2804-1G>A | ChrX: 32490427 | NM_004006.2 | Candidate causal | rs398123909 | - | - | - | - | 606 |
| 435 | DMD | c.2622+1G>A | ChrX: 32509393 | NM_004006.2 | Candidate causal | rs398123901 | - | - | - | - | 606 |
| 436 | DMD | c.9225-285A>G | ChrX: 31279418 | NM_000109.4 | Likely Candidate causal | rs587776747 | - | - | - | - | 613 |
| 437 | DMD | c.3603+2_3603+3insTA | ChrX: 32472776-32472777 | NM_004006.3 | Uncertain Significance/Candidate causal | rs1603633860 | - | - | - | - | 609 |
| 438 | DMD | c.1704+1G>A | ChrX: 32591861 | NM_004006.2 | Candidate causal | rs794727123 | - | - | - | - | 606 |
| 439 | XK | c.508+1G>A | ChrX: 37553802 | NM_021083.4 | Candidate causal | - | - | - | - | - | - |
| 440 | XK | c.509-1G>A | ChrX: 37586888 | NM_021083.4 | Candidate causal | - | - | - | - | - | - |
| 441 | DNASE1L1, TAFAZZIN | c.110-2A>G | ChrX: 153640421 | NM_000116.5 | Candidate causal | rs1603376833 | - | - | - | - | - |
| 442 | TAFAZZIN | c.239-1G>C | ChrX: 153641543 | NM_000116.5 | Candidate causal | rs1603377590 | - | - | - | - | - |
| 443 | TAFAZZIN | c.239-1G>A | ChrX: 153641543 | NM_000116.5 | Candidate causal | rs1603377590 | - | - | - | - | - |
| 444 | TAFAZZIN | c.284+110G>A | ChrX: 153641699 | NM_000116.5 | Candidate causal | rs160337774 | - | - | - | - | - |
| 445 | TAFAZZIN | c.647-1G>C | ChrX: 153648550 | NM_000116.5 | Candidate causal | rs587776741 | - | - | - | - | - |
| 446 | TAFAZZIN | c.700-1G>A | ChrX: 153648996 | NM_000116.5 | Candidate causal | rs397515747 | - | - | - | - | 614 |
| 447 | ACAD8 | c.842-2A>T | Chr11: 134131167 | NM_014384.3 | Candidate causal | - | - | - | - | - | - |
| 448 | BAG3 | c.909+1G>A | Chr10: 121432169 | NM_004281.3 | Candidate causal | rs869025365 | - | - | - | - | - |
| 449 | CRYAB | c.325-2A>G | Chr11: 111779693 | NM_001289808.2 | Uncertain Significance/Candidate causal | rs202024436 | - | 0 | 0 | 0 | - |
| 450 | DSC2 | c.2125+1del | Chr18: 28651570 | NM_024422.6 | Candidate causal | rs794728072 | - | 0 | - | 0 | 615 |
| 451 | GJD2-DT|ACTC1 | c.-36C>G | Chr15: 35087720 | NM_005159.5 | Uncertain Significance | rs886051091 | - | 0 | 0 | 0 | - |
| 452 | GLB1 | c.1480-2A>G | Chr3: 33055804 | NM_000404.4 | Candidate causal | rs587776526 | - | 0 | 0 | 0 | 616 |
| 453 | LAMP2 | c.864+3_864+6del | ChrX: 119580154-119580157 | NM_013995.2 | Likely Candidate causal | rs397516751 | - | - | - | - | 507 |
| 454 | LAMP2 | c.864+5G>C | ChrX: 119580155 | NM_013995.2 | Uncertain Significance | rs1352584474 | - | - | - | - | 506 |
| 455 | LAMP2 | c.929-1G>A | ChrX: 119575750 | NM_002294.3 | Candidate causal | rs727504262 | - | - | - | - | 505 |
| 456 | LAMP2 | c.864+1G>T | ChrX: 119580159 | NM_002294.3 | Candidate causal | rs727503119 | - | - | - | - | - |
| 457 | LAMP2 | c.741+1G>A | ChrX: 119581695 | NM_013995.2 | Candidate causal | rs1251075016 | - | - | - | - | 506 |
| 458 | LAMP2 | c.65-1G>C | ChrX: 119590625 | NM_013995.2 | Candidate causal | rs730880496 | - | - | - | - | 504 |
| 459 | LAMP2 | c.65-2A>G | ChrX: 119590626 | NM_002294.3 | Candidate causal | rs397516743 | - | 0 | - | - | 507 |
| 460 | LAMP2 | c.64+1G>C | ChrX: 119602960 | NM_013995.3 | Candidate causal | - | - | - | - | - | 507 |
| 461 | LAMP2 | c.1093+1G>A | ChrX: 119575584 | NM_013995.3 | Candidate causal | rs727504742 | - | - | - | - | - |
| 462 | MYPN | c.2704-1G>A | Chr10: 69948661 | NM_032578.4 | Candidate causal | rs865921466 | - | 0 | - | - | - |
| 463 | MYPN | c.1246-2A>G | Chr10: 69909795 | NM_032578.4 | Candidate causal | - | - | - | - | - | 617 |
| 464 | NEBL | c.358-27884del | Chr10: 21129753 | NM_213569.2 | Uncertain Significance/Candidate causal | rs768079285 | - | - | 0 | - |  |
| 465 | SCN5A | c.612-229T>G | Chr3: 38655554 | NM_198056.2 | Candidate causal | rs765669597 | - | 0 | 0 | - | - |
| 466 | SCN5A | c.4437+5G>A | Chr3: 38597927 | NM_198056.2 | Uncertain Significance | rs1057520531 | - | - | - | - | 618 |
| 467 | SGCD | c.699+1G>T | Chr5: 156184716 | NM_000337.5 | Candidate causal | rs1554137130 | - | - | - | - | - |
| 468 | SGCD | c.192+1G>A | Chr5: 155771688 | NM_000337.5 | Candidate causal | rs1267810339 | - | - | 0 | - | - |
| 469 | SGCD | c.294+1G>A | Chr5: 155935713 | NM_000337.5 | Candidate causal | rs727503422 | - | 0 | - | 0 | - |
| 470 | SGCD | c.4-1G>A | Chr5: 155771498 | NM_000337.5 | Candidate causal | rs1554094927 | - | 0 | 0 | 0 | 619 |
| 471 | TNNT2 | c.490-1G>C | Chr1: 201332535 | NM_001276345.2 | Candidate causal | rs111344408 | - | 0 | 0 | 0 | - |
| 472 | VCL | c.1544-2A>G | Chr10: 75855412 | NM_014000.3 | Candidate causal | rs727503738 | - | - | - | - | - |
| 473 | FLNC | c.4127+1delG | Chr7:128486517 | NM_001458.5 | Candidate causal | - | - | - | - | - | - |
| 474 | FLNC | c.5539+1G>C | Chr7:128486517 | NM_001458.5 | Candidate causal | - | - | - | - | - | - |
| 475 | FLNC | c.3791-1G>A | Chr7: 128486043 | NM_001458.5 | Candidate causal | - | - | - | - | - | - |
| 476 | FLNC | c.3965-2A>T | Chr7:128486353 | NM_001458.5 | Candidate causal | - | - | - | -- | - | - |

Table 4. The candidate causal/likely candidate causal non-coding variants in Arrhythmogenic right ventricular cardiomyopathy (ARVC).

| No. | Gene | Variant | Genomic location (hg19) | Transcript | Classification | dbSNP | Frequency of variants (%) | | | | Ref. |
| --- | --- | --- | --- | --- | --- | --- | --- | --- | --- | --- | --- |
|  |  |  |  |  |  |  | **Iranome** | **1000 Genome** | **genomAD** | **TOPMED** |  |
| 1 | PKP2 | c.2489+1G>T | Chr12: 32949042 | NM_004572.4 | Candidate causal | rs111517471 | - | 0 | 0 | 0 | 620 |
| 2 | PKP2 | c.2357+1G>A | Chr12: 32949042 | NM_001005242.3 | Candidate causal | rs111517471 | - | 0 | 0 | 0 | 621 |
| 3 | PKP2 | c.2014-1G>C | Chr12: 32955491 | NM_001005242.3 | Candidate causal | rs193922674 | - | 0 | 0 | 0 | 444 |
| 4 | PKP2 | c.1689-1G>C | Chr12: 32977097 | NM_004572.4 | Candidate causal | rs78897684 | - | 0 | 0 | - | 622 |
| 5 | PKP2 | c.1511-2A>T | Chr12: 32994141 | NM_004572.4 | Candidate causal | rs1453983744 | - | 0 | 0 | 0 | 622 |
| 6 | PKP2 | c.1170+1G>A | Chr12: 33021860 | NM_004572.4 | Candidate causal | rs786204392 | - | - | - | - | 622 |
| 7 | PKP2 | c.224-1639_274del | Chr12: 33031916 | NM_004572.4 | Candidate causal | - | - | - | - | - | - |
| 8 | TGFB3 | c.*495C>T | Chr14: 76425035 | NM_003239.5 | Uncertain Significance/Candidate causal | rs387906514 | - | 0 | 0 | - | - |
| 9 | DSC2 | c.2250+2T>C | Chr18: 28650690 | NM_024422.6 | Candidate causal | rs1064793731 | - | 0 | - | - | - |
| 10 | DSC2 | c.523+1G>C | Chr18: 29101207 | NM_001943.5 | Candidate causal | rs553299589 | - | 0 | - | - | 622 |
| 11 | DMD | c.531-11_531-6del | ChrX: 32827734-32827739 | NM_004006.3 | Uncertain Significance/Candidate causal | rs758404687 | - | 0 | 0 | - | - |
| 12 | DSC2 | c.154+1G>A | Chr18: 28673521 | NM_024422.6 | Candidate causal | rs397517393 | - | - | - | - | - |
| 13 | DSC2 | c.474+2T>A | Chr18: 28670989 | NM_024422.6 | Candidate causal | - | - | - | - | - | - |
| 14 | DSC2 | c.1664-1G>C | Chr18: 28654874 | NM_024422.6 | Candidate causal | rs776877367 | - | - | 0 | - | - |
| 15 | DSC2 | c.2250+3A>G | Chr18: 28650689 | NM_024422.6 | Uncertain Significance | rs1555637433 | - | - | - | - | - |
| 16 | DSC2 | c.475-13A>G | Chr18: 28669570 | NM_024422.6 | Uncertain Significance | rs751631188 | - | 0 | 0 | 0 | - |
| 17 | DSC2 | c.155-3C>T | Chr18: 28672266 | NM_024422.6 | Uncertain Significance | rs745988877 | - | 0 | 0 | - | - |
| 18 | DSC2 | c.776-3T>C | Chr18: 28666708 | NM_024422.6 | Uncertain Significance | rs752192642 | - | 0 | 0 | 0 | - |
| 19 | DSC2 | c.943-1G>A | Chr18: 28663027 | NM_024422.6 | Candidate causal | rs796756333 | - | 0 | 0 | - | 623 |
| 20 | DSC2 | c.1663+4A>G | Chr18: 28659809 | NM_024422.6 | Uncertain Significance | rs369849387 | - | 0 | 0 | 0 | 624 |
| 21 | DSC2 | c.1521-7C>T | Chr18: 28659962 | NM_024422.6 | Uncertain Significance | rs374810953 | - | 0 | 0 | 0 | 625 |
| 22 | DSC2 | c.2125+1del | Chr18: 28651570 | NM_024422.6 | Candidate causal | rs794728072 | - | 0 | - | 0 | 626 |
| 23 | DSC2 | c.631-2A>G | Chr18: 28667778 | NM_024422.6 | Candidate causal | rs397514042 | - | 0 | 0 | 0 | 627 |
| 24 | DSC2|DSCAS | c.69+2T>G | Chr18: 28681864 | NM_024422.6 | Candidate causal | - | - | - | - | - | - |
| 25 | DSC2|DSCAS | c.69+2T>C | Chr18:28681864 | NM_024422.6 | Candidate causal | - | - | - | - | - | - |
| 26 | DSG2 | c.1880-2A>G | Chr18: 29121154 | NM_001943.5 | Candidate causal | rs397514038 | - | 0 | - | - | 628 |
| 27 | DSG2 | c.45+1G>C | Chr18: 29078260 | NM_001943.5 | Candidate causal | - | - | - | - | - | 629 |
| 28 | DSG2 | c.691-1G>A | Chr18: 29104410 | NM_001943.5 | Candidate causal | rs1555671441 | - | - | - | - | - |
| 29 | DSG2 | c.690+1G>A | Chr18: 29102213 | NM_001943.5 | Candidate causal | rs750176752 | - | 0 | 0 | - | 630 |
| 30 | DSG2 | c.45+9C>G | Chr18: 29078268 | NM_001943.5 | Uncertain Significance | rs376573409 | - | 0 | 0 | - | - |
| 31 | DSG2 | c.523+1G>C | Chr18: 29101207 | NM_001943.5 | Candidate causal | rs553299589 | - | 0 | - | - | 629 |
| 32 | DSG2 | c.216+1G>T | Chr18: 29099901 | NM_001943.5 | Candidate causal | rs1316380114 | - | 0 | 0 | - | 629 |
| 33 | DSG2 | c.523+1_523+2del | Chr18: 29101207-29101208 | NM_001943.5 | Candidate causal | rs1598810829 | - | - | - | - | 629 |
| 34 | DSG2 | c.45+1G>A | Chr18: 29078260 | NM_001943.5 | Candidate causal | rs1568098570 | - | 0 | 0 | - | 629 |
| 35 | DSG2 | c.523+2T>C | Chr18: 29101208 | NM_001943.5 | Candidate causal | rs397516709 | - | 0 | 0 | 0 | 630 |
| 36 | DSG2 | c.45+6T>C | Chr18: 29078268 | NM_001943.5 | Uncertain Significance | - | - | - | - | - | - |
| 37 | DSG2 | c.82-3T>C | Chr18: 29099763 | NM_001943.5 | Uncertain Significance | rs370099811 | - | 0 | 0 | 0 | - |
| 38 | DSG2 | c.828+5C>T | Chr18: 29104553 | NM_001943.5 | Uncertain Significance | rs373286117 | - | 0 | 0 | 0 | - |
| 39 | DSG2 | c.523+1G>A | Chr18: 29101207 | NM_001943.5 | Candidate causal | rs553299589 | - | 0 | - | - | 621 |
| 40 | DSG2 | c.379-3C>T | Chr18: 29101059 | NM_001943.5 | Uncertain Significance | rs376556524 | - | 0 | 0 | 0 | - |
| 41 | DSG2 | c.829-4G>A | Chr18: 29104662 | NM_001943.5 | Uncertain Significance | rs376424003 | - | 0 | - | 0 | - |
| 42 | DSG2|DSG2-AS1 | c.2335-9T>C | Chr18: 29125675 | NM_001943.5 | Uncertain Significance | rs757355636 | - | 0 | 0 | 0 | - |
| 43 | DSP | c.1420-1G>T | Chr6: 7569418 | NM_004415.4 | Candidate causal | rs1060500609 | - | - | - | - | - |
| 44 | DSP | c.2298-1G>C | Chr6: 7574889 | NM_004415.4 | Candidate causal | rs1417627909 | - | - | - | - | - |
| 45 | DSP | c.2794-1G>A | Chr6: 7577191 | NM_004415.4 | Candidate causal | - | - | - | - | - | - |
| 46 | DSP | c.2298-2A>C | Chr6: 7574888 | NM_004415.4 | Candidate causal | - | - | - | - | - | - |
| 47 | DSP | c.778-2A>G | Chr6: 7565590 | NM_004415.4 | Candidate causal | rs113726158 | - | 0 | - | 0 | - |
| 48 | DSP | c.1267-2A>G | Chr6: 7568668 | NM_004415.4 | Candidate causal | rs1554106830 | - | - | - | - | - |
| 49 | DSP | c.2793+1G>T | Chr6: 7576690 | NM_004415.4 | Candidate causal | rs1554107741 | - | - | - | - | - |
| 50 | DSP | c.2437-1G>C | Chr6: 7575527 | NM_004415.4 | Candidate causal | rs1057517903 | - | 0 | - | - | - |
| 51 | DSP | c.939+1del | Chr6: 7565754 | NM_004415.4 | Candidate causal | - | - | - | - | - | - |
| 52 | DSP | c.2436+2T>C | Chr6: 7575030 | NM_004415.4 | Candidate causal | rs774514264 | - | 0 | 0 | 0 | - |
| 53 | DSP | c.1903+3C>T | Chr6: 7571820 | NM_004415.4 | Uncertain Significance | rs754326012 | - | 0 | - | 0 | - |
| 54 | DSP | c.1903+3del | Chr6: 7571820 | NM_004415.4 | Uncertain Significance | rs1064793548 | - | 0 | - | - | - |
| 55 | DSP | c.5379+8C>G | Chr6: 7581810 | NM_004415.4 | Uncertain Significance | rs771984464 | 0 | 0 | 0 | 0 | - |
| 56 | DSP | c.2131-5G>C | Chr6: 7574314 | NM_004415.4 | Uncertain Significance | rs767939332 | - | 0 | 0 | - | - |
| 57 | DSP | c.1903+4G>A | Chr6: 7571821 | NM_004415.4 | Uncertain Significance | rs757726608 | - | 0 | 0 | 0 | - |
| 58 | DSP | c.1574+14G>T | Chr6: 7569587 | NM_004415.4 | Uncertain Significance | rs200443042 | - | 0 | 0 | 0 | - |
| 59 | DSP | c.2794-4dup | Chr6: 7577187-7577188 | NM_004415.4 | Uncertain Significance | rs397516924 | - | 0 | 0 | 0 | - |
| 60 | DSP | c.1575-15C>G | Chr6: 7570655 | NM_004415.4 | Uncertain Significance | rs369184165 | - | 0 | 0 | 0 | - |
| 61 | DSP | c.727-10T>G | Chr6: 7563959 | NM_004415.4 | Uncertain Significance | rs375327581 | - | 0 | 0 | 0 | - |
| 62 | DSP | c.939+1G>A | Chr6: 7565754 | NM_004415.4 | Candidate causal | rs727504443 | - | 0 | 0 | - | 631 |
| 63 | DSP | c.598-4G>A | Chr6: 7562881 | NM_004415.4 | Uncertain Significance | rs747448946 | - | 0 | 0 | 0 | - |
| 64 | DSP | c.1141-2A>T | Chr6: 7568012 | NM_004415.4 | Candidate causal | rs794728111 | - | - | 0 | - | - |
| 65 | JUP | c.*1027G>C | Chr17:39910969 | NM_002230.4 | Uncertain Significance | rs147905567 | - | 0 | 0 | 0 | - |
| 66 | JUP | c.208+1G>A | Chr17: 39927898 | NM_002230.4 | Candidate causal | rs373761090 | - | 0 | 0 | 0 | - |
| 67 | JUP | c.1924+1G>A | Chr17: 39913885 | NM_002230.4 | Candidate causal | rs1597781765 | - | - | - | - | - |
| 68 | JUP | c.*239G>A | Chr17 :39911757 | NM_002230.4 | Uncertain Significance | rs115919416 | - | 0 | 0 | 0 | - |
| 69 | JUP | c.1055-14del | Chr17 : 39921082 | NM_002230.4 | Uncertain Significance | rs140002183 | - | 0 | 0 | 0 | - |
| 70 | JUP | c.2086+13C>T | Chr17 : 39912414 | NM_002230.4 | Uncertain Significance | rs199935213 | - | 0 | 0 | 0 | - |
| 71 | JUP | c.708-4C>G | Chr17 : 39923836 | NM_002230.4 | Uncertain Significance | rs201313464 | - | 0 | 0 | - | 632 |
| 72 | JUP | c.1054+7A>T | Chr17 : 39921168 | NM_002230.4 | Uncertain Significance | rs371988639 | - | 0 | 0 | 0 | - |
| 73 | PKP2 | c.2358-2A>G | Chr12 : 32945667 | NM_001005242.3 | Candidate causal | - | - | - | - | - | - |
| 74 | PKP2 | c.2167+1003_2167+1008del | Chr12 : 32954329-32955343 | NM_001005242.3 | Uncertain Significance | - | - | - | - | - | - |
| 75 | PKP2 | c.2358-1_2358insTT | Chr12 : 32945665-32945666 | NM_001005242.3 | Candidate causal | - | - | - | - | - | 633 |
| 76 | PKP2 | c.1510+1G>A | Chr12 : 32996115 | NM_004572.4 | Candidate causal | rs1332615728 | - | - | 0 | - | - |
| 77 | PKP2 | c.1034+1G>C | Chr12 : 33030779 | NM_001005242.3 | Candidate causal | rs869025496 | - | - | 0 | - | - |
| 78 | PKP2 | c.1689-2A>G | Chr12 : 32977098 | NM_004572.4 | Candidate causal | rs1555143143 | - | - | - | - | - |
| 79 | PKP2 | c.2569_2577+41del | Chr12 : 32945537-32945586 | NM_004572.4 | Candidate causal | rs1064792928 | - | - | - | - | 634 |
| 80 | PKP2 | c.1378+2T>A | Chr12 : 33003698 | NM_001005242.3 | Candidate causal | rs762103704 | - | - | 0 | - | - |
| 81 | PKP2 | c.2357+1G>T | Chr12 : 32949042 | NM_001005242.3 | Candidate causal | rs111517471 | - | 0 | 0 | 0 | 635 |
| 82 | PKP2 | c.1675-3C>T | Chr12 : 32975568 | NM_001005242.3 | Uncertain Significance | - | - | - | - | - | - |
| 83 | PKP2 | c.2300-1G>A | Chr12 : 32949233 | NM_004572.4 | Uncertain Significance/ Candidate causal | rs1060501184 | - | 0 | - | - | - |
| 84 | PKP2 | c.1379-2103A>G | Chr12 : 32996242 | NM_001005242.3 | Uncertain Significance | rs764608036 | - | - | 0 | - | - |
| 85 | PKP2 | c.1379-2065G>A | Chr12 : 32996204 | NM_001005242.3 | Benign | rs771856087 | - | 0 | 0 | 0 | - |
| 86 | PKP2 | c.1556+1G>A | Chr12 : 32993961 | NM_001005242.3 | Candidate causal | rs397517003 | - | 0 | 0 | 0 | 630 |
| 87 | PKP2 | c.337-2A>T | Chr12 : 33031479 | NM_001005242.3 | Candidate causal | rs786204389 | - | - | - | - | 636 |
| 88 | PKP2 | c.2578-2A>C | Chr12 : 32945428 | NM_004572.4 | Candidate causal | rs1060501188 | - | 0 | 0 | - | - |
| 89 | PKP2 | c.1511-5T>C | Chr12 : 32994144 | NM_004572.4 | Uncertain Significance | rs189036647 | - | 0 | 0 | 0 | - |
| 90 | PKP2 | c.1034+1G>T | Chr12 : 33030779 | NM_001005242.3 | Candidate causal | rs869025496 | - | 0 | 0 | - | - |
| 91 | PKP2 | c.2489+5G>A | Chr12 : 32949038 | NM_004572.4 | Uncertain Significance | rs1555141020 | - | - | - | - | - |
| 92 | PKP2 | c.1170+4_1170+7del | Chr12 : 33021854-33021857 | NM_001005242.3 | Uncertain Significance/ Candidate causal | rs397516988 | - | 0 | 0 | - | - |
| 93 | PKP2 | c.1171-2A>G | Chr12 : 33003909 | NM_001005242.3 | Candidate causal | rs794729133 | - | 0 | 0 | 0 | 636 |
| 94 | PKP2 | c.1035-1G>A | Chr12 : 33021997 | NM_001005242.3 | Candidate causal | rs1555147210 | - | - | - | - | - |
| 95 | PKP2 | c.1034+1del | Chr12 : 33030779 | NM_001005242.3 | Candidate causal | rs1555148011 | - | - | - | - | - |
| 96 | PKP2 | c.1170+2T>A | Chr12 : 33021859 | NM_001005242.3 | Candidate causal | rs397516987 | - | - | - | - | - |
| 97 | PKP2 | c.-15G>T | Chr12 : 33049680 | NM_001005242.3 | Uncertain Significance | rs886049325 | - | 0 | - | - | - |
| 98 | PKP2 | c.1378+1G>C | Chr12 : 33003699 | NM_001005242.3 | Candidate causal | rs397516994 | - | - | 0 | - | 630 |
| 99 | RYR2 | c.*399A>G | Chr1: 237996346 | NM_001035.3 | Uncertain Significance | rs184537159 | - | 0 | 0 | 0 | - |
| 100 | RYR2 | c.9129-7T>C | Chr1: 237863522 | NM_001035.3 | Uncertain Significance | rs1466669135 | - | 0 | - | 0 | - |
| 101 | RYR2 | c.3067-14G>C | Chr1: 237713830 | NM_001035.3 | Uncertain Significance | rs757471501 | - | 0 | 0 | 0 | - |
| 102 | RYR2 | c.1612+10A>G | Chr1: 237620045 | NM_001035.3 | Uncertain Significance | rs886046262 | - | - | 0 | - | - |
| 103 | RYR2 | c.11040-3C>T | Chr1: 237897002 | NM_001035.3 | Uncertain Significance | rs876657984 | - | 0 | 0 | 0 | - |
| 104 | SCN5A | c.998+5G>A | Chr3: 38649637 | NM_000335.5 | Uncertain Significance | rs187531872 | - | 0 | 0 | 0 | 637 |
| 105 | TGFB3 | c.*495C>T | Chr14: 76425035 | NM_003239.4 | Uncertain Significance/ Candidate causal | rs387906514 | - | 0 | 0 | - | - |
| 106 | TMEM43 | c.705+6C>T | Chr3: 14176397 | NM_024334.3 | Uncertain Significance | rs185025731 | - | 0 | 0 | 0 | - |
| 107 | TMEM43 | c.781-3C>T | Chr3: 14177304 | NM_024334.3 | Uncertain Significance | rs780142044 | - | 0 | 0 | 0 | - |

Table 5. The candidate causal/likely candidate causal non-coding variants in Restrictive cardiomyopathy (RCM).

| No. | Gene | Variant | Genomic location (hg19) | Transcript | Classification | dbSNP | Frequency of variants (%) | | | | Ref. |
| --- | --- | --- | --- | --- | --- | --- | --- | --- | --- | --- | --- |
|  |  |  |  |  |  |  | **Iranome** | **1000 Genome** | **genomAD** | **TOPMED** |  |
| 1 | LMNA | c.810+1G>A | Chr1: 156104767 | NM_170707.4 | Candidate causal | rs267607632 | - | - | - | - | 6 |
| 2 | LMNA | c.1968+5G>A | Chr1: 156108553 | NM_170707.4 | Uncertain Significance/Candidate causal | rs797044488 | - | - | - | - | 374 |
| 3 | ALMS1 | c.7671+1G>A | Chr2: 73682423 | NM_015120.4 | Candidate causal | rs1417025395 | - | 0 | 0 | 0 | - |
| 4 | SMAD4 | c.1308+2T>C | Chr18: 48593559 | NM_005359.6 | Candidate causal | rs1555686624 | - | - | - | - | 638 |
| 5 | JAG1 | c.2113+1G>T | Chr20: 10626003 | NM_000214.3 | Candidate causal | rs1294950721 | - | 0 | 0 | 0 | - |
| 6 | ACTA1 | c.*248G>A | Chr1: 229566998 | NM_001100.4 | Uncertain Significance | rs551585351 | - | 0 | 0 | 0 | - |
| 7 | ACTA1 | c.454+3G>T | Chr1: 229568300 | NM_001100.4 | Uncertain Significance | rs200976037 | - | 0 | 0 | 0 | - |
| 8 | TTN-AS1 | c.63406+2_63406+3insTT | Chr2: 179443335-179443336 | NM_001267550.2 | Uncertain Significance/Candidate causal | rs536078303 | - | 0 | 0 | - | 524 |
| 9 | TTN | c.25064-4A>G | Chr2: 179582541 | NM_001267550.2 | Uncertain Significance | rs747247583 | - | 0 | 0 | - | 524 |
| 10 | TNNT2 | c.233+6T>C | Chr1: 201335960 | NM_001276345.2 | Uncertain Significance | rs397516449 | - | 0 | 0 | 0 | 366 |
| 11 | TNNT2 | c.851+1G>T | Chr1: 201328750 | NM_001276345.2 | Candidate causal | rs111377893 | - | - | - | - | 639 |
| 12 | TNNT2 | c.810+5G>C | Chr1: 201330402 | NM_001276345.2 | Uncertain Significance | rs730881113 | - | 0 | 0 | 0 | 640 |
| 13 | TNNT2 | c.851+5G>C | Chr1: 201328746 | NM_001276345.2 | Uncertain Significance | rs193922620 | - | - | - | - | - |
| 14 | TNNT2 | c.852-3C>T | Chr1: 201328386 | NM_001276345.2 | Uncertain Significance | rs749454768 | 0 | 0 | - | 0 | 366 |
| 15 | TNNT2 | c.852-1G>T | Chr1: 201328384 | NM_001276345.2 | Candidate causal | rs730881114 | - | - | - | - | 366 |
| 16 | TNNI3 | c.12-7del | Chr19: 55668683 | NM_000363.5 | Uncertain Significance | rs370714315 | - | - | 0 | 0 | 641 |
| 17 | TNNI3 | c.151-6C>G | Chr19: 55667706 | NM_000363.5 | Uncertain Significance | rs377258542 | 0 | 0 | 0 | 0 | - |
| 18 | TNNI3 | c.373-4C>G | Chr19: 55665578 | NM_000363.5 | Uncertain Significance | rs2288530 | - | 0 | 0 | 0 | 642 |
| 19 | TNNI3 | c.372+7C>T | Chr19: 55666102 | NM_000363.5 | Uncertain Significance | rs367809676 | - | 0 | 0 | 0 | 643 |
| 20 | TNNI3 | c.109-15A>G | Chr19: 55668027 | NM_000363.5 | Uncertain Significance | rs779144176 | - | - | - | - | - |
| 21 | TNNI3 | c.283-9C>T | Chr19: 55666207 | NM_000363.5 | Uncertain Significance | rs759922995 | - | 0 | 0 | - | - |
| 22 | TNNI3 | c.-98C>T | Chr19: 55669055 | NM_000363.5 | Uncertain Significance | rs12973773 | - | 0 | - | 0.01 | - |
| 23 | SMAD4 | c.667+3G>A | Chr18: 48581366 | NM_005359.6 | Uncertain Significance | rs757971589 | - | 0 | 0 | 0 | 644 |
| 24 | SMAD4 | c.249+10A>C | Chr18: 48573675 | NM_005359.6 | Uncertain Significance | rs752243771 | - | 0 | 0 | 0 | - |
| 25 | SMAD4 | c.-128+12A>G | Chr18: 48557005 | NM_005359.6 | Uncertain Significance | rs886053891 | - | 0 | 0 | 0 | - |
| 26 | SMAD4 | c.1308+2T>C | Chr18: 48593559 | NM_005359.6 | Candidate causal | rs1555686624 | - | - | - | - | - |
| 27 | SMAD4 | c.*30A>C | Chr18: 48604867 | NM_005359.6 | Uncertain Significance | rs767288576 | - | 0 | 0 | 0 | - |
| 28 | LMNA | c.1968+5G>A | Chr1: 156108553 | NM_170707.4 | Uncertain Significance/Candidate causal | rs797044488 | - | - | - | - | 645 |
| 29 | GJD2-DT|ACTC1 | c.-36C>G | Chr15: 35087720 | NM_005159.5 | Uncertain Significance | rs886051091 | - | 0 | 0 | 0 | - |
| 30 | ALMS1 | c.11551-3T>C | Chr2: 73826525 | NM_015120.4 | Uncertain Significance | rs555547573 | - | 0 | 0 | 0 | 366 |
| 31 | ALMS1 | c.10210+1G>A | Chr2: 73784482 | NM_015120.4 | Candidate causal | rs374681570 | - | 0 | 0 | - | 646 |
| 32 | ALMS1 | c.11540-2A>G | Chr2: 73827803 | NM_001378454.1 | Candidate causal | rs952110960 | - | 0 | 0 | 0 | 647 |
| 33 | ALMS1 | c.11986-2A>T | Chr2: 73829310 | NM_001378454.1 | Candidate causal | rs1394193524 | - | - | - | - | 646 |
| 34 | ALMS1 | c.9779-1G>A | Chr2: 73761950 | NM_001378454.1 | Candidate causal | - | - | - | - | - | 646 |
| 35 | ALMS1 | c.1237+2T>C | Chr2: 73652032 | NM_015120.4 | Candidate causal | rs916056435 | - | 0 | 0 | 0 | - |
| 36 | ALMS1 | c.1432+2_1432+15del | Chr2:73659418-73659431 | NM_015120.4 | Candidate causal | rs1203193062 | - | 0 | 0 | 0 | - |
| 37 | ALMS1 | c.1237+9C>G | Chr2: 73652039 | NM_015120.4 | Uncertain Significance | rs201471551 | - | 0 | - | - | - |
| 38 | ALMS1 | c.646+2T>C | Chr2: 73646448 | NM_015120.4 | Candidate causal | rs1572911143 | - | - | - | - | 646 |
| 39 | ALMS1 | c.12234-2A>G | Chr2: 73835600 | NM_001378454.1 | Candidate causal | - | - | - | - | - |  |
| 40 | ALMS1 | c.450+2T>C | Chr2: 73635877 | NM_001378454.1 | Candidate causal | - | - | - | - | - | 646 |
| 41 | ALMS1 | c.7671+1G>A | Chr2: 73682423 | NM_015120.4 | Candidate causal | rs1417025395 | - | 0 | 0 | 0 | - |
| 42 | ALMS1 | c.9779-1269_9859del | Chr2: 73760682 | NM_001378454.1 | Candidate causal | - | - | - | - | - | - |
| 43 | ALMS1 | c.12296-1G>A | Chr2:73830367 | NM_015120.4 | Candidate causal | rs1313101326 | - | - | 0 | - | - |
| 44 | ALMS1 | c.9536+1G>T | Chr2: 73718626 | NM_015120.4 | Candidate causal | rs1553409901 | - | - | - | - | - |
| 45 | ALMS1 | c.11666-1G>A | Chr2: 73827804 | NM_015120.4 | Candidate causal | rs750737346 | - | 0 | 0 | - | - |
| 46 | ALMS1 | c.12459+1del | Chr2: 73835701 | NM_015120.4 | Candidate causal | rs750907119 | - | 0 | 0 | 0 | - |
| 47 | ALMS1 | c.1_9del | Chr2: 73612996-73613004 | NM_015120.4 | Likely Candidate causal | rs1553396088 | - | - | - | - | - |
| 48 | ALMS1 | c.11869+2T>A | Chr2: 73828010 | NM_015120.4 | Candidate causal | rs1553421657 | - | 0 | - | 0 | - |
| 49 | ALMS1 | c.12111+1G>A | Chr2: 73828564 | NM_015120.4 | Candidate causal | rs376719320 | - | 0 | 0 | 0 | - |
| 50 | ALMS1 | c.1432+1G>T | Chr2: 73659420 | NM_015120.4 | Candidate causal | rs1553401634 | - | - | - | - | - |
| 51 | ALMS1 | c.7672-2A>G | Chr2: 73716759 | NM_015120.4 | Candidate causal | rs748477695 | - | - | 0 | - | - |
| 52 | ALMS1 | c.10076-1G>C | Chr2: 73784346 | NM_015120.4 | Candidate causal | rs1361885101 | - | - | - | - | - |
| 53 | ALMS1 | c.10381+1G>A | Chr2: 73786270 | NM_015120.4 | Candidate causal | rs766366221 | - | - | - | - | - |
| 54 | ALMS1 | c.325-1G>A | Chr2: 73635749 | NM_015120.4 | Candidate causal | rs764337753 | - | - | - | - | - |

Table 6. The candidate causal/likely candidate causal non-coding variants in Congenital heart disease (CHD).

| No. | Gene | Variant | Genomic location (hg19) | Transcript | Classification | dbSNP | Frequency of variants (%) | | | | Ref. |
| --- | --- | --- | --- | --- | --- | --- | --- | --- | --- | --- | --- |
|  |  |  |  |  |  |  | **Iranome** | **1000 Genome** | **genomAD** | **TOPMED** |  |
| 1 | ALG6 | c.257+5G>A | Chr1: 63868019 | NM_013339.4 | Likely Candidate causal | rs199682486 | - | 0 | 0 | 0 | 648 |
| 2 | RBM8A | c.67+32G>C | Chr1: 145507765 | NM_005105.4 | Uncertain Significance/Candidate causal | rs201779890 | 0 | 0 | 0 | 0 | 649 |
| 3 | RBM8A | c.-21G>A | Chr1: 145507646 | NM_005105.4 | Uncertain Significance/Candidate causal | rs139428292 | 0.03 | 0 | 0.02 | 0.02 | 650 |
| 4 | LMNA | c.810+1G>C | Chr1: 156104767 | NM_170707.4 | Candidate causal | rs267607632 | - | - | - | - | 6 |
| 5 | DARS2 | c.492+2T>C | Chr1: 173800770 | NM_018122.5 | Candidate causal | rs142433332 | - | 0 | 0 | 0 | 651 |
| 6 | GFPT1 | c.685+210G>A | Chr2: 69581411 | NM_002056.4 | Candidate causal | - | - | - | - | - | 652 |
| 7 | KYNU | c.170-1G>T | Chr2: 143676177 | NM_003937.3 | Candidate causal | rs1135401744 | - | 0 | - | 0 | 653 |
| 8 | ZEB2 | c.808-2A>G | Chr2: 145158876 | NM_014795.4 | Candidate causal | rs1560607925 | - | - | - | - | 654 |
| 9 | ZEB2 | c.73+1del | Chr2: 145274844 | NM_014795.4 | Candidate causal | rs786204813 | - | - | - | - | 654 |
| 10 | ZEB2 | c.-69-1G>A | Chr2: 145274987 | NM_014795.4 | Candidate causal | rs587776612 | - | - | - | - | 654 |
| 11 | TTN | c.29963-1G>C | Chr1: 179569135 | NM_001267550.2 | Candidate causal | rs1560513651 | - | - | - | - | - |
| 12 | SATB2 | c.346+2T>G | Chr2: 200298059 | NM_001172509.2 | Candidate causal | rs1559052017 | - | - | - | - | - |
| 13 | TGFBR2 | c.1397-1G>A | Chr3: 30729875 | NM_003242.6 | Candidate causal | rs1553631693 | - | - | - | - | 655 |
| 14 | TGFBR2 | c.1524+1G>T | Chr3: 30730004 | NM_003242.6 | Candidate causal | rs727503475 | - | - | - | - | 655 |
| 15 | CC2D2A | c.4179+1del | Chr4: 15589552 | NM_001080522.2 | Candidate causal | rs386833760 | - | 0 | 0 | 0 | 656 |
| 16 | TTC37 | c.2515+1G>C | Chr5: 94852375 | NM_014639.3 | Candidate causal | rs1060499528 | - | - | - | - | 657 |
| 17 | TTC37 | c.1632+1del | Chr5: 94859358 | NM_014639.3 | Candidate causal | rs1060499527 | - | - | - | - | 658 |
| 18 | NKX2-5 | c.335-1G>T | Chr5: 172660213 | NM_004387.4 | Candidate causal | rs864321645 | - | - | - | - | - |
| 19 | SKIV2L | c.2341-2A>G | Chr6: 3267489 | NM_006929.5 | Candidate causal | rs781763471 | - | 0 | 0 | - | - |
| 20 | FBXL4 | c.859-1G>T | Chr6: 99353547 | NM_001278716.2 | Candidate causal | rs368965675 | - | 0 | 0 | 0 | - |
| 21 | FBXL4 | c.858+1G>T | Chr6: 99365249 | NM_001278716.2 | Candidate causal | rs1394080480 | - | - | - | - | 659 |
| 22 | FBXL4 | c.513-1G>A | Chr6: 99365596 | NM_001278716.2 | Candidate causal | rs1554221258 | - | - | - | - | 660 |
| 23 | FBXL4 | c.1389+3_1389+6del | Chr6: 99328423-99328426 | NM_001278716.2 | Uncertain Significance/Candidate causal | rs1554216735 | - | - | - | - | 661 |
| 24 | FBXL4 | c.858+5G>C | Chr6: 99365245 | NM_001278716.2 | Uncertain Significance/Candidate causal | rs1257765682 | - | 0 | 0 | 0 | 659 |
| 25 | LAMA2 | c.3924+2T>C | Chr6: 129637097 | NM_000426.3 | Candidate causal | rs1554269966 | - | - | - | - | 256 |
| 26 | PEX7 | c.903+1G>C | Chr6: 137219380 | NM_000288.4 | Candidate causal | rs148591292 | - | 0 | 0 | 0 | 662 |
| 27 | GATA4 | c.910-55T>C | Chr8: 11612500 | NM_002052.5 | Uncertain Significance/Candidate causal | rs1554498312 | - | - | - | - | 663 |
| 28 | GATA4 | c.997+2T>G | Chr8: 11612644 | NM_002052.5 | Candidate causal | rs864321705 | - | - | - | - | - |
| 29 | GATA4 | c.997+103G>T | Chr8: 11612745 | NM_002052.5 | Uncertain Significance/Candidate causal | rs113049875 | 0 | 0 | - | - | 663,664 |
| 30 | GATA4 | c.998-26G>A | Chr8: 11614418 | NM_002052.5 | Uncertain Significance/Candidate causal | rs1554498708 | - | - | - | - | 663 |
| 31 | GATA4 | c.617-2A>G | Chr8:11606426 | NM_002052.5 | Candidate causal | rs1405855570 | - | 0 | - | 0 | 665 |
| 32 | CHD7 | c.2442+5G>C | Chr8: 61714157 | NM_017780.4 | Uncertain Significance/Candidate causal | rs387906271 | - | - | - | - | 666 |
| 33 | CHD7 | c.5405-7G>A | Chr8:61763045 | NM_017780.4 | Likely Candidate causal | rs398124321 | - | 0 | - | - | 667 |
| 34 | CHD7 | c.5405-17G>A | Chr8: 61763035 | NM_017780.4 | Likely Candidate causal | rs794727423 | - | - | - | - | 667 |
| 35 | TMEM67 | c.1170-2A>G | Chr8:94800070 | NM_001142301.1 | Candidate causal | rs786205608 | - | - | - | - | - |
| 36 | HNRNPK | c.1008+1G>A | Chr9: 86586586 | NM_031263.4 | Candidate causal | rs1554698658 | - | - | - | - | - |
| 37 | HNRNPK | c.214-35A>G | Chr9: 86590455 | NM_031263.4 | Uncertain Significance/Candidate causal | rs1554700718 | - | - | - | - | - |
| 38 | HNRNPK | c.645+1G>T | Chr9: 86587758 | NM_031263.4 | Candidate causal | - | - | - | - | - | - |
| 39 | HNRNPK | c.1192-14_1192-2del | Chr9: 86585248-86585260 | NM_031263.4 | Candidate causal | - | - | - | - | - | - |
| 40 | HNRNPK | c.646-1G>A | Chr9: 86587105 | NM_031263.4 | Candidate causal | rs1564062144 | - | - | - | - | - |
| 41 | HNRNPK | c.953+1dupG | Chr9: 86586795-86586796 | NM_002140.4 | Candidate causal | rs863223402 | - | - | - | - | 668 |
| 42 | TGFBR1 | c.1255+1G>A | Chr9:101908892 | NM_004612.4 | Candidate causal | rs1554702262 | - | - | - | - | 655 |
| 43 | KAT6B | c.3664+1G>A | Chr10:76785008 | NM_012330.4 | Candidate causal | rs1057516033 | - | - | - | - | 669 |
| 44 | FGFR2 | c.1087+1311G>A | Chr10:123276885 | NM_022970.3 | Likely Candidate causal | rs121918491 | - | 0 | - | - | 670 |
| 45 | FGFR2 | c.1087+1304G>A | Chr10: 123276892 | NM_022970.3 | Candidate causal | rs121918487 | - | 0 | - | - | 670 |
| 46 | TH | c.1198-24T>A | Chr11: 2187017 | NM_199292.3 | Uncertain Significance/Candidate causal | rs587776767 | - | - | - | - | 671 |
| 47 | HBB | c.316-106C>G | Chr11: 5247062 | NM_000518.5 | Likely Candidate causal | rs34690599 | 0 | 0 | 0 | - | 672 |
| 48 | HBB | c.315+1G>A | Chr11: 5247806 | NM_000518.5 | Candidate causal | rs33945777 | 0 | 0 | - | - | 673 |
| 49 | HBB | c.93-21G>A | Chr11: 5248050 | NM_000518.5 | Candidate causal | rs35004220 | - | 0 | 0 | 0 | 674 |
| 50 | HBB | c.92+6T>C | Chr11: 5248154 | NM_000518.5 | Likely candidate causal | rs35724775 | - | 0 | 0 | 0 | 675 |
| 51 | HBB | c.92+5G>C | Chr11: 5248155 | NM_000518.5 | Candidate causal | rs33915217 | 0 | 0 | 0 | - | 673 |
| 52 | HBB | c.92+1G>A | Chr11: 5248159 | NM_000518.5 | Candidate causal | rs33971440 | 0 | 0 | 0 | - | 672 |
| 53 | HBB | c.-79A>G | Chr11: 5248330 | NM_000518.5 | Uncertain Significance/Candidate causal | rs34598529 | - | 0 | 0 | 0 | 676 |
| 54 | KMT2A | c.5794-1G>A | Chr11: 118369084 | NM_005933.4 | Candidate causal | rs1555044700 | - | - | - | - | 677 |
| 55 | CBL | c.1096-1G>T | Chr11: 119148875 | NM_005188.3 | Candidate causal | rs397517076 | - | 0 | 0 | 0 | 438 |
| 56 | CBL | c.1096-1G>C | Chr11: 119148875 | NM_005188.3 | Candidate causal | rs397517076 | - | 0 | 0 | 0 | 439 |
| 57 | CBL | c.1228-1G>A | Chr11: 119149219 | NM_005188.3 | Candidate causal | rs587777540 | - | 0 | 0 | 0 | 678 |
| 58 | CACNA1C | c.1114-304G>C | Chr12: 2613704 | NM_000719.7 | Candidate causal | rs786205745 | - | - | - | - | 679 |
| 59 | KMT2D | c.14075+1G>A | Chr12: 49423183 | NM_003482.3 | Candidate causal | rs1057516039 | - | - | - | - | 669 |
| 60 | KMT2D | c.8366+1G>T | Chr12: 49433004 | NM_003482.3 | Candidate causal | rs1057518149 | - | - | - | - | - |
| 61 | KMT2D | c.8229+1G>A | Chr12: 49433217 | NM_003482.3 | Candidate causal | rs1592133086 | - | - | - | - | - |
| 62 | KMT2D | c.4237-2A>C | Chr12: 49440575 | NM_003482.3 | Candidate causal | rs797045001 | - | - | - | - | 680 |
| 63 | KMT2D | c.400+1G>C | Chr12: 49448310 | NM_003482.3 | Candidate causal | rs587783714 | - | - | - | - | - |
| 64 | RPS26 | c.4-2A>T | Chr12: 56436207 | NM_001029.5 | Candidate causal | rs786203998 | - | - | - | - | 681 |
| 65 | UBE3B | c.545-2A>G | Chr12: 109927722 | NM_130466.4 | Candidate causal | rs398123022 | - | 0 | - | - | 682 |
| 66 | UBE3B | c.1741+2T>C | Chr12: 109947521 | NM_130466.4 | Candidate causal | rs398123020 | - | 0 | - | - | 682 |
| 67 | UBE3B | c.2568+1G>A | Chr12: 109962309 | NM_130466.4 | Candidate causal | rs1555269815 | - | - | - | - | - |
| 68 | MYH6 | c.1410+1G>A | Chr14: 23869917 | NM_002471.3 | Candidate causal | rs1566513862 | - | - | - | - | - |
| 69 | MYH7 | c.5655+5G>C | Chr14: 23883211 | NM_000257.4 | Uncertain Significance/Candidate causal | rs1595070689 | - | - | - | - | 394 |
| 70 | SMAD3 | c.401-6G>A | Chr15: 67457585 | NM_005902.4 | Likely Candidate causal | rs745672741 | - | 0 | - | - | 655 |
| 71 | STRA6 | c.1521-1G>A | Chr15: 74473800 | NM_022369.4 | Candidate causal | rs397518484 | - | 0 | 0 | - | 683 |
| 72 | NR2F2 | c.970+1G>A | Chr15: 96877833 | NM_021005.4 | Candidate causal | rs587777374 | - | 0 | - | - | - |
| 73 | CREBBP | c.4890+1G>A | Chr16: 3781776 | NM_004380.3 | Candidate causal | rs1596793242 | - | - | - | - | - |
| 74 | CREBBP | c.4728+2del | Chr16: 3786035 | NM_004380.3 | Candidate causal | rs1596803811 | - | - | - | - | - |
| 75 | CREBBP | c.4019+1G>A | Chr16: 3790399 | NM_001079846.1 | Candidate causal | rs587783491 | - | - | - | - | - |
| 76 | CREBBP | c.3869-2A>G | Chr16: 3790552 | NM_001079846.1 | Candidate causal | rs587783486 | - | - | - | - | - |
| 77 | CREBBP | c.3868+1G>A | Chr16: 3794894 | NM_001079846.1 | Candidate causal | rs398124145 | - | - | - | - | - |
| 78 | CREBBP | c.3914+1G>A | Chr16: 3795277 | NM_004380.3 | Candidate causal | rs1555475352 | - | - | - | - | - |
| 79 | CREBBP | c.3800+1G>T | Chr16: 3795277 | NM_001079846.1 | Candidate causal | rs1555475352 | - | - | - | - | - |
| 80 | CREBBP | c.3723-2A>T | Chr16: 3795357 | NM_001079846.1 | Candidate causal | rs1567277287 | - | - | - | - | 684 |
| 81 | CREBBP | c.3722+1G>C | Chr16: 3799627 | NM_001079846.1 | Candidate causal | rs200782888 | - | - | - | - | 685 |
| 82 | CREBBP | c.3722+1G>A | Chr16: 3799627 | NM_001079846.1 | Candidate causal | rs200782888 | - | - | - | - | - |
| 83 | CREBBP | c.3779+1G>T | Chr16: 3801726 | NM_004380.3 | Candidate causal | rs587783483 | - | - | - | - | - |
| 84 | CREBBP | c.3665+1G>A | Chr16: 3801726 | NM_001079846.1 | Candidate causal | rs587783483 | - | - | - | - | - |
| 85 | CREBBP | c.3610-1G>C | Chr16: 3807378 | NM_004380.3 | Candidate causal | rs1596852670 | - | - | - | - | - |
| 86 | CREBBP | c.3610-2A>G | Chr16: 3807379 | NM_004380.3 | Candidate causal | rs1596852674 | - | - | - | - | - |
| 87 | CREBBP | c.3255+1G>T | Chr16: 3808854 | NM_001079846.1 | Candidate causal | rs587783480 | - | - | - | - | - |
| 88 | CREBBP | c.2947-1G>T | Chr16: 3817911 | NM_001079846.1 | Candidate causal | rs1555481030 | - | - | - | - | - |
| 89 | CREBBP | c.3060+1G>T | Chr16: 3819174 | NM_004380.3 | Candidate causal | rs1596882004 | - | - | - | - | - |
| 90 | CREBBP | c.2159-1G>T | Chr16: 3824695 | NM_004380.3 | Candidate causal | rs1596897799 | - | - | - | - | - |
| 91 | CREBBP | c.1941+2T>C | Chr16: 3828699 | NM_004380.3 | Candidate causal | rs1596909656 | - | - | - | - | - |
| 92 | CREBBP | c.1827+1G>T | Chr16: 3828700 | NM_001079846.1 | Candidate causal | rs1555483834 | - | - | - | - | - |
| 93 | CREBBP | c.1827+1G>A | Chr16: 3828700 | NM_001079846.1 | Candidate causal | rs1555483834 | - | - | - | - | 686 |
| 94 | CREBBP | c.1824-1G>A | Chr16: 3828819 | NM_004380.3 | Candidate causal | rs1596910004 | - | - | - | - | - |
| 95 | CREBBP | c.1573+1G>A | Chr16: 3832684 | NM_004380.3 | Candidate causal | rs1596920360 | - | - | - | - | - |
| 96 | CREBBP | c.1216+1345C>T | Chr16: 3842042 | NM_001079846.1 | Candidate causal | rs587783464 | - | - | - | - | - |
| 97 | CREBBP | c.1216+1332G>A | Chr16: 3842055 | NM_001079846.1 | Candidate causal | rs587783463 | - | - | - | - | - |
| 98 | CREBBP | c.1216+1312C>T | Chr16: 3842075 | NM_001079846.1 | Candidate causal | rs1302427305 | - | 0 | - | 0 | - |
| 99 | CREBBP | c.1216+2T>A | Chr16: 3843385 | NM_004380.3 | Candidate causal | rs1596947522 | - | - | - | - | - |
| 100 | CREBBP | c.86-1G>T | Chr16: 3901011 | NM_001079846.1 | Candidate causal | rs11644721 | - | - | - | - | - |
| 101 | CREBBP | c.86-2A>C | Chr16: 3901012 | NM_001079846.1 | Candidate causal | rs587783515 | - | - | - | - | - |
| 102 | CREBBP | c.-204_85del | Chr16: 3929833 | NM_004380.3 | Candidate causal | rs1567386034 | - | - | - | - | - |
| 103 | CREBBP | c.3982+3A>T | Chr16: 3794892 | NM_004380.3 | Uncertain Significance/Candidate causal | rs1596823180 | - | - | - | - | - |
| 104 | CREBBP | c.3779+1G>C | Chr16: 3801726 | NM_004380.3 | Candidate causal | - | - | - | - | - | 687 |
| 105 | CREBBP | c.2464-6T>A | Chr16: 3820993 | NM_004380.3 | Uncertain Significance/Candidate causal | - | - | - | - | - | - |
| 106 | CREBBP | c.1941+5G>A | Chr16: 3828696 | NM_004380.3 | Uncertain Significance/Candidate causal | - | - | - | - | - | 244 |
| 107 | CREBBP | c.85+1G>A | Chr16: 3929832 | NM_004380.3 | Candidate causal | rs1597099388 | - | - | - | - | - |
| 108 | CREBBP | c.3061-1G>T | Chr16: 3817911 | NM_004380.3 | Candidate causal | rs1555481030 | - | - | - | - | - |
| 109 | CREBBP | c.1941+1G>T | Chr16: 3828700 | NM_004380.3 | Candidate causal | rs1555483834 | - | - | - | - | 688 |
| 110 | CREBBP | c.3914+1G>T | Chr16: 3795277 | NM_004380.3 | Candidate causal | rs1555475352 | - | - |  | - | 689 |
| 111 | CREBBP | c.4134-1G>T | Chr16: 3789726 | NM_004380.3 | Candidate causal | rs886041048 | - | - | - | - | - |
| 112 | CREBBP | c.4281-11C>G | Chr16: 3788684 | NM_004380.3 | Uncertain Significance/Candidate causal | rs587783493 | - | - | - | - | - |
| 113 | CREBBP | c.4133+1G>A | Chr16: 3790399 | NM_004380.3 | Candidate causal | rs587783491 | - | - | - | - | 690 |
| 114 | CREBBP | c.3983-2A>G | Chr16: 3790552 | NM_004380.3 | Candidate causal | rs587783486 | - | - | - | - | - |
| 115 | CREBBP | c.3914+3G>T | Chr16: 3795275 | NM_004380.3 | Uncertain Significance/Candidate causal | rs587783485 | - | - | - | - | - |
| 116 | CREBBP | c.3837-2A>T | Chr16: 3795357 | NM_004380.3 | Candidate causal | rs1567277287 | - | - | - | - | 684 |
| 117 | CREBBP | c.3369+1G>A | Chr16: 3808854 | NM_004380.3 | Candidate causal | rs587783480 | - | - | - | - | 691 |
| 118 | CREBBP | c.3836+1G>A | Chr16: 3799627 | NM_004380.3 | Candidate causal | rs200782888 | - | - | - | - | 692 |
| 119 | CREBBP | c.3982+1G>A | Chr16: 3794894 | NM_004380.3 | Candidate causal | rs398124145 | - | - | - | - | - |
| 120 | CREBBP | c.3836+1G>C | Chr16: 3799627 | NM_004380.3 | Candidate causal | rs200782888 | - | - | - | - | 693 |
| 121 | CREBBP | c.1941+1G>A | Chr16: 3828700 | NM_004380.3 | Candidate causal | rs1555483834 | - | - | - | - | 694 |
| 122 | CREBBP | c.3369+1G>T | Chr16: 3808854 | NM_004380.3 | Candidate causal | rs587783480 | - | - | - | - | 695 |
| 123 | CREBBP | c.3779+1G>A | Chr16: 3801726 | NM_004380.3 | Candidate causal | rs587783483 | - | - | - | - | 696 |
| 124 | MYH11 | c.4578+1G>A | Chr16: 15815278 | NM_002474.3 | Candidate causal | rs397514037 | - | 0 | - | - | 697 |
| 125 | PIGL | c.427-1G>A | Chr17: 16216860 | NM_004278.4 | Candidate causal | rs770084126 | - | 0 | 0 | 0 | 698 |
| 126 | EFTUD2 | c.2823+1del | Chr17: 42929077 | NM_004247.4 | Candidate causal | rs879253728 | - | - | - | - | 699 |
| 127 | EFTUD2 | c.1414-2A>G | Chr17: 42940276 | NM_004247.4 | Candidate causal | rs1597797917 | - | - | - | - | - |
| 128 | EFTUD2 | c.1149+1G>C | Chr17: 42945174 | NM_004247.4 | Candidate causal | rs797045550 | - | - | - | - | - |
| 129 | EFTUD2 | c.1058+1G>A | Chr17: 42945654 | NM_004247.4 | Candidate causal | rs1085307647 | - | - | - | - | - |
| 130 | KANSL1 | c.1652+1G>A | Chr17: 44144914 | NM_001193466.2 | Candidate causal | rs281865470 | - | 0 | - | - | - |
| 131 | SMAD4 | c.1308+2T>C | Chr18: 48593559 | NM_005359.5 | Candidate causal | rs1555686624 | - | - | - | - | 638 |
| 132 | TXNL4A | c.153+3A>G | Chr18: 77748237 | NM_006701.5 | Uncertain Significance/Candidate causal | rs879255559 | - | - | - | - | - |
| 133 | MEGF8 | c.1788+1G>C | Chr19: 42848226 | NM_001271938.2 | Candidate causal | rs1206116606 | - | 0 | 0 | 0 | - |
| 134 | CCDC114 | c.597+1G>A | Chr19: 48814809 | NM_001364171.2 | Candidate causal | rs606231238 | - | 0 | 0 | 0 | 700 |
| 135 | JAG1 | c.2916+1G>C | Chr20: 10622107 | NM_000214.3 | Candidate causal | rs1568791920 | - | - | - | - | - |
| 136 | JAG1 | c.2113+5G>C | Chr20: 10625999 | NM_000214.3 | Uncertain Significance/Candidate causal | rs886044704 | - | - | - | - | - |
| 137 | JAG1 | c.2113+1G>T | Chr20: 10626003 | NM_000214.3 | Candidate causal | rs1294950721 | - | 0 | 0 | 0 | - |
| 138 | SEC23B | c.689+1G>A | Chr20: 18505665 | NM_006363.6 | Candidate causal | rs398124226 | - | 0 | - | - | 701 |
| 139 | LZTR1 | c.-38T>A | Chr22: 21336623 | NM_006767.4 | Uncertain Significance/Candidate causal | rs1459786357 | - | 0 | 0 | 0 | 496 |
| 140 | LZTR1 | c.2220-17C>A | Chr22: 21350968 | NM_006767.4 | Uncertain Significance/Candidate causal | rs1249726034 | - | - | 0 | - | 702 |
| 141 | EP300 | c.3728+5G>C | Chr22: 41558788 | NM_001429.4 | Likely Candidate causal | rs1114167305 | - | - | - | - | 703 |
| 142 | EP300 | c.3857A>G | Chr22: 41562653 | NM_001429.3 | Likely Candidate causal | rs1555910821 | - | 0 | - | 0 | 704 |
| 143 | EP300 | c.4452+5G>T | Chr22: 41566580 | NM_001429.4 | Uncertain Significance/Candidate causal | rs1555911334 | - | - | - | - | - |
| 145 | EP300 | c.4026-9A>G | Chr22: 41564716 | NM_001429.4 | Uncertain Significance/Candidate causal | - | - | - | - | - | 705 |
| 146 | EP300 | c.1878+1G>A | Chr22: 41536262 | NM_001429.4 | Candidate causal | - | - | - | - | - | 706 |
| 147 | EP300 | c.3262-2A>G | Chr22: 41553171 | NM_001429.4 | Candidate causal | rs1555910114 | - | - | - | - | 707 |
| 148 | MID1 | c.757-1G>C | ChrX: 10463732 | NM_033290.4 | Candidate causal | rs1555895725 | - | - | - | - | - |
| 149 | KDM6A | c.2832+1G>A | ChrX: 44936072 | NM_021140.3 | Candidate causal | rs1135401809 | - | - | - | - | 708 |
| 150 | KDM6A | c.2988+1G>C | ChrX: 44936072 | NM_001291415.1 | Candidate causal | rs1135401809 | - | - | - | - | - |
| 151 | GPC3 | c.1292+1G>T | ChrX: 132826396 | NM_004484.3 | Candidate causal | rs869025182 | - | - | - | - | 709 |
| 152 | GPC3 | c.337+1G>A | ChrX: 133087076 | NM_004484.3 | Candidate causal | rs869025183 | - | - | - | - | 710 |
| 153 | BMPR2 | c.529+1G>A | Chr2: 203378553 | NM_001204.7 | Candidate causal | rs1553508187 | - | - | - | - | 711 |
| 154 | BMPR2 | c.1276+1G>A | Chr2: 203397456 | NM_001204.7 | Candidate causal | rs767070218 | - | 0 | - | 0 | 711 |
| 155 | CAPN15 | c.2904+1_2905-45del | Chr16: 602772 | NM_005632.3 | Candidate causal | - | - | - | - | - | - |
| 156 | CDK13 | c.2898-1G>A | Chr7: 40118318 | NM_003718.5 | Candidate causal | - | - |  | - | - | 712 |
| 157 | CBL | c.1096-4_1096-1del | Chr11: 119148872-119148875 | NM_005188.3 | Candidate causal | rs397517077 | - | - | - | - | - |
| 158 | CBL | c.1228-2A>G | Chr11: 119149218 | NM_005188.4 | Candidate causal | rs727504426 | - | - | - | - | 440 |
| 159 | CBL | c.1096-1G>T | Chr11: 119148875 | NM_005188.4 | Candidate causal | rs397517076 | - | 0 | 0 | 0 | 713 |
| 160 | CBL | c.1096-1G>C | Chr11: 119148875 | NM_005188.4 | Candidate causal | rs397517076 | - | 0 | 0 | 0 | 714 |
| 161 | CHD7 | c.4185+1G>A | Chr8:61749572 | NM_017780.4 | Candidate causal | rs1586418093 | - | - | - | - | - |
| 162 | KDM6A | c.3590-2A>G | ChrX: 44945108 | NM_001291415.2 | Candidate causal | - | - | - | - | - | - |
| 163 | KDM6A | c.564+1G>T | ChrX: 44879976 | NM_001291415.2 | Candidate causal | - | - | - | - | - | - |
| 164 | KDM6A | c.443+5G>C | ChrX: 44870269 | NM_001291415.2 | Uncertain Significance/ Candidate causal | - | - | - | - | - | 715 |
| 165 | KDM6A | c.3095-1G>T | ChrX: 44938390 | NM_001291415.2 | Candidate causal | - | - | - | - | - | - |
| 166 | KDM6A | c.875+1G>A | ChrX: 44913201 | NM_001291415.2 | Candidate causal | - | - | - | - | - | - |
| 167 | KDM6A | c.749-2A>G | ChrX: 44913072 | NM_001291415.2 | Candidate causal | - | - | - | - | - | 716 |
| 168 | KDM6A | c.620-2A>G | ChrX: 44896898 | NM_001291415.2 | Candidate causal | - | - | - | - | - | 717 |
| 169 | KDM6A | c.619+6T>C | ChrX: 44894236 | NM_001291415.2 | Uncertain Significance/Candidate causal | - | - | - | - | - | 717 |
| 170 | KDM6A | c.-571del | ChrX: 44732859 | NM_001291421.1 | Candidate causal | rs1555957146 | - | - | - | - | - |
| 171 | KDM6A | c.2832+2dup | ChrX: 44936072-44936073 | NM_021140.3 | Uncertain Significant/Candidate causal | rs797045643 | - | - | - | - | - |
| 172 | KDM6A | c.3736+2T>C | ChrX: 44949177 | NM_021140.3 | Candidate causal | rs793888510 | - | - | - | - | 718 |
| 173 | KDM6A | c.3144+1G>A | ChrX: 44938597 | NM_021140.3 | Candidate causal | rs1602928572 | - | - | - | - | 719 |
| 174 | KDM6A | c.2938+2T>C | ChrX: 44937752 | NM_021140.3 | Candidate causal | rs1569537333 | - | - | - | - | 719 |
| 175 | KDM6A | c.3301-2A>G | ChrX: 44941819 | NM_001291415.2 | Candidate causal | - | - | - | - | - | 719 |
| 176 | KDM6A | c.1425+1G>T | ChrX: 44920665 | NM_021140.3 | Candidate causal | rs1602799769 | - | - | - | - | 719 |
| 177 | KDM6A | c.2832+1G>A | ChrX: 44936072 | NM_021140.3 | Candidate causal | rs1135401809 | - | - | - | - | 719 |
| 178 | KMT2D | c.14515+1del | Chr12: 49421791 | NM_003482.4 | Candidate causal | - | - | - | - | - | - |
| 179 | KMT2D | c.14076-1G>A | Chr12: 49423020 | NM_003482.4 | Candidate causal | - | - | - | - | - | - |
| 180 | KMT2D | c.5867+5G>A | Chr12: 49436339 | NM_003482.4 | Uncertain Significance/ Candidate causal | - | - | - | - | - | - |
| 181 | KMT2D | c.1259-14T>A | Chr12: 49446221 | NM_003482.4 | Uncertain Significance/ Candidate causal | - | - | - | - | - | - |
| 182 | KMT2D | c.8366+2T>C | Chr12: 49433003 | NM_003482.4 | Candidate causal | - | - | - | - | - | - |
| 183 | KMT2D | c.4741+257_6959del | Chr12: 49434594-49439446 | NM_003482.4 | Candidate causal | - | - | - | - | - | - |
| 184 | KMT2D | c.400+2T>C | Chr12: 49448309 | NM_003482.4 | Candidate causal | - | - | - | - | - | - |
| 185 | KMT2D | c.3906+2T>C | Chr12: 49443463 | NM_003482.4 | Candidate causal | - | - | - | - | - | - |
| 186 | KMT2D | c.16412+5G>C | Chr12: 49416058 | NM_003482.4 | Uncertain Significance/Candidate causal | - | - | - | - | - | - |
| 187 | KMT2D | c.176+1G>A | Chr12: 49448682 | NM_003482.4 | Candidate causal | rs1592162768 | - | - | - | - | - |
| 188 | KMT2D | c.8230-1G>A | Chr12: 49433142 | NM_003482.4 | Candidate causal | rs1592132971 | - | - | - | - | - |
| 189 | KMT2D | c.6110-2A>G | Chr12: 49435775 | NM_003482.4 | Candidate causal | - | - | - | - | - | - |
| 190 | KMT2D | c.8366+2T>G | Chr12: 49433003 | NM_003482.4 | Candidate causal | - | - | - | - | - | - |
| 191 | KMT2D | c.7183_7214delinsACT | Chr12: 49434339-49434370 | NM_003482.3 | Candidate causal | rs1592135715 | - | - | - | - |  |
| 192 | KMT2D | c.14069_14075+1del | Chr12: 49423183-49423190 | NM_003482.3 | Candidate causal | rs1555186619 | - | - | - | - | - |
| 193 | KMT2D | c.16338+1G>T | Chr12: 49416372 | NM_003482.3 | Candidate causal | rs1555184782 | - | - | - | - | - |
| 194 | KMT2D | c.14669_14672del | Chr12: 49421077-49421080 | NM_003482.3 | Candidate causal | rs1555186087 | - | - | - | - | - |
| 195 | KMT2D | c.14515+1G>A | Chr12: 49421791 | NM_003482.3 | Candidate causal | rs1555186232 | - | - | - | - | - |
| 196 | KMT2D | c.14000-3C>G | Chr12: 49423262 | NM_003482.3 | Uncertain Significance/Candidate causal | rs1355372507 | - | - | - | - | - |
| 197 | KMT2D | c.10440+2T>G | Chr12: 49428363 | NM_003482.3 | Candidate causal | rs1555189421 | - | - | - | - | - |
| 198 | KMT2D | c.50-1G>T | Chr12: 49448810 | NM_003482.3 | Candidate causal | rs1555198921 | - | - | - | - | - |
| 199 | KMT2D | c.4237-2A>G | Chr12: 49440575 | NM_003482.3 | Candidate causal | rs797045001 | - | - | - | - | - |
| 200 | KMT2D | c.15921+1G>A | Chr12: 49418592 | NM_003482.3 | Candidate causal | rs1555185299 | - | - | - | - | - |
| 201 | KMT2D | c.16413-1G>C | Chr12: 49415935 | NM_003482.3 | Candidate causal | rs793888515 | - | - | - | - | 718 |
| 202 | KMT2D | c.5319+1G>A | Chr12: 49437650 | NM_003482.3 | Candidate causal | rs1057520722 | - | - | - | - | 720 |
| 203 | KMT2D | c.5868-1G>A | Chr12: 49436114 | NM_003482.3 | Candidate causal | rs867333942 | - | 0 | - | - | 720 |
| 204 | KMT2D | c.49+1del | Chr12: 49449058 | NM_003482.3 | Candidate causal | rs1565826250 | - | - | - | - | 720 |
| 205 | KMT2D | c.16338+2T>C | Chr12: 49416371 | NM_003482.3 | Candidate causal | rs1555184777 | - | - | - | - | 720 |
| 206 | KMT2D | c.14515+1G>T | Chr12: 49421791 | NM_003482.3 | Candidate causal | rs1555186232 | - | - | - | - | 721 |
| 207 | KMT2D | c.14075+1G>A | Chr12: 49423183 | NM_003482.3 | Candidate causal | rs1057516039 | - | - | - | - | 722 |
| 208 | KMT2D | c.840-2A>G | Chr12: 49447106 | NM_003482.3 | Candidate causal | rs1555198201 | - | - | - | - | 720 |
| 209 | KMT2D | c.16338+1G>A | Chr12: 49416372 | NM_003482.4 | Candidate causal | - | - | - | - | - | 720 |
| 210 | KMT2D | c.14076-2A>C | Chr12: 49423021 | NM_003482.3 | Candidate causal | rs1592110749 | - | 0 | - | - | 720 |
| 211 | KRAS | c.451-5642A>C | Chr12: 25368487 | NM_004985.5 | Likely Candidate causal | - | - | - | - | - | - |
| 212 | KRAS | c.451-5642A>T | Chr12: 25368487 | NM_004985.5 | Likely Candidate causal | rs1592798693 | - | - | - | - | - |
| 213 | LZTR1 | c.1785+1G>C | Chr22: 21349017 | NM_006767.4 | Candidate causal | - | - | - | - | - | - |
| 214 | LZTR1 | c.993+1G>A | Chr22: 21346119 | NM_006767.4 | Candidate causal | rs770368435 | - | 0 | 0 | 0 | - |
| 215 | LZTR1 | c.510-2A>G | Chr22: 21343076 | NM_006767.4 | Candidate causal | rs1458682620 | - | 0 | - | 0 | - |
| 216 | LZTR1 | c.2407-2A>G | Chr22: 21351519 | NM_006767.4 | Candidate causal | rs1158550690 | - | 0 | 0 | 0 | 723 |
| 217 | MEGF8 | c.3351-2A>C | Chr19: 42857078 | NM_001271938.2 | Candidate causal | - | - | - | - | - | - |
| 218 | MID1 | c.1142-1G>T | ChrX: 10437881 | NM_000381.4 | Candidate causal | - | - | - | - | - | - |
| 219 | MID1 | c.1655+1G>A | ChrX: 10422909 | NM_000381.4 | Candidate causal | - | - | - | - | - | - |
| 221 | RBM10 | c.1249-1G>A | ChrX: 47040613 | NM_005676.5 | Candidate causal | rs1556778986 | - | 0 | 0 | 0 | - |
| 222 | RBM10 | c.724+2T>C | ChrX: 47038564 | NM_005676.5 | Candidate causal | rs1602582334 | - | - | - | - | 724 |
| 224 | SATB2 | c.474-2A>G | Chr2: 200245212 | NM_001172509.2 | Candidate causal | rs1574566973 | - | - | - | - | - |
| 225 | SKIV2L | c.1647+1G>A | Chr6: 31931511 | NM_006929.5 | Uncertain Significance/Candidate causal | - | - | - | - | - | 725 |
| 226 | SKIV2L | c.1404-2A>G | Chr6: 31931188 | NM_006929.5 | Uncertain Significance/Candidate causal | - | - | - | - | - | 725 |
| 227 | TAB2 | c.1764+1G>A | Chr6: 149718901 | NM_001292034.3 | Candidate causal | - | - | - | - | - | - |
| 228 | TBX1 | c.89_284del | Chr22: 19748451-19748646 | NM_001379200.1 | Candidate causal | - | - | - | - | - | - |
| 229 | TBX1 | c.199_224del | Chr22: 19748554-19748579 | NM_001379200.1 | Candidate causal | - | - | - | - | - | - |
| 230 | TBX | c.756-2A>G | Chr12: 114804198 | NM_181486.4 | Candidate causal | - | - | - | - | - | 244 |
| 231 | TBX5 | c.362+1G>A | Chr12: 114837317 | NM_181486.4 | Candidate causal | - | - | - | - | - | - |
| 233 | TBX5 | c.148-2A>G | Chr12: 114839727 | NM_000192.3 | Candidate causal | rs1565942511 | - | 0 | - | - | - |
| 234 | TBX5 | c.510+5G>C | Chr12: 114836373 | NM_000192.3 | Uncertain Significance/Candidate causal | rs1555226301 | - | - | - | - | - |
| 236 | TBX5 | c.664-2A>G | Chr12: 114823374 | NM_000192.3 | Candidate causal | rs1565935458 | - | - | - | - | - |
| 239 | TBX5 | c.755+2T>C | Chr12: 114823279 | NM_000192.3 | Candidate causal | rs1565935314 | - | - | - | - | 726 |
| 240 | TBX5 | c.242+5G>A | Chr12: 114839626 | NM_000192.3 | Uncertain Significant/Candidate causal | rs1555226575 | - | - | - | - | - |
| 241 | TBX5 | c.510+1G>T | Chr12: 114836377 | NM_000192.3 | Candidate causal | rs1565940841 | - | - | - | - | - |
| 242 | TFAP2B | c.822-1G>C | Chr6: 50805687 | NM_003221.4 | Candidate causal | rs80338916 | - | - | - | - | 727 |
| 243 | TFAP2B | c.601+5G>A | Chr6: 50796397 | NM_003221.4 | Uncertain Significance/Candidate causal | rs80338911 | - | 0 | - | - | 727 |
| 244 | TGFB2 | c.346+1G>T | Chr1: 218520390 | NM_003238.5 | Candidate causal | rs1553292145 | - | - | - | - | 728 |
| 245 | TGFB2 | c.755-5_755-2delinsG | Chr1:218609307-218609310 | NM_003238.5 | Candidate causal | rs1553303161 | - | - | - | - | 728 |
| 246 | TTC37 | c.1221+1G>C | Chr5: 94861290 | NM_014639.4 | Candidate causal | - | - | - | - | - | - |
| 247 | TTC37 | c.4620+1G>C | Chr5: 94803569 | NM_014639.3 | Candidate causal | rs370373017 | - | 0 | 0 | 0 | 729 |
| 248 | TTC37 | c.2578-7_2578-3del | Chr5: 94852116-94852120 | NM_014639.3 | Uncertain Significance/Candidate causal | rs746874042 | - | 0 | - | - | 729 |
| 249 | UBE3B | c.2923-1G>T | Chr12: 109971270 | NM_130466.4 | Candidate causal | rs1409120511 | - | 0 | - | 0 | - |
| 250 | ZEB2 | c.2886+1G>A | Chr2: 145187335 | NM_014795.4 | Candidate causal | - | - | - | - | - | - |
| 251 | ZEB2 | c.331+1G>T | Chr2: 145158761 | NM_014795.4 | Candidate causal | - | - | - | - | - | 687 |
| 252 | ZEB2 | c.916+5G>A | Chr2: 145158876 | NM_014795.4 | Uncertain Insignificance/Candidate causal | rs1560607925 | - | - | - | - | 244 |
| 253 | ZEB2 | c.73+2T>C | Chr2: 145274843 | NM_014795.4 | Candidate causal | rs398124282 | - | - | - | - | 730 |
| 255 | GATA4 | c.*77C>T | Chr8:11616061 | NM_002052.5 | Uncertain Significance | **-** | **-** | - | - | - | 731 |
| 256 | GATA4 | c.*280T>C | Chr8: 11616264 | NM_002052.5 | Uncertain Significance | **-** | **-** | - | - | - | 731 |
| 257 | GATA4 | c.*10T>C | Chr8: 11615994 | NM_002052.5 | Uncertain Significance | rs1303390126 | - | 0 | - | 0 | 731 |
| 258 | GATA4 | c.*479A>G | Chr8: 11616463 | NM_002052.5 | Uncertain Significance | **-** | **-** | - | - | - | 731 |
| 259 | GATA4 | c.*462T>C | Chr8: 11616446 | NM_002052.5 | Uncertain Significance | **-** | **-** | - | - | - | 731 |
| 260 | GATA4 | c.*44T>A | Chr8: 11616028 | NM_002052.5 | Uncertain Significance | **-** | **-** | - | - | - | 731 |
| 261 | GATA4 | c.*218C>T | Chr8: 11616202 | NM_002052.5 | Uncertain Significant | rs1023296405 | **-** | 0 | 0 | 0 | 731 |
| 262 | GATA4 | c.*259A>G | Chr8: 11616243 | NM_002052.5 | Uncertain Significant | **-** | **-** | - | - | - | 731 |
| 263 | GATA4 | c.*442A>G | Chr8: 11616426 | NM_002052.5 | Uncertain Significant | **-** | **-** | - | - | - | 731 |
| 264 | ELN | c.950-3C>G | Chr7:73467490 | NM_000501.4 | Uncertain Significant | - | - | - | - | - | 732 |

Table 8. The candidate causal/likely candidate causal non-coding variants in Coronary artery disease.

| No. | Gene | Variant | Genomic location (hg19) | Transcript | Classification | dbSNP | Frequency of variants (%) | | | | Ref. |
| --- | --- | --- | --- | --- | --- | --- | --- | --- | --- | --- | --- |
|  |  |  |  |  |  |  | **Iranome** | **1000 Genome** | **genomAD** | **TOPMED** |  |
| 1 | CELA2A | c.639+1G>C | Chr1: 15792640 | NM_033440.3 | Likely Candidate causal | - | - | - | - | - | 771 |
| 2 | LMNA | c.1968+5G>A | Chr1: 156108553 | NM_170707.4 | Uncertain Significance/Candidate causal | rs797044488 | - | - | - | - | 772 |
| 3 | APOA2 | c.185+1G>A | Chr1: 161192707 | NM_001643.2 | Candidate causal | rs771259264 | - | - | - | - | 773 |
| 4 | PPARG | c.735+10755G>A | Chr3: 12458335 | NM_001330615.1 | Likely Candidate causal | rs72551362 | - | 0 | 0 | - | 774 |
| 5 | PPARG | c.735+10967T>A | Chr3: 12458547 | NM_001330615.1 | Uncertain Significance/Candidate causal | rs72551363 | - | - | - | - | 775 |
| 6 | PPARG | c.735+10817_735+10821del | Chr3: 12458392-12458396 | NM_001330615.1 | Uncertain Significance/Candidate causal | rs1553650477 | - | - | - | - | - |
| 7 | ABCA1 | c.3738+1G>C | Chr9: 107578423 | NM_005502.4 | Candidate causal | rs796051872 | - | 0 | 0 | - | 776 |
| 8 | ABCG8 | c.965-1G>C | Chr2: 44099114 | NM_001195803.2 | Candidate causal | rs957176669 | - | - | 0 | - | 777 |
| 9 | SELP | c.775+1G>A | Chr1: 169582166 | NM_003005.4 | Uncertain Significance/Candidate causal | rs730880284 | - | 0 | 0 | 0 | - |
| 10 | APOA1 | c.-162C>T | Chr11: 116707751 | NM_001318021.1 | Uncertain Significance/Candidate causal | rs121912725 | - | - | - | - | 778 |
| 11 | APOA1 | c.-240-21C>T | Chr11: 116707850 | NM_001318021.1 | Uncertain Significance/Candidate causal | rs387906570 | - | 0 | 0 | 0 | 779 |
| 12 | LIPA | c.111+1G>A | Chr10: 91007294 | NM_000235.4 | Candidate causal | rs762960877 | - | 0 | 0 | 0 | 780 |
| 13 | LIPA | c.111+2T>G | Chr10: 91007293 | NM_000235.4 | Candidate causal | rs1554869292 | - | - | - | - | - |
| 14 | LIPA | c.428+1G>A | Chr10: 90987956 | NM_000235.4 | Candidate causal | rs1554866004 | - | - | - | - | 781 |
| 15 | LIPA | c.539-2A>G | Chr10: 90984987 | NM_000235.4 | Candidate causal | rs762143630 | - | 0 | 0 | - | 782 |
| 16 | LIPA | c.676-2A>G | Chr10: 90983589 | NM_000235.4 | Candidate causal | rs747508159 | - | 0 | 0 | - | 783 |
| 17 | LIPA | c.676-2A>T | Chr10: 90983589 | NM_000235.4 | Candidate causal | rs747508159 | - | - | 0 | - | 784 |
| 18 | LIPA | c.822+1G>A | Chr10: 90983440 | NM_000235.4 | Candidate causal | rs1204744283 | - | 0 | 0 | 0 | 785 |
| 19 | LIPA | c.894+2T>C | Chr10: 90982266 | NM_000235.4 | Candidate causal | rs1554865199 | - | - | - | - | - |

Table 9. The candidate causal/likely candidate causal non-coding variants in Hypertension.

| No. | Gene | Variant | Genomic location (hg19) | Transcript | Classification | dbSNP | Frequency of variants (%) | | | | Ref. |
| --- | --- | --- | --- | --- | --- | --- | --- | --- | --- | --- | --- |
|  |  |  |  |  |  |  | **Iranome** | **1000 Genome** | **genomAD** | **TOPMED** |  |
| 1 | CELA2A | c.639+1G>C | Chr1: 15792640 | NM_033440.3 | Likely Candidate causal | rs752331004 | - | 0 | 0 | 0 | - |
| 2 | RPL5, DIPK1A | c.74-1G>C | Chr1: 93299101 | NM_000969.5 | Candidate causal | rs1553284997 | - | - | - | - | - |
| 3 | YY1AP1 | c.*930G>T | Chr1: 155629714 | NM_001198906.2 | Candidate causal | rs1057519598 | - | 0 | - | 0 | 786 |
| 4 | YY1AP1 | c.*432_*435del | Chr1: 155630209 | NM_001198906.2 | Candidate causal | rs759089960 | - | 0 | - | 0 | - |
| 5 | YY1AP1 | c.997+23T>G | Chr1: 155642328 | NM_001198906.2 | Uncertain Significance/Candidate causal | rs1558307853 | - | 0 | - | - | - |
| 6 | YY1AP1 | c.826-1G>A | Chr1: 155642523 | NM_001198906.2 | Candidate causal | rs199653824 | - | 0 | - | 0 | - |
| 7 | LMNA | c.1488+5G>C | Chr1: 156106824 | NM_170707.4 | Uncertain Significance/Candidate causal | rs267607543 | - | - | - | - | 787 |
| 8 | PIGM | c.-270C>G | Chr1: 160001799 | NM_145167.2 | Uncertain Significance/Candidate causal | rs587776528 | - | 0 | 0 | 0 | 788 |
| 9 | DARS2 | c.228-21_228-20delinsC | Chr1: 173797450 | NM_018122.5 | Uncertain Significance/Candidate causal | rs1553201258 | - | - | - | - | - |
| 10 | DARS2 | c.492+2T>C | Chr1: 173800770 | NM_018122.5 | Candidate causal | rs142433332 | - | 0 | 0 | 0 | 651 |
| 11 | NPHP1 | c.-94_*455del | Chr2: 110927450 | NM_000272.3 | Likely Candidate causal | - | - | - | - | - | - |
| 12 | NPHP1 | c.1884+1G>T | Chr2: 110886762 | NM_000272.4 | Candidate causal | rs1233478832 | - | 0 | 0 | 0 | 789 |
| 13 | NPHP1 | c.1520+1del | Chr2: 110904329 | NM_000272.4 | Candidate causal | rs747861275 | - | - | - | - | 789 |
| 14 | NPHP1 | c.771+77_771+78del | Chr2: 110922187-110922188 | NM_001128178.3 | Candidate causal | - | - | - | - | - | - |
| 15 | NPHP1 | c.771+169G>T | Chr2: 110922096 | NM_001128178.3 | Candidate causal | - | - | - | - | - | 790 |
| 16 | NPHP1 | c.520-1G>T | Chr3: 132437989 | NM_153240.5 | Candidate causal | rs759262253 |  | 0 | - | - | 791 |
| 17 | NPHP3|NPHP3-ACAD11 | c.3812+1G>T | Chr3: 132401546 | NM_153240.5 | Candidate causal | rs1459151671 | - | 0 | - | - | 792 |
| 18 | NPHP3|NPHP3-ACAD11 | c.958-2A>G | Chr3: 132432132 | NM_153240.5 | Candidate causal | rs780148543 | - | 0 | 0 | 0 | 793 |
| 19 | CLASP1, RNU4ATAC | c.196-604C>T | Chr2: 122288505 | NM_015282.3 | Uncertain Significance | rs181195449 | - | 0 | - | - | 794 |
| 20 | TTC21B | c.2758-2A>G | Chr2: 166756392 | NM_024753.5 | Candidate causal | rs766132877 | - | 0 | 0 | - | 795 |
| 21 | MTX2 | c.544-1G>C | Chr2: 177195311 | NM_006554.5 | Candidate causal | rs1575062905 | - | - | - | - | - |
| 22 | BMPR2 | c.-947_-946delinsAT | Chr2: 203241251 | NM_001204.7 | Uncertain Significance/Candidate causal | rs1085307144 | - | - | - | - | 796 |
| 23 | BMPR2 | c.76+1G>T | Chr2: 203242274 | NM_001204.7 | Candidate causal | rs1085307155 | - | - | - | - | 797 |
| 24 | BMPR2 | c.76+2T>C | Chr2: 203242275 | NM_001204.7 | Candidate causal | rs1085307156 | - | - | - | - | 797 |
| 25 | BMPR2 | c.76+5G>A | Chr2: 203242278 | NM_001204.7 | Uncertain Significance/Candidate causal | rs1085307157 | - | - | - | - | 798 |
| 26 | BMPR2 | c.77-35_86del | Chr2: 203329497 | NM_001204.7 | Candidate causal | rs1085307158 | - | - | - | - | 799 |
| 27 | BMPR2 | c.77-1G>A | Chr2: 203329531 | NM_001204.7 | Candidate causal | rs1085307159 | - | - | - | - | 797 |
| 28 | BMPR2 | c.247+1_247+7del | Chr2: 203329702 | NM_001204.7 | Candidate causal | rs1085307189 | - | - | - | - | 798 |
| 29 | BMPR2 | c.247+1G>A | Chr2: 203329703 | NM_001204.7 | Candidate causal | rs1085307188 | - | - | - | - | 797 |
| 30 | BMPR2 | c.247+2del | Chr2: 203329704 | NM_001204.7 | Candidate causal | rs1085307190 | - | - | - | - | 800 |
| 31 | BMPR2 | c.247+6T>G | Chr2: 203329708 | NM_001204.7 | Uncertain Significance/Candidate causal | rs1085307191 | - | - | - | - | 800 |
| 32 | BMPR2 | c.248-592_413delinsGTAAAGTA | Chr2: 203331650 | NM_001204.7 | Candidate causal | rs1553503473 | - | - | - | - | 801 |
| 33 | BMPR2 | c.248-5_248delinsAC | Chr2: 203332237 | NM_001204.7 | Candidate causal | rs1085307192 | - | - | - | - | 798 |
| 34 | BMPR2 | c.248-1G>A | Chr2: 203332241 | NM_001204.7 | Candidate causal | rs1085307194 | - | - | - | - | 802 |
| 35 | BMPR2 | c.418+5_418+8del | Chr2: 203332417 | NM_001204.7 | Uncertain Significance/Candidate causal | rs1085307230 | - | - | - | - | 798 |
| 36 | BMPR2 | c.418+1G>C | Chr2: 203332413 | NM_001204.7 | Candidate causal | rs1085307226 | - | - | - | - | 798 |
| 37 | BMPR2 | c.418+2_418+4delinsGAG | Chr2: 203332414 | NM_001204.7 | Candidate causal | rs1085307227 | - | - | - | - | 803 |
| 38 | BMPR2 | c.418+3A>T | Chr2: 203332415 | NM_001204.7 | Uncertain Significance/Candidate causal | rs1085307228 | - | - | - | - | 804 |
| 39 | BMPR2 | c.418+5G>A | Chr2: 203332417 | NM_001204.7 | Uncertain Significance/Candidate causal | rs1085307229 | - | - | - | - | 805 |
| 40 | BMPR2 | c.1276+1G>A | Chr2: 203397456 | NM_001204.7 | Candidate causal | rs767070218 | - | 0 | - | 0 | 798 |
| 41 | BMPR2 | c.247+136_418+99del | Chr2: 203329838-203332511 | NM_001204.7 | Candidate causal | rs1574499954 | - | - | - | - | 806 |
| 42 | BMPR2 | c.1277-2A>G | Chr2: 203407032 | NM_001204.7 | Candidate causal | rs1574499954 | - | - | - | - | 806 |
| 43 | BMPR2 | c.1128+2T>G | Chr2: 203395679 | NM_001204.7 | Candidate causal | rs1574493841 | - | - | - | - | 806 |
| 44 | BMPR2 | c.852_852+1insA | Chr2: 203383775-203383776 | NM_001204.7 | Candidate causal | rs1574488501 | - | - | - | - | 806 |
| 46 | BMPR2 | c.621+1G>A | Chr2: 203379703 | NM_001204.7 | Candidate causal | rs1553508321 | - | - | - | - | 806 |
| 47 | BMPR2 | c.529+2dup | Chr2: 203378553-203378554 | NM_001204.7 | Uncertain Significance/Candidate causal | rs1574486038 | - | - | - | - | 806 |
| 48 | BMPR2 | c.418+1G>T | Chr2: 203332413 | NM_001204.7 | Candidate causal | rs1085307226 | - | - | - | - | 806 |
| 49 | BMPR2 | c.417_418+2del | Chr2: 203332410-203332413 | NM_001204.7 | Candidate causal | rs1574464226 | - | - | - | - | 806 |
| 50 | BMPR2 | c.76+2T>G | Chr2: 203242275 | NM_001204.7 | Candidate causal | rs1085307156 | - | - | - | - | 806 |
| 51 | BMPR2 | c.76+1G>A | Chr2: 203242274 | NM_001204.7 | Candidate causal | rs1085307155 | - | - | - | - | 806 |
| 52 | BMPR2 | c.248-329_418+6029del | Chr2: 203331913-203338445 | NM_001204.7 | Candidate causal | - | - | - | - | - | 806 |
| 53 | BMPR2 | c.1277-10_1277-9insGGG | Chr2: 203242274 | NM_001204.7 | Uncertain Significance/Candidate causal | rs886039220 | - | - | - | - | - |
| 54 | BMPR2 | c.1587-1G>A | Chr2: 203419974 | NM_001204.7 | Candidate causal | - | - | - | - | - | - |
| 55 | BMPR2 | c.529+1G>A | Chr2: 203378553 | NM_001204.7 | Candidate causal | rs1553508187 | - | - | - | - | 711 |
| 56 | BMPR2 | c.621+1G>T | Chr2: 203379703 | NM_001204.7 | Candidate causal | rs1553508321 | - | - | - | - | - |
| 57 | BMPR2 | c.1129-1_1129dup | Chr2: 203397306-203397307 | NM_001204.7 | Candidate causal | rs1085307302 | - | - | - | - | 797 |
| 58 | BMPR2 | c.51_814del | Chr2: 203242248-203383737 | NM_001204.6 | Candidate causal | - | - | - | - | - | - |
| 59 | BMPR2 | c.16_20del | Chr2: 203242212-203242216 | NM_001204.7 | Candidate causal | rs1085307146 | - | - | - | - | 797 |
| 60 | BMPR2 | c.248-2A>G | Chr2: 203332240 | NM_001204.7 | Candidate causal | rs1085307193 | - | - | - | - | 798 |
| 61 | BMPR2 | c.419-10T>C | Chr2: 203378432 | NM_001204.7 | Uncertain Significance/Candidate causal | rs1085307231 | - | - | - | - | 805 |
| 62 | BMPR2 | c.529+2T>C | Chr2: 203378554 | NM_001204.7 | Candidate causal | rs1085307241 | - | - | - | - | 807 |
| 63 | BMPR2 | c.852+1G>C,A | Chr2: 203383776 | NM_001204.7 | Candidate causal | rs1085307266 | - | - | - | - | 798 |
| 64 | BMPR2 | c.853-2A>G | Chr2: 203384808 | NM_001204.7 | Candidate causal | rs863223424 | - | 0 | 0 | - | 808 |
| 65 | BMPR2 | c.853-1G>C,A | Chr2: 203384809 | NM_001204.7 | Candidate causal | rs1085307267 | - | - | - | - | 798 |
| 66 | BMPR2 | c.967+2T>C | Chr2: 203384926 | NM_001204.7 | Candidate causal | rs1085307277 | - | - | - | - | 797 |
| 67 | BMPR2 | c.967+4del | Chr2: 203384927 | NM_001204.7 | Uncertain Significance/Candidate causal | rs1085307278 | - | - | - | - | 809 |
| 68 | BMPR2 | c.967+5G>T,C | Chr2: 203384929 | NM_001204.7 | Uncertain Significance/Candidate causal | rs1085307279 | - | - | - | - | 810 |
| 69 | BMPR2 | c.968-3C>G | Chr2: 203395514 | NM_001204.7 | Uncertain Significance/Candidate causal | rs1085307280 | - | - | - | - | 797 |
| 70 | BMPR2 | c.968-2A>C | Chr2: 203395515 | NM_001204.7 | Candidate causal | rs1085307281 | - | - | - | - | 811 |
| 71 | BMPR2 | c.968-1G>T | Chr2: 203395516 | NM_001204.7 | Candidate causal | rs1085307282 | - | - | - | - | 797 |
| 72 | BMPR2 | c.1125_1128+16del | Chr2: 203395674 | NM_001204.7 | Candidate causal | rs878854272 | - | - | - | - | - |
| 73 | BMPR2 | c.1128+1G>T,C,A | Chr2: 203395678 | NM_001204.7 | Candidate causal | rs863223420 | - | - | - | - | 805 |
| 74 | BMPR2 | c.1128+1G>C | Chr2: 203395678 | NM_001204.7 | Candidate causal | rs863223420 | - | - | - | - | ^812^, ^802^ |
| 75 | BMPR2 | c.1129-3C>G | Chr2: 203397305 | NM_001204.7 | Uncertain Significance/Candidate causal | rs748230358 | - | - | - | - | 799 |
| 76 | BMPR2 | c.1276+3A>T | Chr2: 203397458 | NM_001204.7 | Uncertain Significance/Candidate causal | rs1085307329 | - | - | - | - | 810 |
| 77 | BMPR2 | c.1276+3A>G | Chr2: 203397458 | NM_001204.7 | Uncertain Significance/Candidate causal | rs1085307329 | - | - | - | - | 802 |
| 78 | BMPR2 | c.1276+4A>G | Chr2: 203397459 | NM_001204.7 | Uncertain Significance/Candidate causal | rs1085307330 | - | - | - | - | 802 |
| 79 | BMPR2 | c.1277-289_1413+4737del | Chr2: 203406745 | NM_001204.6 | Candidate causal | - | - | - | - | - | - |
| 80 | BMPR2 | c.1277-10_1277-9insGGG | Chr2: 203407025 | NM_001204.7 | Uncertain Significance/Candidate causal | rs886039220 | - | - | - | - | 808 |
| 81 | BMPR2 | c.1277-9A>G,C | Chr2: 203407025 | NM_001204.7 | Uncertain Significance/Candidate causal | rs1085307331 | - | - | - | - | 810 |
| 82 | BMPR2 | c.1277-8A>G | Chr2: 203407026 | NM_001204.7 | Uncertain Significance/Candidate causal | rs1085307332 | - | - | - | - | 797 |
| 83 | BMPR2 | c.1413+1G>A | Chr2: 203407171 | NM_001204.7 | Candidate causal | rs1085307349 | - | - | - | - | 805 |
| 84 | BMPR2 | c.1413+3A>T | Chr2: 203407173 | NM_001204.7 | Uncertain Significance/Candidate causal | rs1085307350 | - | - | - | - | 805 |
| 85 | BMPR2 | c.1414-2A>T | Chr2: 203417437 | NM_001204.7 | Candidate causal | rs1085307351 | - | - | - | - | 802 |
| 86 | CUL3 | c.1377+3A>G | Chr2: 225368366 | NM_003590.5 | Uncertain Significance/Candidate causal | rs199469661 | - | - | - | - | - |
| 87 | CUL3 | c.1377+1G>C | Chr2: 225368368 | NM_003590.5 | Candidate causal | rs199469660 | - | - | - | - | - |
| 88 | CUL3 | c.1207-1G>A | Chr2: 225368540 | NM_003590.5 | Candidate causal | rs199469654 | - | - | - | - | 813 |
| 89 | CUL3 | c.1207-3C>T | Chr2: 225368542 | NM_003590.5 | Uncertain Significance/Candidate causal | rs199469653 | - | - | - | - | 813 |
| 90 | CUL3 | c.1207-5T>A | Chr2: 225368544 | NM_003590.5 | Uncertain Significance/Candidate causal | rs199469652 | - | - | - | - | 813 |
| 91 | CUL3 | c.1207-12T>G | Chr2: 225368551 | NM_003590.5 | Uncertain Significance/Candidate causal | rs199469651 | - | - | - | - | 813 |
| 92 | CUL3 | c.1207-26A>G | Chr2: 225368565 | NM_003590.5 | Uncertain Significance/Candidate causal | rs199469650 | - | - | - | - | 813 |
| 93 | CUL3 | c.1207-28T>G | Chr2:225368567 | NM_003590.5 | Uncertain Significance/Candidate causal | rs199469649 | - | 0 | - | 0 | 813 |
| 94 | CUL3 | c.1376_1377+4del | Chr2: 225368365-225368370 | NM_003590.5 | Candidate causal | rs199469657 | - | - | - | - | - |
| 95 | CYP21A2|LOC106780800 | c.293-13C>G | Chr6: 32006858 | NM_000500.9 | Likely Candidate causal | rs6467 | 0 | 0 | - | 0 | 814 |
| 96 | DARS2 | c.228-21_228-20delinsC | Chr1: 173797450-173797451 | NM_018122.5 | Uncertain Significance/Candidate causal | rs1553201258 | - |  | - | - | - |
| 97 | VHL | c.335_340+5del | Chr3: 10183866 | NM_000551.3 | Candidate causal | rs869025632 | - | - | - | - | 245 |
| 98 | VHL | c.339_340+5del | Chr3: 10183868 | NM_000551.3 | Candidate causal | rs1575922562 | - | - | - | - | 815 |
| 99 | VHL | c.340+2_340+6del | Chr3: 10183871 | NM_000551.3 | Candidate causal | rs869025634 | - | - | - | - | 245 |
| 100 | VHL | c.340+1G>A | Chr3: 10183872 | NM_000551.3 | Candidate causal | rs730882032 | - | - | - | - | 815 |
| 101 | VHL, LOC107303340 | c.341-2A>G | Chr3: 10188196 | NM_000551.3 | Candidate causal | rs869025637 | - | - | - | - | 816 |
| 102 | VHL, LOC107303340 | c.341delG | Chr3: 10188197 | NM_000551.3 | Candidate causal | rs869025638 | - | - | - | - | 245 |
| 103 | VHL, LOC107303340 | c.341-3263del | Chr3: 10184935 | NM_000551.3 | Candidate causal | rs869025640 | - | - | - | - | - |
| 104 | LOC107303340, VHL | c.341-3244AC[2] | Chr3: 10188227 | NM_198156.3 | Candidate causal | rs869025644 | - | - | - | - | - |
| 105 | VHL, LOC107303340 | c.341-3237del | Chr3: 10188234 | NM_198156.3 | Candidate causal | rs1553619952 | - | - | - | - | 245 |
| 106 | VHL, LOC107303340 | c.341-3212del | Chr3: 10188258 | NM_198156.3 | Candidate causal | rs869025646 | - | - | - | - | 245 |
| 107 | VHL, LOC107303340 | c.341-3206del | Chr3: 10188263 | NM_198156.3 | Candidate causal | rs397516442 | - | - | - | - | 245 |
| 108 | VHL, LOC107303340 | c.341-3199TC[2] | Chr3: 10188272 | NM_198156.3 | Candidate causal | rs869025649 | - | - | - | - | 817 |
| 109 | VHL, LOC107303340 | c.341-3183del | Chr3: 10188287 | NM_198156.3 | Uncertain Significance/Candidate causal | rs869025651 | - | - | - | - | 245 |
| 110 | VHL, LOC107303340 | c.341-3179_341-3178del | Chr3: 10188292 | NM_198156.3 | Uncertain Significance/Candidate causal | rs869025652 | - | - | - | - | 245 |
| 111 | VHL, LOC107303340 | c.341-3170del | Chr3: 10188297 | NM_198156.3 | Uncertain Significance/Candidate causal | rs869025653 | - | - | - | - | 245 |
| 112 | VHL, LOC107303340 | c.341-3165del | Chr3: 10188305 | NM_198156.3 | Candidate causal | rs794727253 | - | - | - | - | 245 |
| 113 | VHL, LOC107303340 | c.454_463+17del | Chr3: 10188311 | NM_000551.3 | Candidate causal | rs869025656 | - | - | - | - | 245 |
| 114 | VHL, LOC107303340 | c.463+1G>A | Chr3: 10188321 | NM_000551.3 | Candidate causal | rs869025657 | - | - | - | - | 245 |
| 115 | VHL, LOC107303340 | c.463+1G>C | Chr3: 10188321 | NM_000551.3 | Candidate causal | rs869025657 | - | - | - | - | 245 |
| 116 | VHL, LOC107303340 | c.463+2T>C | Chr3: 10188322 | NM_000551.3 | Candidate causal | rs5030814 | - | - | - | - | 245 |
| 117 | VHL, LOC107303340 | c.464-2A>G | Chr3: 10191469 | NM_000551.3 | Candidate causal | rs5030816 | - | - | - | - | 245 |
| 118 | VHL, LOC107303340 | c.464-1G>T | Chr3: 10191470 | NM_000551.3 | Candidate causal | rs5030817 | - | - | - | - | 245 |
| 119 | VHL, LOC107303340 | c.464-1G>C | Chr3: 10191470 | NM_000551.3 | Candidate causal | rs5030817 | - | - | - | - | 245 |
| 120 | VHL, LOC107303340 | c.464-1G>A | Chr3: 10191470 | NM_000551.3 | Candidate causal | rs5030817 | - | - | - | - | 245 |
| 121 | VHL, LOC107303340 | c.*28_*30delinsC | Chr3: 10191481 | NM_001354723.2 | Candidate causal | rs1553620305 | - | - | - | - | 245 |
| 122 | VHL, LOC107303340 | c.*31del | Chr3: 10191482 | NM_001354723.2 | Likely Candidate causal | rs730882020 | - | - | - | - | 245 |
| 123 | VHL, LOC107303340 | c.*84_*90del | Chr3: 10191537 | NM_001354723.2 | Candidate causal | rs1575932266 | - | - | - | - | 818 |
| 124 | VHL, LOC107303340 | c.*94_*97del | Chr3: 10191547 | NM_001354723.2 | Candidate causal | rs869025664 | - | - | - | - | 245 |
| 125 | VHL, LOC107303340 | c.*139_*140del | Chr3: 10191591 | NM_001354723.2 | Candidate causal | rs1553620362 | - | - | - | - | 245 |
| 126 | LOC107303340|VHL | c.341-951_464-1018del | Chr3: 10187244-10190450 | NM_000551.4 | Candidate causal | - | - | - | - | - | - |
| 127 | LOC107303340|VHL | c.341-1583_464-1477del | Chr3: 10186615-10189994 | NM_000551.4 | Candidate causal | - | - | - | - | - | - |
| 128 | LOC107303340|VHL | c.340+1543_464-1191del | Chr3: 10185381-10190247 | NM_000551.4 | Candidate causal | - | - | - | - | - | - |
| 129 | LOC107303340|VHL | c.340+444_464-1187del | Chr3: 10184309-10190278 | NM_000551.4 | Candidate causal | - | - | - | - | - | - |
| 130 | PPARG | c.735+10755G>A | Chr3: 12458335 | NM_001330615.1 | Uncertain Significant/Candidate causal | rs72551362 | - | 0 | - | - | 774 |
| 131 | NPHP3 | c.2694-2_2694-1del | Chr3: 132408108 | NM_153240.5 | Candidate causal | rs751527253 | - | 0 | 0 | 0 | 819 |
| 132 | NPHP3, NPHP3-ACAD11 | c.1985+1G>A | Chr3: 132418196 | NM_153240.5 | Candidate causal | rs1553773271 | - | 0 | - | 0 | - |
| 133 | PKD2 | c.595+1G>C | Chr4: 88929481 | NM_000297.4 | Candidate causal | rs1578111778 | - | - | - | - | - |
| 134 | PKD2 | c.1319+1G>A | Chr4: 88964610 | NM_000297.4 | Candidate causal | rs1131692280 | - | 0 | 0 | 0 | 733 |
| 135 | PKD2 | c.1320-2del | Chr4: 88967792 | NM_000297.4 | Candidate causal | rs1578135823 | - | - | - | - | - |
| 136 | PKD2 | c.2019+1_2019+5del | Chr4: 88979254-88979258 | NM_000297.4 | Candidate causal | rs1578142941 | - | - | - | - | - |
| 137 | PKD2 | c.2522+1_2522+2del | Chr4: 88989212-88989213 | NM_000297.4 | Candidate causal | - | - | - | - | - | - |
| 138 | PKD2 | c.2119-2A>G | Chr4: 88986524 | NM_000297.4 | Candidate causal | - | - | - | - | - | 820 |
| 139 | PKD2 | c.2118+1G>C | Chr4: 88983157 | NM_000297.4 | Candidate causal | - | - | - | - | - | 821 |
| 140 | PKD2 | c.2020-1_2020del | Chr4: 88983056-88983057 | NM_000297.4 | Candidate causal | rs1553927436 | - | - | - | - |  |
| 141 | PKD2 | c.1898+5G>A | Chr4: 88977424 | NM_000297.4 | Likely Candidate causal | rs1553926929 | - | 0 | 0 | 0 | 822 |
| 142 | PKD2 | c.1094+1G>A | Chr4: 88959654 | NM_000297.4 | Candidate causal | rs58606740 | - | 0 | 0 | 0 | 823 |
| 143 | PKD2 | c.2019+1G>A | Chr4: 88979256 | NM_000297.4 | Candidate causal | rs1553927080 | - | 0 | - | - | 824 |
| 144 | PKD2 | c.1094+3_1094+6del | Chr4: 88959654-88959657 | NM_000297.4 | Likely Candidate causal | rs1553925470 | - | 0 | 0 | 0 | 825 |
| 145 | PKHD1 | c.1694-2A>G | Chr6: 51920529 | NM_138694.4 | Candidate causal | - | - | - | - | - | - |
| 146 | PKHD1 | c.390+1G>A | Chr6: 51944697 | NM_138694.4 | Candidate causal | - | - | - | - | - | - |
| 147 | PKHD1 | c.880+1G>A | Chr6: 51930773 | NM_138694.4 | Candidate causal | - | - | - | - | - | - |
| 148 | PKHD1 | c.1964+1G>T | Chr6: 51918835 | NM_138694.4 | Candidate causal | rs1385712943 | - | 0 | 0 | 0 | 826 |
| 149 | PKHD1 | c.2280-2A>G | Chr6: 51913419 | NM_138694.4 | Candidate causal | rs780675990 | - | 0 | - | - | 826 |
| 150 | PKHD1 | c.6808+1G>T | Chr6: 51771012 | NM_138694.4 | Candidate causal | rs1217968843 | - | 0 | 0 | 0 | 826 |
| 151 | PKHD1 | c.3561-1G>A | Chr6: 51892695 | NM_138694.4 | Candidate causal | - | - | - | - | - | 826 |
| 152 | PKHD1 | c.8440+1G>A | Chr6: 51656033 | NM_138694.4 | Candidate causal | - | - | - | - | - | 826 |
| 153 | PKHD1 | c.5380+2T>G | Chr6: 51887597 | NM_138694.4 | Candidate causal | - | - | - | - | - | 826 |
| 154 | PKHD1 | c.1118+1G>T | Chr6: 51927316 | NM_138694.4 | Candidate causal | - | - | - | - | - | 826 |
| 155 | PKHD1 | c.6866-1G>C | Chr6: 51768526 | NM_138694.4 | Candidate causal | - | - | - | - | - | 826 |
| 156 | PKHD1 | c.281+1G>A | Chr6: 51947189 | NM_138694.4 | Candidate causal | rs1582154630 | - | - | - | - | - |
| 157 | PKHD1 | c.1836+1G>A | Chr6: 51920384 | NM_138694.4 | Candidate causal | rs780898021 | - | 0 | - | 0 | 826 |
| 158 | PKHD1 | c.2140+1G>A | Chr6: 51917873 | NM_138694.4 | Candidate causal | rs1554213853 | - | - | - | - | - |
| 159 | PKHD1 | c.5600+1G>A | Chr6: 51882207 | NM_138694.4 | Candidate causal | rs1581763359 | - | - | - | - | - |
| 160 | PKHD1 | c.5751+3A>G | Chr6: 51875104 | NM_138694.4 | Uncertain Significance/ Candidate causal | rs1581726525 | - | - | - | - | 827 |
| 161 | PKHD1 | c.8174-2A>T | Chr6: 51695789 | NM_138694.4 | Candidate causal | rs1582925274 | - | 0 | - | 0 | - |
| 162 | PKHD1 | c.11399-1G>A | Chr6: 51503755 | NM_138694.4 | Candidate causal | rs1183281205 | - | 0 | 0 | - | - |
| 163 | PKHD1 | c.5601-8C>T | Chr6: 51875265 | NM_138694.4 | Uncertain Significance | rs578030952 | - | 0 | 0 | - | - |
| 164 | PKHD1 | c.11398+2T>C | Chr6: 51512827 | NM_138694.4 | Candidate causal | rs754038777 | - | 0 | 0 | 0 | 826 |
| 165 | PKHD1 | c.6809-2A>T | Chr6: 51768842 | NM_138694.4 | Candidate causal | rs1340926191 | - | 0 | - | 0 | 826 |
| 166 | PKHD1 | c.11174_11175-4798del | Chr6: 51768526 | NM_138694.4 | Candidate causal | - | - |  | - | - |  |
| 167 | PKHD1 | c.778+1G>C | Chr6: 51934254 | NM_138694.4 | Candidate causal | rs753471298 | - | 0 | - | - | 826 |
| 168 | PKHD1 | c.8798-2A>T | Chr6: 51618153 | NM_138694.4 | Candidate causal | rs1582470309 | - | - | - | - | - |
| 169 | PKHD1 | c.9829+1G>A | Chr6: 51612584 | NM_138694.4 | Candidate causal | rs1273202231 | - | 0 | - | - | - |
| 170 | PKHD1 | c.6866-2_6866-1delinsGA | Chr6: 51768526-51768527 | NM_138694.4 | Candidate causal | rs1562581286 | - | - | - | - | - |
| 171 | PKHD1 | c.5751+1G>A | Chr6: 51875106 | NM_138694.4 | Candidate causal | rs775638588 | - | 0 | - | 0 | 826 |
| 172 | PKHD1 | c.8107+1G>C | Chr6: 51712572 | NM_138694.4 | Candidate causal | rs1554270880 | - | - | - | - | - |
| 173 | PKHD1 | c.53-2A>G | Chr6: 51948055 | NM_138694.4 | Candidate causal | rs1554228601 | - | - | - | - | - |
| 174 | PKHD1 | c.3228+1G>C | Chr6: 51900388 | NM_138694.4 | Candidate causal | rs868673401 | - | 0 | - | 0 | - |
| 175 | PKHD1 | c.708-1G>A | Chr6: 51934326 | NM_138694.4 | Candidate causal | rs1554222508 | - | - | - | - | - |
| 176 | PKHD1 | c.8303-2A>G | Chr6: 51656173 | NM_138694.4 | Candidate causal | rs1554243821 | - | - | - | - | - |
| 177 | PKHD1 | c.603-1G>A | Chr6: 51935869 | NM_138694.4 | Candidate causal | rs1554223403 | - | - | - | - | - |
| 178 | PKHD1 | c.2715+1G>C | Chr6: 51909763 | NM_138694.4 | Candidate causal | rs1554209188 | - | - | 0 | - | - |
| 179 | PKHD1 | c.390+1del | Chr6: 51944697 | NM_138694.4 | Candidate causal | rs1554227162 | - | - | - | - | 828 |
| 180 | PKHD1 | c.7486+1G>T | Chr6: 51735301 | NM_138694.4 | Candidate causal | rs1554282419 | - | - | 0 | - | - |
| 181 | PKHD1 | c.7912-2A>G | Chr6: 51712770 | NM_138694.4 | Candidate causal | rs1554271235 | - | - | - | - | 829 |
| 182 | PKHD1 | c.708-2A>C | Chr6: 51934327 | NM_138694.4 | Candidate causal | rs753492974 | - | - | - | - | - |
| 183 | PKHD1 | c.11311-2A>G | Chr6: 51512918 | NM_138694.4 | Candidate causal | rs1554174903 | - | 0 | - | 0 | - |
| 184 | PKHD1 | c.3097+2T>C | Chr6: 51907655 | NM_138694.4 | Candidate causal | rs1554207834 | - | - | - | - | - |
| 185 | PKHD1 | c.2822-1G>C | Chr6: 51907933 | NM_138694.4 | Candidate causal | rs1554208257 | - | - | - | - | - |
| 186 | PKHD1 | c.8950+1G>T | Chr6: 51617998 | NM_138694.4 | Candidate causal | rs1351918391 | - | - | - | - | - |
| 187 | PKHD1 | c.9998+1G>T | Chr6: 51611518 | NM_138694.4 | Candidate causal | rs1554217691 | - | - | - | - | - |
| 188 | PKHD1 | c.11665+1G>A | Chr6: 51497362 | NM_138694.4 | Candidate causal | rs759851475 | 0 | 0 | - | - | 826 |
| 189 | PKHD1 | c.2408-2A>G | Chr6: 51910988 | NM_138694.4 | Candidate causal | rs1554210085 | - | - | - | - | - |
| 190 | PKHD1 | c.6809-2A>G | Chr6: 51768842 | NM_138694.4 | Candidate causal | rs1340926191 | - | 0 | - | 0 | - |
| 191 | PKHD1 | c.11785+1G>T | Chr6: 51491794 | NM_138694.4 | Candidate causal | rs1488844530 | - | 0 | - | 0 | - |
| 192 | PKHD1 | c.2140+1G>C | Chr6: 51917873 | NM_138694.4 | Candidate causal | rs1554213853 | - | - | - | - | - |
| 193 | PKHD1 | c.6866-16_6866del | Chr6: 51768525-51768541 | NM_138694.4 | Candidate causal | rs1554299291 | - | - | - | - | - |
| 194 | PKHD1 | c.6491-1G>A | Chr6: 51774273 | NM_138694.4 | Candidate causal | rs1554132790 | - | - | - | - | 830 |
| 195 | PKHD1 | c.8555-2A>G | Chr6: 51637589 | NM_138694.4 | Candidate causal | rs1020621286 | - | 0 | 0 | - | - |
| 196 | PKHD1 | c.2140+1G>T | Chr6: 51917873 | NM_138694.4 | Candidate causal | rs1554213853 | - | - | - | - | - |
| 197 | PKHD1 | c.282-2A>T | Chr6: 51944808 | NM_138694.4 | Candidate causal | rs1554227278 | - | 0 | 0 | - | - |
| 198 | PKHD1 | c.6490+1G>A | Chr6: 51776596 | NM_138694.4 | Candidate causal | rs1554133736 | - | - | - | - | - |
| 199 | PKHD1 | c.7350+653A>G | Chr6: 51747238 | NM_138694.4 | Likely Candidate causal | rs1240212722 | - | 0 | 0 | 0 | 831 |
| 200 | PKHD1 | c.7912-1G>A | Chr6: 51712769 | NM_138694.4 | Candidate causal | rs1160209891 | - | - | - | - | - |
| 201 | PKHD1 | c.3228+1G>T | Chr6: 51900388 | NM_138694.4 | Candidate causal | rs868673401 | - | 0 | - | 0 | 832 |
| 202 | PKHD1 | c.53-1G>A | Chr6: 51948054 | NM_138694.4 | Candidate causal | rs775511838 | - | 0 | - | - | 826 |
| 203 | PKHD1 | c.2141-2A>C | Chr6: 51915095 | NM_138694.4 | Candidate causal | rs1554212403 | - | - | - | - | - |
| 204 | PKHD1 | c.5909-2del | Chr6: 51799122 | NM_138694.4 | Candidate causal | rs1334913120 | - | 0 | - | 0 | 833 |
| 205 | PKHD1 | c.707+2T>C | Chr6: 51935202 | NM_138694.4 | Candidate causal | rs1554222939 | - | - | - | - | - |
| 206 | PKHD1 | c.5237-2A>C | Chr6: 51887744 | NM_138694.4 | Candidate causal | rs1554197152 | - | - | - | - | - |
| 207 | PKHD1 | c.11507-7G>A | Chr6: 51497528 | NM_138694.4 | Uncertain Significance | rs373438297 | 0 | 0 | 0 | 0 | - |
| 208 | PKHD1 | c.881-1G>A | Chr6: 51929849 | NM_138694.4 | Candidate causal | rs1554220431 | - | 0 | - | 0 | 827 |
| 209 | PKHD1 | c.7351-1G>C | Chr6: 51735438 | NM_138694.4 | Candidate causal | rs1554282540 | - | - | - | - | - |
| 210 | PKHD1 | c.1233+99_1694-441del | Chr6: 51920968-51924627 | NM_138694.4 | Candidate causal | - | - | - | - | - | - |
| 211 | PKHD1 | c.6122-3_6122-1delinsA | Chr6: 51777375-51777377 | NM_138694.4 | Candidate causal | rs1057517378 | - | - | - | - | - |
| 212 | PKHD1 | c.448+2T>C | Chr6: 51941072 | NM_138694.4 | Candidate causal | rs1057517270 | - | - | - | - | - |
| 213 | PKHD1 | c.4_7del | Chr6:51949725-51949728 | NM_138694.4 | Candidate causal | rs1057517220 | - | 0 | - | 0 | - |
| 214 | PKHD1 | c.3561-2A>G | Chr6: 51892696 | NM_138694.4 | Candidate causal | rs1057516982 | - | - | - | - | - |
| 215 | PKHD1 | c.3229-2A>C | Chr6: 51897965 | NM_138694.4 | Candidate causal | rs1057516872 | - | - | - | - | 834 |
| 216 | PKHD1 | c.527+1del | Chr6: 51938260 | NM_138694.4 | Candidate causal | rs1057516697 | - | - | - | - | - |
| 217 | PKHD1 | c.7350+1G>T | Chr6: 51747890 | NM_138694.4 | Candidate causal | rs1057516588 | - | - | - | - | - |
| 218 | PKHD1 | c.2715+2_2715+14del | Chr6: 51909750-51909762 | NM_138694.4 | Candidate causal | rs1057516562 | - | 0 | - | - | - |
| 219 | PKHD1 | c.5752-2A>G | Chr6: 51824826 | NM_138694.4 | Candidate causal | rs1057516407 | - | 0 | - | 0 | 826 |
| 220 | PKHD1 | c.3229-2del | Chr6: 51897965 | NM_138694.4 | Candidate causal | rs1057516283 | - | - | - | - | - |
| 221 | PKHD1 | c.603-2A>G | Chr6: 51935870 | NM_138694.4 | Candidate causal | rs757521428 | - | - | - | - | 687 |
| 222 | PKHD1 | c.5236+1G>A | Chr6: 51889371 | NM_138694.4 | Candidate causal | rs398124487 | - | - | - | - | - |
| 223 | PKHD1 | c.390+1G>T | Chr6: 51944697 | NM_138694.4 | Candidate causal | rs752327566 | - | 0 | 0 | 0 | 828 |
| 224 | PKHD1 | c.8555-2A>C | Chr6: 51637589 | NM_138694.4 | Candidate causal | rs1020621286 | - | 0 | 0 | - | 826 |
| 225 | PKHD1 | c.1233+1G>A | Chr6: 51924725 | NM_138694.4 | Candidate causal | rs886061623 | - | 0 | - | 0 | 835 |
| 226 | PDE8B | c.876+4525A>C | Chr5:76645281 | NM_001029851.3 | Likely Candidate causal | rs121918360 | - | 0 | - | - | 836 |
| 227 | KLHL3 | c.753+1G>A | Chr5: 136997603 | NM_017415.3 | Candidate causal | rs199469648 | - | - | - | - | - |
| 228 | DCDC2 | c.349-2A>G | Chr6: 24302274 | NM_016356.5 | Candidate causal | rs760040426 | - | 0 | - | 0 | 837 |
| 229 | PKHD1 | c.8642+1G>A | Chr6: 51637499 | NM_138694.4 | Candidate causal | rs1485161784 | - | - | - | - | 838 |
| 230 | PKHD1 | c.8303-1G>A | Chr6: 51656172 | NM_138694.4 | Candidate causal | rs786204241 | - | - | - | - | 838 |
| 231 | PKHD1 | c.1233+99_1694-441del | Chr6: 51920969 | NM_138694.4 | Candidate causal | - | - | - | - | - | - |
| 232 | PKHD1 | c.1693+2T>G | Chr6: 51921494 | NM_138694.4 | Candidate causal | rs1581999471 | - | - | - | - | 838 |
| 233 | PKHD1 | c.707+1G>A | Chr6: 51935203 | NM_138694.4 | Candidate causal | rs748365248 | - | - | - | - | 838 |
| 234 | ENPP1 | c.795+1G>A | Chr6: 132179888 | NM_006208.3 | Candidate causal | rs753851892 | - | - | - | - | 839 |
| 235 | ENPP1 | c.2101-2A>T | Chr6: 132203483 | NM_006208.3 | Candidate causal | rs1585841844 | - | - | - | - | - |
| 236 | ASL | c.446+1G>A | Chr7: 65548162 | NM_000048.4 | Candidate causal | rs142637046 | - | 0 | 0 | 0 | 840 |
| 237 | ASL | c.447-1G>A | Chr7: 65548171 | NM_000048.4 | Candidate causal | rs778254333 | - | 0 | 0 | 0 | 840 |
| 238 | ASL | c.524+2T>G | Chr7: 65551651 | NM_000048.4 | Candidate causal | rs869312976 | - | 0 | 0 | - | 840 |
| 239 | ASL | c.1045_1062+7del | Chr7: 65554664 | NM_000048.4 | Candidate causal | rs1329070853 | - | 0 | 0 | 0 | 840 |
| 240 | POR | c.731+1G>A | Chr7: 75610925 | NM_000941.3 | Candidate causal | rs786205099 | - | - | - | - | 841 |
| 241 | CFTR | c.1766+1G>A | Chr7: 117230494 | NM_000492.3 | Candidate causal | rs121908748 | - | 0 | 0 | 0 | 842 |
| 242 | AGK | c.975+1G>T | Chr7: 141341709 | NM_018238.4 | Candidate causal | rs868431923 | - | 0 | - | - | 445 |
| 243 | ANKS6 | c.2512-2A>C | Chr9: 101498907 | NM_173551.5 | Candidate causal | rs397514258 | - | 0 | - | - | 843 |
| 244 | LMX1B | c.741+1G>T | Chr9: 129455603 | NM_002316.3 | Candidate causal | rs1427331961 | - | - | - | - | - |
| 245 | LMX1B | c.819+1G>A | Chr9: 129455881 | NM_001174147.2 | Candidate causal | rs1588307501 |  |  | - | - |  |
| 246 | ENG | c.816+6T>C | Chr9: 130587504 | NM_000118.3 | Uncertain Significance/Candidate causal | rs759191907 | - | 0 | - | - | 843 |
| 247 | CYP17A1 | c.297+2T>C | Chr10: 104596820 | NM_000102.4 | Candidate causal | rs764723654 | - | 0 | 0 | 0 | - |
| 248 | FGFR2 | c.1087+1331C>G | Chr10: 123276865 | NM_022970.3 | Candidate causal | rs121918502 | - | - | - | - | 844 |
| 249 | FGFR2 | c.1087+1311G>A | Chr10: 123276885 | NM_022970.3 | Likely Candidate causal | rs121918491 | - | 0 | - | - | 670 |
| 250 | FGFR2 | c.1087+1304G>A | Chr10: 123276892 | NM_022970.3 | Candidate causal | rs121918487 | - | 0 | - | - | 670 |
| 251 | FGFR2 | c.1087+1303T>A | Chr10: 123276893 | NM_022970.3 | Candidate causal | rs121918488 | - | - | - | - | 845 |
| 252 | FGFR2 | c.1087+1303T>C | Chr10: 123276893 | NM_022970.3 | Candidate causal | rs121918488 | - | - | - | - | 846 |
| 253 | HTRA1 | c.973-1G>A | Chr10: 124266885 | NM_002775.5 | Candidate causal | rs864622783 | - | - | - | - | 847 |
| 254 | HBB | c.316-106C>G | Chr11: 5247062 | NM_000518.5 | Likely Candidate causal | rs34690599 | 0 | 0 | 0 | - | 672 |
| 255 | HBB, LOC106099062, LOC107133510, LOC110006319 | c.315+1G>A | Chr11: 5247806 | NM_000518.5 | Candidate causal | rs33945777 | 0 | 0 | - | - | 673 |
| 256 | HBB, LOC106099062, LOC107133510 | c.93-21G>A | Chr11: 5248050 | NM_000518.5 | Candidate causal | rs35004220 | - | 0 | 0 | 0 | 674 |
| 257 | HBB, LOC106099062, LOC107133510 | c.92+6T>C | Chr11: 5248154 | NM_000518.5 | Likely Candidate causal | rs35724775 | - | 0 | 0 | 0 | 675 |
| 258 | HBB, LOC106099062, LOC107133510 | c.92+5G>C | Chr11: 5248155 | NM_000518.5 | Likely Candidate causal | rs33915217 | 0 | 0 | 0 | - | 673 |
| 259 | HBB, LOC106099062, LOC107133510 | c.92+1G>A | Chr11: 5248159 | NM_000518.5 | Candidate causal | rs33971440 | 0 | 0 | 0 | - | 848 |
| 260 | HBB, LOC106099062, LOC107133510 | c.-79A>G | Chr11: 5248330 | NM_000518.5 | Uncertain Significance/ Candidate causal | rs34598529 | - | 0 | 0 | 0 | 849 |
| 261 | MEN1 | c.1366-2_*132del | Chr11: 64571674 | NM_000244.3 | Likely Candidate causal | rs1565634591 | - | - | - | - | 245 |
| 262 | MEN1 | c.1365+1_1365+11del | Chr11: 64572495 | NM_000244.3 | Uncertain Significance/ Candidate causal | rs764570645 | - | - | - | - | 245 |
| 263 | MEN1 | c.1065-2A>G | Chr11: 64573244 | NM_000244.3 | Candidate causal | rs1565642765 | - | - | - | - | 245 |
| 264 | MEN1 | c.1065-2A>T | Chr11: 64573244 | NM_000244.3 | Candidate causal | rs1565642765 | - | - | - | - | 245 |
| 265 | MEN1 | c.638_1065-143del | Chr11: 64573385 | NM_000244.3 | Candidate causal | - | - | - | - | - | - |
| 266 | MEN1 | c.955_1065-227del | Chr11: 64573469 | NM_000244.3 | Candidate causal | rs1555164870 | - | - | - | - | 245 |
| 267 | MEN1 | c.1064+1G>A | Chr11: 64573703 | NM_000244.3 | Candidate causal | rs1114167489 | - | - | - | - | 245 |
| 268 | MEN1 | c.839+1G>T | Chr11: 64574650 | NM_000244.3 | Candidate causal | rs1060499976 | - | - | - | - | 245 |
| 269 | MEN1 | c.839+1G>A | Chr11: 64574650 | NM_000244.3 | Candidate causal | rs1060499976 | - | - | - | - | 245 |
| 270 | MEN1 | c.799-9G>A | Chr11: 64574700 | NM_000244.3 | Likely Candidate causal | rs794728625 | - | - | - | - | 245 |
| 271 | MEN1 | c.783+1G>C | Chr11: 64575023 | NM_001370259.2 | Candidate causal | - | - |  | - | - | 245 |
| 272 | MEN1 | c.798+1G>A | Chr11: 64575023 | NM_000244.3 | Candidate causal | rs794728652 | - | - | - | - | 850 |
| 273 | MEN1 | c.670-1G>A | Chr11: 64575153 | NM_000244.3 | Candidate causal | rs1592649615 | - |  | - |  | 245 |
| 274 | MEN1 | c.669+1del | Chr11: 64575362 | NM_000244.3 | Candidate causal | rs1057518903 | - | - | - | - | 851 |
| 275 | MEN1 | c.669+1G>T | Chr11: 64575362 | NM_000244.3 | Candidate causal | rs794728622 | - | - | - | - | 245 |
| 276 | MEN1 | c.669+1G>A | Chr11: 64575362 | NM_000244.3 | Candidate causal | rs794728622 | - | - | - | - | 150 |
| 277 | MEN1 | c.461-1G>C | Chr11: 64575572 | NM_000244.3 | Candidate causal | rs1064793672 | - | - | - | - | 245 |
| 278 | MEN1 | c.664_669+2del | Chr11: 64575361-64575368 | NM_000244.3 | Candidate causal | rs386134258 | - | - | - | - | - |
| 279 | MEN1 | c.670-15_673del | Chr11: 64575149-64575167 | NM_000244.3 | Candidate causal | rs1592649598 | - | - | - | - | - |
| 280 | MEN1 | c.1064+2T>C | Chr11: 64573702 | NM_000244.3 | Candidate causal | rs1555164946 | - | - | - | - | - |
| 281 | MEN1 | c.1365+2T>C | Chr11: 64572504 | NM_000244.3 | Candidate causal | rs1555164115 | - | - | - | - | - |
| 282 | MEN1 | c.1366-2A>G | Chr11: 64572290 | NM_000244.3 | Candidate causal | rs1060499986 | - | - | - | - | - |
| 283 | MEN1 | c.654+3A>G | Chr11: 64575360 | NM_001370259.2 | Uncertain Significance/Candidate causal | - | - | - | - | - | 852 |
| 284 | MEN1 | c.927+1G>C | Chr11: 64574482 | NM_000244.3 | Candidate causal | rs398124437 | - | - | - | - | - |
| 285 | MEN1 | c.1366-1G>A | Chr11: 64572289 | NM_000244.3 | Candidate causal | rs794728629 | - | - | - | - | 853 |
| 286 | MEN1 | c.1366-1G>C | Chr11: 64572289 | NM_000244.3 | Candidate causal | rs794728629 | - | - | - | - | 853 |
| 287 | MEN1 | c.784-1G>A | Chr11: 64574692 | NM_001370259.2 | Candidate causal | - | - | - | - | - | 854 |
| 289 | MEN1 | c.928-2A>G | Chr11: 64573842 | NM_000244.3 | Candidate causal | rs1114167498 | - | - | - | - | 855 |
| 290 | MEN1 | c.461-2A>G | Chr11: 64575573 | NM_000244.3 | Candidate causal | rs886042035 | - | - | - | - | 855 |
| 291 | MEN1 | c.654+1G>T | Chr11: 64575362 | NM_001370259.2 | Candidate causal | rs794728622 | - | - | - | - | 856 |
| 292 | MEN1 | c.784-9G>A | Chr11: 64574700 | NM_001370259.2 | Likely Candidate causal | rs794728625 | - | - | - | - | 857 |
| 293 | MEN1 | c.208+3_208+6del | Chr2: 177188193-177188196 | NM_001370259.2 | Likely Candidate causal | rs1575058847 | - | - | - | - | 858 |
| 294 | NEK8 | c.889+2del | Chr17: 27064756 | NM_178170.3 | Candidate causal | rs1555564214 | - | - | - | - | 859 |
| 295 | NEK8 | c.889+1G>T | Chr17: 27064755 | NM_178170.3 | Candidate causal | rs780247729 | - | 0 | 0 | 0 | 859 |
| 296 | NEK8 | c.828-1G>C | Chr17: 27064692 | NM_178170.3 | Candidate causal | rs749866369 | - | - | - | - | 859 |
| 297 | SLC37A4 | c.1124+1G>C | Chr11: 118895900 | NM_001164277.1 | Candidate causal | rs782630676 | - | 0 | - | 0 | - |
| 298 | SLC37A4 | c.381+1G>T | Chr11: 118898903 | NM_001164277.1 | Candidate causal | - | - | - | - | - | 860 |
| 299 | HMBS | c.160+5G>C | Chr11: 118959422 | NM_000190.4 | Uncertain Significance/Candidate causal | - | - | - | - | - | - |
| 300 | HMBS | c.211-1G>A | Chr11: 118959926 | NM_000190.4 | Candidate causal | rs1565754452 | - | - | - | - | 861 |
| 301 | HMBS | c.266+1G>C | Chr11: 118959983 | NM_000190.4 | Candidate causal | rs1565754565 | - | - | - | - | 862 |
| 302 | HMBS | c.499-1G>A | Chr11: 118962122 | NM_000190.4 | Candidate causal | rs1565756481 | - | - | - | - | 863 |
| 303 | HMBS | c.771+1G>C | Chr11: 118963234 | NM_000190.4 | Candidate causal | rs1565758008 | - | - | - | - | 864 |
| 304 | WNK1 | c.759+12272_760-5774del | Chr12: 875762 | NM_213655.4 | Uncertain Significance/Candidate causal | - | - | - | - | - | - |
| 305 | WNK1 | c.2140-3158G>A | Chr12: 977273 | NM_018979.4 | Candidate causal | - | - | - | - | - | - |
| 306 | WNK1 | c.2140-2795del | Chr12: 977636 | NM_018979.4 | Candidate causal | rs387906331 | - | - | - | - | 865 |
| 307 | WNK1 | c.2140-2568C>T | Chr12: 977863 | NM_018979.4 | Candidate causal | rs111033591 | - | 0 | 0 | 0 | 866 |
| 308 | WNK1 | c.2140-3072G>T | Chr12: 977359 | NM_018979.4 | Candidate causal | - | - | - | - | - | - |
| 309 | WNK1 | c.2140-2392T>G | Chr12: 978039 | NM_018979.4 | Candidate causal | - | - | - | - | - | - |
| 310 | WNK1 | c.2140-2518dup | Chr12: 977911-977912 | NM_018979.4 | Candidate causal | rs137852735 | - | 0 | - | 0 | - |
| 311 | WNK1 | c.2140-2160G>T | Chr12: 978271 | NM_018979.4 | Candidate causal | - | - | - | - | - | - |
| 312 | CDKN1B, GPR19 | c.-454_-451del | Chr12: 12870318 | NM_004064.4 | Likely Candidate causal | rs786201010 | - | - | - | - | 867 |
| 313 | PKP2 | c.2489+1G>A | Chr12: 32949042 | NM_004572.3 | Candidate causal | rs111517471 | - | 0 | 0 | 0 | 129 |
| 314 | CEP290 | c.5728_5731delATTA | Chr12: 88465682 | NM_025114.3 | Candidate causal | rs754728136 | - | - | - | - | 868 |
| 315 | CEP164 | c.4286+1G>T | Chr11: 117282634 | NM_014956.5 | Candidate causal | - | - | - | - | - | - |
| 316 | CEP164 | c.2493+1G>A | Chr11: 117263344 | NM_014956.5 | Candidate causal | rs778819060 | - | 0 | - | - | 869 |
| 317 | CEP164 | c.1317+3G>A | Chr11: 117246510 | NM_014956.5 | Uncertain Significance | rs886038607 | - | - | - | - | 870 |
| 318 | CEP164 | c.1409+10G>A | Chr11: 117251431 | NM_014956.5 | Likely Candidate causal | rs199783386 | - | 0 | 0 | 0 | - |
| 319 | CEP290 | c.5445_5448delAACT | Chr12: 88471612 | NM_025114.3 | Candidate causal | rs749331348 | - | 0 | 0 | - | 868 |
| 320 | CEP290 | c.6271-6_6274del | Chr12: 88456552-88456561 | NM_025114.4 | Candidate causal | - | - | - | - | - | - |
| 321 | CEP290 | c.3104-5T>G | Chr12: 88487757 | NM_025114.4 | Uncertain Significance/Candidate causal | rs1302558061 | - | 0 | 0 | 0 | - |
| 322 | CEP290 | c.3573+2T>C | Chr12: 88484503 | NM_025114.4 | Candidate causal | rs1219277452 | - | 0 | 0 | 0 | 871 |
| 323 | CEP290 | c.4030-2A>G | Chr12: 88481723 | NM_025114.4 | Candidate causal | - | - | - | - | - | 871 |
| 324 | CEP290 | c.1523-1G>T | Chr12: 88512521 | NM_025114.4 | Candidate causal | rs1192112844 | - | - | - | - | 871 |
| 325 | CEP290 | c.1409+10G>A | Chr12:117251431 | NM_014956.5 | Likely Candidate causal | rs199783386 | - | 0 | 0 | 0 | - |
| 326 | CEP290 | c.3310-1_3310delinsAA | Chr12:88486609-88486610 | NM_025114.4 | Candidate causal | - | - | - | - | - | - |
| 327 | CEP290 | c.251-2A>G | Chr12: 88532970 | NM_014956.4 | Candidate causal | rs951979448 | - | 0 | - | 0 | 871 |
| 328 | CEP290 | c.942+1G>C | Chr12:88522722 | NM_014956.4 | Candidate causal | - | - | - | - | - | 871 |
| 329 | CEP290 | c.5709+1G>A | Chr12: 88470998 | NM_014956.4 | Candidate causal | rs759850328 | - | 0 | - | 0 | 871 |
| 330 | CEP290 | c.-8_2del | Chr12: 88535083-88535092 | NM_014956.4 | Candidate causal | - | - | - | - | - | 872 |
| 331 | CEP290 | c.1712-2A>T | Chr12: 88510924 | NM_014956.4 | Candidate causal | rs764551108 | - | 0 | - | - | 871 |
| 332 | CEP290 | c.3461+1G>A | Chr12: 88486457 | NM_014956.4 | Candidate causal | rs766952056 | - | - | - | - | 871 |
| 333 | CEP290 | c.6818_6818+1dup | Chr12: 88452623-88452624 | NM_014956.4 | Candidate causal | rs1060499781 | - | - | - | - | 871 |
| 334 | CEP290 | c.1623+1G>A | Chr12: 88512419 | NM_014956.4 | Candidate causal | rs863225186 | - | 0 | - | 0 | 873 |
| 335 | CEP290 | c.4438-3del | Chr12: 88478632 | NM_014956.4 | Uncertain Significance/ Candidate causal | rs747323414 | - | 0 | 0 | 0 | 874 |
| 336 | CEP290 | c.297+1G>T | Chr12: 88532921 | NM_014956.4 | Candidate causal | rs878853360 | - | 0 | 0 | - | 875 |
| 337 | CEP290 | c.1189+1G>A | Chr12: 88519022 | NM_014956.4 | Candidate causal | - | - | - | - | - | 876 |
| 338 | CEP290 | c.5012+2T>C | Chr12: 88476806 | NM_014956.4 | Candidate causal | rs1369768287 | - | 0 | 0 | 0 | 871 |
| 339 | CEP290 | c.6358-1G>A | Chr12: 88454772 | NM_014956.4 | Candidate causal | rs766670248 | - | 0 | 0 | 0 | 871 |
| 340 | CEP290 | c.6012-2A>G | Chr12: 88462424 | NM_014956.4 | Candidate causal | rs555755221 | - | 0 | 0 | 0 | 877 |
| 341 | CEP290 | c.6645+1G>A | Chr12: 88453674 | NM_014956.4 | Candidate causal | rs201218801 | - | 0 | 0 | 0 | 871 |
| 342 | CEP290 | c.1066-1G>A | Chr12: 88519147 | NM_014956.4 | Candidate causal | rs965522059 | - | 0 | - | - | 878 |
| 343 | CEP290 | c.4813-2A>G | Chr12: 88477009 | NM_014956.4 | Candidate causal | rs369523378 | - | 0 | 0 | 0 | 871 |
| 344 | CEP290 | c.2052+1_2052+2del | Chr12: 88508195-88508196 | NM_014956.4 | Candidate causal | rs747835249 | - | 0 | - | 0 | 879 |
| 345 | CEP290 | c.180+1G>A | Chr12: 88534732 | NM_014956.4 | Candidate causal | rs758593134 | - | - | - | - | 871 |
| 346 | CEP290 | c.5587-1G>C | Chr12: 88471122 | NM_014956.4 | Candidate causal | rs968692633 | - | 0 | 0 | - | 880 |
| 347 | CEP290 | c.4437+1G>A | Chr12: 88479815 | NM_025114.4 | Candidate causal | rs760915898 | - | 0 | 0 | 0 | 871 |
| 348 | CEP290 | c.2991+1655A>G | Chr12: 88494960 | NM_014956.4 | Likely Candidate causal | rs281865192 | - | 0 | 0 | 0 | 871 |
| 349 | CEP290 | c.4438-315_7246del | Chr12: 88443158 | NM_025114.3 | Candidate causal | - | - | - | - | - | 881 |
| 350 | TRIP11 | c.4557+1G>T | Chr14: 92469762 | NM_004239.4 | Candidate causal | rs1555386022 | - | - | - | - | - |
| 351 | PLIN1 | c.1210-1G>T | Chr15: 90209174 | NM_002666.5 | Uncertain Significance/Candidate causal | rs1567075176 | - | - | - | - | 359 |
| 352 | PLIN1 | c.1210-1del | Chr15: 90209174 | NM_002666.5 | Uncertain Significance/Candidate causal | - | - | - | - | - | - |
| 353 | PLIN1 | c.964-1G>A | Chr15: 90210413 | NM_002666.5 | Uncertain Significance | rs201579932 | - | 0 | - | - | 882 |
| 354 | PKD1, MIR1225 | c.12445-2A>C | Chr16: 2140197 | NM_001009944.3 | Candidate causal | rs1555444334 | - | - | - | - | 838 |
| 355 | PKD1, LOC105371049 | c.11156+1G>C | Chr16: 2142954 | NM_001009944.3 | Candidate causal | rs1596485727 | - | 0 | - | 0 | - |
| 356 | PKD1 | c.10220+2_10220+3del | Chr16: 2147726 | NM_001009944.3 | Candidate causal | rs1114167366 | - | - | - | - | - |
| 357 | PKD1 | c.3296-1G>A | Chr16: 2161873 | NM_001009944.3 | Candidate causal | rs1567204631 | - | - | - | - | 743 |
| 358 | PKD1 | c.1723-1G>A | Chr16: 2166120 | NM_001009944.3 | Candidate causal | rs1596582948 | - | - | - | -- | 883 |
| 359 | GLIS2 | c.775+1G>T | Chr16: 4385395 | NM_001318918.1 | Candidate causal | rs878855335 | - | - | - | - | - |
| 360 | ABCC6 | c.3736-1G>A | Chr16: 16251667 | NM_001171.5 | Candidate causal | rs63750273 | - | 0 | 0 | 0 | 839 |
| 361 | ABCC6 | c.2787+1G>T | Chr16: 16267140 | NM_001171.5 | Candidate causal | rs72664209 | - | 0 | 0 | 0 | 884 |
| 362 | HSD11B2 | c.664+14C>T | Chr16: 67470059 | NM_000196.4 | Uncertain Significant/Candidate causal | rs376023420 | 0 | 0 | - | - | 885 |
| 363 | MPO | c.2031-2A>C | Chr17: 56348226 | NM_000250.2 | Candidate causal | rs35897051 | 0 | 0 | 0 | 0 | 251 |
| 364 | TBX4 | c.281+1G>T | Chr17: 59534993 | NM_001321120.2 | Candidate causal | rs1555881112 | - | - | - | - | - |
| 365 | TBX4 | c.792-1G>C | Chr17: 59557450 | NM_001321120.2 | Candidate causal | rs1603255224 | - | - | - | - | 886 |
| 366 | SMAD4 | c.1308+2T>C | Chr18: 48593559 | NM_005359.5 | Candidate causal | rs1555686624 | - | - | - | - | 638 |
| 367 | MYH7B | c.750+1G>A | Chr20: 33570359 | NM_020884.5 | Candidate causal | rs1050997719 | - | 0 | 0 | - | 245 |
| 368 | PTGIS | c.1358+2T>C | Chr20: 48127563 | NM_000961.4 | Candidate causal | rs13306026 | - | 0 | 0 | 0 | 887 |
| 369 | ADA2 | c.973-2A>G | Chr22: 17669339 | NM_001282225.2 | Candidate causal | rs139750129 | - | 0 | 0 | 0 | 888 |
| 370 | ADA2 | c.882-2A>G | Chr22: 17670924 | NM_001282225.2 | Candidate causal | - | - | - | - | - | 889 |
| 371 | ADA2 | c.754-2A>G | Chr22: 17672702 | NM_001282225.2 | Candidate causal | - | - | - | - | - | 890 |
| 372 | ABCA3 | c.614-2A>G | Chr16: 2369843 | NM_001282225.2 | Candidate causal | rs773223403 | - | - | - | - | - |
| 373 | ABCC2 | c.1967+1G>A | Chr10: 101571360 | NM_000392.5 | Candidate causal | rs146405172 | - | 0 | 0 | 0 | - |
| 374 | ABCC6 | c.4403+1G>T | Chr16: 16244434 | NM_001171.6 | Candidate causal | rs1006994885 | - | 0 | 0 | - | - |
| 375 | ABCC6 | c.3876_3882+1del | Chr16: 16251573-16251574 | NM_001171.6 | Candidate causal | rs1555507903 | - | - | - | - | - |
| 376 | ABCC6 | c.36+1G>T | Chr16: 16317255 | NM_001171.6 | Candidate causal | rs1555523841 | - | - | - | - | - |
| 377 | ABCC6 | c.1179_1338+2del | Chr16: 16291876-16292037 | NM_001171.6 | Candidate causal | rs1555517253 | - | - | - | - | - |
| 378 | ABCC6 | c.3633+1G>A | Chr16: 16255294 | NM_001171.6 | Candidate causal | rs1555508604 | - | - | - | - | - |
| 379 | ABCC6 | c.600+1G>A | Chr16: 16308180 | NM_001171.6 | Candidate causal | rs1187315015 | - | 0 | 0 | - | - |
| 380 | ABCC6 | c.601-2A>G | Chr16: 16306105 | NM_001171.6 | Candidate causal | rs1555520991 | - | 0 | - | 0 | - |
| 381 | ABCC6 | c.1892_1943+26del | Chr16: 16278790-16278867 | NM_001171.6 | Candidate causal | rs1555513581 | - | - | - | - | - |
| 382 | ABCC6 | c.2416-1_2416del | Chr16: 16271483-16271484 | NM_001171.6 | Candidate causal | rs111113625 | - | - | - | - | - |
| 383 | ABCC6 | c.2071-1G>A | Chr16: 16276446 | NM_001171.6 | Candidate causal | - | - | - | - | - | - |
| 384 | ABCC6 | c.1635+1G>T | Chr16: 16284020 | NM_001171.6 | Candidate causal | rs1264741133 | - | - | - | - | 891 |
| 385 | ABCC6 | c.1944-1G>C | Chr16: 16276788 | NM_001171.6 | Candidate causal | rs1438851867 | - | 0 | - | 0 | 892 |
| 386 | ABCC6 | c.-197_-193del | Chr16: 16315544-16315548 | NM_001351800.1 | Candidate causal | rs1555523438 | - | - | - | - | 893 |
| 387 | ABCC6 | c.1091_1338+3149del | Chr16: 16288729-16295943 | NM_001171.6 | Candidate causal | rs72664204 | - | 0 | 0 | 0 | 894 |
| 388 | ABCC6 | c.998+2_998+3del | Chr16: 16297264-16297265 | NM_001171.6 | Candidate causal | rs72664204 | - | 0 | 0 | 0 | 894 |
| 389 | ABCC6 | c.3507-1G>A | Chr16: 16255422 | NM_001171.6 | Candidate causal | rs72664210 | - | 0 | 0 | 0 | 895 |
| 390 | ABCC6 | c.220-1G>C | Chr16: 16313805 | NM_001171.6 | Candidate causal | rs72664203 | - | - | - | - | 896 |
| 391 | ABCC6 | c.998+2del | Chr16: 16297265 | NM_001171.6 | Candidate causal | rs72664205 | - | - | - | - | 897 |
| 392 | ABCC6 | c.3506+2_3506+5del | Chr16: 16256845-16256848 | NM_001171.6 | Candidate causal | rs72664211 | - | 0 | - | 0 | 898 |
| 393 | ABCC6 | c.37-1G>A | Chr16: 16315689 | NM_001171.6 | Candidate causal | rs72657702 | - | 0 | 0 | 0 | 898 |
| 394 | ABCC6 | c.-269del | Chr16: 16315620 | NM_001351800.1 | Candidate causal | rs72664223 | - | 0 | - | - | 895 |
| 395 | ABCC6 | c.3306+1del | Chr16: 16259479 | NM_001171.6 | Candidate causal | rs797045078 | - | - | - | - | - |
| 396 | ABCC6 | c.4209-2A>C | Chr16: 16244631 | NM_001171.6 | Candidate causal | rs1555506740 | - | 0 | - | - | - |
| 397 | ABCC6 | c.3883-6G>A | Chr16: 16248894 | NM_001171.6 | Uncertain Significance/Candidate causal | rs72664214 | - | 0 | 0 | 0 | 895 |
| 398 | ABCC6 | c.1868-5T>G | Chr16: 16278896 | NM_001171.6 | Uncertain Significance/Candidate causal | rs72664207 | - | 0 | 0 | 0 | 899 |
| 399 | ABCC6 | c.2248-2_2248-1del | Chr16: 16272823-16272824 | NM_001171.6 | Candidate causal | rs111113624 | - | 0 | 0 | 0 | 900 |
| 400 | ANKS6 | c.907+2T>A | Chr9: 101547112 | NM_173551.5 | Candidate causal | rs1438673595 | - | 0 | - | - | 901 |
| 401 | ANKS6 | c.1973-3C>G | Chr91530535 | NM_001171.5 | Likely Candidate causal | rs397514257 | - | 0 | 0 | 0 | 902 |
| 402 | ASL | c.1062+1G>T | Chr7: 65554683 | NM_000048.4 | Candidate causal | rs1554327825 | - | - | - | - | - |
| 403 | ASL | c.1249_1250+12del | Chr7: 65557644-65557657 | NM_000048.4 | Candidate causal | rs1554328381 | - | - | - | - | - |
| 404 | ASL | c.446+2T>C | Chr7: 65548163 | NM_000048.4 | Candidate causal | rs1268519003 | - | 0 | 0 | - | - |
| 405 | ASL | c.13-1G>C | Chr7: 65546789 | NM_000048.4 | Candidate causal | rs1554326239 | - | - | - | - | - |
| 406 | ASL | c.291+1G>T | Chr7: 65547439 | NM_000048.4 | Candidate causal | rs201523601 | - | 0 | - | 0 | - |
| 407 | ASL | c.602+1G>T | Chr7: 65551809 | NM_000048.4 | Candidate causal | - | - | - | - | - | - |
| 408 | ASL | c.919-1G>A | Chr7: 65554262 | NM_000048.4 | Candidate causal | - | - | - | - | - | 903 |
| 409 | ASL | c.603-1G>C | Chr7: 65552320 | NM_000048.4 | Candidate causal | rs1584028936 | - | - | - | - | 903 |
| 410 | ASL | c.719-1G>C | Chr7: 65553793 | NM_000048.4 | Candidate causal | rs369828060 | - | 0 | 0 | 0 | 904 |
| 411 | ASL | c.918+5G>A | Chr7: 65554167 | NM_000048.4 | Uncertain Significance | rs781331391 | - | 0 | - | - | 903 |
| 412 | ASL | c.718+5G>A | Chr7: 65552783 | NM_000048.4 | Uncertain Significance | rs869312990 | - | 0 | - | - | 905 |
| 413 | ASL | c.602+1G>A | Chr7: 65551809 | NM_000048.4 | Candidate causal | rs398123127 | - | 0 | 0 | 0 | 903 |
| 414 | BBS1 | c.-3_37del | Chr11: 66278117-66278156 | NM_024649.5 | Candidate causal | rs113994178 | - | - | - | - | - |
| 415 | BBS1 | c.480-2A>C | Chr11: 66283162 | NM_024649.5 | Candidate causal | - | - | - | - | - | 266 |
| 416 | BBS1 | c.723+1G>C | Chr11: 66287220 | NM_024649.5 | Candidate causal | rs1295318869 | - | - | - | - | 266 |
| 417 | BBS1 | c.432+1G>A | Chr11: 66282150 | NM_024649.5 | Candidate causal | rs587777829 | - | 0 | - | 0 | 266 |
| 418 | BBS1|ZDHHC24 | c.1340-2A>G | Chr11: 66297288 | NM_024649.5 | Candidate causal | rs113994180 | - | 0 | - | - | 906 |
| 419 | BBS1|ZDHHC24 | c.1111-2A>G | Chr11: 66293592 | NM_024649.5 | Candidate causal | rs761969357 | - | 0 | 0 | 0 | 266 |
| 420 | BBS1|ZDHHC24 | c.831-2A>G | Chr11: 66290925 | NM_024649.5 | Candidate causal | rs1057517332 | - | 0 | - | - | 266 |
| 421 | BBS1|ZDHHC24 | c.831-3C>G | Chr11: 66290924 | NM_024649.5 | Uncertain Significance | rs113994179 | - | 0 | - | 0 | 906 |
| 422 | BBS1|ZDHHC24 | c.1110+3G>C | Chr11: 66291356 | NM_024649.5 | Uncertain Significance/Candidate causal | rs762276925 | - | 0 | - | 0 | 268 |
| 423 | BBS1|ZDHHC24 | c.1473+4A>G | Chr11: 66297427 | NM_024649.5 | Uncertain Significance/Candidate causal | rs1486200900 | - | 0 | 0 | - | 269 |
| 424 | BBS1|ZDHHC24 | c.951+1G>A | Chr11: 66291048 | NM_024649.5 | Candidate causal | rs746875134 | - | 0 | 0 | - | 270 |
| 425 | BBS2 | c.941-1G>C | Chr16: 56536369 | NM_024649.5 | Candidate causal | - | - | - | - | - | 907 |
| 426 | BBS2 | c.1398-2A>G | Chr16: 56533821 | NM_024649.5 | Candidate causal | rs767609303 | - | - | 0 | - | 907 |
| 427 | BBS2 | c.804+1_804+14del | Chr16: 56539848-56539861 | NM_024649.5 | Candidate causal | rs1202682571 | - | 0 | 0 | 0 | 907 |
| 428 | BBS2 | c.718-2A>C | Chr16: 56539950 | NM_024649.5 | Candidate causal | - | - | - | - | - | 907 |
| 429 | BBS2 | c.941-2A>C | Chr16: 56536370 | NM_024649.5 | Candidate causal | rs878962682 | - | - | - | - | 907 |
| 430 | BBS2 | c.534+1G>T | Chr16: 56544770 | NM_031885.4 | Candidate causal | rs773862084 | - | 0 | 0 | 0 | 907 |
| 431 | BBS4 | c.76+1G>T | Chr15: 72987570 | NM_024649.5 | Candidate causal | rs1465437164 | - | 0 | 0 | - | 271 |
| 432 | BBS4 | c.406-2A>C | Chr15: 73015133 | NM_024649.5 | Candidate causal | rs113994191 | - | 0 | - | - | 908 |
| 433 | BBS4 | c.333-1G>C | Chr15: 73009118 | NM_024649.5 | Candidate causal | - | - | - | - | - | 908 |
| 434 | BBS4 | c.1248+1G>T | Chr15: 73028308 | NM_024649.5 | Candidate causal | - | - | - | - | - | 908 |
| 435 | BBS4 | c.157-3C>G | Chr15: 73004582 | NM_024649.5 | Uncertain Significance/Candidate causal | rs1567412639 | - | 0 | - | - | 271 |
| 436 | BBS4 | c.220+1G>C | Chr15: 73004649 | NM_024649.5 | Candidate causal | rs113994190 | - | 0 | - | 0 | 908 |
| 437 | BBS4 | c.77-216del | Chr15: 73001821 | NM_024649.5 | Uncertain Significance/Candidate causal | rs113994189 | - | - | - | - | 908 |
| 438 | BBS4 | c.1106+2T>A | Chr15: 73027525 | NM_024649.5 | Candidate causal | rs886041464 | - | - | - | - | 272 |
| 439 | BBS4 | c.712-1G>A | Chr15: 73023645 | NM_024649.5 | Candidate causal | rs377031435 | - | 0 | 0 | 0 | 274 |
| 440 | BBS4 | c.157-2A>G | Chr15: 73004583 | NM_024649.5 | Candidate causal | rs113994192 | - | 0 | - | - | 273 |
| 441 | BBS5 | c.143-1G>A | Chr2: 170343578 | NM_152384.3 | Candidate causal | - | - | - | - | - | 273 |
| 442 | BBS5 | c.817-1G>A | Chr2: 170359604 | NM_152384.3 | Candidate causal | rs1466289570 | - | 0 | - | 0 | 909 |
| 443 | BBS5 | c.143-1G>C | Chr2: 170343578 | NM_152384.3 | Candidate causal | rs1054138918 | - | 0 | - | 0 | 909 |
| 444 | BBS5 | c.619-1G>C | Chr2: 170354136 | NM_152384.3 | Candidate causal | rs753234582 | - | 0 | 0 | 0 | 275 |
| 445 | BBS7 | c.1677-490_1804del | Chr4: 122749643-122750373 | NM_152384.3 | Candidate causal | rs1578522416 | - | - | - | - | 276 |
| 446 | BBS7 | c.1371+1G>A | Chr4: 122760785 | NM_152384.3 | Candidate causal | rs1578537379 | - | - | - | - | 910 |
| 447 | BBS7 | c.1786+1G>T | Chr4: 122749773 | NM_152384.3 | Candidate causal | rs1560638613 | - | - | - | - | 910 |
| 448 | BBS9 | c.263+4A>G | Chr7: 33192467 | NM_152384.3 | Uncertain Significance/Candidate causal | rs370916293 | - | 0 | 0 | - | 271 |
| 449 | BBS9 | c.113-2A>G | Chr7: 33192311 | NM_152384.3 | Candidate causal | rs1562686929 | - | - | - | - | 271 |
| 450 | BBS9 | c.2115+1G>A | Chr7: 33427757 | NM_152384.3 | Candidate causal | rs886039801 | - | - | - | - | 819 |
| 451 | BBS9 | c.1552+2T>A | Chr7: 33392487 | NM_152384.3 | Candidate causal | rs1563049863 | - | 0 | - | - | 819 |
| 452 | BBS9 | c.1789+1G>T | Chr7: 33407475 | NM_152384.3 | Candidate causal | rs201938124 | - | 0 | 0 | - | 911 |
| 453 | BBS9 | c.263+1G>A | Chr7: 33192464 | NM_152384.3 | Candidate causal | rs137962929 | - | 0 | 0 | 0 | 277 |
| 454 | DYNC2H1 | c.10042+2T>G | Chr11: 103116105 | NM_001377.3 | Candidate causal | rs1261505725 | - | 0 | 0 | 0 | 912 |
| 455 | ENG | c.*111G>T | Chr9: 130578085 | NM_000118.3 | Candidate causal | rs1085307436 | - | - | - | - | 797 |
| 456 | ENG|LOC102723566 | c.1742-22T>C | Chr9: 130578354 | NM_000118.3 | Uncertain Significance/Candidate causal | rs1085307434 | - | - | - | - | 913 |
| 457 | FAM186B | c.506-2A>G | Chr12: 49994919 | NM_032130.3 | Uncertain Significance/Candidate causal | rs549662742 | 0 | 0 | 0 | 0 | 914 |
| 458 | G6PC1 | c.447-1G>A | Chr17: 41061319 | NM_000151.4 | Candidate causal | rs1411037881 | - | 0 | 0 | 0 | - |
| 459 | G6PC1 | c.230+1G>C | Chr17: 41053124 | NM_000151.4 | Candidate causal | rs863224023 | - | 0 | - | 0 | - |
| 460 | G6PC1 | c.231-1G>A | Chr17: 41055947 | NM_000151.4 | Candidate causal | rs1555559279 | - | 0 | 0 | 0 | 915 |
| 461 | G6PC1 | c.230+4A>G | Chr17: 41053127 | NM_000151.4 | Uncertain Significance/Candidate causal | rs587776757 | - | 0 | - | - | 916 |
| 462 | G6PC1 | c.446+1G>C | Chr17: 41059646 | NM_000151.4 | Candidate causal | rs1597989983 | - | 0 | - | 0 | 917 |
| 463 | G6PC1 | c.446+2T>C | Chr17: 41059647 | NM_000151.4 | Candidate causal | rs1597989985 | - | - | - | - | 917 |
| 464 | G6PC3 | c.*50C>T | Chr17: 42153127 | NM_001319945.2 | Uncertain Significance/Candidate causal | rs765927570 | - | 0 | 0 | 0 | 918 |
| 465 | IFT27 | c.352+1G>T | Chr22: 37159962 | NM_001177701.3 | Uncertain Significance/Candidate causal | rs780659194 | - | 0 | - | - | 279 |
| 466 | INVS | c.1078+1G>A | Chr9: 103009070 | NM_014425.5 | Candidate causal | rs375416014 | - | 0 | 0 | 0 | 919 |
| 467 | INVS | c.615+1G>A | Chr9: 102992110 | NM_014425.5 | Candidate causal | rs955421639 | - | 0 | - | 0 | - |
| 468 | INVS | c.2786+2T>C | Chr9: 103055327 | NM_014425.5 | Candidate causal | rs1322951938 | - | 0 | - | - | - |
| 469 | NPHP4 | c.518-1G>C | Chr1: 6022010 | NM_014425.5 | Candidate causal | - | - | - | - | - | 820 |
| 470 | NPHP4 | c.517+1G>A | Chr1: 6027358 | NM_014425.5 | Candidate causal | rs1419875412 | - | 0 | - | 0 | 820 |
| 471 | NPHP4 | c.1956-2A>C | Chr1: 5964866 | NM_014425.5 | Candidate causal | - | - | - | - | - | 820 |
| 472 | NPHP4 | c.2611+1G>C | Chr1: 5940173 | NM_014425.5 | Candidate causal | rs374141736 | - | 0 | 0 | 0 | 920 |
| 473 | NPHP4 | c.2304+1G>A | Chr1: 5950927 | NM_014425.5 | Candidate causal | rs757412845 | - | 0 | - | - | 920 |
| 474 | NPHP4 | c.280-1G>C | Chr1: 6029320 | NM_014425.5 | Candidate causal | - | - | - | - | - | 920 |
| 475 | PKD1 | c.12004-7_12012dup | Chr16: 2140800-2140801 | NM_001009944.3 | Candidate causal | rs1596475487 | - | - | - | - | - |
| 476 | PKD1 | c.8791+1G>A | Chr16: 2153266 | NM_001009944.3 | Candidate causal | - | - | - | - | - | - |
| 477 | PKD1 | c.8162-2A>G | Chr16: 2153898 | NM_001009944.3 | Candidate causal | - | - | - | - | - | - |
| 478 | PKD1 | c.288-2A>G | Chr16: 2169188 | NM_001009944.3 | Candidate causal | - | - | - | - | - | 921 |
| 479 | PKD1 | c.2097+2T>C | Chr16: 2165377 | NM_001009944.3 | Candidate causal | - | - | - | - | - | - |
| 480 | PKD1 | c.1606+5G>A | Chr16: 2166829 | NM_001009944.3 | Uncertain Significance/Candidate causal | - | - | - | - | - | - |
| 481 | PKD1 | c.12139-2A>C | Chr16: 2140593 | NM_001009944.3 | Candidate causal | - | - | - | - | - | - |
| 482 | PKD1 | c.8791+40_10050+3del | Chr16: 2149642-2153227 | NM_001009944.3 | Candidate causal | - | - | - | - | - | - |
| 483 | PKD1 | c.9568+1G>C | Chr16: 2150396 | NM_001009944.3 | Candidate causal | - | - | - | - | - | 820 |
| 484 | PKD1 | c.3295+1G>T | Chr16: 2162340 | NM_001009944.3 | Candidate causal | - | - | - | - | - | 820 |
| 485 | PKD1 | c.9202-16G>A | Chr16: 2152273 | NM_001009944.3 | Likely Candidate causal | rs1389523126 | - | - | - | - | 820 |
| 486 | PKD1 | c.1201+1G>A | Chr16: 2167791 | NM_001009944.3 | Candidate causal | rs1596588978 | - | - | - | - | - |
| 487 | PKD1 | c.8017-1G>C | Chr16: 2154644 | NM_001009944.3 | Candidate causal | - | - | - | - | - | - |
| 488 | PKD1 | c.1202-2A>T | Chr16: 2167675 | NM_001009944.3 | Candidate causal | - | - | - | - | - | 820 |
| 489 | PKD1 | c.6916-9G>A | Chr16: 2158042 | NM_001009944.3 | Likely Candidate causal | rs1567190244 | - | 0 | - | - | 922 |
| 490 | PKD1 | c.11713-1G>A | Chr16: 2141176 | NM_001009944.3 | Candidate causal | rs867092741 | - | 0 | - | - | 923 |
| 491 | PKD1|LOC105371049 | c.11017-47_11022del | Chr16: 2143089 - 2143141 | NM_001009944.3 | Candidate causal | - | - | - | - | - | - |
| 492 | PKD1|LOC105371049 | c.10821+1G>A | Chr16: 2143811 | NM_001009944.3 | Candidate causal | rs1596488823 | - | - | - | - | 924 |
| 493 | PKD1|LOC105035371049 | c.11411+1G>C | Chr16: 2142047 | NM_001009944.3 | Candidate causal | - | - | - | - | - | - |
| 494 | PKD5041|LOC105371049 | c.10822-1G>C | Chr16: 2143740 | NM_001009944.3 | Candidate causal | - | - | - | - | - | 820 |
| 495 | PKD1|MIR1225 | c.12444+1G>A | Chr16: 2140285 | NM_001009944.3 | Candidate causal | - | - | - | - | - | 925 |
| 496 | RMND1 | c.829_830+2del | Chr6: 151748615-151748618 | NM_017909.4 | Candidate causal | - | - | - | - | - | 926 |
| 497 | SLC37A4 | c.985+1G>A | Chr11: 118896676 | NM_001164277.1 | Candidate causal | - | - | - | - | - | - |
| 498 | SLC37A4 | c.986-3_989del | Chr11: 118896036-118896042 | NM_001164277.1 | Candidate causal | rs1555190559 | - | - | - | - | - |
| 499 | SLC37A4 | c.1124-1G>A | Chr11: 118895787 | NM_001164277.1 | Candidate causal | rs782202675 | - | - | - | - | - |
| 500 | SLC37A4 | c.871-2A>C | Chr11: 118896792 | NM_001164277.1 | Candidate causal | rs920196110 | - | - | - | - | - |
| 501 | SLC37A4 | c.148+2T>C | Chr11:118899930 | NM_001164277.1 | Candidate causal | rs1449998297 | - | - | 0 | - | - |
| 502 | SLC37A4 | c.529_533del | Chr11: 118898430 | NM_001164277.1 | Candidate causal | rs1170938506 | - | 0 | 0.01 | - | - |
| 503 | SLC37A4 | c.382-1del | Chr11:118898583 | NM_001164277.1 | Candidate causal | rs1555191406 | - | - | - | - | 927 |
| 504 | SLC37A4 | c.381+1G>A | Chr11: 118898903 | NM_001164277.1 | Candidate causal | rs786204637 | - | - | - | - | 928 |
| 505 | SLC37A4 | c.148+1G>T | Chr11: 118899931 | NM_001164277.1 | Candidate causal | - | - | - | - | - | 929 |
| 506 | SLC37A4 | c.786-1G>A | Chr11: 118897399 | NM_001164277.1 | Candidate causal | - | - | - | - | - | 930 |
| 507 | SLC37A4 | c.784+2T>G | Chr11: 118897645 | NM_001164277.1 | Candidate causal | rs1592111559 | - | - | - | - | 930 |
| 508 | SLC37A4 | c.1123+1G>C | Chr11: 118895900 | NM_001164277.1 | Candidate causal | rs782630676 | - | 0 | - | 0 | 244 |
| 509 | SLC37A4 | c.381+2T>G | Chr11: 118898902 | NM_001164277.1 | Candidate causal | rs782645078 | - | - | 0 | - | 931 |
| 510 | TBX4 | c.702+1G>A | Chr17: 59556141 | NM_001321120.2 | Candidate causal | rs1555883342 | - | - | - | - | 711 |
| 511 | TBX6 | c.-48-240A>G | Chr11: 118897399 | NM_001164277.1 | Candidate causal | - | - | - |  |  |  |
| 512 | TMEM67 | c.1057_1065+2del | Chr8: 94793960-94793970 | NM_153704.6 | Candidate causal | - | - | - | - | - | 820 |
| 513 | ASTN2 | c.2806+26456_2806+26462del | Chr9: 119461588-119461594 | NM_001365068.1 | Candidate causal | rs886044106 | - | 0 | 0 | 0 | - |
| 514 | ASTN2 | c.2806+26922del | Chr9: 119461128 | NM_001365068.1 | Candidate causal | rs759376012 | - | 0 | - | 0 | 244 |
| 515 | ASTN2 | c.2806+26570C>T | Chr9: 119461480 | NM_001365068.1 | Candidate causal | rs111033570 | - | 0 | - | 0 | 932 |
| 516 | TTC21B | c.3263+1G>A | Chr2: 166746988 | NM_024753.5 | Candidate causal | rs1574070787 | - | - | - | - | 933 |
| 517 | TTC21B | c.2869-2A>G | Chr2: 166755279 | NM_024753.5 | Candidate causal | rs1553508246 | - | 0 | - | 0 | 933 |
| 518 | TTC21B | c.2868+1G>T | Chr2: 166756279 | NM_024753.5 | Candidate causal | - | - | - | - | - | 790 |
| 519 | TTC8 | c.1049+2_1049+4del | Chr14: 89336544-89336546 | NM_144596.3 | Candidate causal | rs587777807 | - | - | - | - | 354 |
| 520 | VHL | c.311_340+20del | Chr3: 10183841-10183890 | NM_000551.3 | Candidate causal | rs869025629 | - | - | - | - | - |
| 521 | VHL | c.340+1G>C | Chr3: 10183872 | NM_000551.3 | Candidate causal | rs730882032 | - | - | - | - | 934 |
| 522 | VHL | c.-75_-55del | Chr3: 10183457-10183477 | NM_000551.3 | Uncertain Significance/Candidate causal | rs727503744 | - | - | - | - | 935 |
| 523 | VHL | c.341-3221_341-3220dup | Chr3: 8 10188249-10188250 | NM_198156.3 | Candidate causal | rs1559428128 | - | - | - | - | - |
| 524 | VHL|LOC107303340 | c.464-1226_*2771del | Chr3: 10190242-10194417 | NM_000551.4 | Likely Candidate causal | - | - | - | - | - | - |
| 525 | VHL|LOC107303340 | c.463+1008_*2803del | Chr3: 10189293-10194417 | NM_000551.4 | Likely Candidate causal | - | - | - | - | - | - |
| 526 | VHL|LOC107303340 | c.340+307_464-1191del | Chr3: 10184160-10190262 | NM_000551.4 | Candidate causal | - | - | - | - | - | - |
| 527 | VHL|LOC107303340 | c.340+283_463+499del | Chr3: 10184144-10188809 | NM_000551.4 | Candidate causal | - | - | - | - | - | - |
| 528 | VHL|LOC107303340 | c.340+221_464-1411del | Chr3: 10184144-10188809 | NM_000551.4 | Candidate causal | - | - | - | - | - | - |
| 529 | VHL|LOC107303340 | c.463+439_*991del | Chr3: 10188739-10192620 | NM_000551.4 | Likely Candidate causal | - | - | - | - | - | - |
| 530 | VHL|LOC107303340 | c.341-49_*2815del | Chr3: 10188142-10194457 | NM_000551.4 | Likely Candidate causal | - | - | - | - | - | - |
| 531 | VHL|LOC107303340 | c.341-279_464-614del | Chr3: 10187905-10190843 | NM_000551.4 | Candidate causal | - | - | - | - | - | - |
| 532 | VHL|LOC107303340 | c.341-929_*3191del | Chr3: 10187264-10194835 | NM_000551.4 | Likely Candidate causal | - | - | - | - | - | - |
| 533 | VHL|LOC107303340 | c.341-1382_*3401del | Chr3: 10186803-10195037 | NM_000551.4 | Likely Candidate causal | - | - | - | - | - | - |
| 534 | VHL|LOC107303340 | c.341-1406_*3377del | Chr3: 10186788-10195022 | NM_000551.4 | Likely Candidate causal | - | - | - | - | - | - |
| 535 | VHL|LOC107303340 | c.341-1857_464-1328del | Chr3: 10186337-10190139 | NM_000551.4 | Candidate causal | - | - | - | - | - | - |
| 536 | VHL|LOC107303340 | c.340+1580_464-1477del | Chr3: 10185451-10189994 | NM_000551.4 | Candidate causal | - | - | - | - | - | - |
| 537 | VHL|LOC107303340 | c.340+1019_463+450del | Chr3: 10184883-10188763 | NM_000551.4 | Candidate causal | - | - | - | - | - | - |
| 538 | VHL|LOC107303340 | c.340+994_*2333del | Chr3: 10184863-10193980 | NM_000551.4 | Likely Candidate causal | - | - | - | - | - | - |
| 539 | VHL|LOC107303340 | c.340+428_464-1203del | Chr3: 10184256-10190225 | NM_000551.4 | Candidate causal | - | - | - | - | - | - |
| 540 | VHL|LOC107303340 | c.340+365_464-909del | Chr3: 10184219-10190545 | NM_000551.4 | Candidate causal | - | - | - | - | - | - |
| 541 | VHL|LOC107303340 | c.341-3209A>C | Chr3: 10188262 | NM_198156.3 | Likely Candidate causal | rs119103278 | - | - | - | - | 936 |
| 542 | VHL|LOC107303340 | c.463+2T>G | Chr3: 10188322 | NM_000551.4 | Candidate causal | rs5030814 | - | - | - | - | 937 |
| 543 | VHL|LOC107303340 | c.340+617C>G | Chr3: 10186788-10195022 | NM_000551.4 | Candidate causal | - | - | - |  |  | - |
| 544 | VHL|LOC107303340 | c.341-3T>G | Chr3: 10188195 | NM_000551.3 | Uncertain Significance | rs1131690965 | - | - | - | - | 938 |
| 545 | VHL|LOC107303340 | c.341-25_370dup | Chr3:10188172-10188173 | NM_000551.3 | Candidate causal | rs1553619923 | - | - | - | - | - |
| 546 | VHL|LOC107303340 | c.341-3192dup | Chr3: 10188277-10188278 | NM_000551.3 | Candidate causal | rs1553619976 | - | - | - | - | 939 |
| 547 | VHL|LOC107303340 | c.340+770T>C | Chr3: 10184641 | NM_000551.4 | Likely Candidate causal | rs1346312258 | - | 0 | 0 | 0 | 940 |
| 548 | WDPCP | c.633+2T>C | Chr2: 63664553 | NM_015910.7 | Candidate causal | rs763299856 | - | 0 | - | 0 | 941 |
| 549 | WDPCP | c.1916-2A>G | Chr2: 63401969 | NM_015910.7 | Candidate causal | rs763299856 | - | 0 | - | 0 | 941 |
| 550 | WDPCP | c.208+1G>A | Chr2: 63714580 | NM_015910.7 | Candidate causal | - | - | - | - | - | 356 |
| 551 | WDR19 | c.3184-2A>C | Chr4: 39267681 | NM_025132.4 | Candidate causal | rs1020915921 | - | 0 | - | 0 | - |
| 552 | WDR19 | c.3565+1G>A | Chr4: 39274682 | NM_025132.4 | Candidate causal | rs587777352 | - | 0 | 0 | 0 | 790 |

Table 10. The candidate causal/likely candidate causal non-coding variants in Arrhythmia.

| No. | Gene | Variant | Genomic location (hg19) | Transcript | Classification | dbSNP | Frequency of variants (%) | | | | Ref. |
| --- | --- | --- | --- | --- | --- | --- | --- | --- | --- | --- | --- |
|  |  |  |  |  |  |  | **Iranome** | **1000 Genome** | **genomAD** | **TOPMED** |  |
| 1 | LMNA | c.810+1G>C | Chr1: 156104767 | NM_170707.4 | Candidate causal | rs267607632 | - | - | - | - | 6 |
| 2 | SLC25A20 | c.200_326+1del | Chr3: 48921430 | NM_000387.6 | Candidate causal | rs1553686314 | - | - | - | - | 942 |
| 3 | TECRL | c.331+1G>A | Chr4: 65194229 | NM_001010874.5 | Uncertain Significance/Candidate causal | rs1057517699 | - | - | - | - | 943 |
| 4 | FBXL4 | c.1389+3_1389+6del | Chr6: 99328423 | NM_001278716.2 | Uncertain Significance/Candidate causal | rs1554216735 | - | - | - | - | 944 |
| 5 | FBXL4 | c.859-1G>T | Chr6: 99353547 | NM_001278716.2 | Candidate causal | rs368965675 | - | 0 | 0 | 0 | - |
| 6 | FBXL4 | c.858+1G>T | Chr6: 99365249 | NM_001278716.2 | Candidate causal | rs1394080480 | - | - | - | - | - |
| 7 | FBXL4 | c.513-1G>A | Chr6: 99365596 | NM_001278716.2 | Candidate causal | rs1554221258 | - | - | - | - | 660 |
| 8 | TRDN | c.1187-2A>G | Chr6: 123699045 | NM_006073.4 | Candidate causal | rs578024729 | - | 0 | 0 | 0 | - |
| 9 | TRDN | c.1051+2T>C | Chr6: 123759206 | NM_006073.4 | Candidate causal | - | - | - | - | - | - |
| 10 | TRDN | c.22+1G>T | Chr6: 123957898 | NM_006073.4 | Candidate causal | rs1290195833 | - | 0 | - | - | - |
| 11 | TRDN | c.520_550+494del | Chr6: 123836792-123837316 | NM_006073.4 | Candidate causal | rs1562358749 | - | - | - | - | 244 |
| 12 | TRDN | c.232+2T>A | Chr6: 123892066 | NM_006073.4 | Candidate causal | rs1554258777 | - | 0 | - | - | 945 |
| 13 | TRDN | c.1051+1G>A | Chr6: 123759207 | NM_006073.4 | Candidate causal | rs535908547 | - | 0 | - | - | 946 |
| 14 | TRDN | c.1321+2T>C | Chr6: 123687278 | NM_006073.4 | Candidate causal | rs772232730 | - | 0 | 0 | 0 | 947 |
| 15 | TRDN | c.1321+1del | Chr6: 123687279 | NM_006073.4 | Candidate causal | rs754982716 | - | 0 | 0 | 0 | 947 |
| 16 | TRDN | c.1870+1G>A | Chr6: 123580768 | NM_006073.4 | Candidate causal | rs377115913 | - | 0 | 0 | 0 | - |
| 17 | TRDN | c.1166-1G>A | Chr6: 123702536 | NM_006073.4 | Candidate causal | rs1298986609 | - | 0 | - | 0 | 947 |
| 18 | NCF1, LOC106029312 | c.*179G>A | Chr7: 74203683 | NM_000265.6 | Uncertain Significance/ Candidate causal | rs1057519503 | - | - | - | - | 245 |
| 19 | COL1A2 | c.540+5G>A | Chr7: 94035043 | NM_000089.3 | Uncertain Significance/ Candidate causal | rs72656367 | - | - | - | - | 948 |
| 20 | BRAF | c.2128-5_2128delTGTAGA | Chr7: 140434570 | NM_004333.4 | Candidate causal | rs1131692058 | - | - | - | - | 245 |
| 21 | TSC1 | c.2503-2A>G | Chr9: 135776226 | NM_000368.4 | Candidate causal | rs118203704 | - | - | - | - | 245 |
| 22 | TSC1 | c.2041+1G>A | Chr9: 135779797 | NM_000368.4 | Candidate causal | rs397514842 | - | - | - | - | 245 |
| 23 | TSC1 | c.1998-1G>T | Chr9: 135779842 | NM_000368.4 | Candidate causal | rs118203614 | - | - | - | - | 245 |
| 24 | TSC1 | c.1997+1G>A | Chr9: 135780967 | NM_000368.4 | Candidate causal | rs118203610 | - | - | - | - | 666 |
| 25 | TSC1 | c.737+1G>T,A | Chr9: 135796749 | NM_000368.4 | Candidate causal | rs118203438 | - | - | - | - | ^666^, ^256^ |
| 26 | TSC1 | c.664-1G>C | Chr9: 135796824 | NM_000368.4 | Candidate causal | rs118203423 | - | - | - | - | 245 |
| 27 | TSC1 | c.211-1G>A | Chr9: 135801127 | NM_000368.4 | Candidate causal | rs118203353 | - | - | - | - | 245 |
| 28 | TSC1 | c.211-2A>C | Chr9: 135801128 | NM_000368.4 | Candidate causal | rs118203352 | - | - | - | - | 245 |
| 29 | TSC1 | c.-154+1502del | Chr9: 135802652 | NM_001362177.2 | Uncertain Significant/ Candidate causal | rs397514861 | - | - | - | - | 245 |
| 30 | TSC1 | c.-236_-218delinsCAA | Chr9: 135804218 | NM_001362177.2 | Candidate causal | rs1554821022 | - | - | - | - | 245 |
| 31 | KCNQ1 | c.1352-1G>C | Chr11: 2799205 | NM_181798.1 | Candidate causal | rs878854348 | - | - | - | - | 949 |
| 32 | ANO5 | c.41-1G>A | Chr11: 22225349 | NM_213599.2 | Candidate causal | rs398124625 | - | 0 | 0 | 0 | 562 |
| 33 | ANO5 | c.1407+5G>A | Chr11: 22279305 | NM_213599.2 | Uncertain Significant/ Candidate causal | rs281865464 | - | 0 | 0 | - | - |
| 34 | ANO5 | c.1898+1G>A | Chr11: 22284590 | NM_213599.2 | Candidate causal | rs142027093 | - | 0 | 0 | 0 | 562 |
| 35 | PKP2 | c.2146-1G>C | Chr12: 32955491 | NM_004572.3 | Candidate causal | rs193922674 | - | 0 | 0 | 0 | 444 |
| 36 | TSC2 | c.136_138+1del | Chr16: 2098752 | NM_000548.5 | Candidate causal | rs137854117 | - | - | - | - | 245 |
| 37 | TSC2 | c.138_138+1del | Chr16: 2098751 | NM_000548.5 | Candidate causal | rs137854117 | - | - | - | - | 950 |
| 38 | TSC2 | c.138+2T>C | Chr16: 2098756 | NM_000548.5 | Candidate causal | rs137854226 | - | - | - | - | 666 |
| 39 | TSC2 | c.139-1G>A | Chr16: 2100400 | NM_000548.5 | Candidate causal | rs1567386603 | - | - | - | - | 245 |
| 40 | TSC2 | c.226-2A>G | Chr16: 2103341 | NM_000548.5 | Candidate causal | rs45517096 | - | 0 | - | - | 245 |
| 41 | TSC2 | c.336+1G>C | Chr16: 2103454 | NM_000548.5 | Candidate causal | rs45517102 | - | - | - | - | 245 |
| 42 | TSC2 | c.336+1G>A | Chr16: 2103454 | NM_000548.5 | Candidate causal | rs45517102 | - | - | - | - | 245 |
| 43 | TSC2 | c.337-1G>A | Chr16: 2104296 | NM_000548.5 | Candidate causal | rs45517105 | - | - | - | - | 245 |
| 44 | TSC2 | c.481+1G>T | Chr16: 2104442 | NM_000548.5 | Candidate causal | rs45488500 | - | - | - | - | 666 |
| 45 | TSC2 | c.599+1G>A | Chr16: 2105521 | NM_000548.5 | Candidate causal | rs45460895 | - | - | - | - | 666 |
| 46 | TSC2 | c.600-1G>A | Chr16: 2106196 | NM_000548.5 | Candidate causal | rs45517117 | - | 0 | - | - | 245 |
| 47 | TSC2 | c.648+1G>A | Chr16: 2106246 | NM_000548.5 | Candidate causal | rs45488893 | - | - | - | - | 951 |
| 48 | TSC2 | c.774+1G>A | Chr16: 2106771 | NM_000548.5 | Candidate causal | rs45517128 | - | - | - | - | 952 |
| 49 | TSC2 | c.848+1G>A | Chr16: 2107180 | NM_000548.5 | Candidate causal | rs45466296 | - | - | - | - | 245 |
| 50 | TSC2 | c.848+281C>T | Chr16: 2107460 | NM_000548.5 | Likely Candidate causal | rs45517132 | - | 0 | - | - | 953 |
| 51 | TSC2 | c.849-1G>A | Chr16: 2108747 | NM_000548.5 | Candidate causal | rs45506396 | - | 0 | - | - | 245 |
| 52 | TSC2 | c.975+1G>T | Chr16: 2108875 | NM_000548.5 | Candidate causal | rs137854200 | - | - | - | - | 245 |
| 53 | TSC2 | c.976-15G>A | Chr16: 2110656 | NM_000548.5 | Likely Candidate causal | rs45517150 | - | - | - | - | 953 |
| 54 | TSC2 | c.976-1G>A | Chr16: 2110670 | NM_000548.5 | Candidate causal | rs45451199 | - | 0 | - | - | 245 |
| 55 | TSC2 | c.1119+1G>C | Chr16: 2110815 | NM_000548.5 | Candidate causal | rs45475793 | - | - | - | - | 245 |
| 56 | TSC2 | c.1257+1G>C | Chr16: 2112010 | NM_000548.5 | Candidate causal | rs397515066 | - | - | - | - | 245 |
| 57 | TSC2 | c.1257+1G>A | Chr16: 2112010 | NM_000548.5 | Candidate causal | rs397515066 | - | - | - | - | 245 |
| 58 | TSC2 | c.1257+2T>G | Chr16: 2112011 | NM_000548.5 | Candidate causal | rs45509697 | - | - | - | - | 666 |
| 59 | TSC2 | c.1443+1G>T | Chr16: 2113055 | NM_000548.5 | Candidate causal | rs397515257 | - | - | - | - | 245 |
| 60 | TSC2 | c.1444-2A>G | Chr16: 2114271 | NM_000548.5 | Candidate causal | rs45517174 | - | - | - | - | 245 |
| 61 | TSC2 | c.1599+1G>A | Chr16: 2114429 | NM_000548.5 | Candidate causal | rs45517182 | - | - | - | - | 245 |
| 62 | TSC2 | c.1599+2T>C | Chr16: 2114430 | NM_000548.5 | Candidate causal | rs45517183 | - | - | - | - | 851 |
| 63 | TSC2 | c.1717-2A>C | Chr16: 2120455 | NM_000548.5 | Candidate causal | rs1596335963 | - | - | - | - | 245 |
| 64 | TSC2 | c.1839+1G>T | Chr16: 2120580 | NM_000548.5 | Candidate causal | rs45438400 | - | - | - | - | 954 |
| 65 | TSC2 | c.1839+2T>C | Chr16: 2120581 | NM_000548.5 | Candidate causal | rs1555505211 | - | - | - | - | 245 |
| 66 | TSC2 | c.2098-1G>A | Chr16: 2122241 | NM_000548.5 | Candidate causal | rs45517212 | - | 0 | - | - | 245 |
| 67 | TSC2 | c.2220+1G>T | Chr16: 2122365 | NM_000548.5 | Candidate causal | rs137854361 | - | - | - | - | 245 |
| 68 | TSC2 | c.2221-2A>G | Chr16: 2122848 | NM_000548.5 | Candidate causal | rs45502196 | - | 0 | - | - | 951 |
| 69 | TSC2 | c.2355+1G>A | Chr16: 2122985 | NM_000548.5 | Candidate causal | rs45517227 | - | - | - | - | 245 |
| 70 | TSC2 | c.2355+2T>G | Chr16: 2122986 | NM_000548.5 | Candidate causal | rs45517228 | - | - | - | - | 245 |
| 71 | TSC2 | c.2355+2T>C | Chr16: 2122986 | NM_000548.5 | Candidate causal | rs45517228 | - | - | - | - | 245 |
| 72 | TSC2 | c.2355+2_2355+5del | Chr16: 2122983 | NM_000548.5 | Candidate causal | rs137854250 | - | - | - | - | 245 |
| 73 | TSC2 | c.2356-2A>C | Chr16: 2124199 | NM_000548.5 | Candidate causal | rs45517229 | - | 0 | - | - | 245 |
| 74 | TSC2 | c.2356-1G>C | Chr16: 2124200 | NM_000548.5 | Candidate causal | rs45481704 | - | 0 | - | - | 245 |
| 75 | TSC2 | c.2356-1G>A | Chr16: 2124200 | NM_000548.5 | Candidate causal | rs45481704 | - | 0 | - | - | 666 |
| 76 | TSC2 | c.2546-2A>G | Chr16: 2125798 | NM_000548.5 | Candidate causal | rs45517246 | - | 0 | - | - | 666 |
| 77 | TSC2 | c.2546-1G>A | Chr16: 2125799 | NM_000548.5 | Candidate causal | rs45468292 | - | 0 | - | - | 666 |
| 78 | TSC2 | c.2639+1G>C | Chr16: 2125894 | NM_000548.5 | Candidate causal | rs45517252 | - | - | - | - | 245 |
| 79 | TSC2 | c.2640-1G>A | Chr16: 2126068 | NM_000548.5 | Candidate causal | rs397515203 | - | - | - | - | 666 |
| 80 | TSC2 | c.2743-1G>A | Chr16: 2126491 | NM_000548.5 | Candidate causal | rs45517263 | - | 0 | - | - | 954 |
| 81 | TSC2 | c.2838-122G>A | Chr16: 2127477 | NM_000548.5 | Uncertain Significance/Candidate causal | rs1567489890 | - | - | - | - | 955 |
| 82 | TSC2 | c.3131+1G>A | Chr16: 2129198 | NM_000548.5 | Candidate causal | rs45506401 | - | - | - | - | 950 |
| 83 | TSC2 | c.3132-1G>T | Chr16: 2129276 | NM_000548.5 | Candidate causal | rs45443096 | - | 0 | - | - | 245 |
| 84 | TSC2 | c.3397+5G>A | Chr16: 2129675 | NM_000548.5 | Uncertain Significance/Candidate causal | rs45501492 | - | - | - | - | 666 |
| 85 | TSC2 | c.3610+1G>C | Chr16: 2130379 | NM_000548.5 | Candidate causal | rs45517299 | - | - | - | - | 245 |
| 86 | TSC2 | c.3641_3814+28del | Chr16: 2131626 | NM_000548.5 | Candidate causal | rs1555512330 | - | - | - | - | 245 |
| 87 | TSC2 | c.3884-497_5045del | Chr16: 2133199 | NM_000548.3 | Candidate causal | - | - | - | - | - | - |
| 88 | TSC2 | c.4005+1G>A | Chr16: 2133818 | NM_000548.5 | Candidate causal | rs45517324 | - | - | - | - | - |
| 89 | TSC2 | c.4662+1G>A | Chr16: 2135324 | NM_000548.5 | Candidate causal | rs45514095 | - | - | - | - | 666 |
| 90 | TSC2 | c.4663-1G>T | Chr16: 2136193 | NM_000548.5 | Candidate causal | rs45517358 | - | 0 | - | - | 245 |
| 91 | TSC2 | c.4989+1G>A | Chr16: 2136873 | NM_000548.5 | Candidate causal | rs45517386 | - | - | - | - | 245 |
| 92 | TSC2 | c.5068+1G>T | Chr16: 2137943 | NM_000548.5 | Candidate causal | rs45445199 | - | - | - | - | 950 |
| 93 | TSC2 | c.5068+2T>C | Chr16: 2137944 | NM_000548.5 | Candidate causal | rs397515152 | - | - | - | - | 245 |
| 94 | TSC2 | c.5069-2A>G | Chr16: 2138047 | NM_000548.5 | Candidate causal | rs45487291 | - | 0 | - | - | 245 |
| 95 | TSC2 | c.5160+1G>T | Chr16: 2138141 | NM_000548.5 | Candidate causal | rs45517399 | - | - | - | - | 666 |
| 96 | TSC2 | c.5160+1G>A | Chr16: 2138141 | NM_000548.5 | Candidate causal | rs45517399 | - | - | - | - | 956 |
| 97 | TSC2 | c.5160+2_5160+3del | Chr16: 2138140 | NM_000548.5 | Candidate causal | rs137854036 | - | - | - | - | 666 |
| 98 | TSC2 | c.5160+2T>C | Chr16: 2138142 | NM_000548.5 | Candidate causal | rs397515170 | - | - | - | - | 245 |
| 99 | TSC2 | c.5161-1G>A | Chr16: 2138227 | NM_000548.5 | Candidate causal | rs45517404 | - | - | - | - | 245 |
| 100 | TSC2 | c.5252_5259+19del | Chr16: 2138313 | NM_000548.5 | Candidate causal | rs137854397 | - | - | - | - | 245 |
| 101 | TSC2 | c.482_*102del | Chr16: 2105403 | NM_000548.3 | Likely Candidate causal | - | - | - | - | - | - |
| 102 | ACADVL | c.342+1G>C | Chr17: 7124150 | NM_000018.4 | Candidate causal | rs780020193 | - | 0 | - | 0 | - |
| 103 | ACADVL | c.753-2A>C | Chr17: 7125494 | NM_000018.4 | Candidate causal | rs398123092 | - | 0 | 0 | 0 | 565 |
| 104 | ACADVL | c.1269+1G>A | Chr17: 7127050 | NM_000018.4 | Candidate causal | rs773401248 | - | 0 | - | - | 450 |
| 105 | ACADVL | c.1605+2T>C | Chr17: 7127714 | NM_000018.4 | Candidate causal | rs1597537351 | - | - | - | - | 451 |
| 106 | TANGO2 | c.57-1743_*10769del | Chr22: 20029135-20062954 | NM_152906.5 | Candidate causal | - | - | - | - | - | - |
| 107 | TANGO2 | c.146-3605_451+2245del | Chr22: 20036383 | NM_152906.5 | Candidate causal | - | - | - | - | - | - |
| 108 | TANGO2 | c.605+1G>A | Chr22: 20049207 | NM_152906.7 | Candidate causal | rs372949028 | - | 0 | 0 | 0 | 957 |
| 109 | COX7B | c.41-2A>G | ChrX: 77158138 | NM_001866.3 | Candidate causal | rs397514584 | - | 0 | - | - | 958 |
| 110 | ABCC9 | c.2019+2T>C | Chr12: 22035698 | NM_005691.3 | Candidate causal | rs1555100687 | - | - | - | - | - |
| 111 | CACNA1C | c.1114-304G>A | Chr12: 2613704 | NM_000719.7 | Candidate causal | rs786205745 | - | - | - | - | - |
| 112 | CACNA1C | c.1114-316G>A | Chr12: 2613692 | NM_000719.7 | Candidate causal | rs587782933 | - | - | - | - | 959 |
| 113 | CACNA1C | c.1114-304G>C | Chr12: 2613704 | NM_000719.7 | Candidate causal | rs786205745 | - | - | - | - | 960 |
| 114 | CACNA1S | c.5227-2del | Chr1: 201009504 | NM_000069.3 | Candidate causal | - | - | - | - | - | 961 |
| 115 | CACNA1S | c.2854-2A>C | Chr1: 201031644 | NM_000069.3 | Candidate causal | rs1572036396 | - | - | - | - | 961 |
| 116 | CACNA1S | c.3525+1G>A | Chr1: 201028316 | NM_000069.3 | Candidate causal | rs1572033599 | - | - | - | - | 961 |
| 117 | CACNA1S | c.4798-2A>G | Chr1: 201012661 | NM_000069.3 | Candidate causal | rs1428157373 | - | 0 | - | - | 961 |
| 118 | CACNA1S | c.4113+1G>C | Chr1: 201020111 | NM_000069.3 | Candidate causal | rs1558056376 | - | - | - | - | 962 |
| 119 | CACNA1S | c.5049-2A>G | Chr1: 201010719 | NM_000069.3 | Candidate causal | rs148989517 | - | 0 | 0 | 0 | 961 |
| 120 | CACNA1S | c.3414+3A>T | Chr1: 201029783 | NM_000069.3 | Uncertain Significance | rs892742196 | - | 0 | 0 | - | 244 |
| 121 | CACNA1S | c.1948+1G>A | Chr1: 201044622 | NM_000069.3 | Uncertain Significance/Candidate causal | rs745712829 | - | 0 | - | 0 | 961 |
| 122 | CAV3|SSUH2 | c.114+2T>C | Chr3: 8775678 | NM_033337.2 | Candidate causal | rs116840787 | - | 0 | - | 0 | 963 |
| 123 | GLA|RPL36A-HNRNPH2 | c.370-2A>C | ChrX: 100656799 | NM_000169.3 | Candidate causal | - | - | - | - | - | - |
| 124 | GLA|RPL36A-HNRNPH2 | c.801+1G>C | ChrX: 100653772 | NM_000169.3 | Candidate causal | - | - | - | - | - | - |
| 125 | GLA|RPL36A-HNRNPH2 | c.300+2569_300+2583del | ChrX: 100653011-100653025 | NM_001199973.2 | Candidate causal | rs1569302697 | - | - | - | - | - |
| 126 | GLA|RPL36A-HNRNPH2 | c.802-2A>T | ChrX: 100653557 | NM_000169.2 | Candidate causal | rs797044499 | - | 0 | - | 0 | - |
| 127 | GLA|RPL36A-HNRNPH2 | c.300+3090del | ChrX: 100653534 | NM_001199973.2 | Candidate causal | rs869025435 | - | - | - | - | - |
| 128 | GLA|RPL36A-HNRNPH2 | c.301-4203del | ChrX: 100662720 | NM_001199973.2 | Candidate causal | - | - | - | - | - | - |
| 129 | GLA|RPL36A-HNRNPH2 | c.300+5242A>C | ChrX:100655687 | NM_000169.2 | Candidate causal | rs104894838 | - | 0 | - | - | - |
| 130 | GLA|RPL36A-HNRNPH2 | c.300+6256C>T | ChrX: 100656701 | NM_001199973.2 | Likely Candidate causal | rs28935195 | - | 0 | - | - | - |
| 131 | GLA|RPL36A-HNRNPH2 | c.300+6286G>A | ChrX: 100656731 | NM_001199973.2 | Candidate causal | rs104894837 | - | 0 | - | - | - |
| 132 | GLA|RPL36A-HNRNPH2 | c.300+3344dup | ChrX: 100653787-100653788 | NM_001199973.2 | Candidate causal | rs1555985091 | - | - | - | - | - |
| 133 | GLA|RPL36A-HNRNPH2 | c.300+2586_300+2587del | ChrX: 100653031-100653032 | NM_001199973.2 | Candidate causal | rs1555984840 | - | - | - | - | - |
| 134 | GLA|RPL36A-HNRNPH2 | c.301-4080_301-4076del | ChrX: 100662838-100662842 | NM_001199973.2 | Candidate causal | rs869312316 | - | - | - | - | - |
| 135 | GLA|RPL36A-HNRNPH2 | c.802-3_804delinsGGCAACTTT | ChrX: 100653553-100653558 | NM_000169.3 | Candidate causal | rs727504773 | - | - | - | - | - |
| 136 | GLA|RPL36A-HNRNPH2 | c.300+2359_300+2362del | ChrX: 100652800-100652803 | NM_001199973.2 | Candidate causal | rs869312250 | - | - | - | - | 964 |
| 137 | GLA|RPL36A-HNRNPH2 | c.300+2494_300+2496del | ChrX: 100652938-100652940 | NM_001199973.2 | Likely Candidate causal | rs1057519609 | - | - | - | - | 965 |
| 138 | GLA|RPL36A-HNRNPH2 | c.300+2467_300+2519del | ChrX: 100652911-100652963 | NM_001199973.2 | Candidate causal | rs1603037323 | - | - | - | - | 966 |
| 139 | GLA|RPL36A-HNRNPH2 | c.300+2615_300+2625del | ChrX: 100653060-100653070 | NM_001199973.2 | Candidate causal | rs1603037764 | - | - | - | - | 966 |
| 140 | GLA|RPL36A-HNRNPH2 | c.301-4167_301-4155del | ChrX: 100662755-100662767 | NM_001199973.2 | Candidate causal | rs1603047806 | - | - | - | - | 967 |
| 141 | GLA|RPL36A-HNRNPH2 | c.369+2T>G | ChrX: 100658797 | NM_000169.2 | Candidate causal | rs387906483 | - | - | - | - | 966 |
| 142 | GLA|RPL36A-HNRNPH2 | c.300+2561C>G | ChrX: 100653006 | NM_001199973.2 | Candidate causal | rs28935494 | - | - | - | - | 968 |
| 143 | GLA|RPL36A-HNRNPH2 | c.300+2933G>T | ChrX: 100653378 | NM_001199973.2 | Candidate causal | rs28935491 | - | 0 | - | - | 968 |
| 144 | GLA|RPL36A-HNRNPH2 | c.300+3022G>A | ChrX: 100653467 | NM_001199973.2 | Candidate causal | rs28935489 | - | 0 | - | - | 968 |
| 145 | GLA|RPL36A-HNRNPH2 | c.301-4198A>C | ChrX: 100662726 | NM_001199973.2 | Candidate causal | rs104894836 | - | 0 | - | - | 968 |
| 146 | GLA|RPL36A-HNRNPH2 | c.301-4163C>T | ChrX: 100662761 | NM_001199973.2 | Candidate causal | rs104894829 | - | 0 | - | - | 969 |
| 147 | GLA|RPL36A-HNRNPH2 | c.370-532_1278del | ChrX: 100652809-100657329 | NM_000169.3 | Candidate causal | - | - | - | - | - | - |
| 148 | GLA|RPL36A-HNRNPH2 | c.300+6225_300+6226delinsCC | ChrX: 100656670-100656671 | NM_001199973.2 | Candidate causal | rs1603041916 | - | - | - | - | 970 |
| 149 | GLA|RPL36A-HNRNPH2 | c.300+3064dup | ChrX: 100653508-100653509 | NM_001199973.2 | Candidate causal | rs1555985002 | - | - | - | - | - |
| 150 | GLA|RPL36A-HNRNPH2 | c.301-4097_301-4084dup | ChrX: 100662819-100662820 | NM_001199973.2 | Candidate causal | rs1555987175 | - | - | - | - | - |
| 151 | GLA|RPL36A-HNRNPH2 | c.1000-10G>A | ChrX: 100653097 | NM_000169.2 | Uncertain Significance/Candidate causal | rs869312203 | - | - | - | - | - |
| 152 | GLA|RPL36A-HNRNPH2 | c.301-4226C>G | ChrX: 100662698 | NM_001199973.2 | Candidate causal | rs104894848 | - | - | - | - | 971 |
| 153 | GLA|RPL36A-HNRNPH2 | c.999+1G>T | ChrX: 100653357 | NM_000169.2 | Candidate causal | rs1603038103 | - | - | - | - | 972 |
| 154 | GLA|RPL36A-HNRNPH2 | c.640-1del | ChrX:100653935 | NM_000169.2 | Candidate causal | rs1555985200 | - | - | - | - | - |
| 155 | GLA|RPL36A-HNRNPH2 | c.300+2357del | ChrX: 100652802 | NM_001199973.2 | Likely Candidate causal | rs1555984717 | - | - | - | - | - |
| 156 | GLA|RPL36A-HNRNPH2 | c.300+2585_300+2586del | ChrX: 100653029-100653030 | NM_001199973.2 | Candidate causal | rs886044829 | - | - | - | - | - |
| 157 | GLA|RPL36A-HNRNPH2 | c.300+2929C>G | ChrX: 100653374 | NM_001199973.2 | Candidate causal | rs28935492 | - | - | - | - | 966 |
| 158 | GLA|RPL36A-HNRNPH2 | c.300+2930C>T | ChrX: 100653375 | NM_001199973.2 | Candidate causal | rs104894832 | - | 0 | - | - | 973 |
| 159 | GLA|RPL36A-HNRNPH2 | c.300+3068_300+3070del | ChrX: 100653513-100653515 | NM_001199973.2 | Likely Candidate causal | rs1555985004 | - | - | - | - | - |
| 160 | GLA|RPL36A-HNRNPH2 | c.195-1G>T | ChrX: 100658974 | NM_000169.2 | Candidate causal | rs398123206 | - | - | - | - | 687 |
| 161 | GLA|RPL36A-HNRNPH2 | c.300+3109_300+3112del | ChrX: 100653551-100653554 | NM_001199973.2 | Candidate causal | - | - | - | - | - | - |
| 162 | GLA|RPL36A-HNRNPH2 | c.801+3A>G | ChrX: 100653770 | NM_000169.2 | Uncertain Significance/Candidate causal | rs797044748 | - | - | - | - | 970 |
| 163 | GLA|RPL36A-HNRNPH2 | c.999+2T>C | ChrX: 100653356 | NM_000169.2 | Candidate causal | rs886044860 | - | 0 | - | - | 971 |
| 164 | GLA|RPL36A-HNRNPH2 | c.369+1G>A | ChrX: 100658798 | NM_000169.2 | Candidate causal | rs797044669 | - | - | - | - | 974 |
| 165 | GLA|RPL36A-HNRNPH2 | c.300+2502_300+2517del | ChrX: 100652947-100652962 | NM_001199973.2 | Candidate causal | rs876661347 | - | - | - | - | 244 |
| 166 | GLA|RPL36A-HNRNPH2 | c.300+2939C>T | ChrX: 100653384 | NM_000169.2 | Candidate causal | rs398123227 | - | - | - | - | 975 |
| 167 | GLA|RPL36A-HNRNPH2 | c.300+2431_300+2433del | ChrX: 100652873-100652875 | NM_001199973.2 | Likely Candidate causal | rs869312241 | - | - | - | - | - |
| 168 | GLA|RPL36A-HNRNPH2 | c.640-1G>A | ChrX: 100653935 | NM_000169.2 | Candidate causal | rs398123216 | - | - | - | - | 970 |
| 169 | GLA|RPL36A-HNRNPH2 | c.301-4058del | ChrX: 100662866 | NM_001199973.2 | Candidate causal | rs1555987215 | - | - | - | - | 974 |
| 170 | GLA|RPL36A-HNRNPH2 | c.300+2608GA[2] | ChrX: 100653053-100653054 | NM_001199973.2 | Candidate causal | rs398123198 | - | - | - | - | 975 |
| 171 | GLA|RPL36A-HNRNPH2 | c.300+3411_300+3412del | ChrX: 100653855-100653856 | NM_001199973.2 | Candidate causal | rs869312389 | - | - | - | - | - |
| 172 | GLA|RPL36A-HNRNPH2 | c.640-801G>A | ChrX: 100654735 | NM_000169.3 | Uncertain Significance | rs199473684 | - | 0 | - | 0 | 976 |
| 173 | TANGO2 | c.605+1G>A | Chr22: 20049207 | NM_152906.7 | Candidate causal | rs372949028 | - | 0 | 0 | 0 | 957 |
| 174 | SCN5A | c.3228+6C>G | Chr22: 20049207 | NM_198056.2 | Uncertain Significance | rs368048551 | - | 0 | 0 | 0 | - |
| 175 | IGHMBP2 | c.1236-1G>T | Chr11: 68700766 | NM_002180.3 | Candidate causal | - | - | - | - | - | 977 |
| 176 | IGHMBP2 | c.257-2A>G | Chr11: 68675611 | NM_002180.2 | Candidate causal | rs1566424655 | - | - | - | - | 977 |
| 177 | IGHMBP2 | c.1633-2A>G | Chr11: 68702765 | NM_002180.2 | Candidate causal | rs1566445029 | - | - | - | - | 977 |
| 178 | IGHMBP2 | c.2611+1G>A | Chr11: 68704560 | NM_002180.3 | Candidate causal | rs786205090 | - | 0 | - | - | 977 |
| 179 | IGHMBP2 | c.449+1G>A | Chr11: 68675806 | NM_002180.2 | Candidate causal | rs797044802 | - | - | - | - | 977 |
| 180 | IGHMBP2 | c.2611+1G>T | Chr11: 68704560 | NM_002180.2 | Candidate causal | rs786205090 | - | 0 | - | 0 | 978 |
| 181 | IGHMBP2 | c.547+1G>A | Chr11: 68676100 | NM_002180.2 | Candidate causal | rs1057518588 | - | 0 | 0 | 0 | 979 |
| 182 | KCNH2 | c.1128+1820_1128+1821del | Chr7: 150652558-150652559 | NM_172056.2 | Likely Candidate causal | rs1064794494 | - | 0 | 0 | 0 | - |
| 183 | KCNH2 | c.2966-2_2967dup | Chr7: 150644600 - 150644601 | NM_000238.4 | Candidate causal | rs794728464 | - | - | - | - | - |
| 184 | KCNH2 | c.1128+1810C>T | Chr7: 150652569 | NM_172056.2 | Uncertain Significance | rs972201049 | - | 0 | 0 | 0 | 980 |
| 185 | KCNH2 | c.1946-4_1948del | Chr7: 150648206-150648212 | NM_000238.4 | Candidate causal | - | - | - | - | - | 244 |
| 186 | KCNH2 | c.910_916+11del | Chr7: 150655136-150655153 | NM_000238.4 | Candidate causal | rs1563169296 | - | - | - | - | 244 |
| 187 | KCNH2 | c.1129-831_3109dup | Chr7: 150644458-150644459 | NM_000238.4 | Candidate causal | - | - | - | - | - | 244 |
| 188 | KCNH2 | c.2146-3_2146-1delinsAA | Chr7: 150647509-150647511 | NM_172056.2 | Candidate causal | rs1554425320 | - | - | - | - | - |
| 189 | KCNH2 | c.307+2T>A | Chr7: 150671797 | NM_172056.2 | Candidate causal | rs796052196 | - | - | - | - | 981 |
| 190 | KCNH2 | c.76_76+1del | Chr7: 150674925-150674926 | NM_000238.4 | Candidate causal | rs1563193513 | - | - | - | - | 244 |
| 191 | KCNH2 | c.1557+2T>C | Chr7: 150649511 | NM_000238.4 | Candidate causal | rs1584855956 | - | - | - | - | 687 |
| 192 | KCNH2 | c.2145+1G>A | Chr7: 150648008 | NM_172056.2 | Candidate causal | rs886039385 | - | - | - | - | 982 |
| 193 | KCNH2 | c.308-2A>G | Chr7: 150656826 | NM_172056.2 | Candidate causal | rs1057520598 | - | - | - | - | 982 |
| 194 | KCNH2 | c.1129-1G>A | Chr7: 150649942 | NM_172056.2 | Candidate causal | rs794728478 | - | - | - | - | 983 |
| 195 | KCNH2 | c.1945+6T>C | Chr7: 150648530 | NM_172056.2 | Uncertain Significant/Candidate causal | rs794728380 | - | - | - | - | 984 |
| 196 | KCNH2 | c.1946-2A>C | Chr7: 150648210 | NM_172056.2 | Candidate causal | rs794728488 | - | - | - | - | 985 |
| 197 | KCNQ1 | c.604+1G>A | Chr11: 2591985 | NM_000218.3 | Candidate causal | rs752670256 | - | 0 | - | - | - |
| 198 | KCNQ1 | c.96+1G>A | Chr11: 2549249 | NM_181798.1 | Candidate causal | rs762814879 | - | 0 | 0 | 0 | 986 |
| 199 | KCNQ1 | c.96+5G>A | Chr11: 2549253 | NM_181798.1 | Likely Candidate causal | rs397508111 | - | 0 | 0 | 0 | - |
| 200 | KCNQ1 | c.1032+1_1129-1del | Chr11: 2604775-2608798 | NM_000218.3 | Candidate causal | - | - | - | - | - | - |
| 201 | KCNQ1 | c.302+2T>G | Chr11: 2592635 | NM_181798.1 | Candidate causal | rs796052166 | - | - | - | - | 981 |
| 202 | KCNQ1 | c.1732+1G>A | Chr11: 2798263 | NM_000218.3 | Candidate causal | - | - | - | - | - | 982 |
| 203 | KCNQ1 | c.224-1G>C | Chr11: 2592554 | NM_181798.1 | Candidate causal | rs1564820372 | - | - | - | - | 982 |
| 204 | KCNQ1 | c.303-2A>C | Chr11: 2593241 | NM_181798.1 | Candidate causal | rs1564820729 | - | - | - | - | 982 |
| 205 | KCNQ1 | c.651+1G>A | Chr11: 2604776 | NM_181798.1 | Candidate causal | rs397508070 | - | 0 | 0 | 0 | 981 |
| 206 | KCNQ1 | c.386+16231G>A | Chr11: 2482945 | NM_000218.3 | Candidate causal | - | - | - | - | - | - |
| 207 | KCNQ1 | c.1032+2T>C | Chr11: 2604777 | NM_181798.3 | Candidate causal | - | - | - | - | - | 987 |
| 208 | KCNQ1 | c.604+2T>C | Chr11: 2591986 | NM_181798.3 | Candidate causal | - | - | - | - | - | - |
| 209 | KCNQ1 | c.1251+1G>A | Chr11: 2608923 | NM_000218.3 | Candidate causal | rs1589968661 | - | - | - | - | 988 |
| 210 | KCNQ1 | c.541-2A>G | Chr11: 2604663 | NM_181798.1 | Candidate causal | rs397508133 | - | - | - | - | 983 |
| 211 | KCNQ1 | c.541-1G>C | Chr11: 2604664 | NM_181798.1 | Candidate causal | rs387906290 | - | - | - | - | 989 |
| 212 | KCNQ1 | c.1134-2_1134-1del | Chr11: 2790072-2790073 | NM_181798.1 | Candidate causal | rs1564886323 | - | - | - | - | 987 |
| 213 | KCNQ1 | c.1304+2T>C | Chr11: 2797286 | NM_181798.1 | Candidate causal | rs1590081467 | - | - | - | - | - |
| 214 | KCNQ1 | c.302+1G>A | Chr11: 2592634 | NM_181798.1 | Candidate causal | rs1589957233 | - | - | - | - | 982 |
| 215 | KCNQ1 | c.603_604+9del | Chr11: 2591979-2591989 | NM_000218.3 | Candidate causal | rs794728580 | - | - | - | - | - |
| 216 | KCNQ1 | c.1304+1G>A | Chr11: 2797285 | NM_181798.1 | Candidate causal | rs794728531 | - | - | - | - | - |
| 217 | KCNQ1 | c.1133+1G>A | Chr11: 2683312 | NM_181798.1 | Candidate causal | rs397508093 | - | - | - | - | 990 |
| 218 | KCNQ1 | c.1013-1G>T | Chr11: 2683190 | NM_181798.1 | Candidate causal | rs775537394 | - | 0 | 0 | 0 | 991 |
| 219 | LMNA | c.1609-3C>G | Chr1: 156107442 | NM_170707.4 | Likely Candidate causal | rs267607581 | - | - | - | - | 532 |
| 220 | RNF207 | c.1109+1G>A | Chr1: 6271179 | NM_207396.3 | Candidate causal | rs552508881 | - | 0 | 0 | 0 | - |
| 221 | SCN5A | c.3954_3960+1dup | Chr3: 38603904-38603905 | NM_000335.5 | Candidate causal | - | - | - | - | - | - |
| 222 | SCN5A | c.612-189C>T | Chr3: 38655514 | NM_000335.5 | Candidate causal | rs577421914 | - | 0 | 0 | 0 | - |
| 223 | SCN5A | c.393-1C>T | Chr3: 38663981 | NM_000335.5 | Candidate causal | rs759235726 | - | 0 | 0 | - | 992 |
| 224 | SCN5A | c.612-229T>G | Chr3: 38655554 | NM_198056.2 | Candidate causal | rs765669597 | - | 0 | - | - | - |
| 225 | SCN5A | c.1338+2T>A | Chr3: 38647440 | NM_198056.2 | Candidate causal | rs786204839 | - | 0 | - | 0 | 993 |
| 226 | SCN5A | c.3512-1G>C | Chr3: 38616943 | NM_198056.2 | Candidate causal | rs1553698563 | - | - | - | - | - |
| 227 | SCN5A | c.3840+1G>A | Chr3: 38607899 | NM_198056.2 | Candidate causal | rs1366120635 | - | 0 | 0 | 0 | 994 |
| 228 | SCN5A | c.4245+1G>C | Chr3: 38601637 | NM_198056.2 | Candidate causal | rs794728879 | - | - | - | - | 985 |
| 229 | SCN5A | c.4437+5G>A | Chr3: 385979273 | NM_198056.2 | Uncertain Significance | rs1057520531 | - | - | - | - | 618 |
| 230 | CASQ2 | c.940-1G>T | Chr1: 116245617 | NM_001232.4 | Candidate causal | rs876657635 | - | - | - | - | - |
| 231 | CASQ2 | c.737+2T>A | Chr1: 116269611 | NM_001232.4 | Candidate causal | rs1366771173 | - | - | - | - | - |
| 232 | CASQ2 | c.235-2A>G | Chr1: 116287535 | NM_001232.3 | Candidate causal | rs1060502164 | - | - | - | - | 995 |

**References**

241. Lind S, Olsson A, Eriksson M, Rudling M, Eggertsen G, Angelin B. Autosomal recessive hypercholesterolaemia: normalization of plasma LDL cholesterol by ezetimibe in combination with statin treatment. *Journal of internal medicine*. 2004;256(5):406-412.

242. Canizales-Quinteros S, Aguilar-Salinas CA, Huertas-Vázquez A, et al. A novel ARH splice site mutation in a Mexican kindred with autosomal recessive hypercholesterolemia. *Human genetics*. 2005;116(1-2):114-120.

243. Höcker B, Knüppel T, Waldherr R, Schaefer F, Weber S, Tönshoff B. Recurrence of proteinuria 10 years post-transplant in NPHS2-associated focal segmental glomerulosclerosis after conversion from cyclosporin A to sirolimus. *Pediatric Nephrology*. 2006;21(10):1476-1479.

244. Nykamp K, Anderson M, Powers M, et al. Sherloc: a comprehensive refinement of the ACMG–AMP variant classification criteria. *Genetics in Medicine*. 2017/10/01 2017;19(10):1105-1117. <https://doi.org10.1038/gim.2017.37>.

245. Directors ABo. ACMG policy statement: updated recommendations regarding analysis and reporting of secondary findings in clinical genome-scale sequencing. *Genetics in medicine: official journal of the American College of Medical Genetics*. 2015;17(1):68.

246. Campagnoli M, Rossi A, Palmqvist L, et al. A novel splicing mutation causes an undescribed type of analbuminemia. *Biochimica et Biophysica Acta (BBA)-Molecular Basis of Disease*. 2002;1586(1):43-49.

247. Wang F, Zhang Y, Mao J, et al. Spectrum of mutations in Chinese children with steroid-resistant nephrotic syndrome. *Pediatric nephrology*. 2017;32(7):1181-1192.

248. Guaragna MS, Lutaif ACG, Piveta CS, et al. Two distinct WT1 mutations identified in patients and relatives with isolated nephrotic proteinuria. *Biochemical and biophysical research communications*. 2013;441(2):371-376.

249. Walsh R, Thomson KL, Ware JS, et al. Reassessment of Mendelian gene pathogenicity using 7,855 cardiomyopathy cases and 60,706 reference samples. *Genetics in Medicine*. 2017;19(2):192-203.

250. Sperandeo MP, Andria G, Sebastio G. Lysinuric protein intolerance: update and extended mutation analysis of the SLC7A7 gene. *Human mutation*. 2008;29(1):14-21.

251. Yao C, Chen G, Song C, et al. Genome‐wide mapping of plasma protein QTLs identifies putatively causal genes and pathways for cardiovascular disease. *Nature communications*. 2018;9(1):1-11.

252. Medeiros AM, Alves AC, Bourbon M. Mutational analysis of a cohort with clinical diagnosis of familial hypercholesterolemia: considerations for genetic diagnosis improvement. *Genetics in Medicine*. 2016/04/01 2016;18(4):316-324. <https://doi.org10.1038/gim.2015.71>.

253. Shin DG, Han SM, Kim DI, et al. Clinical features of familial hypercholesterolemia in Korea: predictors of pathogenic mutations and coronary artery disease–a study supported by the Korean Society of Lipidology and Atherosclerosis. *Atherosclerosis*. 2015;243(1):53-58.

254. Rodríguez‐Jiménez C, Pernía O, Mostaza J, et al. Functional analysis of new variants at the low‐density lipoprotein receptor associated with familial hypercholesterolemia. *Human mutation*. 2019;40(8):1181-1190.

255. Jiang L, Wu W-F, Sun L-Y, et al. The use of targeted exome sequencing in genetic diagnosis of young patients with severe hypercholesterolemia. *Scientific reports*. 2016;6:36823.

256. Xiong HY, Alipanahi B, Lee LJ, et al. The human splicing code reveals new insights into the genetic determinants of disease. *Science*. 2015;347(6218):1254806.

257. Sharifi M, Walus-Miarka M, Idzior-Waluś B, et al. The genetic spectrum of familial hypercholesterolemia in south-eastern Poland. *Metabolism*. 2016;65(3):48-53.

258. Marduel M, Carrié A, Sassolas A, et al. Molecular spectrum of autosomal dominant hypercholesterolemia in France. *Human mutation*. 2010;31(11):E1811-E1824. <https://doi.org10.1002/humu.21348>.

259. Fan L-l, Lin M-j, Chen Y-q, et al. Novel mutations of low-density lipoprotein receptor gene in China patients with familial hypercholesterolemia. *Applied biochemistry and biotechnology*. 2015;176(1):101-109.

260. Cladaras C, Hadzopoulou-Cladaras M, Felber B, Pavlakis G, Zannis V. The molecular basis of a familial apoE deficiency. An acceptor splice site mutation in the third intron of the deficient apoE gene. *Journal of Biological Chemistry*. 1987;262(5):2310-2315.

261. Hendrickx J, Coucke P, Dams E, et al. Mutations in the phosphorylase kinase gene PHKA2 are responsible for X-linked liver glycogen storage disease. *Human molecular genetics*. 1995;4(1):77-83.

262. Takano K, Goto K, Motobayashi M, et al. Early manifestations of epileptic encephalopathy, brain atrophy, and elevation of serum neuron specific enolase in a boy with beta-propeller protein-associated neurodegeneration. *European journal of medical genetics*. 2017;60(10):521-526.

263. Pelusi S, Baselli G, Pietrelli A, et al. Rare Pathogenic Variants Predispose to Hepatocellular Carcinoma in Nonalcoholic Fatty Liver Disease. *Sci Rep*. 2019;9(1):3682-3682. <https://doi.org10.1038/s41598-019-39998-2>.

264. Zhong S, Magnolo AL, Sundaram M, et al. Nonsynonymous mutations within APOB in human familial hypobetalipoproteinemia: evidence for feedback inhibition of lipogenesis and postendoplasmic reticulum degradation of apolipoprotein B. *J Biol Chem*. 2010;285(9):6453-6464. <https://doi.org10.1074/jbc.M109.060467>.

265. Mykytyn K, Nishimura DY, Searby CC, et al. Evaluation of complex inheritance involving the most common Bardet-Biedl syndrome locus (BBS1). *Am J Hum Genet*. 2003;72(2):429-437. <https://doi.org10.1086/346172>.

266. Mykytyn K, Nishimura DY, Searby CC, et al. Identification of the gene (BBS1) most commonly involved in Bardet-Biedl syndrome, a complex human obesity syndrome. *Nature Genetics*. 2002/08/01 2002;31(4):435-438. <https://doi.org10.1038/ng935>.

267. Beales PL, Badano JL, Ross AJ, et al. Genetic interaction of BBS1 mutations with alleles at other BBS loci can result in non-Mendelian Bardet-Biedl syndrome. *Am J Hum Genet*. 2003;72(5):1187-1199. <https://doi.org10.1086/375178>.

268. Jespersgaard C, Fang M, Bertelsen M, et al. Molecular genetic analysis using targeted NGS analysis of 677 individuals with retinal dystrophy. *Sci Rep*. 2019/02/04 2019;9(1):1219. <https://doi.org10.1038/s41598-018-38007-2>.

269. Chen J, Smaoui N, Hammer MBH, et al. Molecular Analysis of Bardet-Biedl Syndrome Families: Report of 21 Novel Mutations in 10 Genes. *Investigative Ophthalmology & Visual Science*. 2011;52(8):5317-5324. <https://doi.org10.1167/iovs.11-7554>.

270. Fauser S, Munz M, Besch D. Further support for digenic inheritance in Bardet-Biedl syndrome. *Journal of Medical Genetics*. 2003;40(8):e104-e104. <https://doi.org10.1136/jmg.40.8.e104>.

271. Mary L, Chennen K, Stoetzel C, et al. Bardet-Biedl syndrome: Antenatal presentation of forty-five fetuses with biallelic pathogenic variants in known Bardet-Biedl syndrome genes. *Clinical Genetics*. 2019;95(3):384-397. <https://doi.orghttps://doi.org/10.1111/cge.13500>.

272. Yates CL, Monaghan KG, Copenheaver D, et al. Whole-exome sequencing on deceased fetuses with ultrasound anomalies: expanding our knowledge of genetic disease during fetal development. *Genetics in Medicine*. 2017/10/01 2017;19(10):1171-1178. <https://doi.org10.1038/gim.2017.31>.

273. Katsanis N, Eichers ER, Ansley SJ, et al. <em>BBS4</em> Is a Minor Contributor to Bardet-Biedl Syndrome and May Also Participate in Triallelic Inheritance. *The American Journal of Human Genetics*. 2002;71(1):22-29. <https://doi.org10.1086/341031>.

274. Ellingford JM, Barton S, Bhaskar S, et al. Molecular findings from 537 individuals with inherited retinal disease. *Journal of Medical Genetics*. 2016;53(11):761-767. <https://doi.org10.1136/jmedgenet-2016-103837>.

275. Feuillan PP, Ng D, Han JC, et al. Patients with Bardet-Biedl Syndrome Have Hyperleptinemia Suggestive of Leptin Resistance. *The Journal of Clinical Endocrinology & Metabolism*. 2011;96(3):E528-E535. <https://doi.org10.1210/jc.2010-2290>.

276. Lindstrand A, Frangakis S, Carvalho Claudia MB, et al. Copy-Number Variation Contributes to the Mutational Load of Bardet-Biedl Syndrome. *The American Journal of Human Genetics*. 2016;99(2):318-336. <https://doi.org10.1016/j.ajhg.2015.04.023>.

277. Fattahi Z, Rostami P, Najmabadi A, et al. Mutation profile of BBS genes in Iranian patients with Bardet–Biedl syndrome: genetic characterization and report of nine novel mutations in five BBS genes. *Journal of Human Genetics*. 2014/07/01 2014;59(7):368-375. <https://doi.org10.1038/jhg.2014.28>.

278. Kleefstra T, van Zelst-Stams WA, Nillesen WM, et al. Further clinical and molecular delineation of the 9q subtelomeric deletion syndrome supports a major contribution of <em>EHMT1</em> haploinsufficiency to the core phenotype. *Journal of Medical Genetics*. 2009;46(9):598-606. <https://doi.org10.1136/jmg.2008.062950>.

279. Quélin C, Loget P, Boutaud L, et al. Loss of function IFT27 variants associated with an unclassified lethal fetal ciliopathy with renal agenesis. *American Journal of Medical Genetics Part A*. 2018;176(7):1610-1613. <https://doi.orghttps://doi.org/10.1002/ajmg.a.38685>.

280. Hsiung Y-C, Lin P-C, Chen C-S, et al. Identification of a novel <em>LDLR</em> disease-causing variant using capture-based next-generation sequencing screening of familial hypercholesterolemia patients in Taiwan. *Atherosclerosis*. 2018;277:440-447. <https://doi.org10.1016/j.atherosclerosis.2018.08.022>.

281. Meshkov A, Ershova A, Kiseleva A, et al. The LDLR, APOB, and PCSK9 Variants of Index Patients with Familial Hypercholesterolemia in Russia. *Genes (Basel)*. 2021;12(1):66. <https://doi.org10.3390/genes12010066>.

282. Humphries SE, Cranston T, Allen M, et al. Mutational analysis in UK patients with a clinical diagnosis of familial hypercholesterolaemia: relationship with plasma lipid traits, heart disease risk and utility in relative tracing. *Journal of Molecular Medicine*. 2006/03/01 2006;84(3):203-214. <https://doi.org10.1007/s00109-005-0019-z>.

283. Yu W, Nohara A, Higashikata T, Lu H, Inazu A, Mabuchi H. Molecular genetic analysis of familial hypercholesterolemia: spectrum and regional difference of LDL receptor gene mutations in Japanese population. *Atherosclerosis*. 2002;165(2):335-342. <https://doi.org10.1016/S0021-9150(02)00249-6>.

284. Lombardi P, Sijbrands EJ, van de Giessen K, et al. Mutations in the low density lipoprotein receptor gene of familial hypercholesterolemic patients detected by denaturing gradient gel electrophoresis and direct sequencing. *Journal of Lipid Research*. 1995/04/01/ 1995;36(4):860-867. <https://doi.orghttps://doi.org/10.1016/S0022-2275(20)40068-9>.

285. Graham CA, McIlhatton BP, Kirk CW, et al. Genetic screening protocol for familial hypercholesterolemia which includes splicing defects gives an improved mutation detection rate. *Atherosclerosis*. 2005;182(2):331-340. <https://doi.org10.1016/j.atherosclerosis.2005.02.016>.

286. Kim JH, Choi HK, Lee H, et al. Novel and recurrent mutations of the LDL receptor gene in Korean patients with familial hypercholesterolemia. *Molecules and cells*. Aug 31 2004;18(1):63-70.

287. Chater R, Aït Chihab K, Rabès JP, et al. Mutational heterogeneity in low-density lipoprotein receptor gene related to familial hypercholesterolemia in Morocco. *Clinica Chimica Acta*. 2006/11/01/ 2006;373(1):62-69. <https://doi.orghttps://doi.org/10.1016/j.cca.2006.05.007>.

288. Mak Y-T, Pang C-P, Tomlinson B, et al. Mutations in the Low-Density Lipoprotein Receptor Gene in Chinese Familial Hypercholesterolemia Patients. *Arteriosclerosis, Thrombosis, and Vascular Biology*. 1998;18(10):1600-1605. <https://doi.orgdoi:10.1161/01.ATV.18.10.1600>.

289. Fard-Esfahani P, Khatami S, Zeinali C, Taghikhani M, Allahyari M. A modified conformation sensitive gel electrophoresis (CSGE) method for rapid and accurate detection of low density lipoprotein (LDL) receptor gene mutations in Familial Hypercholesterolemia. *Clinical Biochemistry*. 2005/06/01/ 2005;38(6):579-583. <https://doi.orghttps://doi.org/10.1016/j.clinbiochem.2005.02.002>.

290. Sharifi M, Walus-Miarka M, Idzior-Waluś B, et al. The genetic spectrum of familial hypercholesterolemia in south-eastern Poland. *Metabolism - Clinical and Experimental*. 2016;65(3):48-53. <https://doi.org10.1016/j.metabol.2015.10.018>.

291. Maruyama T, Miyake Y, Yamamura T, et al. A novel point mutation in a splice acceptor site of intron 1 of the human low density lipoprotein receptor gene which causes severe hypercholesterolemia: An unexpected absence of exon skipping. *Human Mutation*. 1998;11(6):480-481. <https://doi.orghttps://doi.org/10.1002/(SICI)1098-1004(1998)11:6><480::AID-HUMU11>3.0.CO;2-W.

292. Vaca G, Vàzquez A, Magaña MT, et al. Mutational analysis of the LDL receptor and <em>APOB</em> genes in Mexican individuals with autosomal dominant hypercholesterolemia. *Atherosclerosis*. 2011;218(2):391-396. <https://doi.org10.1016/j.atherosclerosis.2011.06.006>.

293. Leren TP, Manshaus T, Skovholt U, et al. Application of Molecular Genetics for Diagnosing Familial Hypercholesterolemia in Norway: Results from a Family-Based Screening Program. *Semin Vasc Med*. //

22.03.2004 2004;4(01):75-85.

294. Wang J, Dron JS, Ban MR, et al. Polygenic Versus Monogenic Causes of Hypercholesterolemia Ascertained Clinically. *Arteriosclerosis, Thrombosis, and Vascular Biology*. 2016;36(12):2439-2445. <https://doi.orgdoi:10.1161/ATVBAHA.116.308027>.

295. Bertolini S, Pisciotta L, Rabacchi C, et al. Spectrum of mutations and phenotypic expression in patients with autosomal dominant hypercholesterolemia identified in Italy. *Atherosclerosis*. 2013;227(2):342-348. <https://doi.org10.1016/j.atherosclerosis.2013.01.007>.

296. Wintjens R, Bozon D, Belabbas K, et al. Global molecular analysis and APOE mutations in a cohort of autosomal dominant hypercholesterolemia patients in France. *Journal of lipid research*. 2016;57(3):482-491. <https://doi.org10.1194/jlr.P055699>.

297. Amsellem S, Briffaut D, Carrié A, et al. Intronic mutations outside of Alu-repeat-rich domains of the LDL receptor gene are a cause of familial hypercholesterolemia. *Human Genetics*. 2002/12/01 2002;111(6):501-510. <https://doi.org10.1007/s00439-002-0813-4>.

298. Weiss N, Binder G, Keller C. Mutations in the low-density-lipoprotein receptor gene in German patients with familial hypercholesterolaemia. *Journal of Inherited Metabolic Disease*. 2000;23(8):778-790. <https://doi.orghttps://doi.org/10.1023/A:1026704517598>.

299. Yang K-C, Su Y-N, Shew J-Y, et al. LDLR and ApoB are Major Genetic Causes of Autosomal Dominant Hypercholesterolemia in a Taiwanese Population. *Journal of the Formosan Medical Association*. 2007/10/01/ 2007;106(10):799-807. <https://doi.orghttps://doi.org/10.1016/S0929-6646(08)60044-3>.

300. Yu L, Heere-Ress E, Boucher B, et al. Familial hypercholesterolemia. Acceptor splice site (G&#x2192;C) mutation in intron 7 of the LDL-R gene: alternate RNA editing causes exon 8 skipping or a premature stop codon in exon 8. LDL-R<sub>Honduras-1</sub> [LDL-R<sub>1061(&#x2212;1) G&#x2192;C</sub>]. *Atherosclerosis*. 1999;146(1):125-131. <https://doi.org10.1016/S0021-9150(99)00109-4>.

301. ArulJothi KN, Whitthall RA, Futema M, et al. Molecular analysis of the LDLR gene in coronary artery disease patients from the Indian population. *Clinical Biochemistry*. 2016/06/01/ 2016;49(9):669-674. <https://doi.orghttps://doi.org/10.1016/j.clinbiochem.2016.02.009>.

302. Mozas P, Castillo S, Tejedor D, et al. Molecular characterization of familial hypercholesterolemia in Spain: Identification of 39 novel and 77 recurrent mutations in LDLR. *Human Mutation*. 2004;24(2):187-187. <https://doi.orghttps://doi.org/10.1002/humu.9264>.

303. Dušková L, Kopečková L, Jansová E, et al. An APEX-based genotyping microarray for the screening of 168 mutations associated with familial hypercholesterolemia. *Atherosclerosis*. 2011;216(1):139-145. <https://doi.org10.1016/j.atherosclerosis.2011.01.023>.

304. Lombardi MP, Redeker EJ, Defesche JC, et al. Molecular genetic testing for familial hypercholesterolemia: spectrum of LDL receptor gene mutations in the Netherlands. *Clinical Genetics*. 2000;57(2):116-124. <https://doi.orghttps://doi.org/10.1034/j.1399-0004.2000.570205.x>.

305. Nauck MS, Köster W, Dörfer K, et al. Identification of recurrent and novel mutations in the LDL receptor gene in German patients with familial hypercholesterolemia. *Human Mutation*. 2001;18(2):165-166. <https://doi.orghttps://doi.org/10.1002/humu.1171>.

306. Do R, Stitziel NO, Won H-H, et al. Exome sequencing identifies rare LDLR and APOA5 alleles conferring risk for myocardial infarction. *Nature*. 2015/02/01 2015;518(7537):102-106. <https://doi.org10.1038/nature13917>.

307. Reshef A, Nissen H, Triger L, et al. Molecular genetics of familial hypercholesterolemia in Israel. *Human Genetics*. 1996/09/01 1996;98(5):581-586. <https://doi.org10.1007/s004390050263>.

308. Heath KE, Humphries SE, Middleton-Price H, Boxer M. A molecular genetic service for diagnosing individuals with familial hypercholesterolaemia (FH) in the United Kingdom. *European Journal of Human Genetics*. 2001/04/01 2001;9(4):244-252. <https://doi.org10.1038/sj.ejhg.5200633>.

309. Mollaki V, Progias P, Drogari E. Novel LDLR Variants in Patients with Familial Hypercholesterolemia: In Silico Analysis as a Tool to Predict Pathogenic Variants in Children and Their Families. *Annals of Human Genetics*. 2013;77(5):426-434. <https://doi.orghttps://doi.org/10.1111/ahg.12032>.

310. Sun X-M, Patel DD, Knight BL, Soutar AK. Comparison of the Genetic Defect with LDL-Receptor Activity in Cultured Cells from Patients With a Clinical Diagnosis of Heterozygous Familial Hypercholesterolemia. *Arteriosclerosis, Thrombosis, and Vascular Biology*. 1997;17(11):3092-3101. <https://doi.orgdoi:10.1161/01.ATV.17.11.3092>.

311. Widhalm K, Dirisamer A, Lindemayr A, Kostner G. Diagnosis of families with familial hypercholesterolaemia and/or Apo B-100 defect by means of DNA analysis of LDL-receptor gene mutations. *Journal of Inherited Metabolic Disease*. 2007;30(2):239-247. <https://doi.orghttps://doi.org/10.1007/s10545-007-0563-5>.

312. Lind S, Rystedt E, Eriksson M, Wiklund O, Angelin B, Eggertsen G. Genetic characterization of Swedish patients with familial hypercholesterolemia: a heterogeneous pattern of mutations in the LDL receptor gene. *Atherosclerosis*. 2002;163(2):399-407. <https://doi.org10.1016/S0021-9150(02)00038-2>.

313. Usifo E, Leigh SEA, Whittall RA, et al. Low-Density Lipoprotein Receptor Gene Familial Hypercholesterolemia Variant Database: Update and Pathological Assessment. *Annals of Human Genetics*. 2012;76(5):387-401. <https://doi.orghttps://doi.org/10.1111/j.1469-1809.2012.00724.x>.

314. Bernier L, Boulet L, Roy M, Dufour R, Larivière F, Davignon J. Two new large deletions in the low density lipoprotein receptor (<em>LDLR</em>) gene not revealed by PCR-based molecular diagnosis of familial hypercholesterolemia. *Atherosclerosis*. 2008;197(1):118-124. <https://doi.org10.1016/j.atherosclerosis.2007.02.026>.

315. Wang J, Ban MR, Hegele RA. Multiplex ligation-dependent probe amplification of LDLR enhances molecular diagnosis of familial hypercholesterolemia. *Journal of Lipid Research*. 2005/02/01/ 2005;46(2):366-372. <https://doi.orghttps://doi.org/10.1194/jlr.D400030-JLR200>.

316. Jelassi A, Slimani A, Rabès JP, et al. Genomic characterization of two deletions in the LDLR gene in Tunisian patients with familial hypercholesterolemia. *Clinica Chimica Acta*. 2012/12/24/ 2012;414:146-151. <https://doi.orghttps://doi.org/10.1016/j.cca.2012.08.002>.

317. Medeiros AM, Alves AC, Aguiar P, Bourbon M, Pediatric Investigators of the Portuguese Familial Hypercholesterolemia S. Cardiovascular risk assessment of dyslipidemic children: analysis of biomarkers to identify monogenic dyslipidemia. *Journal of lipid research*. 2014;55(5):947-955. <https://doi.org10.1194/jlr.P043182>.

318. Chiou K-R, Charng M-J, Chang H-M. Array-based resequencing for mutations causing familial hypercholesterolemia. *Atherosclerosis*. 2011;216(2):383-389. <https://doi.org10.1016/j.atherosclerosis.2011.02.006>.

319. Chmara M, Wasąg B, Żuk M, et al. Molecular characterization of Polish patients with familial hypercholesterolemia: novel and recurrentLDLR mutations. *Journal of Applied Genetics*. 2010/03/01 2010;51(1):95-106. <https://doi.org10.1007/BF03195716>.

320. Ekström U, Abrahamson M, Sveger T, Lombardi P, Nilsson-Ehle P. An efficient screening procedure detecting six novel mutations in the LDL receptor gene in Swedish children with hypercholesterolemia. *Human Genetics*. 1995/08/01 1995;96(2):147-150. <https://doi.org10.1007/BF00207370>.

321. Tichý L, Freiberger T, Zapletalová P, Soška V, Ravčuková B, Fajkusová L. The molecular basis of familial hypercholesterolemia in the Czech Republic: Spectrum of <em>LDLR</em> mutations and genotype&#x2013;phenotype correlations. *Atherosclerosis*. 2012;223(2):401-408. <https://doi.org10.1016/j.atherosclerosis.2012.05.014>.

322. Benito-Vicente A, Alves AC, Etxebarria A, Medeiros AM, Martin C, Bourbon M. The importance of an integrated analysis of clinical, molecular, and functional data for the genetic diagnosis of familial hypercholesterolemia. *Genetics in Medicine*. 2015/12/01 2015;17(12):980-988. <https://doi.org10.1038/gim.2015.14>.

323. Hattori H, Hirayama T, Nobe Y, et al. Eight novel mutations and functional impairments of the LDL receptor in familial hypercholesterolemia in the north of Japan. *Journal of Human Genetics*. 2002/02/01 2002;47(2):80-87. <https://doi.org10.1007/s100380200005>.

324. Bourbon M, Duarte M, Alves A, Medeiros A, Marques L, Soutar A. Genetic diagnosis of familial hypercholesterolaemia: The importance of functional analysis of potential splice-site mutations. *Journal of medical genetics*. 06/01 2009;46:352-7. <https://doi.org10.1136/jmg.2007.057000>.

325. Medeiros AM, Alves AC, Francisco V, Bourbon M. Update of the Portuguese Familial Hypercholesterolaemia Study. *Atherosclerosis*. 2010;212(2):553-558. <https://doi.org10.1016/j.atherosclerosis.2010.07.012>.

326. Bertolini S, Cassanelli S, Garuti R, et al. Analysis of LDL Receptor Gene Mutations in Italian Patients With Homozygous Familial Hypercholesterolemia. *Arteriosclerosis, Thrombosis, and Vascular Biology*. 1999;19(2):408-418. <https://doi.orgdoi:10.1161/01.ATV.19.2.408>.

327. Taylor A, Tabrah S, Wang D, et al. Multiplex ARMS analysis to detect 13 common mutations in familial hypercholesterolaemia. *Clinical Genetics*. 2007;71(6):561-568. <https://doi.orghttps://doi.org/10.1111/j.1399-0004.2007.00807.x>.

328. Ebhardt M, Schmidt H, Doerk T, et al. Mutation analysis in 46 German families with familial hypercholesterolemia: Identification of 8 new mutations. *Human Mutation*. 1999;13(3):257-257. <https://doi.orghttps://doi.org/10.1002/(SICI)1098-1004(1999)13:3><257::AID-HUMU14>3.0.CO;2-D.

329. Kulseth MA, Berge KE, Bogsrud MP, Leren TP. Analysis of LDLR mRNA in patients with familial hypercholesterolemia revealed a novel mutation in intron 14, which activates a cryptic splice site. *Journal of Human Genetics*. 2010/10/01 2010;55(10):676-680. <https://doi.org10.1038/jhg.2010.87>.

330. Alonso R, Defesche JC, Tejedor D, et al. Genetic diagnosis of familial hypercholesterolemia using a DNA-array based platform. *Clinical Biochemistry*. 2009/06/01/ 2009;42(9):899-903. <https://doi.orghttps://doi.org/10.1016/j.clinbiochem.2009.01.017>.

331. Jelassi A, Najah M, Jguirim I, et al. A novel splice site mutation of the LDL receptor gene in a Tunisian hypercholesterolemic family. *Clinica Chimica Acta*. 2008/06/01/ 2008;392(1):25-29. <https://doi.orghttps://doi.org/10.1016/j.cca.2008.02.019>.

332. deCampo A, Schallmoser K, Schmidt H, Toplak H, Kostner GM. A novel splice-site mutation in intron 7 causes more severe hypercholesterolemia than a combined FH-FDB defect. *Atherosclerosis*. 2001;157(2):524-525. <https://doi.org10.1016/S0021-9150(01)00535-4>.

333. Lehrman MA, Russell DW, Goldstein JL, Brown MS. Exon-Alu recombination deletes 5 kilobases from the low density lipoprotein receptor gene, producing a null phenotype in familial hypercholesterolemia. *Proceedings of the National Academy of Sciences*. 1986;83(11):3679-3683. <https://doi.org10.1073/pnas.83.11.3679>.

334. Wang J, Huff E, Janecka L, Hegele RA. Low density lipoprotein receptor (LDLR) gene mutations in Canadian subjects with familial hypercholesterolemia, but not of French descent. *Human Mutation*. 2001;18(4):359-359. <https://doi.orghttps://doi.org/10.1002/humu.1205>.

335. Fouchier SW, Kastelein JJP, Defesche JC. Update of the molecular basis of familial hypercholesterolemia in The Netherlands. *Human Mutation*. 2005;26(6):550-556. <https://doi.orghttps://doi.org/10.1002/humu.20256>.

336. Cameron J, Holla ØL, Kulseth MA, Leren TP, Berge KE. Splice-site mutation c.313+1, G>A in intron 3 of the LDL receptor gene results in transcripts with skipping of exon 3 and inclusion of intron 3. *Clinica Chimica Acta*. 2009/05/01/ 2009;403(1):131-135. <https://doi.orghttps://doi.org/10.1016/j.cca.2009.02.001>.

337. Mozas P, Cenarro A, Civeira F, Castillo S, Ros E, Pocovi M. Mutation analysis in 36 unrelated Spanish subjects with familial hypercholesterolemia: Identification of 3 novel mutations in the LDL receptor gene. *Human Mutation*. 2000;15(5):483-484. <https://doi.orghttps://doi.org/10.1002/(SICI)1098-1004(200005)15:5><483::AID-HUMU19>3.0.CO;2-Q.

338. Fouchier SW, Defesche JC, Umans-Eckenhausen MA, Kastelein JJ. The molecular basis of familial hypercholesterolemia in The Netherlands. *Human Genetics*. 2001/12/01 2001;109(6):602-615. <https://doi.org10.1007/s00439-001-0628-8>.

339. Punzalan FER, Sy RG, Santos RS, et al. Low Density Lipoprotein &minus; Receptor (LDL-R) Gene Mutations among Filipinos with Familial Hypercholesterolemia. *Journal of Atherosclerosis and Thrombosis*. 2005;12(5):276-283. <https://doi.org10.5551/jat.12.276>.

340. Igartua C, Mozaffari S, Nicolae D, Ober C. Rare non-coding variants are associated with plasma lipid traits in a founder population. *Sci Rep*. 11/27 2017;7. <https://doi.org10.1038/s41598-017-16550-8>.

341. Reeskamp LF, Hartgers ML, Peter J, et al. A Deep Intronic Variant in <i>LDLR</i> in Familial Hypercholesterolemia. *Circulation: Genomic and Precision Medicine*. 2018;11(12):e002385. <https://doi.orgdoi:10.1161/CIRCGEN.118.002385>.

342. Graaf Avd, Avis HJ, Kusters DM, et al. Molecular Basis of Autosomal Dominant Hypercholesterolemia. *Circulation*. 2011;123(11):1167-1173. <https://doi.orgdoi:10.1161/CIRCULATIONAHA.110.979450>.

343. Khoo K, Van Acker P, Defesche J, et al. Low-density lipoprotein receptor gene mutations in a Southeast Asian population with familial hypercholesterolemia. *Clinical Genetics*. 2000;58(2):98-105. <https://doi.orghttps://doi.org/10.1034/j.1399-0004.2000.580202.x>.

344. Taylor A, Wang D, Patel K, et al. Mutation detection rate and spectrum in familial hypercholesterolaemia patients in the UK pilot cascade project. *Clinical Genetics*. 2010;77(6):572-580. <https://doi.orghttps://doi.org/10.1111/j.1399-0004.2009.01356.x>.

345. Peeters AV, Kotze MJ, Scholtz CL, et al. A 3-basepair deletion in repeat 1 of the LDL receptor promoter reduces transcriptional activity in a South African Pedi. *J Lipid Res*. May 1998;39(5):1021-4.

346. De Castro-Orós I, Pampín S, Bolado-Carrancio A, et al. Functional analysis of LDLR promoter and 5′ UTR mutations in subjects with clinical diagnosis of familial hypercholesterolemia. *Human Mutation*. 2011;32(8):868-872. <https://doi.orghttps://doi.org/10.1002/humu.21520>.

347. Smith AJP, Ahmed F, Nair D, et al. A functional mutation in the LDLR promoter (−139C>G) in a patient with familial hypercholesterolemia. *European Journal of Human Genetics*. 2007/11/01 2007;15(11):1186-1189. <https://doi.org10.1038/sj.ejhg.5201897>.

348. Dedoussis G, Pitsavos C, Kelberman D, et al. FH-Pyrgos: a novel mutation in the promoter (−45delT) of the low-density lipoprotein receptor gene associated with familial hypercholesterolemia. *Clinical Genetics*. 2003;64(5):414-419. <https://doi.orghttps://doi.org/10.1034/j.1399-0004.2003.00164.x>.

349. Webb JC, Patel DD, Shoulders CC, Knight BL, Soutar AK. Genetic Variation at a Splicing Branch Point in Intron 9 of the Low Density Lipoprotein (LDL)-Receptor Gene: A Rare Mutation that Disrupts mRNA Splicing in a Patient with Familial Hypercholesterolaemia and a Common Polymorphism. *Human Molecular Genetics*. 1996;5(9):1325-1331. <https://doi.org10.1093/hmg/5.9.1325>.

350. Pirillo A, Garlaschelli K, Arca M, et al. Spectrum of mutations in Italian patients with familial hypercholesterolemia: New results from the LIPIGEN study. *Atherosclerosis Supplements*. 2017/10/01/ 2017;29:17-24. <https://doi.orghttps://doi.org/10.1016/j.atherosclerosissup.2017.07.002>.

351. Sun X-M, Neuwirth C, Wade DP, Knight BL, Soutar AK. A mutation (T-45C) in the promoter region of the low-density-lipoprotein (LDL)-receptor gene is associated with a mild clinical phenotype in a patient with heterozygous familial hypercholesterolaemia (FH). *Human Molecular Genetics*. 1995;4(11):2125-2129. <https://doi.org10.1093/hmg/4.11.2125>.

352. Chiang AP, Beck JS, Yen H-J, et al. Homozygosity mapping with SNP arrays identifies <em>TRIM32</em>, an E3 ubiquitin ligase, as a Bardet–Biedl syndrome gene (<em>BBS11</em>). *Proceedings of the National Academy of Sciences*. 2006;103(16):6287-6292. <https://doi.org10.1073/pnas.0600158103>.

353. Song J, Smaoui N, Ayyagari R, et al. High-Throughput Retina-Array for Screening 93 Genes Involved in Inherited Retinal Dystrophy. *Investigative Ophthalmology & Visual Science*. 2011;52(12):9053-9060. <https://doi.org10.1167/iovs.11-7978>.

354. Ansley SJ, Badano JL, Blacque OE, et al. Basal body dysfunction is a likely cause of pleiotropic Bardet–Biedl syndrome. *Nature*. 2003/10/01 2003;425(6958):628-633. <https://doi.org10.1038/nature02030>.

355. Elliott AM, Simard LR, Coghlan G, et al. A novel mutation in <em>KIAA0196</em>: identification of a gene involved in Ritscher–Schinzel/3C syndrome in a First Nations cohort. *Journal of Medical Genetics*. 2013;50(12):819-822. <https://doi.org10.1136/jmedgenet-2013-101715>.

356. Sanchez-Navarro I, R. J. da Silva L, Blanco-Kelly F, et al. Combining targeted panel-based resequencing and copy-number variation analysis for the diagnosis of inherited syndromic retinopathies and associated ciliopathies. *Scientific Reports*. 2018/03/27 2018;8(1):5285. <https://doi.org10.1038/s41598-018-23520-1>.

357. Lin W-X, Zeng H-S, Zhang Z-H, et al. Molecular diagnosis of pediatric patients with citrin deficiency in China: SLC25A13 mutation spectrum and the geographic distribution. *Scientific reports*. 2016;6(1):1-12.

358. Lu YB, Kobayashi K, Ushikai M, et al. Frequency and distribution in East Asia of 12 mutations identified in the SLC25A13 gene of Japanese patients with citrin deficiency. *Journal of human genetics*. 2005;50(7):338-346.

359. Gandotra S, Le Dour C, Bottomley W, et al. Perilipin deficiency and autosomal dominant partial lipodystrophy. *New England Journal of Medicine*. 2011;364(8):740-748.

360. Okubo M, Ucar SK, Podskarbi T, Murase T, Shin YS, Coker M. Molecular and clinical delineation of 12 patients with glycogen storage disease type III in Western Turkey. *Clinica Chimica Acta*. 2015;439:162-167.

361. Kishnani PS, Austin SL, Arn P, et al. Glycogen storage disease type III diagnosis and management guidelines. *Genetics in Medicine*. 2010;12(7):446-463.

362. Lu C, Qiu Z, Sun M, Wang W, Wei M, Zhang X. Spectrum of AGL mutations in Chinese patients with glycogen storage disease type III: identification of 31 novel mutations. *Journal of Human Genetics*. 2016/07/01 2016;61(7):641-645. <https://doi.org10.1038/jhg.2016.24>.

363. Michon C-C, Gargiulo M, Hahn-Barma V, et al. Cognitive profile of patients with glycogen storage disease type III: a clinical description of seven cases. *Journal of Inherited Metabolic Disease*. 2015/05/01 2015;38(3):573-580. <https://doi.org10.1007/s10545-014-9789-1>.

364. Kishnani PS, Austin SL, Arn P, et al. Glycogen Storage Disease Type III diagnosis and management guidelines. *Genetics in Medicine*. 2010/07/01 2010;12(7):446-463. <https://doi.org10.1097/GIM.0b013e3181e655b6>.

365. Cheng A, Zhang M, Okubo M, Omichi K, Saltiel A. Distinct mutations in the glycogen debranching enzyme found in glycogen storage disease type III lead to impairment in diverse cellular functions. *Human molecular genetics*. 04/01 2009;18:2045-52. <https://doi.org10.1093/hmg/ddp128>.

366. Vořechovský I. Aberrant 3′ splice sites in human disease genes: mutation pattern, nucleotide structure and comparison of computational tools that predict their utilization. *Nucleic Acids Research*. 2006;34(16):4630-4641. <https://doi.org10.1093/nar/gkl535>.

367. Aoyama Y, Ozer I, Demirkol M, et al. Molecular features of 23 patients with glycogen storage disease type III in Turkey: a novel mutation p.R1147G associated with isolated glucosidase deficiency, along with 9 AGL mutations. *Journal of Human Genetics*. 2009/11/01 2009;54(11):681-686. <https://doi.org10.1038/jhg.2009.100>.

368. Goldstein JL, Austin SL, Boyette K, et al. Molecular analysis of the AGL gene: Identification of 25 novel mutations and evidence of genetic heterogeneity in patients with Glycogen Storage Disease Type III. *Genetics in Medicine*. 2010/07/01 2010;12(7):424-430. <https://doi.org10.1097/GIM.0b013e3181d94eaa>.

369. Okubo M, Horinishi A, Suzuki Y, Murase T, Hayasaka K. Compound heterozygous patient with glycogen storage disease type III: identification of two novel AGL mutations, a donor splice site mutation of Chinese origin and a 1-bp deletion of Japanese origin. *Am J Med Genet*. 2000/07// 2000;93(3):211-214. <https://doi.org10.1002/1096-8628(20000731)93:3&lt;211::aid-ajmg10&gt;3.0.co;2-z>.

370. Okubo M, Aoyama Y, Murase T. A novel donor splice site mutation in the glycogen debranching enzyme gene is associated with glycogen storage disease type III. *Biochem Biophys Res Commun*. 1996/07// 1996;224(2):493-499. <https://doi.org10.1006/bbrc.1996.1055>.

371. Lucchiari S, Donati MA, Parini R, et al. Molecular Characterisation of GSD III Subjects and Identification of Six Novel Mutations in AGL. *Human mutation*. 12/01 2002;20:480. <https://doi.org10.1002/humu.9093>.

372. Zhang Y, Xu M, Chen X, et al. Genetic analysis and clinical assessment of four patients with Glycogen Storage Disease Type IIIa in China. *BMC Medical Genetics*. 2018/04/04 2018;19(1):54. <https://doi.org10.1186/s12881-018-0560-6>.

373. Okubo M, Horinishi A, Nakamura N, et al. A novel point mutation in an acceptor splice site of intron 32 (IVS32 A-12-->G) but no exon 3 mutations in the glycogen debranching enzyme gene in a homozygous patient with glycogen storage disease type IIIb. *Human genetics*. 02/01 1998;102:1-5. <https://doi.org10.1007/s004390050646>.

374. Hisama FM, Lessel D, Leistritz D, et al. Coronary artery disease in a Werner syndrome‐like form of progeria characterized by low levels of progerin, a splice variant of lamin A. *American journal of medical genetics Part A*. 2011;155(12):3002-3006.

375. Becker JR, Deo RC, Werdich AA, Panàkovà D, Coy S, MacRae CA. Human cardiomyopathy mutations induce myocyte hyperplasia and activate hypertrophic pathways during cardiogenesis in zebrafish. *Disease models & mechanisms*. 2011;4(3):400-410.

376. Watkins H, McKenna WJ, Thierfelder L, et al. Mutations in the Genes for Cardiac Troponin T and α-Tropomyosin in Hypertrophic Cardiomyopathy. *New England Journal of Medicine*. 1995;332(16):1058-1065. <https://doi.org10.1056/nejm199504203321603>.

377. Buratti E, Chivers M, Královicová J, et al. Aberrant 5′ splice sites in human disease genes: Mutation pattern, nucleotide structure and comparison of computational tools that predict their utilization. *Nucleic acids research*. 02/01 2007;35:4250-63. <https://doi.org10.1093/nar/gkm402>.

378. Ceyhan-Birsoy O, Agrawal P, Hidalgo C, et al. Recessive truncating titin gene, TTN, mutations presenting as centronuclear myopathy. *Neurology*. 08/23 2013;81. <https://doi.org10.1212/WNL.0b013e3182a6ca62>.

379. Roberts AM, Ware JS, Herman DS, et al. Integrated allelic, transcriptional, and phenomic dissection of the cardiac effects of titin truncations in health and disease. *Science Translational Medicine*. 2015;7(270):270ra6-270ra6. <https://doi.org10.1126/scitranslmed.3010134>.

380. Ware JS, Li J, Mazaika E, et al. Shared Genetic Predisposition in Peripartum and Dilated Cardiomyopathies. *New England Journal of Medicine*. 2016;374(3):233-241. <https://doi.org10.1056/NEJMoa1505517>.

381. Quinzii CM, López LC, Gilkerson RW, et al. Reactive oxygen species, oxidative stress, and cell death correlate with level of CoQ10 deficiency. *The FASEB Journal*. 2010;24(10):3733-3743.

382. Mignot C, Apartis E, Durr A, et al. Phenotypic variability in ARCA2 and identification of a core ataxic phenotype with slow progression. *Orphanet Journal of Rare Diseases*. 2013/10/28 2013;8(1):173. <https://doi.org10.1186/1750-1172-8-173>.

383. Pronicka E, Piekutowska-Abramczuk D, Ciara E, et al. New perspective in diagnostics of mitochondrial disorders: Two years' experience with whole-exome sequencing at a national paediatric centre. *Journal of Translational Medicine*. 06/12 2016;14. <https://doi.org10.1186/s12967-016-0930-9>.

384. Ortiz-Genga M, Cuenca S, dal ferro M, et al. Truncating FLNC Mutations Are Associated With High-Risk Dilated and Arrhythmogenic Cardiomyopathies. *Journal of the American College of Cardiology*. 12/01 2016;68:2440-2451. <https://doi.org10.1016/j.jacc.2016.09.927>.

385. Posey J, Rosenfeld J, James R, et al. Molecular Diagnostic Experience of Whole-Exome Sequencing in Adult Patients. *Genetics in medicine : official journal of the American College of Medical Genetics*. 12/03 2015;18. <https://doi.org10.1038/gim.2015.142>.

386. Brea Calvo G, Haack T, Karall D, et al. COQ4 Mutations Cause a Broad Spectrum of Mitochondrial Disorders Associated with CoQ10 Deficiency. *Am J Hum Genet*. 02/05 2015;96:309-317. <https://doi.org10.1016/j.ajhg.2014.12.023>.

387. Cortes VA, Smalley SV, Goldenberg D, Lagos CF, Hodgson MI, Santos JL. Divergent metabolic phenotype between two sisters with congenital generalized lipodystrophy due to double AGPAT2 homozygous mutations. A clinical, genetic and in silico study. *PLoS One*. 2014;9(1).

388. Kassem HS, Azer RS, Ayad MS, et al. Early Results of Sarcomeric Gene Screening from the Egyptian National BA-HCM Program. *Journal of Cardiovascular Translational Research*. 2013/02/01 2013;6(1):65-80. <https://doi.org10.1007/s12265-012-9425-0>.

389. Burns C, Bagnall RD, Lam L, Semsarian C, Ingles J. Multiple gene variants in hypertrophic cardiomyopathy in the era of next-generation sequencing. *Circulation: Cardiovascular Genetics*. 2017;10(4):e001666.

390. Miller EM, Hinton RB, Czosek R, et al. Genetic testing in pediatric left ventricular noncompaction. *Circulation: Cardiovascular Genetics*. 2017;10(6):e001735.

391. Ito K, Patel PN, Gorham JM, et al. Identification of pathogenic gene mutations in LMNA and MYBPC3 that alter RNA splicing. *Proceedings of the National Academy of Sciences*. 2017;114(29):7689-7694.

392. Viswanathan SK, Sanders HK, McNamara JW, et al. Hypertrophic cardiomyopathy clinical phenotype is independent of gene mutation and mutation dosage. *PLoS One*. 2017;12(11).

393. Weissler-Snir A, Hindieh W, Gruner C, et al. Lack of phenotypic differences by cardiovascular magnetic resonance imaging in MYH7 (β-myosin heavy chain)-versus MYBPC3 (myosin-binding protein C)-related hypertrophic cardiomyopathy. *Circulation: Cardiovascular Imaging*. 2017;10(2):e005311.

394. Ackerman MJ, Priori SG, Willems S, et al. HRS/EHRA expert consensus statement on the state of genetic testing for the channelopathies and cardiomyopathies: this document was developed as a partnership between the Heart Rhythm Society (HRS) and the European Heart Rhythm Association (EHRA). *Europace*. 2011;13(8):1077-1109.

395. Janin A, Chanavat V, Rollat‐Farnier PA, et al. Whole MYBPC3 NGS sequencing as a molecular strategy to improve the efficiency of molecular diagnosis of patients with hypertrophic cardiomyopathy. *Human Mutation*. 2020;41(2):465-475.

396. Ingles J, Burns C, Bagnall R, et al. NON-FAMILIAL HYPERTROPHIC CARDIOMYOPATHY: PREVALENCE, NATURAL HISTORY AND CLINICAL IMPLICATIONS. *Journal of the American College of Cardiology*. 03/01 2017;69:839. <https://doi.org10.1016/S0735-1097(17)34228-6>.

397. Cecconi M, Parodi MI, Formisano F, et al. Targeted next-generation sequencing helps to decipher the genetic and phenotypic heterogeneity of hypertrophic cardiomyopathy. *International journal of molecular medicine*. 2016;38(4):1111-1124.

398. Zou Y, Wang J, Liu X, et al. Multiple gene mutations, not the type of mutation, are the modifier of left ventricle hypertrophy in patients with hypertrophic cardiomyopathy. *Molecular biology reports*. 2013;40(6):3969-3976.

399. Alfares AA, Kelly MA, McDermott G, et al. Results of clinical genetic testing of 2,912 probands with hypertrophic cardiomyopathy: expanded panels offer limited additional sensitivity. *Genetics in Medicine*. 2015/11/01 2015;17(11):880-888. <https://doi.org10.1038/gim.2014.205>.

400. Murphy SL, Anderson JH, Kapplinger JD, et al. Evaluation of the Mayo Clinic phenotype-based genotype predictor score in patients with clinically diagnosed hypertrophic cardiomyopathy. *Journal of cardiovascular translational research*. 2016;9(2):153-161.

401. Kühnisch J, Herbst C, Al‐Wakeel‐Marquard N, et al. Targeted panel sequencing in pediatric primary cardiomyopathy supports a critical role of TNNI3. *Clinical Genetics*. 2019;96(6):549-559.

402. Frisso G, Detta N, Coppola P, et al. Functional Studies and In Silico Analyses to Evaluate Non-Coding Variants in Inherited Cardiomyopathies. *Int J Mol Sci*. 2016;17(11):1883. <https://doi.org10.3390/ijms17111883>.

403. Marston S, Copeland ON, Jacques A, et al. Evidence From Human Myectomy Samples That <i>MYBPC3</i> Mutations Cause Hypertrophic Cardiomyopathy Through Haploinsufficiency*. *Circulation Research*. 2009;105(3):219-222. <https://doi.orgdoi:10.1161/CIRCRESAHA.109.202440>.

404. Flavigny J, Robert P, Camelin J-C, Schwartz K, Carrier L, Berrebi-Bertrand I. Biomolecular interactions between human recombinant β-MyHC and cMyBP-Cs implicated in familial hypertrophic cardiomyopathy. *Cardiovascular research*. 2003;60(2):388-396.

405. Lu C, Wu W, Liu F, et al. Molecular analysis of inherited cardiomyopathy using next generation semiconductor sequencing technologies. *Journal of translational medicine*. 2018;16(1):241.

406. Adalsteinsdottir B, Teekakirikul P, Maron BJ, et al. Nationwide study on hypertrophic cardiomyopathy in Iceland: evidence of a MYBPC3 founder mutation. *Circulation*. 2014;130(14):1158-1167.

407. Calore C, De Bortoli M, Romualdi C, et al. A founder MYBPC3 mutation results in HCM with a high risk of sudden death after the fourth decade of life. *Journal of medical genetics*. 2015;52(5):338-347.

408. Sedaghat-Hamedani F, Haas J, Zhu F, et al. Clinical genetics and outcome of left ventricular non-compaction cardiomyopathy. *European heart journal*. 2017;38(46):3449-3460.

409. Ross SB, Bagnall RD, Ingles J, Van Tintelen JP, Semsarian C. Burden of recurrent and ancestral mutations in families with hypertrophic cardiomyopathy. *Circulation: Cardiovascular Genetics*. 2017;10(3):e001671.

410. Bagnall RD, Ingles J, Dinger ME, et al. Whole Genome Sequencing Improves Outcomes of Genetic Testing in Patients With Hypertrophic Cardiomyopathy. *Journal of the American College of Cardiology*. 2018/07/24/ 2018;72(4):419-429. <https://doi.orghttps://doi.org/10.1016/j.jacc.2018.04.078>.

411. Waldmüller S, Müller M, Rackebrandt K, et al. Array-Based Resequencing Assay for Mutations Causing Hypertrophic Cardiomyopathy. *Clinical Chemistry*. 2008;54(4):682-687. <https://doi.org10.1373/clinchem.2007.099119>.

412. Walsh R, Thomson KL, Ware JS, et al. Reassessment of Mendelian gene pathogenicity using 7,855 cardiomyopathy cases and 60,706 reference samples. *Genetics in Medicine*. 2017/02/01 2017;19(2):192-203. <https://doi.org10.1038/gim.2016.90>.

413. Brito D, Miltenberger-Miltenyi G, Vale Pereira S, Silva D, Diogo AN, Madeira H. Sarcomeric hypertrophic cardiomyopathy: Genetic profile in a Portuguese population [10.1016/j.repce.2011.12.021]. *Revista Portuguesa de Cardiologia (English edition)*. 2012;31(9):577-587. <https://doi.org10.1016/j.repce.2011.12.021>.

414. Christiaans I, Mook ORF, Alders M, Bikker H, Lekanne dit Deprez RH. Large next-generation sequencing gene panels in genetic heart disease: challenges in clinical practice. *Netherlands Heart Journal*. 2019/06/01 2019;27(6):299-303. <https://doi.org10.1007/s12471-019-1251-4>.

415. uacute, ntilde, ez L, et al. Somatic MYH7, MYBPC3, TPM1, TNNT2 and TNNI3 mutations in sporadic hypertrophic cardiomyopathy. *Circulation Journal*. 2013;77(9):2358-2365. <https://doi.org10.1253/circj.CJ-13-0294>.

416. Shamsi A, Hertecant J, Souid A-K, Al Jasmi F. Whole exome sequencing diagnosis of inborn errors of metabolism and other disorders in United Arab Emirates. *Orphanet journal of rare diseases*. 07/08 2016;11:94. <https://doi.org10.1186/s13023-016-0474-3>.

417. Wang J, Wang Y, Zou Y, et al. Malignant effects of multiple rare variants in sarcomere genes on the prognosis of patients with hypertrophic cardiomyopathy. *European Journal of Heart Failure*. 09/01 2014;16. <https://doi.org10.1002/ejhf.144>.

418. Lopes L, Syrris P, Guttmann O, et al. Novel genotype–phenotype associations demonstrated by high-throughput sequencing in patients with hypertrophic cardiomyopathy. *Heart (British Cardiac Society)*. 10/01 2014. <https://doi.org10.1136/heartjnl-2014-306387>.

419. Waldmüller S, Erdmann J, Binner P, et al. Novel correlations between the genotype and the phenotype of hypertrophic and dilated cardiomyopathy: results from the German Competence Network Heart Failure. *European Journal of Heart Failure*. 2011;13(11):1185-1192. <https://doi.orghttps://doi.org/10.1093/eurjhf/hfr074>.

420. Mademont-Soler I, Mates J, Yotti R, et al. Additional value of screening for minor genes and copy number variants in hypertrophic cardiomyopathy. *PLoS One*. 2017;12(8):e0181465-e0181465. <https://doi.org10.1371/journal.pone.0181465>.

421. Havndrup O. Outcome of clinical versus genetic family screening in hypertrophic cardiomyopathy with focus on cardiac β-myosin gene mutations. *Cardiovascular Research - CARDIOVASC RES*. 02/01 2003;57:347-357. <https://doi.org10.1016/S0008-6363(02)00711-3>.

422. Ko C, Arscott P, Concannon M, et al. Genetic testing impacts the utility of prospective familial screening in hypertrophic cardiomyopathy through identification of a nonfamilial subgroup. *Genetics in Medicine*. 2018/01/01 2018;20(1):69-75. <https://doi.org10.1038/gim.2017.79>.

423. Mak C, Chen S, Mok NS, et al. Genetic basis of channelopathies and cardiomyopathies in Hong Kong Chinese patients: A 10-year regional laboratory experience. *Hong Kong Medical Journal*. 03/02 2018;24. <https://doi.org10.12809/hkmj176870>.

424. Fourey D, Care M, Siminovitch K, et al. Prevalence and Clinical Implication of Double Mutations in Hypertrophic Cardiomyopathy: Revisiting the Gene-Dose Effect. *Circulation: Cardiovascular Genetics*. 2017;10:e001685.

425. Richard P, Charron P, Carrier L, et al. Hypertrophic Cardiomyopathy. *Circulation*. 2003;107(17):2227-2232. <https://doi.orgdoi:10.1161/01.CIR.0000066323.15244.54>.

426. Lin J, Zheng D-D, Tao Q, et al. Two novel mutations of the MYBPC3 gene identified in Chinese families with hypertrophic cardiomyopathy. *Canadian Journal of Cardiology*. 2010/12/01/ 2010;26(10):518-522. <https://doi.orghttps://doi.org/10.1016/S0828-282X(10)70464-5>.

427. Carrier L, Bonne G, Bahrend E, et al. Organization and Sequence of Human Cardiac Myosin Binding Protein C Gene (MYBPC3) and Identification of Mutations Predicted to Produce Truncated Proteins in Familial Hypertrophic Cardiomyopathy. *Circulation Research*. 1997;80(3):427-434. <https://doi.orgdoi:10.1161/01.res.0000435859.24609.b3>.

428. Funada A, Konno T, Fujino N, et al. Impact of Renin-Angiotensin System Polymorphisms on Development of Systolic Dysfunction in Hypertrophic Cardiomyopathy

&ndash; Evidence From a Study of Genotyped Patients &ndash. *Circulation Journal*. 2010;74(12):2674-2680. <https://doi.org10.1253/circj.CJ-10-0482>.

429. Ho CY, Day SM, Ashley EA, et al. Genotype and Lifetime Burden of Disease in Hypertrophic Cardiomyopathy. *Circulation*. 2018;138(14):1387-1398. <https://doi.orgdoi:10.1161/CIRCULATIONAHA.117.033200>.

430. Ingles J, Doolan A, Chiu C, Seidman J, Seidman C, Semsarian C. Compound and double mutations in patients with hypertrophic cardiomyopathy: implications for genetic testing and counselling. *Journal of Medical Genetics*. 2005;42(10):e59-e59. <https://doi.org10.1136/jmg.2005.033886>.

431. Zeller R, Ivandic BT, Ehlermann P, et al. Large-scale mutation screening in patients with dilated or hypertrophic cardiomyopathy: a pilot study using DGGE. *Journal of Molecular Medicine*. 2006/08/01 2006;84(8):682-691. <https://doi.org10.1007/s00109-006-0056-2>.

432. Erdmann J, Raible J, Maki-Abadi J, et al. Spectrum of clinical phenotypes and gene variants in cardiac myosin-binding protein C mutation carriers with hypertrophic cardiomyopathy. *Journal of the American College of Cardiology*. 09/01 2001;38:322-30. <https://doi.org10.1016/S0735-1097(01)01387-0>.

433. Jääskeläinen P, Kuusisto J, Miettinen R, et al. Mutations in the cardiac myosin-binding protein C gene are the predominant cause of familial hypertrophic cardiomyopathy in eastern Finland. *Journal of Molecular Medicine*. 2002/07/01 2002;80(7):412-422. <https://doi.org10.1007/s00109-002-0323-9>.

434. Xin B, Puffenberger E, Tumbush J, Bockoven JR, Wang H. Homozygosity for a novel splice site mutation in the cardiac myosin-binding protein C gene causes severe neonatal hypertrophic cardiomyopathy. *American Journal of Medical Genetics Part A*. 2007;143A(22):2662-2667. <https://doi.orghttps://doi.org/10.1002/ajmg.a.31981>.

435. Klaassen S, Probst S, Oechslin E, et al. Mutations in Sarcomere Protein Genes in Left Ventricular Noncompaction. *Circulation*. 2008;117(22):2893-2901. <https://doi.orgdoi:10.1161/CIRCULATIONAHA.107.746164>.

436. Broendberg AK, Christiansen MK, Nielsen JC, Pedersen LN, Jensen HK. Targeted next generation sequencing in a young population with suspected inherited malignant cardiac arrhythmias. *European Journal of Human Genetics*. 2018/03/01 2018;26(3):303-313. <https://doi.org10.1038/s41431-017-0060-8>.

437. Liu Y, Li D, Ding Y, et al. Further delineation of AGPAT2 and BSCL2 related congenital generalized lipodystrophy in young infants. *European journal of medical genetics*. 2019;62(9):103542.

438. Martinelli S, Stellacci E, Pannone L, et al. Molecular diversity and associated phenotypic spectrum of germline CBL mutations. *Human mutation*. 2015;36(8):787-796.

439. Seaby EG, Gilbert RD, Andreoletti G, et al. Unexpected findings in a child with atypical hemolytic uremic syndrome: An example of how genomics is changing the clinical diagnostic paradigm. *Frontiers in pediatrics*. 2017;5:113.

440. Loh ML, Sakai DS, Flotho C, et al. Mutations in CBL occur frequently in juvenile myelomonocytic leukemia. *Blood*. 2009;114(9):1859-1863. <https://doi.org10.1182/blood-2009-01-198416>.

441. Kerr B, Allanson J, Delrue MA, et al. The diagnosis of Costello syndrome: nomenclature in Ras/MAPK pathway disorders. *American Journal of Medical Genetics Part A*. 2008;146(9):1218-1220.

442. members ATF, Elliott PM, Anastasakis A, et al. 2014 ESC Guidelines on diagnosis and management of hypertrophic cardiomyopathy: the Task Force for the Diagnosis and Management of Hypertrophic Cardiomyopathy of the European Society of Cardiology (ESC). *European heart journal*. 2014;35(39):2733-2779.

443. Kim J, Cho SY, Yang A, et al. An atypical case of Noonan syndrome with KRAS mutation diagnosed by targeted exome sequencing. *Annals of pediatric endocrinology & metabolism*. 2017;22(3):203.

444. Assis FR, Krishnan A, Zhou X, et al. Cardiac sympathectomy for refractory ventricular tachycardia in arrhythmogenic right ventricular cardiomyopathy. *Heart rhythm*. 2019;16(7):1003-1010. <https://doi.org10.1016/j.hrthm.2019.01.019>.

445. Calvo SE, Compton AG, Hershman SG, et al. Molecular diagnosis of infantile mitochondrial disease with targeted next-generation sequencing. *Science translational medicine*. 2012;4(118):118ra10-118ra10.

446. Mayr JA, Zimmermann FA, Horváth R, et al. Deficiency of the mitochondrial phosphate carrier presenting as myopathy and cardiomyopathy in a family with three affected children. *Neuromuscular Disorders*. 2011/11/01/ 2011;21(11):803-808. <https://doi.orghttps://doi.org/10.1016/j.nmd.2011.06.005>.

447. Ghezzi D, Baruffini E, Haack T, et al. Mutations of the Mitochondrial-tRNA Modifier MTO1 Cause Hypertrophic Cardiomyopathy and Lactic Acidosis. *Am J Hum Genet*. 05/16 2012;90:1079-87. <https://doi.org10.1016/j.ajhg.2012.04.011>.

448. Morscher RJ, Ducker GS, Li SH-J, et al. Mitochondrial translation requires folate-dependent tRNA methylation. *Nature*. 2018;554(7690):128-132. <https://doi.org10.1038/nature25460>.

449. Miller MJ, Burrage LC, Gibson JB, et al. Recurrent ACADVL molecular findings in individuals with a positive newborn screen for very long chain acyl-coA dehydrogenase (VLCAD) deficiency in the United States. *Molecular genetics and metabolism*. 2015;116(3):139-145.

450. Diekman EF, Ferdinandusse S, Van Der Pol L, et al. Fatty acid oxidation flux predicts the clinical severity of VLCAD deficiency. *Genetics in Medicine*. 2015;17(12):989-994.

451. Cox KB, Hamm DA, Millington DS, et al. Gestational, pathologic and biochemical differences between very long-chain acyl-CoA dehydrogenase deficiency and long-chain acyl-CoA dehydrogenase deficiency in the mouse. *Human Molecular Genetics*. 2001;10(19):2069-2077. <https://doi.org10.1093/hmg/10.19.2069>.

452. Shchelochkov O, Wong L-J, Shaibani A, Shinawi M. Atypical presentation of VLCAD deficiency associated with a novel ACADVL splicing mutation. *Muscle & Nerve*. 2009;39(3):374-382. <https://doi.orghttps://doi.org/10.1002/mus.21157>.

453. Andresen BS, Olpin S, Poorthuis BJHM, et al. Clear Correlation of Genotype with Disease Phenotype in Very–Long-Chain Acyl-CoA Dehydrogenase Deficiency. *The American Journal of Human Genetics*. 1999/02/01/ 1999;64(2):479-494. <https://doi.orghttps://doi.org/10.1086/302261>.

454. Miller MJ, Burrage LC, Gibson JB, et al. Recurrent ACADVL molecular findings in individuals with a positive newborn screen for very long chain acyl-coA dehydrogenase (VLCAD) deficiency in the United States. *Molecular Genetics and Metabolism*. 2015/11/01/ 2015;116(3):139-145. <https://doi.orghttps://doi.org/10.1016/j.ymgme.2015.08.011>.

455. Andresen B, Bross P, Vianey-Saban C, et al. Cloning and characterization of human very-long-chain acyl-CoA dehydrogenase cDNA, chromosomal assignment of the gene and identification in four patients of nine different mutations within the VLCAD gene. *Human molecular genetics*. 05/01 1996;5:461-72.

456. Ko JM, Seo J, Choi M, Song J, Lee K-A, Shin CH. Rare Korean Cases of Very-long-chain Acyl-CoA Dehydrogenase Deficiency with a Novel Recurrent Mutation. *Ann Clin Lab Sci*. 2016 2016;46(1):97-101.

457. Siu W-K, Mak CM, Siu SL-Y, et al. Molecular diagnosis for a fatal case of very long-chain acyl-CoA dehydrogenase deficiency in Hong Kong Chinese with a novel mutation: a preventable death by newborn screening. *Diagn Mol Pathol*. 2012/09// 2012;21(3):184-187. <https://doi.org10.1097/pdm.0b013e31825554d0>.

458. Pena LDM, van Calcar SC, Hansen J, et al. Outcomes and genotype-phenotype correlations in 52 individuals with VLCAD deficiency diagnosed by NBS and enrolled in the IBEM-IS database. *Molecular Genetics and Metabolism*. 2016/08/01/ 2016;118(4):272-281. <https://doi.orghttps://doi.org/10.1016/j.ymgme.2016.05.007>.

459. Coughlin CR, Ficicioglu C. Genotype–phenotype correlations: sudden death in an infant with very-long-chain acyl-CoA dehydrogenase deficiency. *Journal of Inherited Metabolic Disease*. 2010/12/01 2010;33(3):129-131. <https://doi.org10.1007/s10545-009-9041-6>.

460. Hoffmann L, Haussmann U, Mueller M, Spiekerkoetter U. VLCAD enzyme activity determinations in newborns identified by screening: a valuable tool for risk assessment. *Journal of Inherited Metabolic Disease*. 2012;35(2):269-277. <https://doi.orghttps://doi.org/10.1007/s10545-011-9391-8>.

461. Scalais E, Bottu J, Wanders RJA, Ferdinandusse S, Waterham HR, De Meirleir L. Familial very long chain acyl-CoA dehydrogenase deficiency as a cause of neonatal sudden infant death: Improved survival by prompt diagnosis. *American Journal of Medical Genetics Part A*. 2015;167(1):211-214. <https://doi.orghttps://doi.org/10.1002/ajmg.a.36803>.

462. Nino Martinez M, Mateus E, Fonseca D, et al. Identification and Functional Characterization of GAA Mutations in Colombian Patients Affected by Pompe Disease. *JIMD reports*. 02/22 2013;7:39-48. <https://doi.org10.1007/8904_2012_138>.

463. Kroos M, Pomponio RJ, van Vliet L, et al. Update of the Pompe disease mutation database with 107 sequence variants and a format for severity rating. *Human Mutation*. 2008;29(6):E13-E26. <https://doi.orghttps://doi.org/10.1002/humu.20745>.

464. Bali DS, Goldstein JL, Banugaria S, et al. Predicting cross-reactive immunological material (CRIM) status in Pompe disease using GAA mutations: Lessons learned from 10 years of clinical laboratory testing experience. *American Journal of Medical Genetics Part C: Seminars in Medical Genetics*. 2012;160C(1):40-49. <https://doi.orghttps://doi.org/10.1002/ajmg.c.31319>.

465. Ng B, Mathews KD, Bartlett HL. A 16-Week-Old Infant With Failure to Thrive and Hypotonia. *Clinical Pediatrics*. 2013;52(11):1075-1078. <https://doi.org10.1177/0009922813502125>.

466. Parini R, De Lorenzo P, Dardis A, et al. Long term clinical history of an Italian cohort of infantile onset Pompe disease treated with enzyme replacement therapy. *Orphanet Journal of Rare Diseases*. 2018/02/08 2018;13(1):32. <https://doi.org10.1186/s13023-018-0771-0>.

467. Amiñoso C, Gordillo-Marañón M, Hernández J, Solera J. Reevaluating the pathogenicity of the mutation c.1194 +5 G&gt;A in GAA gene by functional analysis of RNA in a 61-year-old woman diagnosed with Pompe disease by muscle biopsy. *Neuromuscular Disorders*. 2019;29(3):187-191. <https://doi.org10.1016/j.nmd.2018.12.003>.

468. Musumeci O, La Marca G, Spada M, et al. LOPED study: Looking for an early diagnosis in a late-onset Pompe disease high-risk population. *Journal of neurology, neurosurgery, and psychiatry*. 03/17 2015;87. <https://doi.org10.1136/jnnp-2014-310164>.

469. Semplicini C, Letard P, De Antonio M, et al. Late-onset Pompe disease in France: molecular features and epidemiology from a nationwide study. *Journal of Inherited Metabolic Disease*. 2018;41(6):937-946. <https://doi.orghttps://doi.org/10.1007/s10545-018-0243-7>.

470. Joshi PR, Gläser D, Schmidt S, et al. Molecular diagnosis of German patients with late-onset glycogen storage disease type II. *Journal of Inherited Metabolic Disease*. 2008;31(S2):261-265. <https://doi.orghttps://doi.org/10.1007/s10545-008-0820-2>.

471. Gallardo E, de Luna N, Diaz-Manera J, et al. Comparison of dysferlin expression in human skeletal muscle with that in monocytes for the diagnosis of dysferlin myopathy. *PLoS One*. 2011;6(12):e29061-e29061. <https://doi.org10.1371/journal.pone.0029061>.

472. Shin YS. Glycogen Storage Disease: Clinical, Biochemical, and Molecular Heterogeneity. *Seminars in Pediatric Neurology*. 2006/06/01/ 2006;13(2):115-120. <https://doi.orghttps://doi.org/10.1016/j.spen.2006.06.007>.

473. Wan L, Lee CC, Hsu CM, et al. Identification of eight novel mutations of the acid α-glucosidase gene causing the infantile or juvenile form of glycogen storage disease type II. *Journal of Neurology*. 2008/06/01 2008;255(6):831-838. <https://doi.org10.1007/s00415-008-0714-0>.

474. Kroos M, Hoogeveen-Westerveld M, Michelakakis H, et al. Update of the pompe disease mutation database with 60 novel GAA sequence variants and additional studies on the functional effect of 34 previously reported variants. *Human Mutation*. 2012;33(8):1161-1165. <https://doi.orghttps://doi.org/10.1002/humu.22108>.

475. Pittis M, Donnarumma M, Montalvo A, et al. Molecular and functional characterization of eight novel GAA mutations in Italian infants with Pompe disease. *Human mutation*. 06/01 2008;29:E27-36. <https://doi.org10.1002/humu.20753>.

476. Montalvo ALE, Bembi B, Donnarumma M, et al. Mutation profile of the GAA gene in 40 Italian patients with late onset glycogen storage disease type II. *Human Mutation*. 2006;27(10):999-1006. <https://doi.orghttps://doi.org/10.1002/humu.20374>.

477. Palmer RE, Amartino HM, Niizawa G, Blanco M, Pomponio RJ, Chamoles NA. Pompe disease (glycogen storage disease type II) in Argentineans: Clinical manifestations and identification of 9 novel mutations. *Neuromuscular Disorders*. 2007;17(1):16-22. <https://doi.org10.1016/j.nmd.2006.09.004>.

478. Mori M, Haskell G, Kazi Z, et al. Sensitivity of whole exome sequencing in detecting infantile- and late-onset Pompe disease. *Molecular Genetics and Metabolism*. 2017/12/01/ 2017;122(4):189-197. <https://doi.orghttps://doi.org/10.1016/j.ymgme.2017.10.008>.

479. Sampaolo S, Esposito T, Farina O, et al. Distinct disease phenotypes linked to different combinations of GAA mutations in a large late-onset GSDII sibship. *Orphanet Journal of Rare Diseases*. 2013/10/10 2013;8(1):159. <https://doi.org10.1186/1750-1172-8-159>.

480. van Capelle CI, van der Beek NAME, Hagemans MLC, et al. Effect of enzyme therapy in juvenile patients with Pompe disease: A three-year open-label study. *Neuromuscular Disorders*. 2010;20(12):775-782. <https://doi.org10.1016/j.nmd.2010.07.277>.

481. Stroppiano M, Bonuccelli G, Corsolini F, Filocamo M. Aberrant splicing at catalytic site as cause of infantile onset glycogen storage disease type II (GSDII): Molecular identification of a novel IVS9 (+2GT -> GC) in combination with rare IVS10 (+1GT -> CT). *Am J Med Genet*. 06/01 2001;101:55-8. <https://doi.org10.1002/ajmg.1310>.

482. Shinjo S, Silva R, Andrade F, et al. Pompe disease in a Brazilian series: Clinical and molecular analyses with identification of nine new mutations. *Journal of neurology*. 07/01 2009;256:1881-90. <https://doi.org10.1007/s00415-009-5219-y>.

483. Labrousse P, Chien Y-H, Pomponio RJ, et al. Genetic heterozygosity and pseudodeficiency in the Pompe disease newborn screening pilot program. *Molecular Genetics and Metabolism*. 2010/04/01/ 2010;99(4):379-383. <https://doi.orghttps://doi.org/10.1016/j.ymgme.2009.12.014>.

484. Huie ML, Anyane-Yeboa K, Guzman E, Hirschhorn R. Homozygosity for Multiple Contiguous Single-Nucleotide Polymorphisms as an Indicator of Large Heterozygous Deletions: Identification of a Novel Heterozygous 8-kb Intragenic Deletion (IVS7&#x2013;19 to IVS15&#x2013;17) in a Patient with Glycogen Storage Disease Type II. *The American Journal of Human Genetics*. 2002;70(4):1054-1057. <https://doi.org10.1086/339691>.

485. Remiche G, Ronchi D, Magri F, et al. Extended phenotype description and new molecular findings in late onset glycogen storage disease type II: a northern Italy population study and review of the literature. *Journal of Neurology*. 2014/01/01 2014;261(1):83-97. <https://doi.org10.1007/s00415-013-7137-2>.

486. Kishnani PS, Goldenberg PC, DeArmey SL, et al. Cross-reactive immunologic material status affects treatment outcomes in Pompe disease infants. *Molecular Genetics and Metabolism*. 2010/01/01/ 2010;99(1):26-33. <https://doi.orghttps://doi.org/10.1016/j.ymgme.2009.08.003>.

487. Nilsson MI, Kroos MA, Reuser AJ, et al. Novel GAA sequence variant c.1211 A>G reduces enzyme activity but not protein expression in infantile and adult onset Pompe disease. *Gene*. 2014/03/01/ 2014;537(1):41-45. <https://doi.orghttps://doi.org/10.1016/j.gene.2013.12.033>.

488. Huang H-P, Chen P-H, Hwu W-L, et al. Human Pompe disease-induced pluripotent stem cells for pathogenesis modeling, drug testing and disease marker identification. *Human Molecular Genetics*. 2011;20(24):4851-4864. <https://doi.org10.1093/hmg/ddr424>.

489. Adams EM, Becker JA, Griffith L, Segal A, Plotz PH, Raben N. Glycogenosis type II: A juvenile-specific mutation with an unusual splicing pattern and a shared mutation in African Americans. *Human Mutation*. 1997;10(2):128-134. <https://doi.orghttps://doi.org/10.1002/(SICI)1098-1004(1997)10:2><128::AID-HUMU5>3.0.CO;2-G.

490. Kroos M, Manta P, Mavridou I, et al. Seven cases of Pompe disease from Greece. *Journal of Inherited Metabolic Disease*. 2006;29(4):556-563. <https://doi.orghttps://doi.org/10.1007/s10545-006-0280-5>.

491. Nazari F, Sinaei F, Nilipour Y, et al. Late‐onset pompe disease in Iran: A clinical and genetic report. *Muscle & nerve*. 2017;55(6):835-840.

492. Bergsma AJ, Kroos M, Hoogeveen‐Westerveld M, Halley D, van der Ploeg AT, Pijnappel W. Identification and Characterization of Aberrant GAA Pre‐m RNA Splicing in P ompe Disease Using a Generic Approach. *Human mutation*. 2015;36(1):57-68.

493. Montagnese F, Barca E, Musumeci O, et al. Clinical and molecular aspects of 30 patients with late-onset Pompe disease (LOPD): unusual features and response to treatment. *Journal of neurology*. 2015;262(4):968-978.

494. Pérez-López J, Selva-O'Callaghan A, Grau-Junyent JM, et al. Delayed diagnosis of late-onset Pompe disease in patients with myopathies of unknown origin and/or hyperCKemia. *Molecular genetics and metabolism*. 2015;114(4):580-583.

495. Loeffen J, Elpeleg O, Smeitink J, et al. Mutations in the complex I NDUFS2 gene of patients with cardiomyopathy and encephalomyopathy. *Annals of Neurology: Official Journal of the American Neurological Association and the Child Neurology Society*. 2001;49(2):195-201.

496. Romano AA, Allanson JE, Dahlgren J, et al. Noonan syndrome: clinical features, diagnosis, and management guidelines. *Pediatrics*. 2010;126(4):746-759.

497. Johnston JJ, van der Smagt JJ, Rosenfeld JA, et al. Autosomal recessive Noonan syndrome associated with biallelic LZTR1 variants. *Genetics in Medicine*. 2018/10/01 2018;20(10):1175-1185. <https://doi.org10.1038/gim.2017.249>.

498. Böhm M, Pronicka E, Karczmarewicz E, et al. Retrospective, multicentric study of 180 children with cytochrome C oxidase deficiency. *Pediatric research*. 2006;59(1):21.

499. Chiang H-L, Wang NH-H, Song I-W, et al. Genetic epidemiological study doesn't support GLA IVS4+ 919G> A variant is a significant mutation in Fabry disease. *Molecular genetics and metabolism*. 2017;121(1):22-27.

500. Ishii S, Nakao S, Minamikawa-Tachino R, Desnick RJ, Fan J-Q. Alternative Splicing in the &#x3b1;-Galactosidase A Gene: Increased Exon Inclusion Results in the Fabry Cardiac Phenotype. *The American Journal of Human Genetics*. 2002;70(4):994-1002. <https://doi.org10.1086/339431>.

501. Gremer L, Luca A, Merbitz-Zahradnik T, et al. Duplication of Glu37 in the switch I region of HRAS impairs effector/GAP binding and underlies Costello syndrome by promoting enhanced growth factor-dependent MAPK and AKT activation. *Human molecular genetics*. 12/01 2009;19:790-802. <https://doi.org10.1093/hmg/ddp548>.

502. Teng Y, Wang T, Hwu W, Lin S, Lee-Chen G. Identification and characterization of -3c–g acceptor splice site mutation in human α- l-iduronidase associated with mucopolysaccharidosis type IH/S [<https://doi.org/10.1034/j.1399-0004.2000.570207.x>]. *Clinical Genetics*. 2000/02/01 2000;57(2):131-136. <https://doi.orghttps://doi.org/10.1034/j.1399-0004.2000.570207.x>.

503. Hashida Y, Wada T, Saito T, Ohta K, Kasahara Y, Yachie A. Early diagnosis of Danon disease: Flow cytometric detection of lysosome-associated membrane protein-2-negative leukocytes. *Journal of cardiology*. 2015;66(2):168-174.

504. Boucek D, Jirikowic J, Taylor M. Natural history of Danon disease. *Genetics in Medicine*. 2011/06/01 2011;13(6):563-568. <https://doi.org10.1097/GIM.0b013e31820ad795>.

505. Horváth J, Ketelsen UP, Geibel-Zehender A, et al. Identification of a Novel LAMP2 Mutation Responsible for X-Chromosomal Dominant Danon Disease. *Neuropediatrics*. //

04.11.2003 2003;34(05):270-273.

506. Nishino I, Fu J, Tanji K, et al. Primary LAMP-2 deficiency causes X-linked vacuolar cardiomyopathy and myopathy (Danon disease). *Nature*. 2000/08/01 2000;406(6798):906-910. <https://doi.org10.1038/35022604>.

507. Arad M, Maron BJ, Gorham JM, et al. Glycogen Storage Diseases Presenting as Hypertrophic Cardiomyopathy. *New England Journal of Medicine*. 2005;352(4):362-372. <https://doi.org10.1056/NEJMoa033349>.

508. Rigaud C, Lebre A-S, Touraine R, et al. Natural history of Barth syndrome: a national cohort study of 22 patients. *Orphanet Journal of Rare Diseases*. 2013/05/08 2013;8(1):70. <https://doi.org10.1186/1750-1172-8-70>.

509. Van Maldergem L. Berardinelli-Seip Congenital Lipodystrophy. In: Adam MP, Ardinger HH, Pagon RA, Wallace SE, Bean LJH, Stephens K, Amemiya A, eds. *GeneReviews(®)*. Seattle (WA): University of Washington, Seattle

Copyright © 1993-2020, University of Washington, Seattle. GeneReviews is a registered trademark of the University of Washington, Seattle. All rights reserved.; 1993.

510. Heathcote K, Rajab A, Magré J, et al. Molecular Analysis of Berardinelli-Seip Congenital Lipodystrophy in Oman. *Evidence for Multiple Loci*. 2002;51(4):1291-1293. <https://doi.org10.2337/diabetes.51.4.1291>.

511. Magré J, Delépine M, Khallouf E, et al. Identification of the gene altered in Berardinelli–Seip congenital lipodystrophy on chromosome 11q13. *Nature Genetics*. 2001/08/01 2001;28(4):365-370. <https://doi.org10.1038/ng585>.

512. Theis JL, Bos JM, Bartleson VB, et al. Echocardiographic-determined septal morphology in Z-disc hypertrophic cardiomyopathy. *Biochem Biophys Res Commun*. 2006/12/29/ 2006;351(4):896-902. <https://doi.orghttps://doi.org/10.1016/j.bbrc.2006.10.119>.

513. Antonicka H, Mattman A, Carlson CG, et al. Mutations in <em>COX15</em> Produce a Defect in the Mitochondrial Heme Biosynthetic Pathway, Causing Early-Onset Fatal Hypertrophic Cardiomyopathy. *The American Journal of Human Genetics*. 2003;72(1):101-114. <https://doi.org10.1086/345489>.

514. Schiff M, Froissart R, Olsen RKJ, Acquaviva C, Vianey-Saban C. Electron transfer flavoprotein deficiency: Functional and molecular aspects. *Molecular Genetics and Metabolism*. 2006/06/01/ 2006;88(2):153-158. <https://doi.orghttps://doi.org/10.1016/j.ymgme.2006.01.009>.

515. Olsen RKJ, Pourfarzam M, Morris AAM, et al. Lipid-storage myopathy and respiratory insufficiency due to ETFQO mutations in a patient with late-onset multiple acyl-CoA dehydrogenation deficiency. *Journal of Inherited Metabolic Disease*. 2004;27(5):671-678. <https://doi.orghttps://doi.org/10.1023/B:BOLI.0000042986.10291.e9>.

516. Andersen PS, Havndrup O, Bundgaard H, et al. Myosin light chain mutations in familial hypertrophic cardiomyopathy: phenotypic presentation and frequency in Danish and South African populations. *Journal of Medical Genetics*. 2001;38(12):e43-e43. <https://doi.org10.1136/jmg.38.12.e43>.

517. Davit-Spraul A, Piraud M, Dobbelaere D, et al. Liver glycogen storage diseases due to phosphorylase system deficiencies: Diagnosis thanks to non invasive blood enzymatic and molecular studies. *Molecular Genetics and Metabolism*. 2011/09/01/ 2011;104(1):137-143. <https://doi.orghttps://doi.org/10.1016/j.ymgme.2011.05.010>.

518. Burwinkel B, Bakker HD, Herschkovitz E, Moses SW, Shin YS, Kilimann MW. Mutations in the Liver Glycogen Phosphorylase Gene (<em>PYGL</em>) Underlying Glycogenosis Type VI (Hers Disease). *The American Journal of Human Genetics*. 1998;62(4):785-791. <https://doi.org10.1086/301790>.

519. Chang S, Rosenberg MJ, Morton H, Francomano CA, Biesecker LG. Identification of a Mutation in Liver Glycogen Phosphorylase in Glycogen Storage Disease Type VI. *Human Molecular Genetics*. 1998;7(5):865-870. <https://doi.org10.1093/hmg/7.5.865>.

520. Chen R, Tsuji T, Ichida F, et al. Mutation analysis of the G4.5 gene in patients with isolated left ventricular noncompaction. *Molecular Genetics and Metabolism*. 2002/12/01/ 2002;77(4):319-325. <https://doi.orghttps://doi.org/10.1016/S1096-7192(02)00195-6>.

521. Sakamoto O, Ohura T, Katsushima Y, et al. A novel intronic mutation of the TAZ (G4.5) gene in a patient with Barth syndrome: creation of a 5' splice donor site with variant GC consensus and elongation of the upstream exon. *Human Genetics*. 2001/11/01 2001;109(5):559-563. <https://doi.org10.1007/s00439-001-0612-3>.

522. Bione S, D'Adamo P, Maestrini E, Gedeon AK, Bolhuis PA, Toniolo D. A novel X-linked gene, G4.5. is responsible for Barth syndrome. *Nature Genetics*. 1996/04/01 1996;12(4):385-389. <https://doi.org10.1038/ng0496-385>.

523. Xing Y, Ichida F, Matsuoka T, et al. Genetic analysis in patients with left ventricular noncompaction and evidence for genetic heterogeneity. *Molecular Genetics and Metabolism*. 2006/05/01/ 2006;88(1):71-77. <https://doi.orghttps://doi.org/10.1016/j.ymgme.2005.11.009>.

524. Kühnisch J, Herbst C, Al-Wakeel-Marquard N, et al. Targeted panel sequencing in pediatric primary cardiomyopathy supports a critical role of TNNI3. *Clinical Genetics*. 2019;96(6):549-559. <https://doi.orghttps://doi.org/10.1111/cge.13645>.

525. Lopes LR, Barbosa P, Torrado M, et al. Cryptic Splice-Altering Variants in <i>MYBPC3</i> Are a Prevalent Cause of Hypertrophic Cardiomyopathy. *Circulation: Genomic and Precision Medicine*. 2020;13(3):e002905. <https://doi.orgdoi:10.1161/CIRCGEN.120.002905>.

526. Fan L-L, Huang H, Jin J-Y, et al. Whole exome sequencing identifies a novel mutation (c. 333+ 2T> C) of TNNI3K in a Chinese family with dilated cardiomyopathy and cardiac conduction disease. *Gene*. 2018;648:63-67.

527. van Rijsingen IA, Nannenberg EA, Arbustini E, et al. Gender‐specific differences in major cardiac events and mortality in lamin A/C mutation carriers. *European journal of heart failure*. 2013;15(4):376-384.

528. Rogozhina Y, Mironovich S, Shestak A, et al. New intronic splicing mutation in the LMNA gene causing progressive cardiac conduction defects and variable myopathy. *Gene*. 2016;595(2):202-206.

529. Lee H, Deignan JL, Dorrani N, et al. Clinical Exome Sequencing for Genetic Identification of Rare Mendelian Disorders. *JAMA*. 2014;312(18):1880-1887. <https://doi.org10.1001/jama.2014.14604>.

530. Chen C-H, Tang S-C, Su Y-N, Yang C-C, Jeng J-S. Cardioembolic stroke related to limb-girdle muscular dystrophy 1B. *BMC Research Notes*. 2013/01/29 2013;6(1):32. <https://doi.org10.1186/1756-0500-6-32>.

531. van Rijsingen IAW, Nannenberg EA, Arbustini E, et al. Gender-specific differences in major cardiac events and mortality in lamin A/C mutation carriers. *European Journal of Heart Failure*. 2013;15(4):376-384. <https://doi.orghttps://doi.org/10.1093/eurjhf/hfs191>.

532. Chrestian N, Valdmanis P, Echahidi N, et al. A novel mutation in a large French–Canadian family with LGMD1B. *The Canadian journal of neurological sciences Le journal canadien des sciences neurologiques*. 08/01 2008;35:331-4. <https://doi.org10.1017/S031716710000891X>.

533. OTOMO J, KURE S, SHIBA T, et al. Electrophysiological and Histopathological Characteristics of Progressive Atrioventricular Block Accompanied by Familial Dilated Cardiomyopathy Caused by a Novel Mutation of Lamin A/C Gene. *Journal of Cardiovascular Electrophysiology*. 2005;16(2):137-145. <https://doi.orghttps://doi.org/10.1046/j.1540-8167.2004.40096.x>.

534. Fatkin D, MacRae C, Sasaki T, et al. Missense Mutations in the Rod Domain of the Lamin A/C Gene as Causes of Dilated Cardiomyopathy and Conduction-System Disease. *New England Journal of Medicine*. 1999;341(23):1715-1724. <https://doi.org10.1056/nejm199912023412302>.

535. Yaou R, Bécane HM, Demay L, et al. Autosomal dominant limb-girdle muscular dystrophy associated with conduction defects (LGMD1B): A description of 8 new families with the LMNA gene mutations. *Revue neurologique*. 02/01 2005;161:42-54.

536. van Spaendonck-Zwarts KY, van Rijsingen IAW, van den Berg MP, et al. Genetic analysis in 418 index patients with idiopathic dilated cardiomyopathy: overview of 10 years' experience. *European Journal of Heart Failure*. 2013;15(6):628-636. <https://doi.orghttps://doi.org/10.1093/eurjhf/hft013>.

537. Muchir A, Bonne G, van der Kooi AJ, et al. Identification of mutations in the gene encoding lamins A/C in autosomal dominant limb girdle muscular dystrophy with atrioventricular conduction disturbances (LGMD1B). *Human Molecular Genetics*. 2000;9(9):1453-1459. <https://doi.org10.1093/hmg/9.9.1453>.

538. Hoorntje ET, van Spaendonck‐Zwarts KY, Te Rijdt WP, et al. The first titin (c. 59926+ 1G> A) founder mutation associated with dilated cardiomyopathy. *European journal of heart failure*. 2018;20(4):803-806.

539. Akinrinade O, Koskenvuo JW, Alastalo T-P. Prevalence of Titin Truncating Variants in General Population. *PLoS One*. 2015;10(12):e0145284-e0145284. <https://doi.org10.1371/journal.pone.0145284>.

540. Ceyhan-Birsoy O, Agrawal PB, Hidalgo C, et al. Recessive truncating titin gene, <em>TTN</em>, mutations presenting as centronuclear myopathy. *Neurology*. 2013;81(14):1205-1214. <https://doi.org10.1212/WNL.0b013e3182a6ca62>.

541. Herman DS, Lam L, Taylor MRG, et al. Truncations of Titin Causing Dilated Cardiomyopathy. *New England Journal of Medicine*. 2012;366(7):619-628. <https://doi.org10.1056/NEJMoa1110186>.

542. Horvat C, Johnson R, Lam L, et al. A gene-centric strategy for identifying disease-causing rare variants in dilated cardiomyopathy. *Genetics in Medicine*. 2019/01/01 2019;21(1):133-143. <https://doi.org10.1038/s41436-018-0036-2>.

543. Vissing C, Rasmussen T, Dybro A, et al. Dilated cardiomyopathy caused by truncating titin variants: long-term outcomes, arrhythmias, response to treatment and sex differences. *Journal of Medical Genetics*. 10/26 2020:jmedgenet-2020. <https://doi.org10.1136/jmedgenet-2020-107178>.

544. Hazebroek MR, Krapels I, Verdonschot J, et al. Prevalence of Pathogenic Gene Mutations and Prognosis Do Not Differ in Isolated Left Ventricular Dysfunction Compared With Dilated Cardiomyopathy. *Circulation: Heart Failure*. 2018;11(3):e004682. <https://doi.orgdoi:10.1161/CIRCHEARTFAILURE.117.004682>.

545. Haas J, Frese K, Peil B, et al. Atlas of the clinical genetics of human dilated cardiomyopathy. *European Heart Journal*. 08/27 2014;36. <https://doi.org10.1093/eurheartj/ehu301>.

546. O'Grady GL, Lek M, Lamande SR, et al. Diagnosis and etiology of congenital muscular dystrophy: We are halfway there. *Annals of Neurology*. 2016;80(1):101-111. <https://doi.orghttps://doi.org/10.1002/ana.24687>.

547. Zhang C, Zhang H, Wu G, et al. Titin-Truncating Variants Increase the Risk of Cardiovascular Death in Patients With Hypertrophic Cardiomyopathy. *Canadian Journal of Cardiology*. 2017;33(10):1292-1297. <https://doi.org10.1016/j.cjca.2017.05.020>.

548. Harris E, Töpf A, Vihola A, et al. A &#x2018;second truncation&#x2019; in <em>TTN</em> causes early onset recessive muscular dystrophy. *Neuromuscular Disorders*. 2017;27(11):1009-1017. <https://doi.org10.1016/j.nmd.2017.06.013>.

549. Kang PB, Morrison L, Iannaccone ST, et al. Evidence-based guideline summary: evaluation, diagnosis, and management of congenital muscular dystrophy: report of the Guideline Development Subcommittee of the American Academy of Neurology and the Practice Issues Review Panel of the American Association of Neuromuscular & Electrodiagnostic Medicine. *Neurology*. 2015;84(13):1369-1378.

550. Straub V, Murphy A, Udd B, Group LWS. 229th ENMC international workshop: Limb girdle muscular dystrophies-Nomenclature and reformed classification Naarden, the Netherlands, 17-19 March 2017. *Neuromuscular disorders: NMD*. 2018;28(8):702.

551. Ojrzyńska N, Bilińska ZT, Franaszczyk M, Płoski R, Grzybowski J. Restrictive cardiomyopathy due to novel desmin gene mutation. *Kardiologia polska*. 2017;75(7):723. <https://doi.org10.5603/kp.2017.0129>.

552. Henderson M, De Waele L, Hudson J, et al. Recessive desmin-null muscular dystrophy with central nuclei and mitochondrial abnormalities. *Acta Neuropathologica*. 2013/06/01 2013;125(6):917-919. <https://doi.org10.1007/s00401-013-1113-x>.

553. Brunetti-Pierri N, Scaglia F. GM1 gangliosidosis: review of clinical, molecular, and therapeutic aspects. *Molecular genetics and metabolism*. 2008;94(4):391-396.

554. Chiriboga ASL. Teaching NeuroImages: Prominent spinal cord atrophy and white matter changes in adult polyglucosan body disease. *Neurology*. 2017;88(20):e194-e195.

555. Bruno C, van Diggelen OP, Cassandrini D, et al. Clinical and genetic heterogeneity of branching enzyme deficiency (glycogenosis type IV). *Neurology*. 2004;63(6):1053-1058. <https://doi.org10.1212/01.wnl.0000138429.11433.0d>.

556. Chong JX, Ouwenga R, Anderson RL, Waggoner DJ, Ober C. A population-based study of autosomal-recessive disease-causing mutations in a founder population. *The American Journal of Human Genetics*. 2012;91(4):608-620.

557. Pugh TJ, Kelly MA, Gowrisankar S, et al. The landscape of genetic variation in dilated cardiomyopathy as surveyed by clinical DNA sequencing. *Genetics in Medicine*. 2014/08/01 2014;16(8):601-608. <https://doi.org10.1038/gim.2013.204>.

558. Lim BC, Ki C-S, Kim J-W, et al. <em>Fukutin</em> mutations in congenital muscular dystrophies with defective glycosylation of dystroglycan in Korea. *Neuromuscular Disorders*. 2010;20(8):524-530. <https://doi.org10.1016/j.nmd.2010.06.005>.

559. Deo RC, Musso G, Tasan M, et al. Prioritizing causal disease genes using unbiased genomic features. *Genome Biology*. 2014/12/03 2014;15(12):534. <https://doi.org10.1186/s13059-014-0534-8>.

560. Begay RL, Tharp CA, Martin A, et al. FLNC Gene Splice Mutations Cause Dilated Cardiomyopathy. *JACC: Basic to Translational Science*. 2016/08/01/ 2016;1(5):344-359. <https://doi.orghttps://doi.org/10.1016/j.jacbts.2016.05.004>.

561. Allali S, Dorboz I, Samaan S, et al. Mutation in the AGK gene in two siblings with unusual Sengers syndrome. *Metabolic brain disease*. 2017;32(6):2149-2154.

562. Savarese M, Di Fruscio G, Tasca G, et al. Next generation sequencing on patients with LGMD and nonspecific myopathies: findings associated with ANO5 mutations. *Neuromuscular Disorders*. 2015;25(7):533-541.

563. Elting M, Kariminejad A, de Sonnaville ML, et al. Johanson–Blizzard syndrome caused by identical UBR1 mutations in two unrelated girls, one with a cardiomyopathy. *American Journal of Medical Genetics Part A*. 2008;146(23):3058-3061.

564. Majczenko K, Davidson AE, Camelo-Piragua S, et al. Dominant mutation of CCDC78 in a unique congenital myopathy with prominent internal nuclei and atypical cores. *The American Journal of Human Genetics*. 2012;91(2):365-371.

565. Evans M, Andresen BS, Nation J, Boneh A. VLCAD deficiency: follow-up and outcome of patients diagnosed through newborn screening in Victoria. *Molecular genetics and metabolism*. 2016;118(4):282-287.

566. Senefeld C, Senefeld J. Very long-chain acyl-CoA dehydrogenase deficiency nomenclature: compound heterozygosity. *Journal of Human Genetics*. 01/27 2020;65:1-2. <https://doi.org10.1038/s10038-020-0727-9>.

567. Siu W-K, Mak CM, Siu SL-Y, et al. Molecular Diagnosis for a Fatal Case of Very Long-chain Acyl-CoA Dehydrogenase Deficiency in Hong Kong Chinese With a Novel Mutation: A Preventable Death by Newborn Screening. *Diagnostic Molecular Pathology*. 2012;21(3):184-187. <https://doi.org10.1097/PDM.0b013e31825554d0>.

568. Andresen BS, Olpin S, Poorthuis BJHM, et al. Clear Correlation of Genotype with Disease Phenotype in Very&#x2013;Long-Chain Acyl-CoA Dehydrogenase Deficiency. *The American Journal of Human Genetics*. 1999;64(2):479-494. <https://doi.org10.1086/302261>.

569. Mellerio J, Pulkkinen L, McMillan J, et al. Pyloric atresia-junctional epidermolysis bullosa syndrome: mutations in the integrin beta4 gene (ITGB4) in two unrelated patients with mild disease. *The British journal of dermatology*. 1998;139(5):862-871.

570. Birnkrant DJ, Bushby KM, Amin RS, et al. The respiratory management of patients with Duchenne muscular dystrophy: a DMD care considerations working group specialty article. *Pediatric pulmonology*. 2010;45(8):739-748.

571. Santos R, Goncalves A, Oliveira J, et al. New variants, challenges and pitfalls in DMD genotyping: implications in diagnosis, prognosis and therapy. *Journal of human genetics*. 2014;59(8):454-464.

572. Cho A, Seong MW, Lim BC, et al. Consecutive analysis of mutation spectrum in the dystrophin gene of 507 Korean boys with Duchenne/Becker muscular dystrophy in a single center. *Muscle & nerve*. 2017;55(5):727-734.

573. Alame M, Lacourt D, Zenagui R, et al. Implementation of a reliable next-generation sequencing strategy for molecular diagnosis of dystrophinopathies. *The Journal of Molecular Diagnostics*. 2016;18(5):731-740.

574. Isik E, Onay H, Atik T, et al. Clinical utility of a targeted next generation sequencing panel in severe and pediatric onset Mendelian diseases. *European journal of medical genetics*. 2019;62(10):103725.

575. Niba ETE, Nishida A, Vu DC, et al. Cryptic splice activation but not exon skipping is observed in minigene assays of dystrophin c. 9361+ 1G> A mutation identified by NGS. *Journal of human genetics*. 2017;62(5):531-537.

576. Narayanaswami P. Dismantling limb-girdle muscular dystrophy: the role of whole-exome sequencing. *JAMA neurology*. 2015;72(12):1409-1411.

577. Zhong J, Xu T, Chen G, Liao H, Zhang J, Lan D. Genetic analysis of the dystrophin gene in children with Duchenne and Becker muscular dystrophies. *Muscle & nerve*. 2017;56(1):117-121.

578. Guo R, Zhu G, Zhu H, et al. DMD mutation spectrum analysis in 613 Chinese patients with dystrophinopathy. *Journal of human genetics*. 05/14 2015;60. <https://doi.org10.1038/jhg.2015.43>.

579. Okubo M, Minami N, Goto K, et al. Genetic diagnosis of Duchenne/Becker muscular dystrophy using next-generation sequencing: validation analysis of DMD mutations. *Journal of human genetics*. 2016;61(6):483-489.
[truncated: 100,890 more chars]
